# Downregulation of KEAP1 in melanoma promotes resistance to immune checkpoint blockade

**DOI:** 10.1038/s41698-023-00362-3

**Published:** 2023-03-02

**Authors:** Douglas B. Fox, Richard Y. Ebright, Xin Hong, Hunter C. Russell, Hongshan Guo, Thomas J. LaSalle, Ben S. Wittner, Nicolas Poux, Joanna A. Vuille, Mehmet Toner, Nir Hacohen, Genevieve M. Boland, Debattama R. Sen, Ryan J. Sullivan, Shyamala Maheswaran, Daniel A. Haber

**Affiliations:** 1 Massachusetts General Hospital Cancer Center and Harvard Medical School, Boston, MA 02114 USA; 2 Broad Institute of Harvard and MIT, Cambridge, MA 02139 USA; 3 Center for Engineering in Medicine, Massachusetts General Hospital and Harvard Medical School, Boston, MA 02114 USA; 4 Shriners Hospitals for Children, Boston, MA 02114 USA; 5 Department of Surgery, Massachusetts General Hospital and Harvard Medical School, Boston, MA 02114 USA; 6 Department of Medicine, Massachusetts General Hospital and Harvard Medical School, Boston, MA 02114 USA; 7 Howard Hughes Medical Institute, Bethesda, MD 20815 USA; 8 Present Address: Department of Biochemistry, School of Medicine and Key University Laboratory of Metabolism and Health of Guangdong, Southern University of Science and Technology, Shenzhen, 518055 China; 9 Present Address: Liangzhu Laboratory, Zhejiang University Medical Center, Hangzhou, 311121 China

**Keywords:** Cancer therapeutic resistance, Tumour biomarkers, Melanoma, Metastasis, Tumour heterogeneity

## Abstract

Immune checkpoint blockade (ICB) has demonstrated efficacy in patients with melanoma, but many exhibit poor responses. Using single cell RNA sequencing of melanoma patient-derived circulating tumor cells (CTCs) and functional characterization using mouse melanoma models, we show that the KEAP1/NRF2 pathway modulates sensitivity to ICB, independently of tumorigenesis. The NRF2 negative regulator, KEAP1, shows intrinsic variation in expression, leading to tumor heterogeneity and subclonal resistance.

The treatment of metastatic melanoma has been revolutionized by ICB, including antibodies that target programmed death receptor 1 (PD1) or its ligand PD-L1, to activate cytotoxic T cell killing of tumor cells, often resulting in durable and complete responses^1,2^. Following initial success in melanoma, ICB has been deployed against multiple other cancers, including lung, breast, and liver^3^. However, only a subset of cases achieves tumor eradication, underscoring the importance of understanding both intrinsic and acquired mechanisms of resistance. A number of baseline tumor markers appear to have predictive value for clinical response to ICB, including the extent of T cell infiltration^4,5^, expression of immune checkpoint molecules^2,4^, and tumor mutational burden^6^. In addition, somatically acquired genetic alterations can limit the efficacy of ICB through loss of antigen presentation or interferon gamma signaling^7,8^. While these studies have focused on genetic determinants of immune cell activation, less is known about the extent to which homeostatic cell mechanisms, including activation of adaptive stress response pathways, by tumor cells contributes to ICB sensitivity.

The isolation of whole CTCs from patient blood samples offers a potentially important non-invasive strategy to assess genetic mutations and gene expression changes associated with therapeutic response^9–11^. Furthermore, analysis of individual CTCs provides insight into tumor heterogeneity, including the potential treatment-induced selection of resistant clones. To this end, we used a microfluidic device (CTC-iChip) to deplete hematopoietic cells from blood specimens and enrich for untagged melanoma CTCs, using conditions optimized for single cell RNA sequencing (scRNAseq)^12^. CTCs were freshly isolated from 15 patients with metastatic melanoma, prior to or within 8 weeks of ICB treatment initiation, and subjected to scRNAseq. The identity of 47 CTCs was confirmed by expression of melanoma lineage markers as previously described^12^, and Response Evaluation Criteria in Solid Tumors (RECIST) criteria was used to classify patients as having complete response (CR; 4 patients), partial response (PR; 7 patients) or progressive disease (PD; 4 patients), assessed after 12 weeks of ICB initiation (8 patients received anti-PD1; 1 received anti-CTLA4 after progression on anti-PD1; 6 received dual therapy) (Supplementary Fig. 1A). Comparing single CTCs from patients with CR versus PD by differential expression analysis (Fig. 1a and Supplementary Fig. 1B), we find that expression of the antigen presentation gene HLA-A is substantially decreased in a subset of CTCs from PD patients (Fig. 1c), providing validation that the transcriptomic characterization of CTCs can detect known mechanisms of resistance to ICB^13^. Notably*, KEAP1* expression is elevated in CTCs from CR patients and undetectable in all but one CTC from patients with PD (Fig. 1b, c). *KEAP1* expression in CTCs is not correlated with a specific treatment regimen. KEAP1 is responsible for degradation of the stress response transcription factor NRF2^14^, and in contrast to KEAP1 levels, NRF2 target genes are highly expressed in CTCs from PD patients (Fig. 1a–c). Gene Set Enrichment Analysis (GSEA) of all transcripts demonstrates enrichment for the “NRF2 gene signature” in CTCs from PD patients (Fig. 1d). Additionally, the “Hallmark Reactive Oxygen Species”, “Hallmark Heme Metabolism” and “Hallmark Oxidative Phosphorylation” are other gene signatures significantly associated with PD (Supplementary Fig. 1C). Thus, loss of *KEAP1* expression and associated upregulation of NRF2 signaling within individual melanoma CTCs are associated with poor response to ICB in our CTC cohort.

**Fig. 1:**
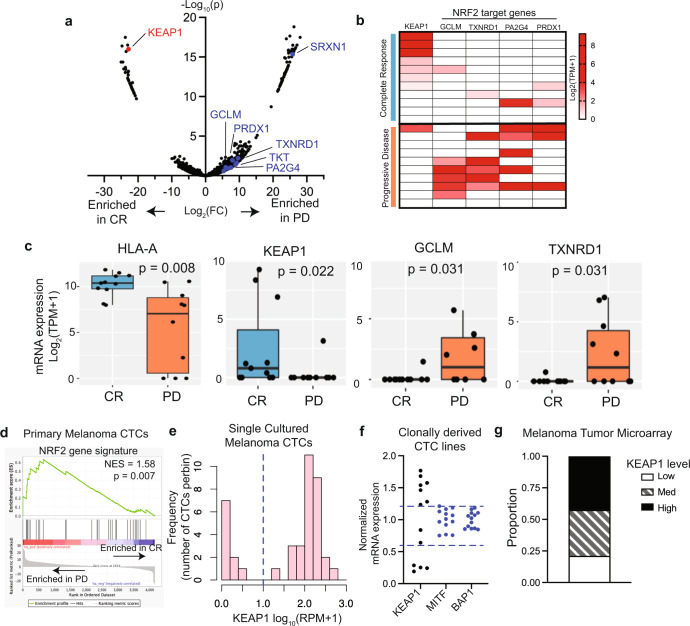
Low *KEAP1* expression is associated with poor response to immune checkpoint blockade. **a** Volcano plot showing baseline gene expression within patient-derived single CTCs correlated with subsequent ICB complete response (CR; 11 CTCs) or progressive disease (PD; 10 CTCs). *KEAP1* enriched in CR is labelled (red) and NRF2 target genes enriched in PD are labelled (blue). **b** Heatmap showing expression of *KEAP1* and the NRF2 target genes *GCLM, TXNRD1, PA2G4* and *PRDX1*, in primary CTCs derived from patients with complete response (CR) or progressive disease (PD) following ICB. **c** Bar graphs showing the baseline expression of *HLA-A*, *KEAP1*, and the NRF2 target genes *GCLM* and *TXNRD1*, in freshly isolated CTCs derived from patients with subsequent complete response (CR) or progressive disease (PD) following ICB. Line represents the median value, box spans 25th to 75th percentile, and whiskers span 5th to 95th percentile. *P* value determined by Wilcoxon rank sum test. **d** Gene set enrichment plots showing enrichment of the *NRF2* gene signature in CTCs from patients with PD. **e** Histogram showing single cell heterogeneity in KEAP1 mRNA expression across cultured CTCs, all isolated from melanoma patient MEL167. KEAP1 mRNA quantitated using scRNAseq (RPM). **f** Plot showing variability in mRNA expression of *KEAP1*, compared with the melanoma lineage markers *MITF* and *BAP1*, in single cell-derived clonal sublines of MEL167 CTCs. **g** Quantification of KEAP1 staining of melanoma tumor microarrays (*n* = 33 tumors). Tumors were categorized as high, medium, and low by setting intensity bins that span equal thirds of the total range of intensity measured.

To further analyze transcriptional downregulation of *KEAP1* in melanoma CTCs, we used scRNAseq of ex vivo cultured CTCs, derived from an oligoclonal population of CTCs from a single patient with metastatic melanoma (MEL167). We again find that *KEAP1* mRNA expression displays a striking degree of heterogeneity and a distinctly bimodal distribution, with some single tumor cells having abundant reads and others completely lacking in *KEAP1* expression (Fig. 1e). In agreement, staining for KEAP1 protein in these cultured CTCs shows high variability (Supplementary Fig. 2A). Differential *KEAP1* expression within CTCs is heritable, as demonstrated in 13 single cell-derived clonal sublines from the oligoclonal CTC culture, showing a 10-fold difference in the range of KEAP1 RNA expression (Fig. 1f). In comparison, expression of the melanoma lineage markers MITF and BAP1 is virtually identical across all 13 sublines (Fig. 1f). In addition to heterogeneous expression within melanoma CTCs, we find large variation in total KEAP1 expression across microarrays of human primary melanomas. (Fig. 1g and Supplementary Fig. 2B). Intra- and inter-tumor variation in KEAP1 expression thus appears to be a consistent feature in melanoma. Genetic mutations or promoter methylation are common mechanisms of *KEAP1* inactivation^14^, but these are not reported in melanoma. However, while we find methylation of the *KEAP1* promoter to be highly variable across single melanoma CTCs, neither promoter methylation nor intragenic methylation is correlated with expression of *KEAP1* mRNA at the single cell level (Supplementary Fig. 2C–F). Epigenetic changes in other *KEAP1* regulatory sequences or cofactors, or changes in *KEAP1* mRNA stability, are thus likely to drive heterogeneity of *KEAP1* expression in melanoma.

We next asked if *KEAP1* expression is associated with immunological phenotypes in melanoma samples, using GSEA for genes ranked by their correlation with *KEAP1* expression. Notably, the “Hallmark Interferon Gamma Response” and “Hallmark TNFA Signaling via NFKB” gene signatures correlated with *KEAP1* expression in melanoma CTCs (Fig. 2a) and in tumor samples and cell lines from the Cancer Genome Atlas (TCGA, Supplementary Fig. 3A, B). These signatures annotate interactions between tumor cells and microenvironment, through expression of cytokines and cell surface proteins^15^, raising the possibility that *KEAP1* expression within tumor cells may modulate microenvironmental signals. Indeed, cibersort^16^ analysis to infer immune cell populations within TCGA tumors indicates that cytotoxic CD8 T cells, regulatory T cells, and activated natural killer cell populations are significantly decreased in tumors with low expression of *KEAP1* (Fig. 2b). This effect is likely indirect, since siRNA-mediated *KEAP1* knockdown in melanoma CTCs and CRISPR-mediated knockout of *KEAP1* in B16-F10 mouse melanoma cells expressing ovalbumin (B16-OVA) has no effect on baseline or interferon gamma (IFNγ) induced expression of key immune markers such as PD-L1 and HLA antigen presentation molecules (Supplementary Fig. 3C–H).

**Fig. 2:**
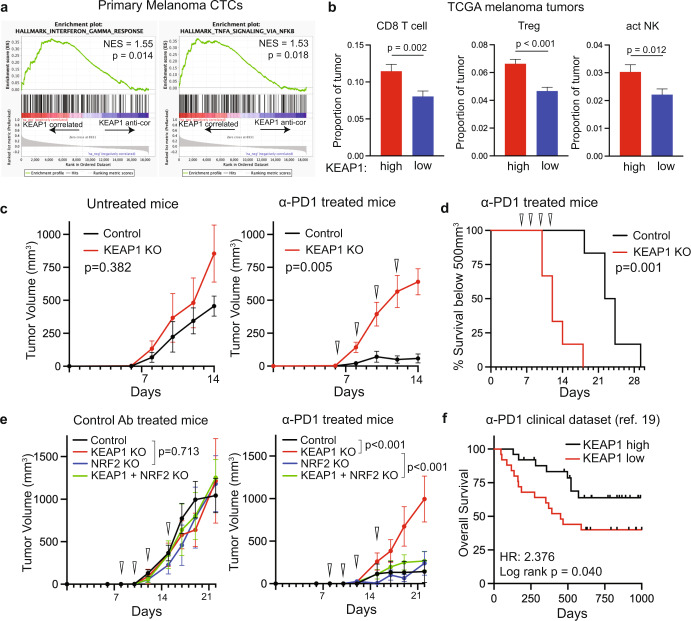
*KEAP1* loss induces resistance to anti-PD1 therapy. **a** GSEA plots showing enrichment for “Hallmark Interferon Gamma Response” and “Hallmark TNFA Signaling via NFKB” signatures correlated with *KEAP1* expression in freshly isolated melanoma CTCs. **b** Plots showing abundance of CD8 T cells, Regulatory T cells (Treg), and activated natural killer cells (act NK) inferred by cibersort analysis in TCGA melanoma tumors with high (top third, *n* = 155) and low (bottom third, *n* = 155) *KEAP1* mRNA expression. Significance determined by Student’s *t* test. **c** Tumor growth curves generated from control and *KEAP1* knockout B16-OVA cells, treated with anti-PD1 antibody (α-PD1) or untreated. Triangles denote days mice received treatment. Significance determined by two-way ANOVA (*n* = 6 per condition). **d** Kaplan–Meier (KM) survival plot for anti-PD1 treated mice from panel **c**. Mouse survival is derived from sacrifice once tumors reached 500 mm^3^ in size. Significance determined by log-rank (Mantel-Cox) test (*n* = 6 per condition). **e** Tumor growth curves generated from control, *KEAP1* knockout, *NRF2* knockout, and *KEAP1/NRF2* double knockout B16-OVA cells, treated with anti-PD1 antibody or matched isotype control antibody (control Ab). Triangles denote days mice received treatment. Significance determined by two-way ANOVA (*n* = 6 per condition). **f** KM plot for overall survival for anti-PD1 treated melanoma patients with high (top third, *n* = 25) and low (bottom third, *n* = 25) *KEAP1* mRNA expression in their tumors. Significance determined by log-rank (Mantel-Cox) test. Data derived from Liu et al. (ref. ^19^). Data are represented as mean ± SEM.

To model the functional impact of *KEAP1* expression on melanoma tumor growth and response to immune checkpoint inhibition in vivo, we used the well-established B16-OVA model, in which anti-PD1 antibody treatment mediates a potent anti-tumor response^17^. We achieved CRISPR-mediated knockout of *KEAP1* in these cells, with a concordant induction of NRF2 and its canonical target genes (Supplementary Fig. 3e, f). Following subcutaneous implantation and generation of palpable tumors into fully immunocompetent C57BL/6 J mice, anti-PD1 was administered every other day, for a total of four treatments in one cohort of mice with another cohort left untreated. Remarkably, loss of *KEAP1* completely abrogated the potent response to anti-PD1 treatment, measured both by tumor size and mouse survival (Fig. 2c, d and Supplementary Fig. 4A). Since KEAP1 has been shown to regulate stability of cellular proteins in addition to NRF2^18^, we tested whether simultaneous CRISPR-mediated knockout of *NRF2* and *KEAP1* in B16-OVA cells (Supplementary Fig. 4B) is sufficient to rescue the *KEAP1-*null phenotype. Whereas *NRF2*-knockout alone does not affect the response of tumors to anti-PD1, when combined with *KEAP1* loss in the double knockout B16-OVA cells, it completely abrogates *KEAP1* knockout-mediated resistance to anti-PD1 treatment (Fig. 2e and Supplementary Fig. 4C). Thus, the effect of *KEAP1* expression on ICB is mediated through its regulation of NRF2 activity.

The induction of stress pathway modulators by NRF2 has been implicated as a contributor to tumor progression in a number of cancers^14^. However in B16-OVA melanoma cells, *NRF2* knockout alone does not significantly inhibit primary tumor growth in the absence of anti-PD1 (Fig. 2e), nor does *KEAP1* knockout enhance tumor growth in the absence of treatment (Fig. 2c, e and Supplementary Fig. 4A). However, the highly effective anti-tumor response in this ICB model might limit detection of potentially enhanced immune response in *NRF2* knockout tumors treated with anti-PD1. To extend these studies to clinical samples from melanoma patients treated with ICB, we interrogated publicly available datasets from Liu et al.^19^, including RNAseq from 74 ICB-naïve patients with advanced melanoma treated with antibody against PD1. Patients with tumors having low expression of *KEAP1* have a worse Overall Survival, compared with those with high *KEAP1* (Fig. 2f). Thus, loss of *KEAP1* expression contributes toward resistance to ICB in melanoma, both in mouse models and patient datasets, and the impact of both KEAP1 loss and NRF2 activation should be further interrogated as larger clinical datasets are generated.

The KEAP1-NRF2 pathway is well established as a major regulator of the cellular response to oxidative and metabolic stress, contributing to cancer progression and resistance to chemotherapies and targeted therapies. Our analysis demonstrates that it also constitutes a mechanism of resistance to ICB, distinct from the characteristic alterations in antigen presentation pathways identified to date. Indeed, the stabilization of NRF2 resulting from *KEAP1* knockout is sufficient to completely ablate the sensitivity of mouse melanomas to anti-PD1 treatment. The link between *KEAP1* loss and altered cytokine expression is supported by interactions between NRF2 and NF-κB^14^, and our findings in melanoma are consistent with a preliminary report showing that *KEAP1* mutations cause resistance to anti-PD1 treatment in a transgenic mouse model of lung cancer^20^. Most significantly, our study of patient-derived single CTC cultures points to heterogeneity in *KEAP1* expression within individual tumors as an important variable in ICB. Taken together, our results highlight the critical impact of KEAP1/NRF2 signaling in the response to immunotherapy in melanoma, one of the most highly immunotherapy responsive cancers, together with the contribution of intrinsic heterogeneity in fundamental homeostatic cellular pathways that modulate such therapeutic responses.

## Methods

### Plasmids, cloning, and viral transduction

Small guide RNA sequences from the Broad Institute genome-wide Brunello library were used^21^, and oligos were designed following the GeCKO protocol^22,23^. Control and Keap1 targeting small guide RNAs (sgCtl and sgKeap1 #1; see Supplementary Table 2) were cloned into the LentiCRISPRv2 puro backbone using golden gate assembly^24^. Rosa26, Keap1, and Nfe2l2 (NRF2) targeting small guide RNAs (sgRosa, sgKeap1 #2, and sgNfe2l2) were cloned into the LentiGuide hygro backbone using golden gate assembly^24^. LentiCRISPRv2 puro was a gift from Brett Stringer (Addgene #98290)^25^, and LentiGuide-hygro was a gift from Rizwan Haq (Addgene #160090)^26^. To generate lentivirus, HEK293T cells were transfected with psPAX2 and pMDG.2 packaging plasmids (gifts from Didier Trono, EPFL, Lausanne, Switzerland; Addgene plasmids #12559 and #12660) and the lentiviral expression construct. Viral supernatant was collected after 48 and 72 h and filtered. This virus was used to transduce B16-OVA cells with 6 µg/mL polybrene (EMD Millipore).

### Cell lines

B16-OVA cells were generated using lentiviral transduction to express chicken ovalbumin under the PGK promoter^17,27^. These were cultured in DMEM (Gibco) with 10% FBS and 1X Pen/Strep (Gibco). To assess the effect of KEAP1 loss on response to anti-PD1 (Fig. 2c, d), control cells were transduced to express Cas9 and sgCtl with lentiCRISPR v2. KEAP1 knockout cells were expressing sgKeap1 #1. To assess the effect of KEAP1 and NRF2 double knockout (Fig. 2e), control cells were transduced to express Cas9 and sgCtl with lentiCRISPR v2 puro and sgRosa with LentiGuide-hygro. KEAP1 knockout cells were transduced to express Cas9 and sgKeap1 #2 with lentiCRISPRv2 puro and sgRosa with LentiGuide-hygro. KEAP1/NRF2 double knockout cells were transduced to express Cas9 and sgKeap1 #2 with lentiCRISPRvs puro and sgNfe2l2 with LentiGuide-hygro. See “Plasmids, cloning, and viral transduction” section for small guide design and cloning details. See Supplementary Table 2 for small guide RNA sequences. HEK293T were obtained from the ATCC and maintained per ATCC methods. Cell lines were routinely checked for mycoplasma (MycoAlert, Lonza).

### qRT-PCR and western blot analyses

For qRT-PCR analysis, RNA was extracted using the Qiagen RNeasy minikit and reverse transcribed using Superscript IV VILO (Invitrogen). qRT-PCR was performed using SYBR green (Applied biosystems), and gene expression was normalized to GAPDH expression. See Supplementary Table 2 for primer sequences. For western blot analysis, whole cell lysates were prepared with RIPA buffer (Sigma). Proteins were separated by electrophoresis using 4–15% polyacrylamide gradient-SDS gels (Bio-Rad), and transferred onto Nitrocellulose membrane (Bio-Rad). Immunoblots probed for KEAP1 (Abcam ab227828), NRF2 (Cell Signaling Technologies #12721), and Tubulin (Sigma #T9026) were imaged using enhanced chemiluminescence (EMD Millipore). Antibody dilutions are included in Supplementary Table 2. Western blots within a panel are derived from the same experiment. See source data for uncropped western blots.

### Human subjects and CTC isolation

Patient samples analyzed in this study were previously published by Hong et al.^12^. In brief, metastatic melanoma patients being evaluated or treated at the Massachusetts General Hospital Cancer Center provided written informed consent, and blood collections were performed as per IRB protocol (DF/HCC-0500), in accordance with the U.S. ethical guidelines. CTCs were isolated from 5 to 10 mL of whole peripheral blood samples drawn in EDTA vacutainers, and blood samples were processed within 4 h of being collected from the patient. Whole blood samples were first spiked with biotinylated antibodies against CD45 (R&D Systems, clone 2D1) and CD66 (AbD Serotec, clone 80H3), followed by incubation with Dynabeads MyOne Streptavidin T1 (Invitrogen) to magnetically label white blood cells. CTCs were then isolated from the blood sample using CTC-iChip microfluidic capture^28,29^. Patient response to immunotherapy was determined using radiological data and RECIST criteria.

### CTC culture

Melanoma CTCs were cultured in ultra-low attachment plates (Corning) at 4% oxygen in RPMI-1640 media with GlutaMAX (Gibco) supplemented with 1x b27, EGF (20 ng/mL), FGF (20 ng/mL), and 1x antibiotic/antimycotic (Life Technologies)^30^. CTC lines were routinely checked for mycoplasma (MycoAlert, Lonza). See Supplementary Table 2.

### Isogenic melanoma CTC culture RNA sequencing analysis

Establishment and validation of isogenic melanoma CTC lines and RNA sequencing was previously published by Hong et al.^12^ Expression values (Log_2_(TPM + 1)) for KEAP1, MITF, and BAP1 for each isogenic cell line were normalized to the average expression for all isogenic cell lines for each gene.

### Primary CTC single cell RNA sequencing analysis

Primary CTC isolation and generation of the CTC single cell RNA sequencing (scRNAseq) dataset was previously published by Hong et al.^12^. using the SMART-seq2 protocol^31^. Briefly, after microfluidic enrichment, CTCs were identified by size (>10 μm) and lack of CD45 staining, and they were collected by micromanipulation. The identity of melanoma CTCs was then validated by RNA sequencing by their expression of melanoma CTC markers and separation from white blood cells in hierarchical clustering analysis^12^. Putative CTCs that clustered with white blood cells, or did not express known melanoma markers, were discarded. Additionally, ‘RNA-SeQC’ analysis was used for further quality control of melanoma CTCs. Specifically, any CTCs which had percent MT > 25%, rRNA rate >10%, exonic rate <10%, exon CV MAD > 2.5, or median exon CV > 2 were excluded from further analysis. 46 CTCs from 15 patients receiving immune checkpoint inhibitors targeting PD1 or both PD1 and CTLA4 were selected for differential sequencing analysis. One patient had previously progressed on anti-PD1 therapy (pembrolizumab) prior to CTC isolation and was receiving anti-CTLA4 therapy (ipilimumab) at the time of CTC isolation. The best overall response after three months was used to separate CTCs by “Complete Response”, “Partial Response”, and “Progressive Disease”. Differential gene expression analysis was performed using ‘DESeq2’ comparing CTCs from patients with “Complete Response” compared to “Progressive Disease” (see Supplementary Table 1 for full differential sequencing results). Statistical comparisons of individual genes were made using Wilcoxon rank sum test for significance on log2 transformation of normalized counts (transcripts per million+1).

### Gene set enrichment analysis

Gene set enrichment analysis (GSEA) was performed using GSEA_4.1.0 (Broad Institute) and a rank metric(fold change)-sorted list of genes from differential expression analysis. The “NRF2 gene signature” was derived by Romero et al.^32^ (see Supplementary Table 3), and the “Hallmarks_Reactive_Oxygen_Species” gene signature is from the Molecular Signatures Database (MSigDB) hallmarks gene sets^33^. For correlating *KEAP1* expression to gene signatures, Pearson correlations were generated to rank genes based on their correlation with KEAP1 expression in the primary melanoma CTC dataset, melanoma lines from the cancer cell line encyclopedia, and tumors from the Cancer Genome Atlas (Skin cutaneous melanoma, TCGA, Firehose Legacy, previously known as TCGA Provisional). This ranked list of Pearson correlations was used for GSEA using GSEA_4.1.0 (Broad Institute).

### Cibersort analysis

Cibersort analysis^16^ was performed using transcriptomic data from the Cancer Genome Atlas (Skin cutaneous melanoma, TCGA, Firehose Legacy, previously known as TCGA Provisional). Using the online interface (https://cibersortx.stanford.edu/), cell fractions were imputed using the LM22 module. The top and bottom thirds (*n* = 155) of tumors ranked by KEAP1 expression were used to compare immune populations in KEAP1 “high” and “low” tumors.

### Interferon gamma stimulated PD-L1 and HLA expression assays

For melanoma CTC experiments, 500,000 Mel167 CTC cells were treated with 30 pmol of *KEAP1*-targeting siRNA smart pools or non-targeting controls (Horizon Discovery) overnight. Media was refreshed the next day, and 48 h after siRNA treatment, cells were collected for qRT-PCR validation of KEAP1 knockdown. Cells were also treated overnight with vehicle control or 100 ng/mL interferon gamma (Cell Signaling Technology #80385). Cells were collected, rinsed twice with PBS containing 2% fetal bovine serum, and incubated on ice for twenty minutes for cell-surface staining with a PE-conjugated anti-PD-L1 antibody (Biolegend #329705, 1:20) or a PE-congugated anti-HLA-A/B/C antibody (Biolegend #311405, 1:20). After two more washes, cells were analyzed by flow cytometry. KEAP1 knockout and control B16-ova cells, described above, were treated overnight with vehicle control or 100 ng/mL interferon gamma (Cell Signaling Technology #39127). Cells were stained with a PE-conjugated anti-PD-L1 antibody (Biolegend #155403, 1:20), as described above, and analyzed using flow cytometry. The expression of the MHC-I HLA gene, H2-d1 was analyzed by qRT-PCR. Antibody dilutions are included in Supplementary Table 2.

### Paired single cell DNA methylation and RNA sequencing

Cultured live MEL167 CTCs were used for paired single cell DNA methylation and RNA sequencing analysis to obtain the transcriptomes and DNA methylomes from the same single cells^34,35^. Single cells were first lysed in 5 μl DNA/RNA lysis buffer. 0.5 μl Magnetic MyOne Carboxylic Acid Beads (Invitrogen, Cat#65011) were then added to each single cell lysate to facilitate separation of the nucleus from the cytoplasm. After centrifugation and magnetic separation, the cytoplasmic RNA containing supernatant was transferred into a new tube for scRANseq amplification using the SMART-seq2 protocol^31^, while the pellet containing aggregated beads with the intact nucleus was resuspended in DNA methylation lysis buffer and subjected to single cell whole genome methylation sequencing using the scBS-seq protocol^36^.

### Paired single cell DNA methylation and RNA sequencing analysis

For single cell RNA sequencing analysis, raw fastq reads generated from HiSeq X sequencer were first cleaned using trimgalore (v0.4.3) to remove the adapter-polluted reads and reads with low sequencing quality. Cleaned reads were aligned to the human (hg19) genome using Tophat (v2.1.1)^37^. PCR duplicates were further removed using samtools (v1.3.1)^38^, gene counts were computed using HTseq (v0.6.1)^39^, gene expression level (FPKM) was further calculated using cufflinks (v2.1.1)^37^. Gene expression matrix was subjected to R (v3.1.2) or Prism9 for graphics. For single cell DNA methylation sequencing analysis, raw fastq reads from the were first trimmed using trimgalore (v0.4.3) (https://github.com/FelixKrueger/TrimGalore), and cleaned reads were aligned to the human hg19 genome (in silico bisulfite converted) using Bismark tool (v0.17.0)^40^. Samtools (v1.3.1)^38^ was used to remove PCR duplicates, and CpG methylation calls were extracted using the Bismark methylation extractor^40^. 0.1% lambda DNA was spiked in, prior to bisulfite treatment, for each sample to assess the bisulfite conversion efficiency. Only samples with more than 4 million unique CpG sites covered at least once and with a bisulfite conversion rate >98% were used in this study.

### Tumor growth experiments

Animal care and animal experiments were performed with the approval of, and in accordance with, guidelines approved by the Massachusetts General Hospital (MGH) Institutional Animal Care and Use committee (IACUC No. 2010N000006). Generation of KEAP1 and KEAP1/NRF2 knockout B16-OVA cells is described in the “Cell lines” section. One million cells were injected subcutaneously into the flank of 6-week-old female C57BL/6 J mice. Tumor growth was monitored by anesthetizing mice and measuring tumor size by palpation with calipers every two days. Once tumor formation was detected by palpation (greater than 1 mm × 1 mm) in all mice, cohorts were subjected to either 4 treatments of 100 µg of anti-PD1 (Leinco #P377) or 4 control treatments. Tumor volume was calculated using the formula: Volume = L_1_^2^ × L_2_ × π/6, where L_1_ is the shorter of the two measurements, and L_2_ is the longer measurement. For Fig. 2c, d, the control cohort was untreated. For Fig. 2e, the control cohort was treated with 4 100 µg doses of Rat IgG2a control antibody. For survival analysis, events were determined by the day tumors surpasses 500 mm^3^. Conditions were compared using Kaplan–Meier survival curves and evaluated by the hazard ratio and p-value calculated from a log-rank (Mantel–Cox) test.

### Immunocytochemistry

Cultured melanoma CTCs were collected by cytospin and fixed with 2% paraformaldehyde. Cells were permeabilized, and heat-induced epitope retrieval was performed in 1x citrate buffer (pH 6) (Thermo scientific AP-9003). Cells were blocked for 30 min in Bloxall (Vector Laboratories). Cells were incubated in KEAP1 primary antibody (Sigma HPA005558) diluted 1:500 in antibody diluent (Dako S0809) overnight at 4 °C. Cells were rinsed and incubated in HRP labelled anti-rabbit secondary antibody (Dako K4003) for 30 min before incubation with DAB substrate (Vector laboratories SK-4100). Cells were counterstained with hematoxylin (Vector Laboratories H3404), dehydrated, and mounted with DPX mountant (Sigma). Antibody dilutions are included in Supplementary Table 2.

### Tissue microarray staining

A melanoma tissue microarray (US Biomax ME1002b) was stained for KEAP1 (Sigma Aldrich HPA005558) with AP IgG magenta using the ImmPRESS Duet Double Staining Polymer immunohistochemistry kit (Vector Laboratories MP-7724). The slide was counterstained with hematoxylin (Vector Laboratories H3404) and scanned at 40x resolution using an Aperio Scanscope (Leica Biosystems). Image quantification was performed using ImageJ. Tumors were categorized as high, medium, and low by setting intensity bins that span equal thirds of the total range of intensity measured. Antibody dilutions are included in Supplementary Table 2.

### Patient survival analysis

RNA sequencing and survival data for tumors from anti-CTLA4 naïve melanoma patients receiving anti-PD1 therapy are publicly available from Liu et al.^19^ To determine the association of KEAP1 RNA expression on patient survival, tumors were binned into high, medium, and low cohorts of equal size by RNA expression value. All surviving patients were censored at 1000 days, and Kaplan–Meier survival curves were generated to compare the high and low expression cohorts. Hazard ratios were determined from log-rank test. *p* values were determined by log-rank (Mantel–Cox) test.

### Statistical analysis

Plots and statistical analyses were generated using Prism 9 and RStudio software. Specific statistical tests are identified in figure legends for each experiment.

### Reporting summary

Further information on research design is available in the Nature Research Reporting Summary linked to this article.

## Supplementary information

Supplementary Info

REPORTING SUMMARY

## Data Availability

RNA sequencing data of primary melanoma CTCs from this study were published by Hong et al.^12^ and are deposited into the Gene Expression Omnibus repository under accession number GSE157745. Matched RNA and bisulfite sequencing data for cultured melanoma CTCs are deposited under accession number GSE218431. RNA sequencing and survival data for tumors from anti-CTLA4 naïve melanoma patients receiving anti-PD1 therapy are publicly available from Liu et al.^19^.

## Code availability

No previously unreported custom computer codes or algorithms were used to generate or process the data presented in this manuscript.
