# Supplementary material for: Downregulation of KEAP1 in melanoma promotes resistance to immune checkpoint blockade
Source: NPJ Precis Oncol. 2023 Mar 2;7:25. doi: 10.1038/s41698-023-00362-3 (PMC9981575; doi:10.1038/s41698-023-00362-3)
Supplement: Supplementary file 1 — Supplementary Info [file 41698_2023_362_MOESM1_ESM.pdf]

Supplementary Figure 1: Single CTC-derived gene signatures associated with resistance to ICB

A

Patient ID	Immunotherapy received	Sequence validated CTCs	Days between Tx start and blood draw	Response
Mel-188	Nivolumab	3	28	CR
Mel-194	Ipilimumab + Nivolumab	3	44	CR
Mel-233	Ipilimumab + Nivolumab	2	0	CR
PEM-62	Pembrolizumab	3	0	CR
Mel-196	Ipilimumab + Nivolumab	1	44	PR
Mel-201	Nivolumab	2	-12	PR
Mel-208	Ipilimumab + Nivolumab	4	20	PR
Mel-209	Ipilimumab + Nivolumab	1	0	PR
Mel-232	Ipilimumab + Nivolumab	7	0	PR
PEM-61	Pembrolizumab	4	0	PR
PEM-95	Pembrolizumab	6	0	PR
Mel-192	Pembrolizumab then Ipilimumab	2	35	PD
Mel-195	Pembrolizumab	4	41	PD
PEM-93	Pembrolizumab	3	22	PD
PEM-97	Pembrolizumab	1	17	PD

CR: complete response
PR: partial response
PD: progressive disease

B

Top genes enriched in PD

symbol	log2FoldChange	p value
GDPD5	26.18	1.56E-19
TXNL4A	25.01	2.28E-18
GYPC	28.01	3.39E-18
PREPL	23.94	5.10E-18
FECH	27.07	3.15E-17
DARS	25.84	3.19E-17
ACSL6	26.69	6.71E-17
RGCC	25.77	1.01E-16
EID1	26.47	1.01E-16
ASNA1	25.62	1.04E-16
SKIV2L	24.55	1.31E-16
SUOX	24.51	1.58E-16
PPME1	26.14	2.05E-16
ATP6V1G1	25.19	2.05E-16
CYB5A	25.34	2.26E-16
NDUFS8	24.58	3.17E-16
SRXN1	25.80	4.62E-16
SHISA5	25.73	5.40E-16
WDR46	25.67	6.23E-16
RRAGA	25.52	9.36E-16
NELFE	24.66	1.07E-15
ZNF174	23.42	1.23E-15
UROS	23.66	1.43E-15
DPM2	24.94	4.41E-15
MBNL3	24.08	4.41E-15
NAPB	24.57	1.15E-14
BLOC1S2	24.46	1.51E-14
NDUFS5	24.33	2.06E-14
NDUFB9	24.16	3.23E-14
DNAJC7	24.08	3.74E-14
CD36	24.04	4.14E-14
SLC22A4	24.03	4.22E-14
KRR1	23.88	6.25E-14
PSMC3	23.80	7.25E-14
RNF13	23.64	8.95E-14
UBFD1	23.39	1.18E-13
ANXA3	23.46	1.18E-13
ETAA1	23.54	1.27E-13
NHP2	23.46	1.62E-13
FAF2	23.44	1.68E-13
THEGL	23.34	1.84E-13
C12orf29	23.20	3.05E-13
PSMB6	23.16	3.26E-13
COX6B1	23.16	3.31E-13
MRPL37	23.14	3.38E-13
TIMM17B	22.55	5.57E-13
CDK1	22.78	6.32E-13
JADE1	22.82	6.63E-13
KLHL15	22.77	8.33E-13
ATP6V0E1	22.68	1.01E-12

Top genes Enriched in CR

symbol	log2FoldChange	p value
IER5	-23.74	3.39E-18
RGL2	-23.03	3.15E-17
C15orf52	-24.71	4.00E-17
KEAP1	-22.80	1.01E-16
UBL4A	-23.49	2.05E-16
ENO2	-24.97	4.41E-15
TFPI	-24.49	4.41E-15
CHTF8	-23.72	2.04E-14
TAF7	-24.20	3.23E-14
TEX15	-23.63	3.56E-14
NT5DC2	-22.82	6.34E-14
DIO2	-22.97	8.88E-14
ITGB8	-23.75	8.95E-14
TRIM4	-23.73	9.25E-14
CCDC30	-22.88	1.86E-13
CRY1	-23.06	4.60E-13
CYP46A1	-23.02	5.09E-13
FAM210A	-22.92	6.32E-13
FAM53C	-22.78	8.70E-13
ZMAT2	-22.72	1.01E-12
FGD4	-21.65	1.72E-12
LZTFL1	-22.41	2.01E-12
DUSP7	-22.37	2.21E-12
FBXO3	-22.24	3.01E-12
TSPAN15	-22.11	4.11E-12
ULK3	-21.94	6.14E-12
ZNF394	-21.79	9.09E-12
FAM104A	-21.74	1.00E-11
RPS19BP1	-21.62	1.30E-11
RAB33B	-21.61	1.31E-11
BEND6	-21.61	1.31E-11
PIGA	-21.56	1.45E-11
TUFM	-21.47	1.80E-11
DOLPP1	-21.25	2.99E-11
TXNDC17	-21.22	3.20E-11
L3HYPDH	-21.21	3.29E-11
TPRA1	-21.16	3.67E-11
TBCC	-21.12	4.01E-11
REEP3	-20.84	7.79E-11
FAM189B	-20.48	1.86E-10
RNF8	-9.07	4.61E-03
C1orf43	-9.32	6.20E-03
NDFIP1	-9.94	6.96E-03
HINFP	-8.15	9.81E-03
MYBL2	-8.33	1.38E-02
RNF166	-8.43	1.74E-02
GPATCH8	-8.47	1.90E-02
CCPG1	-8.22	1.92E-02
UBE2E3	-8.98	1.95E-02
TMED8	-8.23	2.04E-02

C

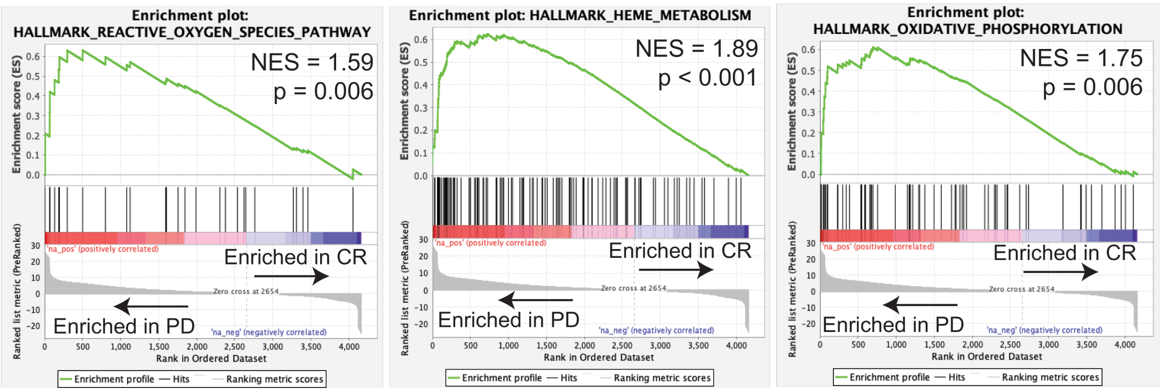

Supplementary Figure 1: Single CTC-derived gene signatures associated with resistance to ICB.

A) Table showing patient ID, immunotherapy treatments received, number of validated CTCs analyzed, days between immune checkpoint treatment initiation (Tx start) and blood draw (a positive value indicates the number of days after treatment initiation blood was drawn, and a negative value indicates number of days before treatment initiation blood was drawn), and clinical responses.

B) Table showing the fold change and adjusted p value for the top 50 genes enriched in CTCs from patients with PD and CR.

C) Gene set enrichment plots showing enrichment of the "Hallmark Reactive Oxygen Species Pathway", "Hallmark Heme Metabolism", and "Hallmark Oxidative phosphorylation" signatures in CTCs from patients with PD.

Supplementary Figure 2: Lack of correlation between *KEAP1* gene promoter methylation and expression at single cell level

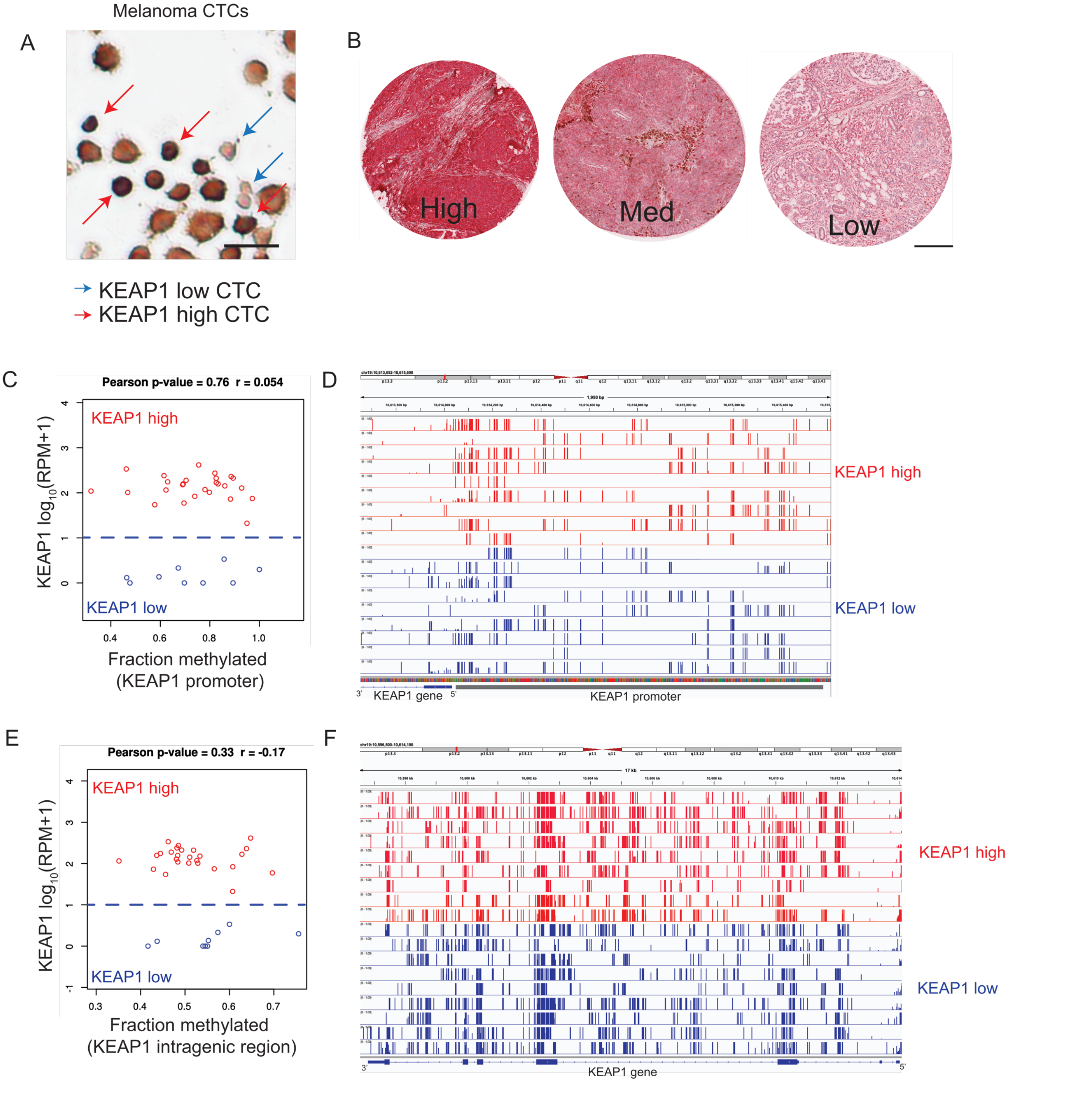

Supplementary Figure 2: Lack of correlation between *KEAP1* gene promoter methylation and expression at single cell level.

A) Immunocytochemistry for KEAP1 protein (brown) in MEL167 CTCs showing heterogeneity in protein expression. Scale bar represents 50μm.

B) Representative immunohistochemistry images used for quantification of KEAP1 (red) staining of melanoma tumor microarrays (quantified in Figure 1G). Scale bar represents 200µm.

C) Dot plot showing *KEAP1* expression versus fraction of methylated CpGs in the *KEAP1* promoter for single melanoma CTCs (n=35). Individual *KEAP1* high CTCs are labelled as red dots and *KEAP1* low CTCs are labelled as blue dots. Correlation determined by Pearson coefficient and p-value.

D) Representative IGV tracks showing CpG methylation patterns in the *KEAP1* promoter for single melanoma CTCs (n=35). Each track represents a single CTC. *KEAP1* high CTCs are labelled as red tracks and *KEAP1* low CTCs are labelled as blue tracks. The *KEAP1* promoter (chr19:10614056-10615555) is marked by a grey bar. *KEAP1* exons are marked as blue bars, and KEAP1 introns are marked as blue lines connecting exons.

E) Dot plot showing *KEAP1* expression versus fraction of methylated intragenic CpGs in the *KEAP1* gene body for single melanoma CTCs (n=35). Correlation determined by Pearson coefficient and p-value.

F) Representative IGV tracks showing intragenic CpG methylation patterns in the *KEAP1* gene body for single melanoma CTCs (n=35). Each track represents a single CTC. *KEAP1* high CTCs are labelled as red tracks and *KEAP1* low CTCs are labelled as blue tracks. *KEAP1* exons are marked as blue bars, and KEAP1 introns are marked as blue lines connecting exons.

Supplementary Figure 3: *KEAP1* expression is associated with immune signatures

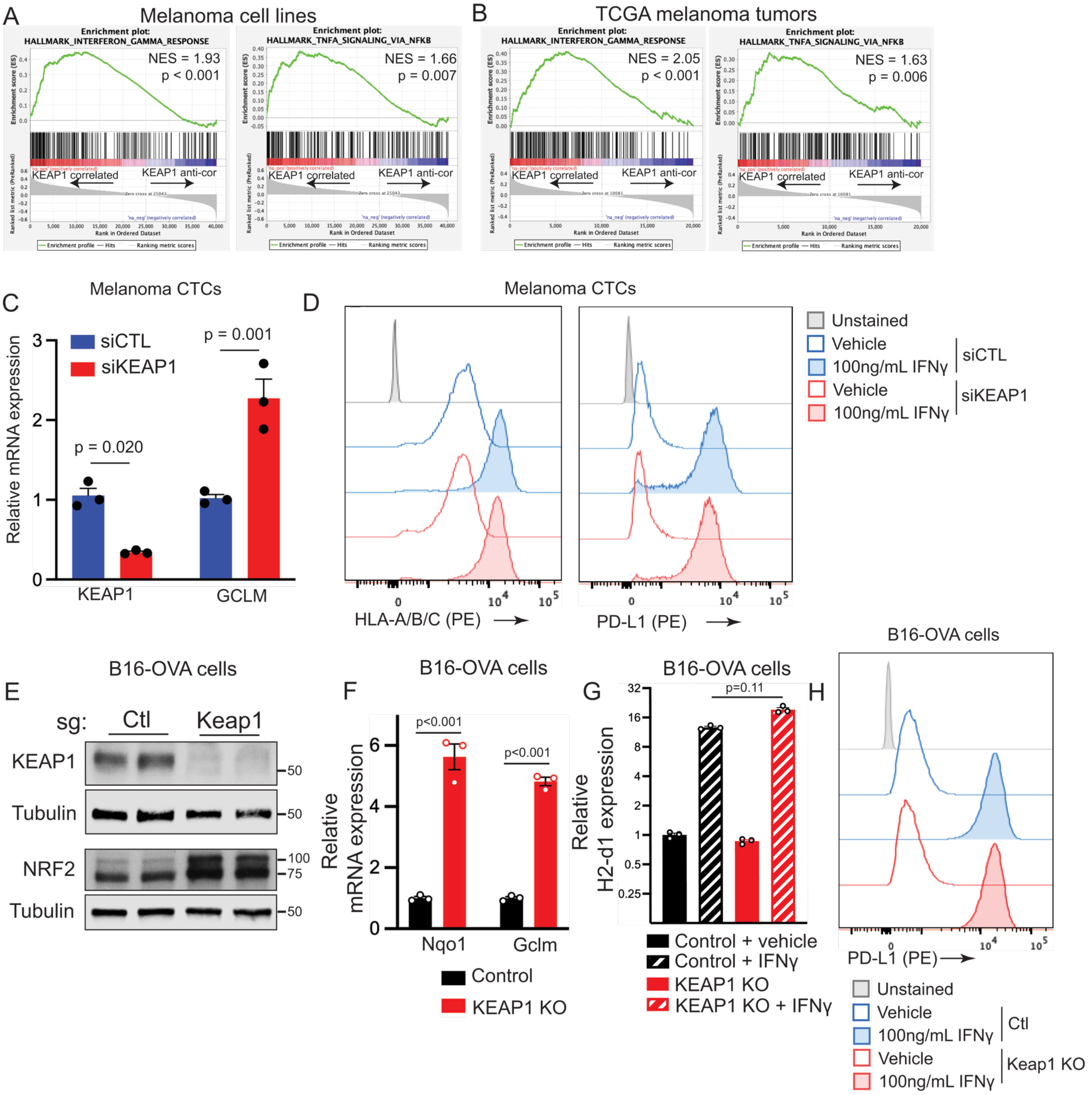

Supplementary Figure 3: *KEAP1* expression is associated with immune signatures.

A) Gene set enrichment analysis (GSEA) plots showing enrichment for "Hallmark Interferon Gamma Response" and "Hallmark TNFA Signaling via NFKB" signatures in genes correlated with *KEAP1* expression in melanoma cell lines.

B) GSEA plots showing enrichment for "Hallmark Interferon Gamma Response" and "Hallmark TNFA Signaling via NFKB" signatures in genes correlated with *KEAP1* expression in tumor samples from The Cancer Genome Atlas.

C) qRT-PCR confirming knockdown of *KEAP1* expression and upregulation of the NRF2 target gene *GCLM* in melanoma CTCs treated with *KEAP1*-targeting siRNA (siKEAP1), versus non-targeting controls (siCTL). Significance determined by two-way ANOVA (n=3 per condition).

D) Histograms showing flow cytometric analyses of cell surface expression of HLA-A/B/C (left) and PD-L1 (right) in melanoma CTCs treated with siRNA targeting KEAP1 versus control, either at baseline or following treatment with 100ng/mL IFNγ or vehicle control.

E) Western blots confirming absent expression of targeted protein in *KEAP1* knockout and resulting NRF2 protein stabilization in B16-OVA cells.

F) qRT-PCR showing expression of NRF2 target genes, *Nqo1* and *Gclm*, in B16-OVA cells following *KEAP1* knockout (red), compared with control cells (black). Significance determined by two-way ANOVA (n=3 per condition).

G) qRT-PCR for murine HLA ortholog *H2-d1* in *KEAP1* knockout (KO; red) B16-OVA cells versus parental cells (black), following treatment with 100ng/mL IFNγ or vehicle control. Significance determined by two-way ANOVA (n=3 per condition).

H) Histograms showing flow cytometric analyses of PD-L1cell surface expression in *KEAP1* knockout B16-OVA cells (red) versus parental controls (blue), following treatment with 100ng/mL IFNγ or vehicle control.

Data are represented as mean ± SEM.

Supplementary Figure 4: Effect of KEAP1 and NRF2 knockout on baseline B16-OVA tumorigenesis and response to anti-PD1 treatment

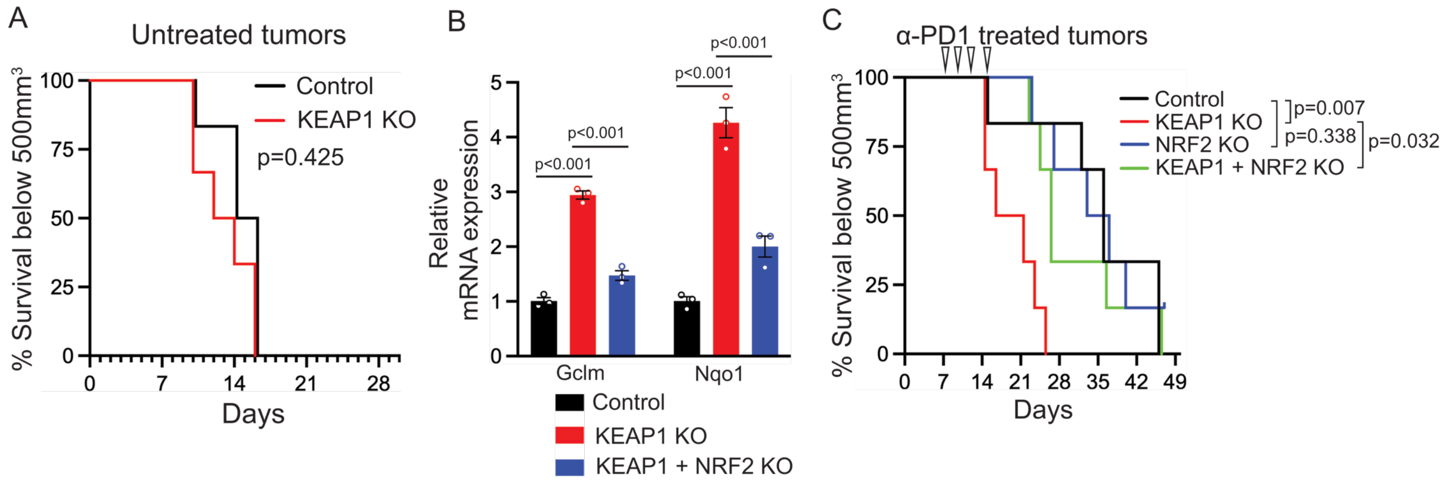

Supplementary Figure 4: Effect of *KEAP1* and *NRF2* knockout on baseline B16-OVA tumorigenesis and response to anti-PD1 treatment.

A) Kaplan-Meier survival plots for untreated mice from Figure 2C. Mouse survival is derived from sacrifice once tumors reached $500mm^3$ in size. Significance determined by log-rank (Mantel-Cox) test (n=6 per condition).

B) qRT-PCR showing expression of NRF2 target genes, *Nqo1* and *Gclm*, in B16-OVA cells with either *KEAP1* knockout, dual *KEAP1/NRF2* knockout, or control knockout. Significance determined by two-way ANOVA (n=3 per condition).

C) KM survival plots for anti-PD1 treated mice from Figure 2E. Mouse survival is derived from sacrifice once tumors reached $500mm^3$ in size. Significance determined by log-rank (Mantel-Cox) test (n=6 per condition).

Data are represented as mean ± SEM.

Uncropped westerms

Supplementary Figure 3E - Top

KEAP1

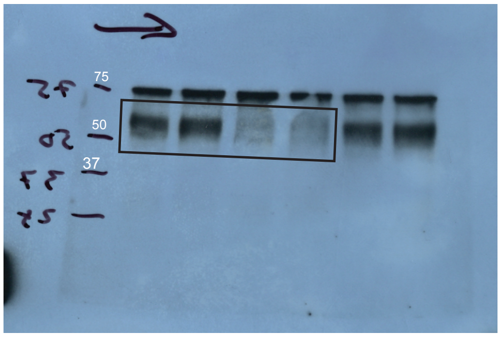

Tubulin (loading control)

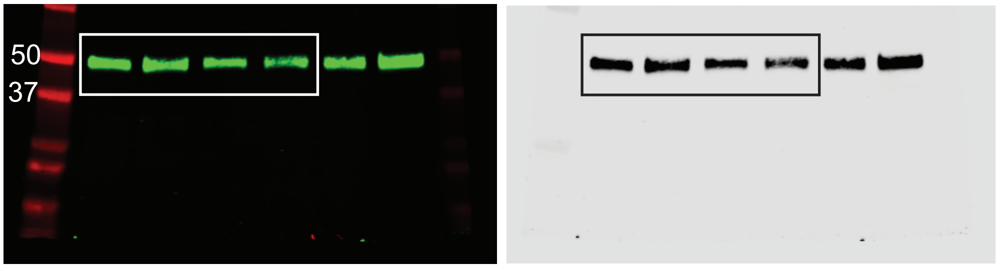

Supplementary Figure 3E - Bottom

NRF2

Exposure used in figure

Different exposure with molecular weight markers

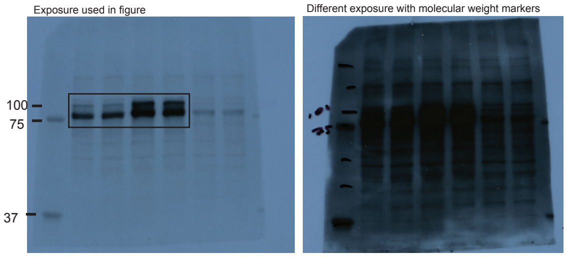

Tubulin (loading control)

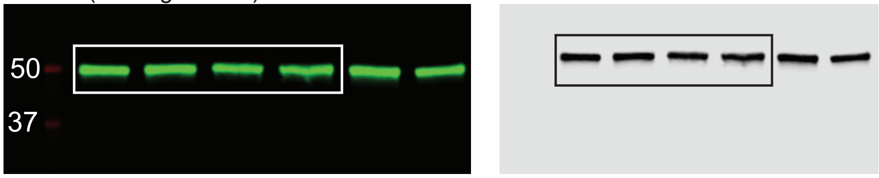

FACS gating strategy

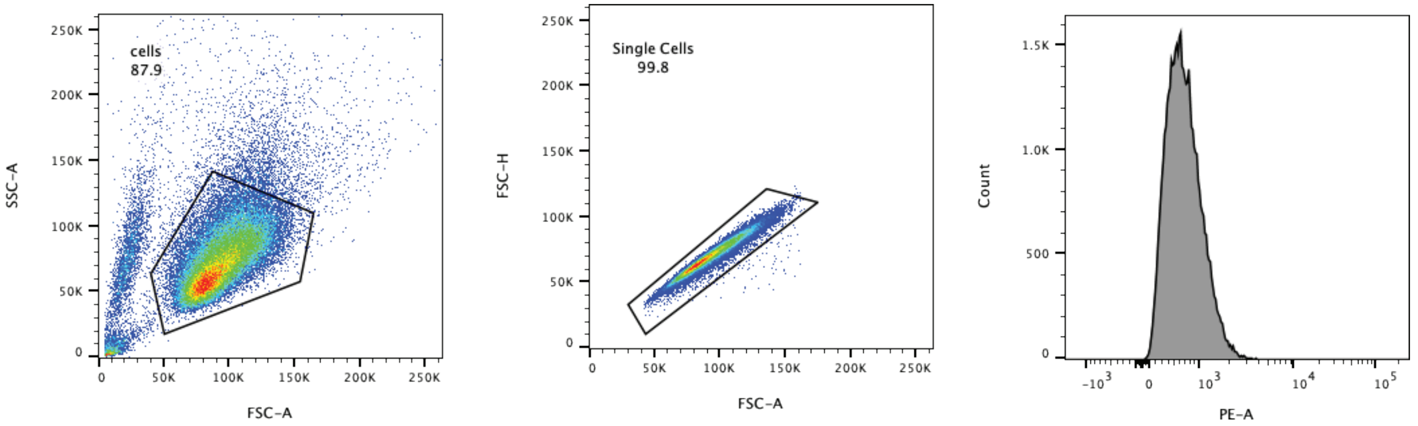

Gate 1 (Cells): SSC-A vs FSC-A → Gate 2 (single cells): FSC-H vs FSC-A → Histogram for PE-labelled antibodies

Supplementary Table 1 - DESeq results

symbol	baseMean	log2FoldChang	lfcSE	stat	pvalue	padj	rank	-log10(p)
GDPD5	105.047302	26.1779719	2.64140648	9.91061849	3.7432E-23	1.5587E-19	22.426755	18.80724427
TXNL4A	45.331209	25.0054416	2.61360182	9.56742584	1.096E-21	2.2819E-18	20.9601876	17.64170683
GYPC	392.255243	28.0062129	2.95348767	9.48242079	2.4845E-21	3.3936E-18	20.604759	17.46934344
PREPL	21.1843181	23.9383602	2.54993773	9.38782148	6.1254E-21	5.1012E-18	20.2128652	17.29232453
FECH	251.849273	27.0689937	2.95353026	9.16496237	4.9564E-20	3.1512E-17	19.3048313	16.50153014
DARS	82.5744277	25.8369535	2.82613836	9.14214035	6.1228E-20	3.1869E-17	19.2130478	16.49662704
ACSL6	151.463729	26.6913644	2.95360909	9.03686424	1.6123E-19	6.7136E-17	18.792553	16.17304229
RGCC	78.1174592	25.7653589	2.86957392	8.97880998	2.7371E-19	1.0142E-16	18.5627077	15.99387054
EID1	129.56715	26.4728043	2.95364262	8.96276485	3.1664E-19	1.0142E-16	18.4994379	15.99387054
ASNA1	70.4830564	25.6212532	2.86215304	8.95174117	3.4992E-19	1.0408E-16	18.4560329	15.98265021
SKIV2L	32.7454835	24.5499538	2.75265543	8.91864396	4.7203E-19	1.3104E-16	18.3260283	15.88260887
SUOX	31.6910786	24.5056213	2.75641087	8.89040946	6.0884E-19	1.5845E-16	18.2154959	15.80010518
PPME1	102.545948	26.1446705	2.95370377	8.85148698	8.6361E-19	2.0514E-16	18.0636831	15.68795955
ATP6V1G1	58.9725761	25.1877548	2.84713613	8.84669844	9.0147E-19	2.0514E-16	18.0450508	15.68795955
CYB5A	98.9709134	25.3440213	2.87160674	8.82572847	1.0875E-18	2.2642E-16	17.9635724	15.64509163
NDUFS8	53.1294884	24.5843518	2.79925934	8.7824488	1.5995E-18	3.1716E-16	17.7960065	15.49871512
SRXN1	80.2147889	25.8007848	2.95378528	8.7348207	2.4404E-18	4.6191E-16	17.6125338	15.33544573
SHISA5	76.313439	25.7337778	2.95380386	8.71208078	2.9835E-18	5.4014E-16	17.5252784	15.2674955
WDR46	73.0639647	25.6720564	2.95382118	8.69113424	3.5884E-18	6.2259E-16	17.4451007	15.2058012
RRAGA	66.1675902	25.5215493	2.95386468	8.64005367	5.6187E-18	9.3584E-16	17.2503671	15.02879638
NELFE	35.5941949	24.6583295	2.86065609	8.61981613	6.7062E-18	1.074E-15	17.1735254	14.96898801
ZNF174	14.6011541	23.4170622	2.72300531	8.59971227	7.9916E-18	1.2325E-15	17.0973652	14.90921822
UROS	17.3287636	23.65723	2.75767886	8.57867475	9.5972E-18	1.4272E-15	17.0178535	14.84550082
DPM2	43.2295822	24.9390631	2.95410362	8.44217617	3.1148E-17	4.4147E-15	16.506565	14.35509721
MBNL3	32.0481241	24.0784152	2.85471536	8.43461156	3.323E-17	4.4147E-15	16.4784636	14.35509721
NAPB	33.1025874	24.5662218	2.95431484	8.31537029	9.1466E-17	1.1541E-14	16.0387392	13.93774241
BLOC1S2	30.7062383	24.4612247	2.95438539	8.27963231	1.2356E-16	1.5132E-14	15.9081355	13.8201037
NDUFS5	28.0740422	24.332394	2.95447663	8.23577137	1.784E-16	2.0635E-14	15.7485946	13.68538633
NDUFB9	24.6498091	24.1550105	2.95462399	8.1753247	2.9507E-16	3.2333E-14	15.5300747	13.4903476
DNAJC7	52.1133313	24.0799979	2.95398542	8.15169828	3.5885E-16	3.7356E-14	15.4450886	13.42763787
CD36	32.9559443	24.0374594	2.95431895	8.13637925	4.0727E-16	4.1363E-14	15.3901126	13.38338576
SLC22A4	22.476477	24.0253275	2.95474109	8.13111091	4.2537E-16	4.2173E-14	15.3712291	13.37496771
KRR1	20.2410078	23.8765413	2.95488986	8.08034898	6.4582E-16	6.2539E-14	15.1898906	13.20384832
PSMC3	27.0699434	23.8040058	2.95451605	8.05682062	7.8304E-16	7.2458E-14	15.1062137	13.13991546
RNF13	16.9871466	23.6357399	2.94596995	8.02307568	1.0313E-15	8.9465E-14	14.986616	13.04834649
UBFD1	14.217218	23.3853744	2.92972028	7.9821185	1.4384E-15	1.179E-13	14.8421118	12.92850152
ANXA3	14.9728005	23.4581391	2.93901092	7.98164406	1.444E-15	1.179E-13	14.8404421	12.92850152
ETAA1	45.3731453	23.5441686	2.95407149	7.97007408	1.5858E-15	1.2699E-13	14.7997536	12.89624618
NHP2	14.9669813	23.4582423	2.95540566	7.93740184	2.0646E-15	1.6221E-13	14.6851634	12.78992857
FAF2	14.7568251	23.4379871	2.9554339	7.93047245	2.1831E-15	1.6834E-13	14.6609189	12.77380196
THEGL	13.778868	23.3419304	2.94828893	7.91711087	2.4309E-15	1.8404E-13	14.6142276	12.73507956
C12orf29	12.4513207	23.2015572	2.95580683	7.84948357	4.1775E-15	3.0518E-13	14.3790795	12.51544362
PSMB6	20.777565	23.1631873	2.95484974	7.83904067	4.54E-15	3.2594E-13	14.3429427	12.48685999
COX6B1	12.0949962	23.1596143	2.95587696	7.83510767	4.6844E-15	3.3061E-13	14.329345	12.4806863
MRPL37	11.9982245	23.1447623	2.95589594	7.83003285	4.8774E-15	3.3849E-13	14.3118094	12.47044992
TIMM17B	7.80186706	22.5487996	2.90540337	7.76098762	8.4271E-15	5.5699E-13	14.0743242	12.25415405
CDK1	9.20642616	22.7766648	2.94231832	7.74106073	9.8591E-15	6.3159E-13	14.0061636	12.19956628
JADE1	9.49339695	22.8241233	2.9515359	7.73296485	1.0507E-14	6.629E-13	13.9785199	12.17855316
KLHL15	9.16476505	22.7719087	2.95665975	7.70190372	1.3405E-14	8.3313E-13	13.8727206	12.07928468
ATP6V0E1	8.58955198	22.6838305	2.95687596	7.67155295	1.6993E-14	1.0108E-12	13.7697394	11.99532672
ZNF443	13.7459003	22.644281	2.95557938	7.66153708	1.8372E-14	1.0775E-12	13.7358416	11.96758927
TIMP3	8.35536537	22.6430166	2.95697271	7.65749934	1.8959E-14	1.0965E-12	13.7221884	11.9600102
NCBP2.AS2	9.83647843	22.6111882	2.95643599	7.64812373	2.0393E-14	1.1633E-12	13.6905126	11.93432476
NINJ1	7.92208505	22.568204	2.95716538	7.63170165	2.3168E-14	1.3036E-12	13.6351206	11.88484164
UNC119	7.53171053	22.5014892	2.95735065	7.60866459	2.7694E-14	1.5376E-12	13.5576105	11.81316107
ZNF592	6.66993907	22.3352294	2.95785396	7.55116032	4.314E-14	2.2738E-12	13.3651225	11.64323883
PLD6	21.9280171	22.1944342	2.95477484	7.51137917	5.8508E-14	3.0077E-12	13.2327873	11.52176159
GAB3	5.9944485	22.1835498	2.95835283	7.4986153	6.4496E-14	3.2751E-12	13.1904706	11.48477369
COX5B	5.25836717	22.0041037	2.9590334	7.43624716	1.0359E-13	5.1349E-12	12.9846992	11.28946773

TRAF6	4.18202795	21.687714	2.96044993	7.32581687	2.3745E-13	1.1236E-11	12.6244317	10.9494037
ORMDL2	4.12112463	21.6625505	2.9605521	7.31706446	2.5345E-13	1.1858E-11	12.5961006	10.92597991
MRPL51	4.06470392	21.6465638	2.96065002	7.3114227	2.6433E-13	1.223E-11	12.5778559	10.91258769
SEC61G	3.99931799	21.625821	2.96076558	7.30413144	2.7906E-13	1.2769E-11	12.554297	10.89382766
ZNF644	3.88534169	21.5842734	2.96097757	7.2895768	3.1093E-13	1.3629E-11	12.5073372	10.86555005
MEA1	3.55269365	21.4612131	2.96167114	7.24631872	4.2825E-13	1.8196E-11	12.3683009	10.74001627
ANAPC4	6.76655274	21.3681896	2.95779302	7.22436949	5.0343E-13	2.1175E-11	12.2980591	10.6741836
CA3	3.12637041	19.4723915	2.96277432	6.57235058	4.9527E-11	1.9274E-09	10.3051573	8.715030322
CA1	4648.1631	15.3398701	2.95042464	5.19920757	2.0014E-07	7.7165E-06	6.69866625	5.112579289
PRDX2	520.398948	14.9619346	2.95346886	5.06588533	4.0651E-07	1.5529E-05	6.39093241	4.808848189
DUS1L	65.0113499	10.3513931	2.14290391	4.83054469	1.3616E-06	5.1543E-05	5.8659503	4.287832266
SLC4A1	1217.94564	12.8811859	2.67441309	4.81645334	1.4613E-06	5.4819E-05	5.83525373	4.26106599
TFDP2	168.847579	8.72145519	1.84406921	4.72946196	2.2512E-06	8.3695E-05	5.64759436	4.077301659
RAP2C	85.6992375	11.8441861	2.51859452	4.7026967	2.5675E-06	9.461E-05	5.59049347	4.024061197
PPIP5K2	67.4096494	9.17500494	1.9596559	4.68194693	2.8416E-06	0.00010379	5.54643239	3.983826518
RPL7A	68.6404708	11.5255247	2.47310252	4.66035055	3.1567E-06	0.0001143	5.50076491	3.941952032
SHPRH	123.042533	8.57083586	1.86467639	4.59642001	4.2981E-06	0.00015429	5.3667216	3.81166887
EPHA4	66.0906887	10.5246989	2.30145844	4.57305624	4.8066E-06	0.00017107	5.31816106	3.766836203
ATP2B1	100.640044	10.0431783	2.19705975	4.57119034	4.8496E-06	0.00017113	5.31429272	3.766664004
RNH1	46.5036031	11.2687085	2.47479292	4.5533945	5.2787E-06	0.00018471	5.27747179	3.733508035
EPB42	470.714067	13.2628916	2.92421143	4.53554469	5.7455E-06	0.00019937	5.2406721	3.700342626
VPS39	121.610785	12.3448448	2.72586726	4.52877693	5.9326E-06	0.00020416	5.22675427	3.690028923
PSMB4	77.8208167	12.0236393	2.67324432	4.49777044	6.867E-06	0.00023438	5.16323432	3.630083431
CTSB	297.64909	9.5826567	2.1737823	4.4082872	1.0419E-05	0.00035273	4.98216846	3.452562846
CAT	181.263678	12.8851626	2.9535766	4.36256252	1.2855E-05	0.00043167	4.89093524	3.364846207
OCIAD1	82.2602841	10.9095843	2.50795281	4.34999584	1.3614E-05	0.00045351	4.86601389	3.343413181
HADHA	147.065157	12.60477	2.92097395	4.31526272	1.5941E-05	0.00052682	4.79747572	3.278335542
OAT	137.913869	12.5288367	2.95362855	4.24184576	2.2169E-05	0.00072686	4.65425581	3.138548805
CYB561	30.3231165	10.2624671	2.43641309	4.21212113	2.5298E-05	0.00082247	4.59690728	3.084881185
CDC27	126.264787	11.3767083	2.70198109	4.21050627	2.548E-05	0.00082247	4.5938022	3.084881185
C18orf25	118.383377	9.66919127	2.31997531	4.16779921	3.0755E-05	0.00098512	4.51207768	3.006510308
FAM122A	74.4968819	11.0149664	2.64714634	4.16107195	3.1676E-05	0.00100685	4.49927342	2.997033992
NAA10	36.3514769	10.7263268	2.58103738	4.15582002	3.2412E-05	0.00102246	4.48929027	2.990353484
HBD	1042.49838	12.1042301	2.92246677	4.14178538	3.4461E-05	0.00107892	4.46266868	2.967009596
LBR	111.640771	8.61461466	2.08709917	4.12755406	3.6664E-05	0.00113933	4.43575748	2.943351559
CD44	132.494602	10.6079463	2.5765313	4.11714242	3.836E-05	0.00118319	4.41612249	2.926945537
ALAS2	403.933848	12.0516961	2.9520863	4.08243353	4.4567E-05	0.00136452	4.35099087	2.865019057
CARS	99.6008248	10.8771527	2.69168395	4.04102149	5.3219E-05	0.00161754	4.27393425	2.791144101
MORF4L2	50.1854096	9.34428529	2.32092575	4.02610263	5.6709E-05	0.00171113	4.24634844	2.766716803
MBOAT2	171.887886	11.141289	2.80987093	3.96505364	7.3379E-05	0.00219018	4.13442558	2.659521163
GHITM	278.794783	11.7062468	2.95297775	3.96421776	7.3637E-05	0.00219018	4.13290385	2.659521163
ZC3H13	47.426234	9.37084318	2.36684256	3.95921694	7.5196E-05	0.00222068	4.12380579	2.65351418
MGST3	63.7518288	10.54801	2.68759274	3.92470549	8.6836E-05	0.00254637	4.06130066	2.594078281
AHSP	1412.49217	11.258705	2.9026054	3.87882728	0.00010496	0.00305635	3.97897103	2.514796348
ZNF571	24.8316678	10.0290523	2.60583298	3.84869346	0.00011875	0.00343384	3.92536822	2.464219993
NDUFS7	39.1222552	10.8353527	2.82803246	3.83141029	0.00012741	0.00365889	3.89479365	2.436650927
OSBP2	215.481681	11.2220008	2.95260488	3.800712	0.00014428	0.0040773	3.8407911	2.389627561
LMBR1	48.8996854	9.41457664	2.47776821	3.79961959	0.00014492	0.0040773	3.83887657	2.389627561
ATP6AP2	24.9836261	9.9695837	2.62803908	3.79354469	0.00014851	0.00415036	3.82823878	2.381914323
FDFT1	38.1475866	11.1920903	2.95419597	3.78854024	0.00015154	0.00420661	3.81948691	2.376067446
SDCBP	160.247712	11.1727317	2.95359832	3.78275259	0.0001551	0.00425851	3.80937822	2.370742637
ST6GALNAC4	60.3297841	11.1722633	2.9539078	3.7821977	0.00015545	0.00425851	3.80840977	2.370742637
NID1	19.692901	7.75502827	2.05308543	3.77725551	0.00015857	0.00431548	3.79978984	2.364970546
EIF3H	52.0676305	11.0815897	2.95398801	3.7513997	0.00017585	0.00472413	3.75485739	2.325678363
EIF4E	99.1979349	5.94448005	1.58713464	3.74541635	0.00018009	0.00480715	3.74449871	2.318112583
TBRG4	21.6610487	9.02779697	2.42266761	3.72638696	0.00019424	0.0051518	3.7116521	2.288041036
NAA20	28.7797698	10.3942575	2.80056829	3.71148155	0.00020605	0.00543032	3.68602804	2.265174406
GTF3C1	43.3206799	9.90600238	2.67195341	3.70740087	0.0002094	0.00548384	3.67902883	2.260915235
ZNF83	29.097734	8.82951842	2.38333922	3.70468389	0.00021165	0.00550831	3.67437246	2.258981727
PAGR1	42.2147533	9.87761969	2.6866742	3.67652307	0.00023643	0.00611499	3.62628919	2.213604341
PA2G4	28.2702762	9.61474289	2.61793926	3.67263788	0.00024006	0.00617042	3.61968101	2.2096853
GEMIN6	33.9249607	10.8398246	2.95429658	3.66917278	0.00024334	0.00620327	3.6137926	2.207379241

RPL28	43.3913706	7.17451019	1.9578449	3.66449364	0.00024783	0.00625429	3.60584894	2.20382216
ADARB2	11.3063611	4.72007211	1.29039426	3.6578527	0.00025434	0.00637988	3.59459022	2.19518759
SLC9A8	38.4215742	6.87289757	1.88395291	3.64812599	0.00026416	0.0065866	3.57813279	2.181338538
FAM217B	22.8771426	7.42738906	2.053826	3.61636724	0.00029877	0.00736132	3.52466819	2.133044177
HBB	43220.5691	8.04316256	2.23371495	3.60080079	0.00031724	0.00772504	3.49861399	2.112099383
RPL3	201.548755	8.15675573	2.26894901	3.59494888	0.00032446	0.00785484	3.48884516	2.104862886
C14orf119	52.1299774	10.2743865	2.8636025	3.58792342	0.00033332	0.00802285	3.47713583	2.095671209
BTRC	163.181725	7.53465773	2.10201992	3.58448446	0.00033774	0.00808258	3.4714115	2.092450027
DHX29	285.774191	7.13660282	1.99322924	3.58042251	0.00034304	0.00816237	3.46465641	2.088183739
SLC33A1	14.2279935	8.32574983	2.32795014	3.57642962	0.00034832	0.00824091	3.45802279	2.084024733
ATR	27.3970274	9.26418848	2.60062206	3.56229712	0.00036762	0.00864851	3.43459617	2.063058713
RPS6KA1	20.5182557	8.15321283	2.28992633	3.56046949	0.00037019	0.00866	3.43157259	2.062481869
SAR1B	33.2728872	6.94198016	1.96511693	3.53260412	0.00041149	0.00957227	3.38564266	2.018984968
PSMG4	26.7975966	9.15204486	2.59890754	3.52149691	0.00042912	0.00987208	3.3674236	2.005591455
APAF1	32.276669	7.44734382	2.11601294	3.51951715	0.00043233	0.0098914	3.36418153	2.004742193
TFR2	16.560006	7.46206294	2.13122209	3.50130706	0.00046298	0.01053474	3.33443582	1.977376191
THAP3	43.4255891	9.99289942	2.86094999	3.49286057	0.00047788	0.01080153	3.32068483	1.966514749
RPS4X	229.372284	9.38169519	2.69487361	3.48131176	0.00049896	0.01112183	3.30193047	1.95382375
NDUFA2	23.023861	10.285471	2.9547105	3.48104188	0.00049947	0.01112183	3.30149286	1.95382375
TXNRD1	13.6279367	7.99716274	2.30372781	3.47140088	0.00051775	0.01146762	3.28587953	1.940526657
LPIN2	83.9785784	8.02336933	2.31864119	3.46037557	0.00053942	0.01188442	3.26807096	1.925022044
TPGS2	54.9220455	10.172094	2.95341726	3.44417773	0.0005728	0.01255334	3.24199772	1.901240602
CTSC	16.4240832	9.42868956	2.74021429	3.44085848	0.00057987	0.01264181	3.23666806	1.898190703
ATP5H	39.4671837	9.13730917	2.65831883	3.43725105	0.00058765	0.01274467	3.23088077	1.89467128
FAM126B	15.9113688	9.53873049	2.77709842	3.43478301	0.00059303	0.01279467	3.22692446	1.892971045
C1orf52	55.4799218	8.48624416	2.47222015	3.43264096	0.00059773	0.01282969	3.22349271	1.891783717
TPM1	163.815872	5.77130835	1.68379635	3.42755723	0.00060904	0.0130053	3.21535567	1.885879562
RNF130	11.3587666	6.43951893	1.89422437	3.39955448	0.00067496	0.01426661	3.17072369	1.845679195
GPC5	33.144435	8.81743709	2.61280038	3.37470753	0.00073894	0.01554017	3.13138978	1.808544246
RPS6	62.4891558	7.86494003	2.34612766	3.35230694	0.00080141	0.01670802	3.09614463	1.777075054
SNX3	61.5034527	9.07809	2.70831602	3.35193158	0.0008025	0.01670802	3.09555578	1.777075054
RSAD2	37.3997317	8.29159214	2.47634352	3.34832064	0.00081303	0.01684305	3.08989403	1.77357937
HSBP1L1	36.1913019	8.44153396	2.52268993	3.34624318	0.00081915	0.01688575	3.0866391	1.772479746
RPL36A	12.8691347	7.36741245	2.20515839	3.34099015	0.0008348	0.01712372	3.07841659	1.766401908
PLEKHB2	124.109417	9.37094768	2.8091421	3.33587527	0.00085031	0.01735639	3.07042116	1.760540608
SYNE1	30.9901643	5.42422594	1.62939698	3.32897753	0.00087165	0.01761927	3.05965569	1.754012187
CCND1	38.9337327	8.34983848	2.50941071	3.32741008	0.00087657	0.01763309	3.05721204	1.753671663
PSMB1	15.3258442	7.61897828	2.29204852	3.32409118	0.00088707	0.0177585	3.05204118	1.750593798
FKTN	12.1748389	7.30502978	2.1996964	3.32092636	0.00089719	0.01787516	3.04711458	1.747750148
XRCC6	141.866214	7.42978925	2.24006763	3.31677006	0.00091065	0.0180568	3.04065075	1.743359323
PIM1	753.13894	7.87510742	2.37878638	3.3105568	0.00093111	0.01831537	3.03100109	1.737184389
ORMDL3	18.1502641	8.1366955	2.45861166	3.30946754	0.00093474	0.01831537	3.02931102	1.737184389
NARS	27.1263974	8.27930418	2.502188	3.30882579	0.00093688	0.01831537	3.02831551	1.737184389
RNF11	13.6366588	9.31496018	2.82159261	3.30131293	0.00096233	0.01872506	3.01667379	1.727576845
HBA2	6474.5629	6.74517862	2.04508203	3.29824356	0.00097292	0.01884291	3.01192419	1.724851926
NUCB1	16.4411296	8.00211658	2.43270697	3.28938778	0.00100406	0.01920277	2.99824212	1.716636225
BARD1	141.321592	7.04026079	2.14230961	3.28629473	0.00101515	0.0193017	2.99347093	1.714404328
AKNA	13.9114625	6.30453709	1.92342958	3.27775821	0.00104635	0.01962613	2.9803231	1.707165349
SURF4	18.0219102	8.5728925	2.61655398	3.27640575	0.00105137	0.01963193	2.97824278	1.707036922
NDUFV3	28.9737886	9.52241269	2.90899443	3.27343793	0.00106248	0.01975069	2.97368036	1.70441766
ATRX	194.418093	6.99571411	2.1439953	3.26293351	0.00110265	0.02037173	2.95756079	1.690972023
DDI2	13.8472712	7.75828177	2.37826586	3.26215917	0.00110567	0.02037173	2.9563743	1.690972023
HDAC3	12.9079489	9.62875665	2.95572483	3.25766342	0.00112334	0.02044727	2.94949049	1.689364755
EDEM3	30.4422771	7.99295112	2.45401772	3.25708778	0.00112562	0.02044727	2.94860967	1.689364755
C7orf73	101.368113	8.0490364	2.47196248	3.2561321	0.00112941	0.02044727	2.94714764	1.689364755
BTN2A1	8.17068222	8.77577015	2.70183623	3.24807627	0.00116188	0.02094404	2.93483827	1.678939525
GDE1	100.557606	8.44194663	2.60014867	3.2467169	0.00116744	0.02095362	2.93276375	1.678741013
CLP1	16.462155	9.58824545	2.95522944	3.24450119	0.00117657	0.02102669	2.92938399	1.677229187
SESN3	18.2223334	8.98829662	2.77642728	3.23736072	0.00120641	0.02142318	2.91850575	1.669116089
ANKH	33.2415218	8.75032055	2.70343752	3.2367386	0.00120904	0.02142318	2.91755895	1.669116089
RPL31	19.7531609	8.52483274	2.63616856	3.23379653	0.00122156	0.02152436	2.91308359	1.667069675
SLTM	55.3561181	6.55082321	2.02625339	3.23297336	0.00122509	0.02152436	2.91183205	1.667069675

TRMT112	73.6786326	8.75098194	2.71574231	3.22231675	0.00127158	0.02224739	2.89565469	1.652720925
EXOC3L2	53.5300287	6.42767083	2.00151335	3.21140543	0.00132087	0.02291717	2.87913849	1.639839012
WWC1	21.4206702	7.37929016	2.29892532	3.20988685	0.00132787	0.02294299	2.87684368	1.639350005
NDUFA13	31.3726171	9.4593477	2.9543685	3.20181714	0.00136564	0.02337842	2.86466487	1.631184832
SMIM1	17.7559447	9.45896715	2.95509603	3.20090008	0.00136999	0.02337842	2.86328251	1.631184832
PRDX1	22.9761334	9.2003913	2.88922811	3.18437691	0.00145066	0.02455507	2.83843439	1.609858775
E2F4	36.1932861	9.40227384	2.95424173	3.18263524	0.00145941	0.02460323	2.83582166	1.609007897
BOD1L1	18.3108632	5.62448388	1.76941925	3.17871748	0.00147928	0.02483762	2.82994903	1.604889986
SAMD4B	9.53216716	6.70221654	2.11191826	3.17352081	0.00150602	0.02518503	2.82216896	1.598857584
SH3BP2	7.95150065	6.67923559	2.10713396	3.1698201	0.00152533	0.02540595	2.8166352	1.595064489
CCND2	16.5364812	9.35740919	2.95521857	3.16640172	0.00154337	0.02560404	2.81152857	1.591691575
MPC2	16.288481	9.3343907	2.95524756	3.15858165	0.00158539	0.02619666	2.79986418	1.581754
IQSEC1	13.2306421	6.79940536	2.15688126	3.15242452	0.00161921	0.02660829	2.79069769	1.574982986
FBXL20	45.0736524	6.81455884	2.1621667	3.15172685	0.00162308	0.02660829	2.78965999	1.574982986
RPLP0	54.7088355	8.07698151	2.56802324	3.14521355	0.00165966	0.02710121	2.77998183	1.567011294
DHPS	15.9339601	8.21310529	2.61808886	3.13706132	0.0017065	0.02775736	2.76789261	1.556621856
HSPA1A	14.4106598	9.26663211	2.95548985	3.13539636	0.00171622	0.02777282	2.76542689	1.556379968
UBE2K	106.377391	6.46209731	2.06152785	3.13461558	0.00172079	0.02777282	2.76427098	1.556379968
AP4E1	8.64851737	6.2875005	2.01003857	3.12804968	0.0017597	0.02818234	2.75456029	1.550022913
RPL27	14.3183735	6.97530421	2.23420522	3.1220517	0.00179595	0.02865269	2.7457048	1.542834591
RPL27A	30.7585478	5.34731009	1.71509649	3.11778966	0.00182213	0.02895932	2.73942113	1.538211696
MRPL42	16.3599246	7.45667975	2.39288565	3.11618724	0.00183206	0.02899051	2.73706053	1.537744214
X8.Mar	166.310977	7.9083309	2.5386025	3.11523009	0.00183801	0.02899051	2.73565101	1.537744214
ST13	24.7447701	6.63534332	2.13452442	3.1085816	0.00187988	0.0295389	2.7258705	1.529605652
DCTN3	25.8543534	9.17863909	2.95457373	3.10658658	0.00189261	0.02962715	2.72293915	1.52831007
RCL1	8.28001968	7.2770722	2.34345738	3.10527183	0.00190104	0.02963949	2.72100822	1.528129341
RPL6	30.8529696	9.17114668	2.95438581	3.10424815	0.00190763	0.02963949	2.71950527	1.528129341
MGAT1	10.8694648	7.49294382	2.41914265	3.09735509	0.00195256	0.03022473	2.70939599	1.519637545
MINPP1	18.9566874	8.19861536	2.6499779	3.09384292	0.00197582	0.03047155	2.70425248	1.516105522
RPL37	11.2191087	4.3391128	1.40490744	3.08853999	0.00201143	0.03090619	2.6964959	1.50995447
RCBTB1	13.0342986	9.1254244	2.95570281	3.08739578	0.00201919	0.03091136	2.69482376	1.509881944
HAGH	117.982572	7.9595121	2.58191375	3.08279551	0.00205066	0.0312782	2.6881063	1.504758227
SGTA	17.6750685	7.54196178	2.44748759	3.08151176	0.00205952	0.03129874	2.68623326	1.504473102
GSPT1	14.452128	5.86831944	1.90516166	3.08022125	0.00206847	0.03132038	2.68435103	1.504173006
CIZ1	11.4007191	6.91637194	2.24885985	3.07550154	0.00210149	0.03159061	2.67747299	1.500442041
PET100	30.1852667	5.65262816	1.84166609	3.06930132	0.0021456	0.0321377	2.6684511	1.492985176
MXI1	63.1333075	9.0378603	2.94958605	3.06411142	0.00218318	0.03258331	2.66091129	1.487004769
COG4	9.77043323	9.04845895	2.95646533	3.06056657	0.00220919	0.03285376	2.65576763	1.483414941
WEE1	12.1292944	9.02050312	2.95587502	3.05172007	0.00227534	0.03371717	2.64295334	1.472148942
GLE1	10.4679575	6.84317349	2.25633095	3.03287667	0.00242235	0.03576825	2.61576387	1.446502255
DDX39A	94.4057134	6.94281393	2.29503345	3.02514716	0.00248512	0.03643679	2.60465232	1.438459942
NRBF2	21.6623582	8.92296358	2.95479863	3.01982121	0.00252924	0.03695352	2.59701006	1.432344197
TRIM5	12.6123927	8.14342469	2.69788789	3.01844444	0.00254076	0.03699204	2.59503638	1.431891692
TIMM23	21.2341296	8.89319975	2.95482749	3.00971877	0.0026149	0.03770556	2.58254543	1.423594627
CASP8	21.7382532	7.18648718	2.38776834	3.00970872	0.00261498	0.03770556	2.58253107	1.423594627
DACH1	56.7591517	7.61273878	2.52958408	3.00948241	0.00261693	0.03770556	2.58220751	1.423594627
TKT	7.66748713	8.87763504	2.95729572	3.00194363	0.00268262	0.0385187	2.57144109	1.414328365
TBC1D10B	35.8006741	7.13541056	2.37877102	2.99962061	0.00270316	0.03868028	2.56812812	1.412510389
ZNF131	25.2938167	7.34801443	2.45138865	2.9974906	0.00272212	0.03881821	2.56509232	1.410964455
SMIM12	6.80474671	6.1799523	2.06245682	2.99640324	0.00273185	0.03882396	2.56354327	1.410900174
SLC25A32	10.6927283	8.83845253	2.95620505	2.98979684	0.00279163	0.03940457	2.55414206	1.404453355
CSNK1G3	17.2709854	7.8041533	2.61322361	2.98640853	0.00282275	0.03970926	2.54932717	1.401108163
HIGD1A	76.8381699	7.72285547	2.5959386	2.97497617	0.00293011	0.04066198	2.53311561	1.390811435
SEC14L3	8.79907913	7.08802475	2.38337334	2.97394647	0.00293996	0.04066198	2.53165805	1.390811435
PRR5	22.7922488	6.41676026	2.15797271	2.97351317	0.00294412	0.04066198	2.53104483	1.390811435
CCDC91	70.3214087	6.53141259	2.19670991	2.97327042	0.00294645	0.04066198	2.53070132	1.390811435
CHORDC1	26.8473209	8.15060683	2.74154508	2.97299756	0.00294907	0.04066198	2.53031521	1.390811435
WDR19	10.8464258	6.65590675	2.23986115	2.97157114	0.0029628	0.04071653	2.52829733	1.390229236
UBE2A	6.18064515	8.05674014	2.7150302	2.96745876	0.00300273	0.04099459	2.52248435	1.387273472
EMC4	80.4560197	8.68835322	2.93010656	2.96520043	0.00302486	0.04114305	2.51929503	1.385703554
HFM1	9.78689614	6.98640371	2.35815112	2.96266157	0.00304992	0.04123329	2.51571199	1.384751982
NDUFAB1	11.8871874	7.54089685	2.55023462	2.95694239	0.00310706	0.04186992	2.50765014	1.378097894

FBXO25	9.24247399	8.21701065	2.79047348	2.94466538	0.00323304	0.04342704	2.49038878	1.362239753
TMEM245	38.5446471	7.14376112	2.43421931	2.93472371	0.00333845	0.04455544	2.47645538	1.35109925
UBE2V1	74.9561888	8.55757853	2.92931297	2.92136027	0.00348507	0.04621597	2.45778904	1.335207965
RAB3IP	13.3173224	6.60328315	2.26398309	2.91666629	0.00353794	0.04666142	2.45124944	1.331042071
ZNF616	14.1846999	6.94483447	2.38131145	2.91639065	0.00354107	0.04666142	2.45086571	1.331042071
UBE2Q2	14.0921841	7.548971	2.59183623	2.91259568	0.00358438	0.0470832	2.44558551	1.327134047
GSTP1	20.5660402	8.58343736	2.95487474	2.90483967	0.00367441	0.04811401	2.43481207	1.317728465
YTHDF2	32.3251584	6.93524638	2.39077579	2.90083513	0.0037217	0.04858039	2.42925906	1.313539024
FAM117A	145.124212	7.25816308	2.50899897	2.89285215	0.00381761	0.04966912	2.41820849	1.303913504
TSPAN5	11.3243802	6.21037238	2.1474914	2.89191955	0.00382896	0.04966912	2.41691919	1.303913504
PRDM2	28.0961302	7.72302636	2.67733537	2.88459431	0.00391918	0.05038554	2.4068044	1.297694095
C9orf78	14.4815567	7.01996313	2.43392323	2.88421715	0.00392388	0.05038554	2.4062842	1.297694095
ASCC3	12.6424406	6.40021681	2.2192754	2.88392185	0.00392756	0.05038554	2.40587693	1.297694095
ESRRG	12.8278408	7.47556712	2.59251527	2.88351903	0.00393259	0.05038554	2.40532145	1.297694095
PLCL2	9.26449434	8.51927536	2.95663513	2.88140909	0.00395901	0.05056851	2.40241294	1.296119821
ZC3HAV1	42.4237414	6.46134062	2.24614693	2.8766331	0.00401943	0.05116118	2.39583592	1.291059437
NFIA	23.248765	8.49716077	2.95470698	2.87580489	0.00402999	0.05116118	2.39469631	1.291059437
IPO5	6.16375925	6.32462364	2.20004958	2.87476413	0.00404329	0.05117409	2.39326465	1.290949832
ASPM	7.67987502	8.49024149	2.95729076	2.87095257	0.00409237	0.05163826	2.38802518	1.287028402
ARAF	92.4381772	7.45743332	2.6029935	2.86494504	0.00417082	0.05246912	2.37977895	1.280096221
EIF2S2	75.3131327	6.73819261	2.35349681	2.86305577	0.00419577	0.05262402	2.37718863	1.278815993
PTPN4	13.5487731	6.773861	2.36727091	2.86146422	0.00421689	0.05273013	2.37500763	1.277941139
ZNF347	33.8527292	6.68178953	2.34123136	2.85396379	0.00431775	0.05382961	2.36474299	1.268978739
RB1	215.577326	6.1345026	2.15022754	2.85295508	0.00433147	0.05383958	2.36336425	1.26889834
NDUFS2	16.4534921	7.14863338	2.51595022	2.84132545	0.00449264	0.05534725	2.34749801	1.25690399
COL25A1	25.2323984	7.99660206	2.8156308	2.84007479	0.0045103	0.0554008	2.34579498	1.256483954
RANBP2	20.9662503	6.58622457	2.32051721	2.83825715	0.00453606	0.05555342	2.343321	1.255289192
RAB11B	34.545598	7.13143359	2.51677465	2.83356064	0.00460326	0.05621104	2.33693474	1.250178401
ZNF121	47.764575	8.33453431	2.94290075	2.83208135	0.00462461	0.05630663	2.33492506	1.249440442
KRIT1	7.89025996	8.36158255	2.95719255	2.82754079	0.0046907	0.05694487	2.32876198	1.244545378
GLRX5	24.2058848	8.12672768	2.8752774	2.82641517	0.00470722	0.05697927	2.32723541	1.244283135
MTRNR2L7	4.64531942	6.16782799	2.1875725	2.81948507	0.00481008	0.05786117	2.31784798	1.237612776
SERPINH1	11.1259502	7.17475155	2.54992482	2.81371101	0.00489732	0.05859901	2.31004123	1.232109753
COL2A1	11.9331708	7.48576321	2.66347034	2.81052997	0.004946	0.05879226	2.30574604	1.230679861
MPST	7.11206734	6.76261051	2.4067165	2.80989078	0.00495583	0.05879226	2.30488347	1.230679861
GID8	7.26322401	6.82302766	2.43077504	2.80693505	0.00500153	0.05916586	2.30089691	1.227928849
NBPF12	16.1330108	6.4653455	2.30883067	2.80026837	0.00510601	0.06013734	2.29191803	1.220855769
RPS10	17.1467079	7.79199878	2.78375271	2.79909877	0.00512455	0.06013734	2.29034461	1.220855769
TMEM231	10.0597219	7.73822932	2.77038412	2.79319726	0.00521899	0.06087355	2.2824139	1.2155714
SP100	14.8551345	7.85655641	2.81463141	2.79132691	0.00524924	0.06105544	2.27990336	1.21427567
NCAPG	7.43907468	7.39000003	2.64847288	2.79028722	0.00526613	0.06108124	2.27850841	1.214092138
TSPO	7.94514819	8.24633725	2.95716561	2.78859501	0.00529372	0.06123072	2.27623889	1.213030669
CFHR4	7.27385839	7.35601437	2.64182168	2.78444772	0.0053619	0.06180064	2.27068158	1.209007053
CENPC	8.475245	7.04906896	2.53217851	2.78379622	0.00537268	0.06180064	2.2698092	1.209007053
CPSF6	15.7828266	5.84122347	2.0994859	2.7822161	0.00539891	0.06193127	2.26769409	1.208089994
CRLF3	10.2893033	6.47273147	2.32793768	2.78045737	0.00542824	0.06209666	2.26534107	1.20693173
RYR3	11.3476358	6.74262506	2.42696005	2.77821839	0.00546579	0.06235489	2.26234731	1.205129453
RAB6A	25.3542065	6.60012155	2.38008877	2.77305688	0.00555324	0.06302485	2.25545345	1.2004882
RNF213	67.2891083	4.66440945	1.68210089	2.77296652	0.00555478	0.06302485	2.25533286	1.2004882
SLC25A39	613.780855	8.0082052	2.89208057	2.76901179	0.00562266	0.06358307	2.25005818	1.19665853
BUB3	6.73104663	8.13504161	2.93868276	2.76826125	0.00563563	0.06358307	2.24905784	1.19665853
CTNND1	8.52056528	7.14702346	2.58253681	2.76744301	0.00564979	0.06358307	2.24796753	1.19665853
P4HA2	9.20941526	7.43306284	2.69058819	2.76261632	0.00573401	0.0642713	2.24154135	1.191982942
PSAP	520.672119	6.29627178	2.27946377	2.76217235	0.00574182	0.0642713	2.24095072	1.191982942
CENPE	9.94792868	7.06201982	2.55881293	2.75988124	0.00578224	0.06455024	2.23790406	1.190102169
C16orf58	12.6776508	8.14642591	2.95578419	2.75609631	0.00584958	0.06512371	2.23287554	1.186260861
CHCHD1	12.5456174	8.14188438	2.95579164	2.75455288	0.00587724	0.06512371	2.23082663	1.186260861
SERBP1	113.791046	5.08455844	1.84888069	2.75007385	0.00595818	0.06563459	2.22488613	1.182867211
DDX27	5.80600058	6.76615777	2.46316257	2.74693918	0.00601543	0.06605873	2.22073341	1.180069797
COPS3	85.678507	7.99977469	2.91304492	2.74618995	0.00602918	0.06605873	2.21974143	1.180069797
DCUN1D1	115.712813	6.7129988	2.4455281	2.74500988	0.00605091	0.06605873	2.21817948	1.180069797
TRPT1	21.0883325	6.77317129	2.46832105	2.74403984	0.00606882	0.06605873	2.21689594	1.180069797

UBB	81.0798665	6.17094609	2.25140146	2.74093545	0.00612645	0.06628127	2.21279081	1.178609165
LAMTOR5	12.1995314	8.10152408	2.95585892	2.74083584	0.00612831	0.06628127	2.21265916	1.178609165
UBN1	20.3298984	4.69022739	1.71265403	2.73857259	0.00617065	0.06656633	2.20966883	1.176745415
LRRC40	8.49900118	6.49328204	2.38512561	2.72240674	0.00648083	0.06937323	2.18836921	1.158808095
CDC42SE1	72.0080185	7.15740997	2.63273117	2.71862546	0.00655538	0.06999128	2.18340219	1.154956075
DDB1	144.293207	6.71701965	2.47273782	2.71643019	0.00659901	0.07027694	2.18052114	1.153187178
RMND5A	292.293288	6.20431367	2.29060582	2.70859072	0.00675696	0.07124465	2.17024842	1.147247739
MYADM	6.20188976	6.85621687	2.53195879	2.70787065	0.00677164	0.07124465	2.16930608	1.147247739
CREB5	3.93289048	5.33702812	1.9749571	2.70235141	0.0068851	0.07207168	2.16209006	1.142235357
ATP1B2	13.8366424	5.92361743	2.19216436	2.7021776	0.0068887	0.07207168	2.16186301	1.142235357
DCP2	13.7746608	7.58894883	2.81457676	2.69630196	0.00701141	0.07317168	2.15419478	1.135656958
JAZF1	11.1239721	7.96676553	2.95609757	2.69502794	0.00703827	0.07326842	2.1525339	1.135083166
FADS1	115.06756	4.93269039	1.8329709	2.6910904	0.00712189	0.07386334	2.14740477	1.131571055
CDK12	26.8733256	5.98611393	2.22536014	2.68995288	0.00714621	0.07386334	2.14592418	1.131571055
SCARB1	8.15744662	7.25675583	2.69783963	2.68983958	0.00714864	0.07386334	2.14577673	1.131571055
LPAR2	9.78224558	7.51318941	2.79524905	2.68784257	0.00719153	0.07393957	2.14317875	1.131123052
ITSN2	139.026633	4.63478387	1.7250676	2.68672593	0.00721561	0.07394585	2.14172676	1.131086176
HIF1A	10.9017089	7.93924002	2.95614909	2.68566969	0.00723846	0.07394585	2.14035377	1.131086176
FAM210B	12.5928892	7.5650618	2.81716171	2.6853488	0.00724541	0.07394585	2.13993673	1.131086176
UQCR11	25.948633	5.9410579	2.21346627	2.6840517	0.00727359	0.0739688	2.13825144	1.13095143
FUT1	5.82853674	7.92580139	2.95851532	2.67897933	0.0073847	0.07463561	2.13166742	1.127053914
HEMGN	2167.95373	5.01291247	1.87179973	2.67812437	0.00740357	0.07464522	2.13055868	1.126998012
CSNK2A2	11.667148	7.89927344	2.95597532	2.67230697	0.00753317	0.07576841	2.12302222	1.120511843
DNAJB14	33.8594788	6.00284338	2.25039543	2.6674616	0.00764266	0.07650011	2.11675533	1.116337939
EEF1B2	8.42909685	7.88311345	2.95695701	2.6659547	0.007677	0.07659078	2.11480826	1.115823493
GTF2H3	8.59698871	7.31344867	2.74571346	2.66358772	0.00773123	0.07659078	2.11175168	1.115823493
ZNF99	6.21203444	5.73165881	2.15324479	2.66187051	0.00777078	0.07659078	2.10953557	1.115823493
FBXO30	10.5204196	6.8145497	2.56046447	2.6614506	0.00778048	0.07659078	2.10899385	1.115823493
ZNF582	3.9634579	6.48026571	2.43626034	2.65992332	0.00781584	0.07674281	2.1070241	1.114962286
MEAF6	10.0022682	7.81404473	2.93892483	2.65881068	0.0078417	0.07674281	2.1055897	1.114962286
NCBP1	6.2012362	6.31756775	2.37793095	2.65674988	0.0078898	0.07693937	2.10293425	1.113851402
ZBTB1	108.632135	7.59387729	2.86327715	2.65216285	0.0079978	0.07737423	2.0970297	1.111403676
NSUN3	6.01771093	7.84482959	2.95836122	2.65174839	0.00800762	0.07737423	2.09649659	1.111403676
WWP2	6.58981264	7.18915817	2.71114845	2.65170215	0.00800872	0.07737423	2.09643713	1.111403676
CDC34	5.25260973	6.30411037	2.37945493	2.64939263	0.00806366	0.07772471	2.09346785	1.109440877
SERINC1	433.308621	6.0563661	2.28668782	2.64853211	0.00808422	0.07774291	2.09236204	1.109339215
VCP	190.053563	5.51690713	2.08525006	2.64568132	0.00815266	0.07822043	2.08870077	1.106679776
BSG	171.159897	5.87680503	2.22201118	2.64481345	0.0081736	0.07824105	2.0875868	1.106565336
SMARCC1	10.9991186	5.85871275	2.21626926	2.64350223	0.00820532	0.07834188	2.08590435	1.106006034
GTF2H5	5.65053726	6.99775098	2.64783012	2.64282475	0.00822176	0.07834188	2.08503532	1.106006034
PSMB7	40.0794534	7.73405144	2.93215512	2.6376679	0.00834783	0.07936155	2.07842646	1.100389852
LMO7	5.67035137	5.90291737	2.23945517	2.63587208	0.00839214	0.07950642	2.07612748	1.099597781
MAP2K3	369.838145	6.15350293	2.33484906	2.63550353	0.00840126	0.07950642	2.07565582	1.099597781
EMC3	89.6464732	5.99941456	2.27805904	2.63356412	0.00844939	0.07978062	2.07317475	1.098102615
RUFY4	3.86941197	5.64539773	2.14844725	2.62766411	0.00859734	0.08063092	2.0656361	1.093498382
RBM4	5.98864799	7.13496678	2.71533486	2.62765631	0.00859753	0.08063092	2.06562613	1.093498382
RUVBL2	13.9668446	7.75531955	2.95557516	2.62396289	0.00869133	0.08104703	2.06091399	1.091262897
XPNPEP3	8.32023974	6.29195405	2.39818927	2.62362697	0.0086999	0.08104703	2.06048568	1.091262897
MORN2	8.4987578	7.58302642	2.89030068	2.62361161	0.00870029	0.08104703	2.06046609	1.091262897
PSMD10	9.61251602	7.74011976	2.95655232	2.61795461	0.00884586	0.08221908	2.05326008	1.085027372
KLRD1	16.2154064	5.6492702	2.15918585	2.61638904	0.00888652	0.08241311	2.05126807	1.08400369
AGO2	426.110641	4.01923389	1.53783941	2.61355891	0.00896046	0.08288789	2.04766953	1.081508907
TMEM9B	9.39917358	7.72528535	2.95658462	2.61290859	0.00897753	0.08288789	2.04684309	1.081508907
RPL21	174.776079	4.64721009	1.77923852	2.61190955	0.00900381	0.08294658	2.04557381	1.081201527
RPL17	16.9983803	5.79044537	2.22105732	2.60706706	0.00913215	0.08394318	2.03942709	1.076014566
SLC11A2	100.852287	6.98390673	2.68261963	2.60339061	0.00923067	0.08466193	2.03476668	1.07231181
SPTAN1	5.56709154	5.83997439	2.24458812	2.60180223	0.00927353	0.0848681	2.03275486	1.071255536
UBL5	22.8317443	7.37562108	2.83851747	2.59840609	0.00936577	0.08515076	2.0284567	1.06981146
JCHAIN	8.88467348	7.10029657	2.73782617	2.59340665	0.00950303	0.08602352	2.02213774	1.065382785
REEP1	8.04462589	7.50101488	2.89754291	2.58875023	0.00963249	0.08700586	2.01626129	1.06045149
SH3BGRL	105.535447	7.39746703	2.85866638	2.58773359	0.00966097	0.08707417	2.01497942	1.060110673
USP6NL	40.2638413	4.39698629	1.70048839	2.58571967	0.00971759	0.08739537	2.0124413	1.058511569

TMEM267	4.76285422	6.75043172	2.61228475	2.58411022	0.00976306	0.08761504	2.01041409	1.057421348
HSPA1B	4.95215037	5.99519781	2.32488523	2.578707	0.00991709	0.08827385	2.0036159	1.054167936
PHF6	10.7929247	3.47282358	1.34696777	2.57825292	0.00993013	0.08827385	2.00304512	1.054167936
ATP5J2	15.9492886	6.77533394	2.62831536	2.57782382	0.00994247	0.08827385	2.00250581	1.054167936
ZNF529	25.5332271	5.54689131	2.1581905	2.57015834	0.0101652	0.08986817	1.9928839	1.046394086
SEC11C	4.55377189	7.60110007	2.95992183	2.56800703	0.01022851	0.09023625	1.9901877	1.044618981
RPL14	70.2222207	5.42108513	2.11208748	2.56669536	0.01026728	0.09038677	1.98854471	1.043895132
CKS1B	8.61811781	7.58052894	2.95691773	2.56365906	0.01035752	0.09079732	1.98474409	1.041926983
CELA2A	8.61811781	7.58052894	2.95691773	2.56365906	0.01035752	0.09079732	1.98474409	1.041926983
SNAPC2	10.1565156	7.57020808	2.95635835	2.56065307	0.01044756	0.0912662	1.98098501	1.039690044
COX7C	8.50402757	7.56867874	2.9569465	2.55962654	0.01047847	0.09128106	1.97970213	1.039619304
MLF2	154.755017	6.59701793	2.57934177	2.55763622	0.01053863	0.09161345	1.97721595	1.038040746
KDM5A	124.289367	5.2659484	2.05980487	2.5565278	0.01057226	0.09171438	1.97583206	1.037562581
VIP	34.6046191	5.340077	2.0915389	2.55318082	0.01067441	0.09240796	1.97165627	1.034290622
RCC1L	4.36044755	5.81696443	2.28237687	2.54864326	0.01081429	0.09293382	1.96600215	1.031826232
CEP170B	3.76188008	5.86454787	2.30119135	2.54848337	0.01081924	0.09293382	1.96580306	1.031826232
PCK2	8.23986876	7.53544411	2.95702842	2.54831643	0.01082442	0.09293382	1.96559521	1.031826232
CDH2	7.68019651	6.90022605	2.71246163	2.54389813	0.01096231	0.09335749	1.96009811	1.029850813
TRABD	7.60550186	6.89326155	2.70976283	2.54386158	0.01096345	0.09335749	1.96005267	1.029850813
ASH2L	18.1514367	6.71456167	2.6421163	2.54135735	0.0110423	0.09383701	1.95694047	1.027625831
NAA16	13.7094056	6.71899217	2.65148686	2.53404694	0.01127536	0.09562242	1.94786948	1.019440252
FASTK	8.12711525	7.49104534	2.95709357	2.53324596	0.01130116	0.09564642	1.94687689	1.019331272
WWTR1	4.47591575	5.51337572	2.18014131	2.52890751	0.01144182	0.09664042	1.94150498	1.014841181
RIOX2	7.89809038	7.47604856	2.95717956	2.52810098	0.01146814	0.09666664	1.94050716	1.014723388
RAD54L2	8.69443532	7.47178194	2.95685492	2.52693559	0.01150626	0.09679206	1.93906581	1.014160286
CSNK2A1	6.81575473	6.46206244	2.55962375	2.52461419	0.01158254	0.09684675	1.93619631	1.013914934
LHFPL2	9.33400148	7.45070834	2.95661159	2.52001594	0.01173495	0.09772868	1.93051868	1.009977965
HAUS2	6.96753791	6.46925881	2.57028652	2.51694072	0.01183787	0.09838904	1.92672627	1.007053273
TP53	11.8068652	5.12881684	2.03847059	2.51601218	0.01186911	0.09845212	1.92558191	1.006774911
TMEM138	4.54814881	6.67528284	2.65672496	2.51259839	0.01198457	0.09904299	1.92137761	1.004176258
SENP1	40.4468355	6.42777903	2.55850275	2.51232055	0.01199401	0.09904299	1.92103563	1.004176258
ANP32E	9.30744149	7.42655346	2.95666535	2.51180048	0.0120117	0.09904299	1.9203956	1.004176258
RPS6KB1	7.04017485	7.31194784	2.91209873	2.51088597	0.01204286	0.09910369	1.9192704	1.003910195
ATP6V1A	30.3421407	6.50094154	2.59018498	2.50983678	0.0120787	0.09920256	1.91797989	1.003477127
IARS	10.9555411	7.416904	2.9561448	2.50897859	0.01210808	0.09924814	1.91692463	1.003277625
TMC6	9.99057873	5.35133361	2.13353167	2.50820444	0.01213464	0.09927044	1.91597298	1.003180037
LRRC8C	9.05125522	7.40623126	2.95671128	2.50488822	0.01224901	0.10000956	1.91189902	0.999958474
PHOSPHO1	40.336981	7.31110706	2.92111122	2.50285131	0.01231973	0.10039012	1.90939885	0.99830903
TOMM5	7.90906038	7.36101499	2.94591824	2.49871666	0.01246439	0.10122639	1.90432888	0.99470625
ADAT1	22.4099575	6.02930628	2.41491843	2.49669148	0.0125358	0.10122639	1.90184805	0.99470625
SEC62	95.0132764	5.3555845	2.14573903	2.49591606	0.01256323	0.10122639	1.90089859	0.99470625
WNK1	397.108378	4.88203461	1.95601354	2.49591043	0.01256343	0.10122639	1.90089171	0.99470625
TOMM20	6.71823094	7.23664051	2.90028699	2.49514636	0.01259052	0.10122639	1.89995638	0.99470625
RNF114	28.0947076	6.73442933	2.69975526	2.4944592	0.01261492	0.10122639	1.8991154	0.99470625
ATXN7L1	8.46492259	7.3746329	2.95694449	2.49400451	0.01263109	0.10122639	1.89855904	0.99470625
ATG2A	8.97921431	7.37340495	2.95678683	2.49372219	0.01264114	0.10122639	1.89821363	0.99470625
GALNT10	173.979788	3.99450446	1.60280311	2.4921991	0.01269549	0.10146641	1.8963507	0.993677703
TMUB2	4.82017958	6.22222297	2.49781106	2.49107031	0.01273589	0.10159435	1.89497066	0.993130439
SAP30BP	5.16123942	6.32765505	2.54157029	2.4896636	0.01278641	0.10168409	1.89325152	0.992746982
MLLT10	5.76783352	5.65788526	2.2733696	2.48876613	0.01281873	0.10168409	1.89215514	0.992746982
RPL29	67.3063971	6.57813104	2.64355918	2.48836156	0.01283332	0.10168409	1.89166101	0.992746982
FLII	124.835698	6.15214514	2.47268442	2.48804299	0.01284482	0.10168409	1.89127196	0.992746982
IDS	16.8746346	7.09576632	2.85576245	2.48471868	0.01296538	0.10209638	1.88721459	0.990989662
OTUD7B	5.309056	6.36182118	2.56595667	2.47931746	0.01316341	0.10325837	1.88063163	0.986074722
ANXA7	296.967064	5.30765173	2.14087111	2.47920191	0.01316767	0.10325837	1.88049092	0.986074722
KIAA0907	7.13148968	7.32691614	2.9575873	2.47732878	0.01323699	0.103598	1.87821075	0.984648636
MCTP2	7.1059249	7.32503706	2.95759271	2.47668891	0.01326074	0.103598	1.87743215	0.984648636
GCLM	4.48227435	6.12273191	2.47740867	2.47142588	0.01345754	0.10461843	1.87103417	0.980391809
AKIRIN1	236.334912	5.88102484	2.3796097	2.47142413	0.01345761	0.10461843	1.87103205	0.980391809
RAB4A	3.74172762	7.31795194	2.96131669	2.47118181	0.01346673	0.10461843	1.87073774	0.980391809
UBE4A	5.05259299	4.14166129	1.67865131	2.46725527	0.01361533	0.10533289	1.86597197	0.977435984
ZDHHC3	8.05969205	6.94677032	2.81674495	2.46624044	0.01365396	0.10533289	1.86474123	0.977435984

SUMO1	6.97015251	7.29389899	2.95768334	2.46608516	0.01365989	0.10533289	1.86455295	0.977435984
ALG2	6.93829671	7.29068012	2.95769282	2.46498895	0.01370175	0.10537486	1.86322404	0.977262969
ATG13	25.5364446	6.02989126	2.44658253	2.46461796	0.01371594	0.10537486	1.8627744	0.977262969
FUT8	3.86067137	5.62423316	2.28575267	2.46056068	0.01387201	0.10583361	1.85786058	0.975376387
PGS1	9.72538653	6.38247333	2.59399165	2.46048338	0.013875	0.10583361	1.85776701	0.975376387
PLAA	5.79051559	7.03398288	2.86039211	2.45909743	0.01392868	0.10598815	1.85609	0.974742686
CLDND1	6.84834764	7.27188292	2.95774859	2.45858723	0.01394849	0.10598815	1.85547285	0.974742686
PIM2	87.4664509	5.68626555	2.31429734	2.45701598	0.01400965	0.10625895	1.85357286	0.97363448
SF3B5	7.49672141	7.25903216	2.95739873	2.45453279	0.01410678	0.10680115	1.85057211	0.971424077
GFOD2	5.65693186	5.94841423	2.42476188	2.45319522	0.01415935	0.10700458	1.84895677	0.970597644
TMC3	5.8972171	7.05406455	2.87643539	2.45236328	0.01419213	0.10705802	1.84795242	0.970380779
XK	488.512977	5.66077056	2.30942422	2.45116099	0.01423962	0.10722206	1.84650145	0.969715861
LAPTM4A	6.73118337	7.24705606	2.95782337	2.45013145	0.01428041	0.10731306	1.84525941	0.969347428
DCK	3.55878408	7.25421651	2.96171725	2.44932784	0.01431231	0.10731306	1.84429023	0.969347428
HSPE1	6.72222217	7.24346252	2.9578343	2.44890748	0.01432903	0.10731306	1.84378336	0.969347428
PWP1	6.62762671	7.22473772	2.95789168	2.44252951	0.01458474	0.10903204	1.8361014	0.962445869
TSSC4	11.4966911	7.21788655	2.95594984	2.44181631	0.01461358	0.10905188	1.83524338	0.962366857
ZNFX1	5.51246046	5.85919882	2.40054197	2.44078166	0.01465551	0.10911991	1.83399899	0.96209601
ECSIT	6.6039793	7.21818967	2.95791192	2.44029906	0.01467511	0.10911991	1.8334187	0.96209601
ACTB	843.967064	3.59398713	1.47346271	2.43914358	0.01472212	0.10927433	1.83202973	0.961481866
KLF1	6.57563396	7.20979892	2.95793799	2.43744086	0.01479163	0.10952495	1.82998386	0.960486918
AVIL	6.30991793	7.15041816	2.93620535	2.4352582	0.01488117	0.10983991	1.82736302	0.959239831
ZMIZ2	6.40758062	6.6413087	2.72776446	2.43470754	0.01490383	0.10983991	1.8267021	0.959239831
ATP7A	6.51706475	7.19870669	2.95797268	2.43366233	0.01494693	0.10996294	1.82544795	0.958753662
R3HDM2	79.159988	4.06551601	1.6714156	2.43237888	0.01500001	0.10996484	1.82390853	0.958746141
CXADR	5.54860547	6.97101489	2.86717649	2.43131698	0.01504405	0.11009387	1.82263533	0.958236873
RAD23A	6.4574426	7.18365579	2.95802018	2.42853508	0.01515996	0.11059065	1.819302	0.956281604
NUCB2	4.23117191	4.61426248	1.90176682	2.42630297	0.01525353	0.11104143	1.81662963	0.954514936
PITHD1	6.39309694	7.17323879	2.95805334	2.42498629	0.01530897	0.11125049	1.81505417	0.95369807
PIGT	103.155179	5.43966097	2.24702503	2.42082794	0.0154852	0.11233518	1.81008305	0.949484219
GOLGA4	8.17401979	6.36371481	2.62990835	2.41974775	0.01553128	0.11243023	1.80879284	0.949116885
ATG101	6.44976276	7.15641778	2.95810739	2.41925557	0.01555231	0.11243023	1.80820512	0.949116885
RASAL2	15.0915654	5.51131446	2.27915925	2.41813487	0.01560029	0.11247225	1.80686723	0.948954614
RHBDD1	5.6246149	6.14800905	2.54274963	2.4178586	0.01561214	0.11247225	1.8065375	0.948954614
MAP2K2	131.159857	4.87734996	2.01797965	2.41694705	0.01565129	0.11255956	1.80544976	0.948617598
RAN	6.64051772	6.69029697	2.77148666	2.41397408	0.01577958	0.11298242	1.80190443	0.946989142
USP9X	618.561712	3.69828967	1.5321539	2.41378471	0.01578779	0.11298242	1.80167872	0.946989142
CCT8	9.49596402	5.8507308	2.42396842	2.41369926	0.01579149	0.11298242	1.80157688	0.946989142
RBM15	2.74795912	5.41057634	2.24310352	2.41209391	0.0158612	0.11311837	1.79966408	0.94646687
YOD1	428.255791	5.19523269	2.15405016	2.41184388	0.01587208	0.11311837	1.79936626	0.94646687
EPSTI1	33.2736271	6.57111946	2.72503764	2.41138668	0.01589199	0.11311837	1.79882172	0.94646687
GLYATL1	6.19834842	7.12836902	2.95819891	2.40969902	0.01596569	0.113449	1.79681243	0.945199329
PRRC2B	5.5397035	6.15639862	2.55773157	2.40697605	0.01608522	0.11384798	1.79357287	0.943674685
MARS	4.7139978	6.46717263	2.68731749	2.40655324	0.01610386	0.11384798	1.79307011	0.943674685
TMEM94	4.16074869	6.01681479	2.50184702	2.40494912	0.01617472	0.11415513	1.79116329	0.942504583
ZCCHC8	8.33368591	5.54535982	2.30789484	2.40277838	0.01627104	0.1144893	1.78858456	0.941235107
TMEM208	6.10984337	7.10766178	2.95826761	2.40264328	0.01627706	0.1144893	1.78842412	0.941235107
MYO1E	6.03367962	7.08926273	2.95832947	2.39637363	0.0165582	0.11627041	1.78098683	0.934530798
ABCF1	6.8769303	7.00948381	2.938931	2.38504538	0.01707701	0.1197116	1.76758805	0.921863777
C22orf15	10.9223218	5.7872641	2.43012305	2.38146957	0.01724372	0.12050266	1.76336915	0.919003354
ETS1	2.78456353	5.41709178	2.27476653	2.38138363	0.01724774	0.12050266	1.76326782	0.919003354
TMEM187	6.96492183	7.02837536	2.95769281	2.37630336	0.01748708	0.12197018	1.75728272	0.913746326
TPD52L2	6.9529762	7.02150698	2.95771311	2.37396486	0.01759823	0.12254015	1.75453112	0.911721587
ELOA	6.08677303	5.7711114	2.43620543	2.36889357	0.01784139	0.12384964	1.7485714	0.907105264
DNAJB1	121.204299	4.56121208	1.92780985	2.36600725	0.01798109	0.12458114	1.74518393	0.904547685
LRRC47	3.68470145	5.56542561	2.35307746	2.3651689	0.01802185	0.12465612	1.74420063	0.904286398
CCT2	29.0868472	6.05152608	2.56019198	2.36370011	0.01809345	0.12466798	1.74247857	0.904245071
MICB	6.0113876	5.99582598	2.53752218	2.36286643	0.0181342	0.12466798	1.7415015	0.904245071
FGFR1OP2	122.930758	4.25570926	1.80333214	2.35991427	0.01827916	0.12502996	1.7380438	0.902985915
TTF2	12.409294	5.81592571	2.46490875	2.35948925	0.01830011	0.12502996	1.73754628	0.902985915
GUCD1	35.1310725	5.87438388	2.49000229	2.35918815	0.01831497	0.12502996	1.73719385	0.902985915
GLIPR1L1	4.62011983	6.70414763	2.85237207	2.35037627	0.01875444	0.12760375	1.72689586	0.894136559

MRPS36	6.49014004	6.92566297	2.9474825	2.34968756	0.01878918	0.12763153	1.72609228	0.894042039
TAB3	33.3282401	6.50209995	2.76833013	2.34874442	0.01883683	0.12774685	1.72499215	0.893649799
LRRC14	5.88358694	6.51532788	2.77645936	2.34663182	0.01894396	0.12814939	1.72252914	0.892283466
SENP7	6.55460088	6.94044187	2.95796001	2.34636095	0.01895774	0.12814939	1.72221347	0.892283466
AGO1	55.6352063	5.89791084	2.51722538	2.34302057	0.01912833	0.12898497	1.71832298	0.889460906
PTAR1	99.7515614	6.33934864	2.70596799	2.34272862	0.0191433	0.12898497	1.71798315	0.889460906
SEC13	4.13325103	5.24187978	2.24061502	2.33948256	0.01931047	0.12967998	1.71420709	0.887127062
ANKRD27	6.82593621	6.46326441	2.76336122	2.33891406	0.01933988	0.12967998	1.71354618	0.887127062
C1D	6.40922957	6.90444068	2.95807413	2.33409995	0.01959049	0.13086597	1.70795473	0.883173279
METTL22	6.42374844	6.90284221	2.95807926	2.33355553	0.01961901	0.13086597	1.70732297	0.883173279
PSMB5	7.65068898	6.89672629	2.95732433	2.33208316	0.01969632	0.13086597	1.70561498	0.883173279
USH2A	2.9880704	6.07651829	2.60639199	2.3313908	0.01973276	0.13086597	1.70481212	0.883173279
ZNHIT1	83.05835	5.26730253	2.25934104	2.3313446	0.0197352	0.13086597	1.70475855	0.883173279
MRVI1	6.36902275	6.89365429	2.95810887	2.33042616	0.01978364	0.13086597	1.70369383	0.883173279
CYBB	5.26575675	6.89333998	2.95903869	2.32958765	0.01982796	0.13086597	1.70272206	0.883173279
ACP1	191.551446	4.87847805	2.09465491	2.32901277	0.01985839	0.13086597	1.70205599	0.883173279
RPS7	10.5970569	6.07375753	2.60818812	2.32872679	0.01987354	0.13086597	1.70172468	0.883173279
MRFAP1	50.8483081	5.34662994	2.29632387	2.32834315	0.01989389	0.13086597	1.70128029	0.883173279
WDR33	8.71227149	5.38449251	2.31472573	2.32619029	0.0200084	0.13141165	1.69878761	0.881366144
PLAUR	7.51356439	6.87012765	2.9573967	2.32303216	0.02017742	0.13183713	1.69513427	0.879962245
EML4	54.1158021	4.31585749	1.85906421	2.32152148	0.02025872	0.13183713	1.69338808	0.879962245
AP1M2	5.19128997	6.86869191	2.95913481	2.32118249	0.020277	0.13183713	1.69299637	0.879962245
DGCR8	3.47228635	5.74886244	2.47689645	2.32099426	0.02028715	0.13183713	1.69277888	0.879962245
NDUFA1	5.30345712	6.86769729	2.95913873	2.3208433	0.0202953	0.13183713	1.69260447	0.879962245
UVRAG	4.66417001	6.72343722	2.89743789	2.32047674	0.0203151	0.13183713	1.69218099	0.879962245
WDR75	5.16548876	6.86428928	2.95915215	2.31968109	0.02035814	0.13183713	1.69126199	0.879962245
MED28	42.2023242	4.80109276	2.07034729	2.31897942	0.02039615	0.13187823	1.69045175	0.879826901
ZNF30	310.911741	6.70149679	2.89146201	2.31768454	0.02046647	0.13212773	1.68895702	0.879006011
MED22	6.18751917	6.85335697	2.95824095	2.31670006	0.02052008	0.13226873	1.68782103	0.878542824
ATG14	4.85320149	6.77638184	2.9306722	2.31222784	0.02076513	0.13324277	1.68266533	0.87535634
ESR2	2.97663377	4.99087486	2.15899429	2.31166654	0.02079607	0.13324277	1.6820188	0.87535634
PITPNB	5.67239207	6.73159615	2.92440693	2.30186712	0.02134267	0.13630501	1.67075131	0.865488183
POLR1D	5.99305355	6.80806538	2.9583938	2.30127084	0.02137633	0.13631091	1.67006692	0.865469377
SMARCAD1	7.57891314	6.04899187	2.62957105	2.30037209	0.02142715	0.13642607	1.66903562	0.865102647
SLC25A46	5.64760358	6.72341855	2.92919345	2.29531394	0.02171514	0.13804861	1.66323738	0.859967961
MAPRE1	4.37304528	6.62517201	2.88916937	2.29310614	0.0218419	0.13864276	1.66070968	0.858102801
SRP14	10.7259687	5.57947984	2.43800155	2.28854647	0.02210572	0.14010381	1.65549539	0.85355004
PQLC1	267.273761	5.67482605	2.48082341	2.28747682	0.02216801	0.14028508	1.65427335	0.852988524
MITD1	4.81538503	6.76513092	2.95955678	2.28585948	0.02226248	0.14048518	1.65242645	0.852369475
FRG1	8.57721474	6.01087635	2.63096321	2.28466758	0.02233233	0.14068352	1.65106602	0.851756759
GRPEL1	4.53030028	6.67236171	2.92236022	2.28320987	0.02241801	0.14100995	1.64940296	0.850750232
ARHGEF2	2.75223652	4.70428271	2.06595123	2.2770541	0.022783	0.14233724	1.64238918	0.846681447
ATP13A3	6.62193959	4.48009073	1.96917911	2.27510575	0.02289959	0.14233724	1.64017236	0.846681447
ATAD2B	197.86984	4.3838998	1.92701318	2.27497136	0.02290765	0.14233724	1.6400195	0.846681447
HSP90AA1	216.318138	4.53310607	1.99263677	2.27492845	0.02291022	0.14233724	1.6399707	0.846681447
PANK3	112.482808	6.58661719	2.89586823	2.27448788	0.02293667	0.14233724	1.63946965	0.846681447
ELOF1	4.881984	5.46373039	2.40386248	2.2728964	0.02303242	0.14271877	1.63766036	0.845518914
ABCB10	33.517034	6.62028815	2.9134398	2.27232707	0.02306676	0.14271917	1.63701335	0.845517696
ID1	4.68919614	6.72201269	2.95974143	2.27114863	0.02313798	0.14276506	1.63567452	0.845378065
ZKSCAN1	5.30807152	5.53511368	2.43722743	2.27106983	0.02314275	0.14276506	1.63558501	0.845378065
NEDD8	4.64619206	6.7169053	2.95976366	2.26940596	0.02324365	0.14278572	1.63369567	0.845315238
SLC45A4	30.2708146	3.86364575	1.70250067	2.26939455	0.02324434	0.14278572	1.63368272	0.845315238
IFT52	4.67216097	6.71664865	2.95976478	2.26931839	0.02324897	0.14278572	1.63359626	0.845315238
RIMKLB	33.6296486	4.80424376	2.11861312	2.26763618	0.02335139	0.14320352	1.63168726	0.844046309
PRMT9	5.57620287	6.707424	2.95875084	2.26697832	0.02339155	0.14322143	1.63094099	0.843991988
PLAG1	34.4108264	5.42604666	2.39499835	2.26557428	0.02347746	0.14334333	1.62934887	0.843622523
HDLBP	18.2167957	5.42062869	2.39452894	2.26375576	0.02358914	0.14381432	1.62728788	0.842197863
USP8	49.7827837	4.91236613	2.17173745	2.26195212	0.02370036	0.14426178	1.62524503	0.84084872
ACOT13	4.15786246	6.54207168	2.893895	2.26064583	0.0237812	0.14426178	1.62376629	0.84084872
ACAD8	8.16631134	6.68438848	2.95687356	2.26062709	0.02378236	0.14426178	1.62374508	0.84084872
MBD3	10.1701606	6.04103213	2.67263925	2.26032456	0.02380111	0.14426178	1.6234027	0.84084872
GALNT4	4.66976483	6.68526429	2.95990313	2.25860915	0.02390771	0.14426385	1.62146205	0.840842478

TMPRSS11A	4.54417871	6.68468076	2.95990573	2.25841002	0.02392011	0.14426385	1.62123685	0.840842478
BMI1	14.6753772	6.44825509	2.85744616	2.25664973	0.02402997	0.14459653	1.61924676	0.839842137
ATP6V1B2	6.52929465	6.66322824	2.95800693	2.25260738	0.02428392	0.14496674	1.61468129	0.838731641
PTPRF	109.027761	4.44295258	1.97261893	2.25231164	0.02430258	0.14496674	1.61434753	0.838731641
MDH1	3.96197027	5.68505766	2.52419806	2.25222329	0.02430816	0.14496674	1.61424783	0.838731641
YIPF6	2.93882327	5.51485432	2.44888157	2.25198898	0.02432297	0.14496674	1.61398343	0.838731641
TMED4	113.299088	5.28603419	2.34993767	2.24943592	0.02448478	0.14564944	1.61110387	0.836691191
KIF3C	14.4789882	6.61917571	2.94487416	2.24769391	0.02459571	0.14610065	1.60914055	0.835347852
ANAPC16	4.47107273	6.63553229	2.96006864	2.2416819	0.02498194	0.14809976	1.60237386	0.829445636
SNRPD1	6.24197048	5.13016753	2.28887361	2.24135028	0.02500339	0.14809976	1.60200103	0.829445636
PDE7B	5.30526901	6.63023973	2.95904176	2.24067122	0.02504738	0.14814956	1.6012377	0.829299641
CALM2	417.358509	4.07921313	1.82256771	2.23816823	0.02521009	0.14890043	1.59842566	0.827104054
MAD2L1	7.61561383	5.53094697	2.47280176	2.23671264	0.02530513	0.14925007	1.59679148	0.826085458
CCT4	23.3136701	6.53086784	2.92223536	2.23488769	0.02542472	0.14974335	1.59474377	0.824652462
FLRT2	3.83542907	5.3296675	2.38741102	2.23240466	0.02558823	0.15049349	1.59195976	0.822482294
DNTTIP2	115.173552	5.5012583	2.46498599	2.23176047	0.0256308	0.15053123	1.59123787	0.822373387
IMP3	4.30493941	6.59856667	2.96030101	2.22901882	0.02581265	0.15085726	1.58816737	0.821433796
HECTD3	4.35462504	6.49861558	2.91582522	2.22873975	0.02583123	0.15085726	1.58785499	0.821433796
XPO5	6.0516928	4.93238094	2.21454042	2.22727068	0.02592919	0.15121729	1.58621106	0.820398555
JAKMIP2	4.23330805	6.58231628	2.96037823	2.22347139	0.02618403	0.15220046	1.5819635	0.817584025
TMEM59	53.3305846	4.9780676	2.2406157	2.22174092	0.02630082	0.1525301	1.58003073	0.816644449
SRSF9	3.66016203	6.37071275	2.86905261	2.22049353	0.02638528	0.15280713	1.57863822	0.815856388
CEP290	2.83194402	5.45985441	2.45972369	2.21970233	0.02643898	0.15290544	1.57775529	0.815577069
ARID3A	26.0628454	4.75095284	2.14267833	2.21729635	0.02660285	0.15356771	1.57507188	0.813700091
PSD4	9.51113164	5.59262322	2.52326587	2.21642249	0.02666258	0.15356771	1.57409781	0.813700091
STRADB	291.321429	4.72514213	2.13238618	2.21589418	0.02669875	0.15356771	1.57350907	0.813700091
NFYA	5.0448721	6.55746531	2.95933044	2.21586113	0.02670101	0.15356771	1.57347225	0.813700091
C2orf49	3.3767885	6.25930127	2.82811957	2.21323785	0.02688125	0.15439108	1.57055051	0.811377791
SLC6A15	5.98563114	6.53353142	2.95326503	2.21230785	0.0269454	0.15454635	1.56951535	0.810941252
C1orf131	4.15303198	6.54667047	2.96055062	2.21130165	0.02701496	0.15455792	1.56839575	0.810908746
HBA1	92.3361091	6.4046467	2.89645106	2.21120487	0.02702165	0.15455792	1.56828809	0.810908746
PMPCA	3.291707	5.9457027	2.69012358	2.21019686	0.0270915	0.15474488	1.5671669	0.810383712
ZBTB25	22.7003259	4.79805862	2.17429255	2.20672173	0.02733351	0.15587166	1.56330465	0.807232828
ROMO1	8.92770366	6.21550475	2.81785657	2.20575626	0.02740107	0.15587166	1.56223247	0.807232828
PPP2R5E	4.92025021	5.36314993	2.43276736	2.20454698	0.0274859	0.15610834	1.56089004	0.8065739
NCOR1	147.630447	4.66947282	2.11887883	2.20374698	0.02754214	0.15610834	1.56000226	0.8065739
MAPK1IP1L	102.869854	4.4521832	2.02080056	2.20317793	0.02758221	0.15610834	1.55937094	0.8065739
AMMECR1L	24.5249461	4.75698825	2.15993692	2.2023737	0.02763892	0.15610834	1.5584789	0.8065739
IDI1	45.848558	6.39448906	2.90424001	2.20177707	0.02768106	0.15610834	1.55781729	0.8065739
PSME3	5.88093756	5.61797362	2.55195755	2.20143694	0.02770511	0.15610834	1.55744018	0.8065739
FMNL3	11.3828907	5.24782353	2.38543369	2.19994526	0.02781078	0.15644198	1.55578684	0.805646685
C7orf50	4.03871002	6.51222841	2.96072121	2.19954125	0.02783946	0.15644198	1.5553392	0.805646685
GCNT4	4.55197146	6.41412803	2.91754034	2.19847107	0.02791555	0.15665818	1.55415375	0.805046937
AXL	4.82782849	2.68395216	1.22230205	2.19581745	0.02810501	0.15729741	1.55121622	0.803278436
FNTB	22.9374925	4.72286579	2.1545307	2.19206242	0.028375	0.15817068	1.54706413	0.800874015
USP49	16.6748763	5.96898882	2.72446366	2.19088582	0.02846006	0.15843272	1.54576425	0.80015513
CCT5	3.97998898	6.48524823	2.96085764	2.19032761	0.02850049	0.15844596	1.54514773	0.80011883
ASIC4	19.7161434	6.39832281	2.92480283	2.18760826	0.02869815	0.15911997	1.5421461	0.798275315
TUBG1	64.166212	6.24888514	2.85855628	2.18602838	0.02881353	0.15954725	1.54040354	0.797110664
MCRIP1	3.44124383	6.28505712	2.87683218	2.18471455	0.02890978	0.15965562	1.53895517	0.796815794
PDE11A	7.68003009	4.78140674	2.18991117	2.18337932	0.02900789	0.15977361	1.53748389	0.796494966
PTRH2	3.88337502	6.45585368	2.96100915	2.18028832	0.0292361	0.16060568	1.53408062	0.794239101
MGME1	3.89346748	6.45352793	2.96102127	2.17949394	0.02929499	0.16071277	1.53320658	0.793949616
METTL23	3.72003976	5.57742644	2.55964425	2.17898501	0.02933278	0.16071277	1.53264674	0.793949616
AHNAK	4.43142929	4.96723885	2.28033703	2.17829154	0.02938434	0.16078369	1.53188408	0.793758015
DNM1L	15.4289175	6.35488681	2.91932322	2.17683564	0.02949283	0.16116556	1.53028351	0.792727758
ETNK1	275.565162	3.90461576	1.79528958	2.17492253	0.02963592	0.16173523	1.52818156	0.791195375
SLC35B4	2.86016676	5.45869075	2.51091885	2.17398135	0.02970654	0.1619084	1.52714799	0.790730626
ZNF146	3.78889137	5.88098993	2.7078896	2.17179826	0.02987088	0.16237906	1.52475194	0.789469989
GJC1	3.02072733	3.31416783	1.52890234	2.16767791	0.0301832	0.16386291	1.5202347	0.785519344
AIG1	23.5570454	5.42566846	2.50614283	2.16494782	0.03039168	0.16453701	1.51724528	0.78373641
KDM5C	4.41273187	6.36863441	2.94223484	2.16455679	0.03042164	0.16453701	1.51681733	0.78373641

DCAF13	3.29477806	6.21873701	2.87305789	2.16450111	0.03042591	0.16453701	1.51675641	0.78373641
GLTSCR1L	3.7533622	6.39420715	2.96133688	2.15922991	0.03083233	0.1660906	1.51099358	0.779654933
RPS5	82.774644	4.80748479	2.22648821	2.15922311	0.03083286	0.1660906	1.51098616	0.779654933
C6orf1	4.06800156	6.25562813	2.89985797	2.15721881	0.03098862	0.16634242	1.50879776	0.778996991
SPTA1	12.5255506	5.82832166	2.70192443	2.15710017	0.03099786	0.16634242	1.50866829	0.778996991
ACSF3	3.72042448	6.38791365	2.96137111	2.15707975	0.03099945	0.16634242	1.50864599	0.778996991
ZNF367	3.83055524	5.89216481	2.73395836	2.15517723	0.03114797	0.16670969	1.50657028	0.778039159
EPN1	4.7600587	4.1218785	1.91389149	2.15366364	0.03126656	0.1671296	1.5049199	0.776946638
GABARAP	59.329972	5.77773701	2.68473328	2.15207114	0.03139175	0.16747824	1.50318445	0.776041608
ZFYVE21	71.0438771	6.1583171	2.86249273	2.15138262	0.03144601	0.16747824	1.50243442	0.776041608
TAF3	3.66797332	6.36778632	2.96148158	2.15020291	0.03153917	0.16747824	1.50114977	0.776041608
TM9SF2	3.64178861	6.36718141	2.96148492	2.14999623	0.03155551	0.16747824	1.50092475	0.776041608
RLIM	32.1416107	4.96022466	2.30738639	2.14971566	0.03157771	0.16747824	1.50061933	0.776041608
CFAP69	3.33884507	5.69215438	2.64841795	2.1492659	0.03161333	0.16747824	1.50012978	0.776041608
SIDT1	5.30293853	6.3555659	2.95908982	2.14781108	0.03172877	0.16787622	1.49854681	0.775010823
SPA17	11.1146306	3.8864124	1.8102563	2.14688517	0.03180242	0.16796149	1.49753977	0.77479028
MARCKS	3.70985201	6.35665386	2.96154333	2.14639908	0.03184115	0.16796149	1.49701121	0.77479028
RNF6	3.63390298	6.3539523	2.96155838	2.14547596	0.03191481	0.16796149	1.49600771	0.77479028
USP4	57.1800574	5.43208778	2.53207759	2.14530858	0.03192818	0.16796149	1.4958258	0.77479028
AQP1	5.23368974	6.34575448	2.95912823	2.14446755	0.03199544	0.16796149	1.49491188	0.77479028
BLOC1S6	7.43576265	4.89203879	2.28143569	2.14428082	0.03201039	0.16796149	1.494709	0.77479028
ENTPD5	5.28769604	5.60405213	2.61410273	2.14377655	0.0320508	0.16796149	1.49416119	0.77479028
IRGQ	5.34959728	6.34311269	2.95913862	2.14356727	0.03206758	0.16796149	1.49393387	0.77479028
SURF6	2.77972661	5.69908621	2.6603633	2.14222103	0.0321757	0.1683161	1.492472	0.773874346
METAP2	3.17520114	6.15437223	2.87391192	2.14146167	0.03223683	0.16842427	1.49164773	0.773595326
PCBP1	51.8852734	3.62001251	1.69333069	2.137806	0.03253249	0.16958732	1.48768267	0.77060662
OSBP	3.55289321	6.33115655	2.96168652	2.13768625	0.03254222	0.16958732	1.48755287	0.77060662
FNBP1L	304.46673	3.47217058	1.62463428	2.13720135	0.03258162	0.16958732	1.48702735	0.77060662
SYVN1	5.84317281	5.61394996	2.628778	2.135574	0.03271415	0.16962448	1.48526433	0.770511466
CTBP1	25.6476511	4.55431794	2.13265589	2.13551467	0.03271899	0.16962448	1.48520007	0.770511466
USP34	152.282676	2.81212633	1.31708488	2.13511398	0.0327517	0.16962448	1.48476614	0.770511466
RIC8A	7.284701	6.0173494	2.82063809	2.13332913	0.03289774	0.17016916	1.48283399	0.769119147
SLC4A11	17.3364367	6.23904427	2.92579909	2.13242402	0.032972	0.17022883	1.48185465	0.768966892
UBE3A	13.698606	4.80694595	2.25446164	2.13219239	0.03299103	0.17022883	1.48160408	0.768966892
SEM1	2.90520519	6.03829962	2.83349182	2.13104537	0.0330854	0.17028119	1.48036355	0.768833312
SLC48A1	3.52531642	6.31035907	2.96180516	2.13057873	0.03312386	0.17028119	1.47985901	0.768833312
ZNF410	3.69885695	6.1182553	2.87392434	2.12888531	0.03326375	0.17066672	1.47802878	0.767851147
IFIT1	3.58884512	6.29710767	2.96188163	2.12604974	0.03349912	0.17157482	1.47496663	0.765546455
POLDIP3	14.764681	6.2274274	2.93179775	2.12409857	0.0336619	0.17219676	1.47286135	0.763975032
HAS2	3.45070161	6.28973496	2.96192447	2.12352983	0.03370948	0.17222855	1.47224797	0.763894854
CASP10	3.71033002	6.1246275	2.88583456	2.12230721	0.03381195	0.17252603	1.4709298	0.763145381
KCTD7	17.7300388	5.03029773	2.37165129	2.12101069	0.0339209	0.17252603	1.46953259	0.763145381
DMD	3.88132834	5.38663806	2.53979063	2.12089847	0.03393035	0.17252603	1.46941169	0.763145381
HDAC2	46.7751	5.03267165	2.37293709	2.12086181	0.03393343	0.17252603	1.4693722	0.763145381
TRAPPC1	2.93921211	6.05129375	2.85950662	2.11620204	0.03432763	0.17389324	1.46435621	0.759717305
RPS12	107.104386	6.11818243	2.89423083	2.11392345	0.03452181	0.1746644	1.4619065	0.75779561
IRF1	35.0751186	4.52641337	2.14224965	2.11292525	0.03460717	0.17473747	1.46083396	0.757613956
OR52A5	3.50839091	6.25826638	2.96210974	2.1127733	0.03462018	0.17473747	1.46067073	0.757613956
PNISR	147.083751	5.2470695	2.48524943	2.11128486	0.03474783	0.17495104	1.45907226	0.757083468
PABPC1	286.85121	3.67612335	1.74159018	2.11078553	0.03479075	0.17495104	1.4585362	0.757083468
PDE3A	12.1514101	5.22802811	2.47722167	2.11044016	0.03482046	0.17495104	1.4581655	0.757083468
CAMSAP1	4.23304421	4.83482792	2.29255208	2.10892828	0.03495077	0.17495104	1.45654322	0.757083468
ILKAP	3.37433848	6.24698977	2.9621771	2.10891839	0.03495163	0.17495104	1.45653261	0.757083468
PLEKHM1	116.431899	4.20285841	1.99294359	2.10886973	0.03495583	0.17495104	1.45648041	0.757083468
FAM49A	3.75589462	6.13398519	2.91012279	2.10780975	0.03504745	0.17498511	1.45534358	0.756998911
SREBF2	4.11958533	5.43049384	2.58264041	2.10269064	0.03549282	0.17699653	1.44985949	0.752035244
MEGF11	6.53213402	4.72623185	2.24942676	2.10108279	0.0356337	0.17748651	1.44813909	0.750834642
AZIN1	107.067544	4.84913136	2.30906572	2.10004043	0.03572529	0.17773009	1.4470243	0.750239035
DDX18	3.94490243	6.21320846	2.96090748	2.09841358	0.03586863	0.17808843	1.44528525	0.749364297
DDX54	7.71245747	6.19990514	2.95526516	2.0979184	0.03591235	0.17808843	1.44475612	0.749364297
APOL6	2.83237811	5.65377164	2.69538998	2.09757092	0.03594307	0.17808843	1.44438488	0.749364297
NPRL3	97.0946427	4.47144697	2.13250855	2.09680143	0.03601116	0.17808843	1.44356293	0.749364297

SCLY	3.63451256	6.08748865	2.90724864	2.09390025	0.03626887	0.17893787	1.44046601	0.747297739
UBE2L6	3.91000015	6.19354892	2.96100915	2.09170206	0.03646518	0.17948108	1.43812168	0.745981322
PDCL	3.231951	6.19518367	2.96249323	2.09120602	0.0365096	0.17948757	1.43759293	0.745965615
CNIH3	3.35138741	6.18736593	2.9625419	2.0885328	0.0367498	0.18034547	1.43474502	0.743894754
ST7L	24.4920835	5.33096499	2.55358447	2.08763997	0.03683032	0.18034547	1.43379446	0.743894754
MAP2K4	3.05041937	5.55484265	2.66120555	2.0873407	0.03685735	0.18034547	1.43347592	0.743894754
SLC35F6	5.56401955	6.1648693	2.95873612	2.08361579	0.03719513	0.18157154	1.42951392	0.740952231
APOBEC3C	27.5264921	5.7330857	2.75245677	2.08289764	0.03726056	0.18167793	1.42875068	0.74069783
TANGO2	128.001438	5.48006274	2.63167753	2.08234584	0.03731089	0.18171059	1.42816437	0.740619762
NNT	3.23040022	5.64208968	2.71165674	2.08067991	0.03746321	0.18223928	1.42639496	0.739358005
FBXL12	25.0969992	5.65108457	2.71685918	2.08000643	0.03752494	0.18232656	1.42567996	0.739150058
TCF3	3.71934727	5.27639354	2.53945366	2.07776721	0.03773081	0.18265317	1.42330392	0.738372792
GUCY1A2	28.62929	5.12168282	2.46602943	2.07689444	0.0378113	0.18265317	1.42237836	0.738372792
PTGES3	3.7216717	4.6219755	2.22557991	2.07675109	0.03782454	0.18265317	1.42222636	0.738372792
FANCM	10.3434663	4.25853302	2.050904	2.07641753	0.03785535	0.18265317	1.42187272	0.738372792
SLC25A37	559.723599	4.43743752	2.13837405	2.07514561	0.03797304	0.18300896	1.42052462	0.737527638
PSMB2	4.48652395	5.0240795	2.42429541	2.0723875	0.03822932	0.18339502	1.41760345	0.736612452
ANKRD17	12.7096562	4.58409057	2.21411974	2.07038964	0.03841587	0.18386631	1.41548931	0.735497846
VAV1	3.34503482	6.0623089	2.9293463	2.06950912	0.03849834	0.18404945	1.41455804	0.735065472
RPS14	143.908342	4.60045613	2.22382826	2.06871017	0.03857329	0.18419632	1.41371329	0.734719058
GMPS	39.2291337	5.00178861	2.41849775	2.06813863	0.03862699	0.18424145	1.41310914	0.734612664
CCNI	84.7596153	4.20066163	2.032196	2.06705536	0.03872894	0.18439797	1.41196441	0.734243858
NMI	2.88841647	5.47031953	2.64669514	2.06684912	0.03874837	0.18439797	1.41174653	0.734243858
PNRC1	3.07169144	6.11832895	2.96298303	2.06492203	0.03893038	0.18491968	1.40971138	0.733016869
NCOA2	387.48398	3.73944575	1.81446349	2.06090989	0.03931164	0.18633084	1.40547887	0.729715247
CKAP2	3.07331356	6.10405041	2.96307686	2.06003783	0.03939492	0.18640962	1.40455972	0.729531675
SZRD1	3.37228479	5.97754082	2.90971591	2.0543383	0.03994296	0.18763422	1.39855975	0.72668796
PRKAG2	4.38858745	6.0809743	2.96026791	2.05419728	0.0399566	0.18763422	1.39841146	0.72668796
GNL2	4.37700586	5.29508905	2.57776114	2.05414263	0.03996189	0.18763422	1.39835399	0.72668796
LAMTOR4	3.00314335	6.08659818	2.96319278	2.05406757	0.03996915	0.18763422	1.39827506	0.72668796
EIF1B	98.4736892	3.54095642	1.72568777	2.05191024	0.04017839	0.18837637	1.39600751	0.724973582
FBXO8	2.98705069	6.07725241	2.96325541	2.05087027	0.04027958	0.18837637	1.39491504	0.724973582
SHOC2	3.59586889	6.07372838	2.96165893	2.0507859	0.0402878	0.18837637	1.39482643	0.724973582
SCLT1	6.87984501	4.29403692	2.09406319	2.05057657	0.0403082	0.18837637	1.3946066	0.724973582
ZNF562	32.8267444	3.96701485	1.93726427	2.04774067	0.04058542	0.18913731	1.39162999	0.723222791
SLC16A7	3.00857111	5.54653184	2.70897425	2.04746569	0.04061238	0.18913731	1.39134153	0.723222791
MED12	232.484658	4.18739156	2.04556894	2.04705472	0.04065271	0.18913731	1.39091048	0.723222791
SNCA	268.672676	4.18458919	2.04812055	2.04313618	0.04103896	0.19050861	1.38680366	0.720085384
ALAD	4.83708389	5.65973082	2.77250941	2.04137479	0.04121359	0.19089364	1.38495957	0.71920854
USP10	6.34537817	4.90389066	2.40280649	2.0409012	0.04126065	0.19089907	1.38446395	0.719196198
CRYM	17.6117345	4.40541971	2.15905303	2.04044072	0.04130645	0.19089907	1.38398213	0.719196198
ARNTL	9.77393488	4.90915211	2.40756507	2.03905272	0.04144477	0.19107274	1.38253031	0.718801268
ZHX1	2.9005396	6.04122872	2.96350056	2.03854482	0.04149548	0.19107274	1.38199924	0.718801268
CNOT6L	3.69735133	4.53074017	2.22297137	2.03814598	0.04153534	0.19107274	1.38158228	0.718801268
USP25	2.90925107	6.03851194	2.96351929	2.03761519	0.04158843	0.19107274	1.38102746	0.718801268
ALKBH5	15.7382647	5.20084081	2.55365838	2.03662355	0.04168778	0.19107274	1.37999122	0.718801268
TSFM	2.92711106	6.03553242	2.96353987	2.03659565	0.04169058	0.19107274	1.37996208	0.718801268
GTF3C2	4.20477916	6.02401223	2.96054107	2.03476733	0.04187429	0.19160939	1.37805254	0.717583215
RNF167	8.88426694	4.16631572	2.04817275	2.03416227	0.04193524	0.19167764	1.37742089	0.71742855
PCGF3	3.61853764	5.2172515	2.56783035	2.03177421	0.04217652	0.19256911	1.37492927	0.715413387
MAPKAPK3	10.6524937	5.96754515	2.93912045	2.03038468	0.04231745	0.19278979	1.37348049	0.71491596
TSPYL2	10.1080245	5.04640278	2.48682964	2.0292515	0.04243268	0.19283017	1.37229953	0.714825016
TMEM216	2.85395495	6.01341959	2.96369392	2.02902856	0.04245538	0.19283017	1.37206725	0.714825016
COX20	2.98319463	6.01239088	2.96370114	2.02867651	0.04249125	0.19283017	1.37170049	0.714825016
TNIP3	2.86931078	6.0111711	2.96370971	2.02825907	0.04253381	0.19283017	1.37126567	0.714825016
UBAP1	18.7811796	4.8582541	2.39556116	2.02802341	0.04255786	0.19283017	1.37102023	0.714825016
UROD	498.049029	5.33150947	2.63206945	2.02559605	0.0428062	0.19374457	1.36849335	0.712770461
GMPR	639.499353	3.56734001	1.76291046	2.02355144	0.04301633	0.19429125	1.36636669	0.71154675
ATL3	6.75577686	5.3899616	2.66366563	2.02351284	0.0430203	0.19429125	1.36632655	0.71154675
STOM	932.444915	3.2305754	1.59782631	2.02185644	0.04319119	0.19485167	1.36460488	0.71029586
SLC35D3	4.04042798	5.96909582	2.95447084	2.02036038	0.04334602	0.19533856	1.36305077	0.70921202
UQCRFS1	2.79603002	5.98401034	2.96390237	2.01896338	0.04349103	0.19556872	1.36160035	0.708700612

RAB13	2.79603002	5.98401034	2.96390237	2.01896338	0.04349103	0.19556872	1.36160035	0.708700612
SRSF8	149.910184	4.93663112	2.4467883	2.01759634	0.04363332	0.19578571	1.36018175	0.708219008
DENND1A	9.42120168	4.58284086	2.2730886	2.01612945	0.04378644	0.19626129	1.35866035	0.707165343
FBN1	4.01770221	3.66059456	1.81643687	2.01526109	0.0438773	0.1962873	1.35776011	0.707107806
SSBP3	37.882683	4.22012671	2.09417584	2.01517304	0.04388652	0.1962873	1.35766885	0.707107806
AHR	6.70448681	4.86736379	2.41844158	2.01260342	0.04415637	0.19692691	1.35500667	0.705694929
RAB27A	2.99985958	4.95951244	2.46440226	2.0124606	0.04417141	0.19692691	1.35485877	0.705694929
CSRNP2	2.74064938	5.96301813	2.9640537	2.01177803	0.04424334	0.19703665	1.35415209	0.705452981
TSPAN6	2.74732498	5.9587761	2.96408454	2.01032596	0.0443967	0.1975084	1.3526493	0.704414424
LLPH	3.50563205	5.4951907	2.73586349	2.00857635	0.04458208	0.19809596	1.35083966	0.703124379
IMPDH2	3.51054943	5.49061316	2.73473047	2.00773466	0.0446715	0.19809596	1.34996951	0.703124379
STX17	4.05590168	3.51862952	1.75428631	2.00573276	0.04488477	0.19863167	1.34790102	0.701951506
HYOU1	3.66166705	5.82748682	2.90637777	2.0050686	0.04495571	0.19863167	1.34721511	0.701951506
KIAA1462	4.17780683	5.12428516	2.55580571	2.00495881	0.04496745	0.19863167	1.34710173	0.701951506
ELP4	14.0244198	5.11775512	2.55616633	2.00212133	0.04527168	0.19969417	1.34417335	0.69963462
GIT2	58.4087694	5.29393415	2.65129689	1.99673382	0.04585411	0.20141776	1.33862176	0.695902248
TONSL	3.37111133	5.12336273	2.56589306	1.99671717	0.04585592	0.20141776	1.33860463	0.695902248
CD9	2.92710097	5.7714729	2.89213406	1.99557586	0.04598011	0.20175045	1.33743	0.69518549
SCYL2	18.090822	4.93456294	2.47409008	1.99449607	0.04609787	0.20205424	1.33631915	0.694532036
AK6	58.402202	5.41257179	2.71649232	1.99248558	0.04631781	0.20280478	1.33425203	0.692921824
MORC3	16.1028102	4.15734399	2.08755033	1.99149402	0.0464266	0.20306762	1.3332331	0.692359329
RPL7	141.799085	3.27866172	1.64915062	1.98809113	0.04680161	0.20449309	1.32973918	0.689321364
OPTN	81.1397685	4.38811086	2.21167161	1.9840698	0.04724806	0.20579594	1.32561603	0.686563197
ILF2	3.59237701	5.79748711	2.92431221	1.98251305	0.04742185	0.20633707	1.32402152	0.685422741
HDX	5.5417869	5.8619474	2.95762532	1.98197769	0.04748174	0.206382	1.3234734	0.685328185
STRAP	22.4207978	4.73247364	2.38906627	1.9808884	0.04760379	0.20669675	1.32235846	0.68466635
PRKACB	54.2007994	5.29200116	2.67348444	1.97943967	0.04776653	0.20705143	1.32087634	0.683921771
ZC3H15	9.81732541	5.23460971	2.64470913	1.97927615	0.04778492	0.20705143	1.3207091	0.683921771
TCEA1	27.9572565	5.55385863	2.81262126	1.97462016	0.04831127	0.20868063	1.31595155	0.680517861
POC1B	33.7197934	3.51891463	1.78348109	1.97305969	0.04848876	0.20923027	1.3143589	0.679375491
DNHD1	2.74909221	4.67421741	2.37284272	1.96988084	0.04885203	0.20979261	1.3111174	0.678209816
CLEC16A	76.8209743	5.08406242	2.58147711	1.96943928	0.04890267	0.20979261	1.31066744	0.678209816
HMGCL	3.67726643	5.83209628	2.96154303	1.96927623	0.04892138	0.20979261	1.31050131	0.678209816
RPL38	92.4840374	4.77894139	2.43485124	1.96272418	0.04967823	0.21234946	1.30383387	0.672948835
RBM25	26.1805268	3.69437392	1.88276781	1.96220368	0.04973878	0.21234946	1.30330491	0.672948835
MBTPS1	2.71715935	5.67682027	2.89351247	1.96191319	0.04977259	0.21234946	1.30300974	0.672948835
PAPD7	3.85726754	3.93930837	2.01204982	1.95785827	0.05024665	0.21415254	1.29889292	0.669276766
EEF1D	3.33814266	5.68947713	2.90776488	1.95664965	0.05038867	0.21434819	1.29766708	0.668880176
NCOA4	548.903658	4.13988234	2.11613861	1.95633798	0.05042535	0.21434819	1.29735106	0.668880176
ZPR1	40.5090046	4.18750304	2.14183167	1.9551037	0.05057083	0.21465539	1.29609991	0.668258195
GRK5	5.25582224	5.77888632	2.95794578	1.9536823	0.0507388	0.21514905	1.2946598	0.667260567
MRPL32	23.770366	5.35879709	2.74470411	1.95241341	0.05088914	0.21534796	1.29337487	0.666859243
HIST1H3I	4.20238197	5.07753836	2.60267128	1.95089499	0.05106954	0.21582869	1.29183807	0.66589082
MRPL1	9.33348852	4.83253026	2.47747716	1.95058519	0.05110641	0.21582869	1.29152463	0.66589082
DPYD	30.2041034	4.8304333	2.4780516	1.94928681	0.05126118	0.21626298	1.29021138	0.665017807
SSPN	5.30550447	5.46666366	2.80707599	1.94745838	0.05147981	0.21696549	1.28836311	0.66360933
IDH2	16.6295626	5.36450557	2.75597761	1.94649824	0.05159492	0.21720018	1.28739307	0.663139822
AUH	4.2147984	5.76014929	2.96046591	1.94569013	0.05169197	0.21720018	1.28657689	0.663139822
BAG4	32.3338867	5.40755103	2.78045671	1.94484274	0.05179391	0.2173203	1.28572131	0.662899699
ARMC8	283.071201	4.08681128	2.10163671	1.94458503	0.05182494	0.2173203	1.28546117	0.662899699
CACUL1	13.9541925	4.31098324	2.22127096	1.94077325	0.05228579	0.21859239	1.28161635	0.660364964
TTC14	3.50137205	5.74387859	2.96204912	1.93915711	0.05248221	0.21899881	1.27998788	0.659558252
RPS15A	146.704001	3.59274392	1.85319443	1.93867619	0.05254078	0.21899881	1.27950348	0.659558252
PIK3R1	28.0706055	4.43597274	2.29068015	1.93653084	0.05280272	0.21956672	1.27734371	0.658433495
RPL35A	142.149511	4.20744496	2.17283835	1.93638195	0.05282094	0.21956672	1.27719388	0.658433495
DOCK8	2.96174947	4.93867827	2.5522178	1.93505361	0.05298372	0.21993365	1.27585758	0.657708321
TRHDE	2.85827245	5.18022967	2.67755928	1.93468347	0.05302915	0.21993365	1.27548533	0.657708321
C19orf44	4.92401337	5.39323785	2.79105339	1.93233059	0.05331872	0.22074619	1.27312032	0.656106782
GP5	6.16730379	4.65311406	2.40815712	1.93223026	0.05333109	0.22074619	1.27301952	0.656106782
TBC1D31	2.8570789	5.72068918	2.96390964	1.93011592	0.05359248	0.2211433	1.27089618	0.655326211
RNASEH2B	46.9732754	4.54743238	2.35605109	1.93010771	0.05359349	0.2211433	1.27088793	0.655326211
AES	7.38615852	5.11379764	2.6509057	1.92907565	0.05372147	0.2211433	1.2698521	0.655326211

ZPBP	5.84130129	4.55471778	2.36250558	1.92791831	0.05386529	0.22138998	1.26869102	0.654842046
AP2A1	35.2776396	3.65188922	1.89486231	1.92725835	0.05394744	0.22138998	1.26802915	0.654842046
FLCN	457.565858	4.75415037	2.46882464	1.92567357	0.05414515	0.22178114	1.26644047	0.654075388
TERF2IP	156.906743	4.57277352	2.37485162	1.92549863	0.05416701	0.22178114	1.26626516	0.654075388
ACER3	9.44895184	4.38556358	2.28037066	1.92318014	0.05445743	0.22275122	1.26394285	0.652179912
PSMF1	78.1483404	5.32596461	2.77191643	1.92140158	0.0546811	0.22331608	1.26216276	0.651080009
EIF3A	300.887715	4.91870216	2.56018464	1.92122946	0.05470279	0.22331608	1.26199056	0.651080009
UCP2	98.2292017	5.29281631	2.75714168	1.91967513	0.05489895	0.22389737	1.26043598	0.649951005
HIPK1	238.513773	3.7419133	1.95018306	1.91874977	0.05501601	0.22415525	1.25951092	0.64945109
YBX3	144.875667	2.82200531	1.47190106	1.91725204	0.05520592	0.22458283	1.25801435	0.648623447
TMEM11	174.559796	5.54229241	2.89101988	1.9170717	0.05522882	0.22458283	1.25783421	0.648623447
CEP104	3.56647464	5.20299105	2.7152542	1.91620772	0.05533866	0.22480994	1.25697135	0.648184495
VPS35	81.4332107	5.21385187	2.72359259	1.91432885	0.05557815	0.22556279	1.25509591	0.646732548
GCDH	2.88235689	5.20389711	2.71963139	1.91345677	0.0556896	0.22575847	1.25422588	0.646355945
ASCC2	21.5979265	3.85478778	2.01584716	1.91224209	0.05584515	0.22589245	1.25301454	0.646098278
MYEOV	19.1362667	3.40943845	1.78318042	1.91199859	0.05587638	0.22589245	1.25277177	0.646098278
MCC	7.20692691	3.3689212	1.76365622	1.91019154	0.05610856	0.22661108	1.25097091	0.644718855
CR1	16.6490436	4.82000489	2.52570341	1.90838119	0.05634197	0.22733328	1.249168	0.643336981
SERF2	67.4998021	3.32290024	1.74259905	1.90686448	0.05653814	0.22790398	1.24765849	0.642248093
RSL1D1	14.2874227	5.55822957	2.91743304	1.90517811	0.05675692	0.22856463	1.24598116	0.640990978
CALD1	46.5569022	4.40513026	2.31438964	1.90336587	0.05699282	0.22905123	1.24417984	0.640067374
CISD2	62.4207444	4.57176336	2.40241958	1.9029829	0.05704278	0.22905123	1.24379934	0.640067374
ATP1A1	38.3737967	4.16627486	2.19070984	1.90179219	0.05719833	0.22945456	1.24261667	0.639303301
TRIM38	6.10824655	4.0630383	2.1419894	1.89685267	0.05784739	0.23161203	1.23771626	0.635238882
FAM20B	10.2929162	5.03261854	2.65504804	1.89549058	0.05802744	0.231887	1.2363666	0.634723596
CDH23	11.2851363	4.30023104	2.26922846	1.895019	0.05808988	0.23191398	1.23589949	0.634673073
KRI1	9.01448595	5.03774513	2.66147054	1.89284272	0.05837879	0.23243685	1.23374492	0.633695024
ZSCAN25	3.80956208	5.59944128	2.96129597	1.89087526	0.058641	0.23291504	1.23179865	0.632802464
SCAMP3	3.56098778	5.16789437	2.73345146	1.89061135	0.05867624	0.23291504	1.2315377	0.632802464
NOP2	3.67933448	5.57206904	2.96150953	1.88149624	0.05990445	0.23666235	1.22254094	0.625870829
PIGQ	3.3149564	5.10282966	2.71680508	1.87824651	0.06034745	0.23771728	1.21934107	0.623939253
PMS1	7.49123777	4.53180553	2.41389023	1.87738675	0.06046511	0.23774948	1.21849518	0.62388042
KIAA0430	479.212353	4.42798245	2.35998743	1.87627374	0.0606177	0.23812463	1.21740054	0.623195686
TLCD2	3.93245371	3.71409766	1.98172143	1.87417747	0.06090597	0.23903153	1.21534015	0.621544814
PPIE	12.6399116	3.91455753	2.09120273	1.8719168	0.06121812	0.23957917	1.21312005	0.620550953
ANGPT2	3.47928228	5.47484081	2.92587729	1.87117923	0.06132024	0.23975067	1.21239613	0.620240162
NSD2	240.232163	3.77630826	2.01858598	1.87076909	0.06137709	0.23975067	1.21199368	0.620240162
HBS1L	11.2498806	3.81198737	2.04302771	1.86585202	0.06206207	0.241746	1.20717373	0.616640711
SLC43A1	5.39674044	5.51085306	2.9555329	1.86458863	0.06223909	0.24220893	1.20593678	0.615809842
FBXO7	243.660321	4.4107023	2.36871763	1.86206335	0.06259416	0.24336329	1.20346618	0.613744928
FAR1	12.2148097	5.34607944	2.87607632	1.85881	0.06305407	0.24446663	1.20028685	0.611780407
ATXN7	42.9013552	3.90013646	2.09953558	1.85761865	0.06322319	0.24489428	1.19912361	0.611021356
RPS29	26.9070579	3.81366781	2.05360194	1.85706281	0.06330222	0.24497252	1.19858107	0.610882625
CACNB4	3.48234144	5.47614617	2.95203263	1.85504256	0.06359015	0.24584959	1.19661016	0.609330511
STAT6	85.5428659	2.71231131	1.46276807	1.85423196	0.06370598	0.24584959	1.19581979	0.609330511
TCEANC	3.02463463	5.0633403	2.73228558	1.85315194	0.06386058	0.24618971	1.19476711	0.608730095
TKFC	6.87122875	5.27931132	2.85080081	1.85186959	0.06404455	0.24618971	1.19351781	0.608730095
MACROD2	3.15514688	5.04101139	2.7221653	1.85183883	0.06404897	0.24618971	1.19348785	0.608730095
UBA52	66.7330308	3.76969397	2.03629283	1.85125338	0.06413311	0.24618971	1.1929177	0.608730095
NDUFB3	32.6678527	5.28125078	2.85481602	1.84994435	0.06432157	0.24662525	1.19164336	0.607962466
RBBP4	77.4884093	4.03834235	2.18561088	1.84769502	0.06464647	0.24741537	1.18945516	0.60657333
WDFY1	3.7732631	5.08700525	2.7628574	1.84121166	0.06559055	0.25056793	1.18315874	0.601074522
ATP6V1E1	26.7271256	5.33584771	2.90227364	1.83850607	0.06598787	0.25170187	1.1805359	0.599113549
UBE2I	68.8623064	4.44321238	2.4194575	1.83644985	0.06629115	0.25254928	1.17854443	0.597653873
HNRNPU	340.072159	2.95890665	1.61450191	1.83270557	0.06684637	0.254037	1.17492218	0.595103019
ILF3	80.7130949	4.20157823	2.29310897	1.83226278	0.06691228	0.254037	1.17449418	0.595103019
EIF4G1	413.156936	3.07267662	1.67711941	1.83211559	0.0669342	0.254037	1.17435191	0.595103019
ATG9A	433.185808	3.53053278	1.92732843	1.83182727	0.06697716	0.254037	1.17407328	0.595103019
FARP2	2.80484667	4.06084509	2.21690501	1.83176324	0.0669867	0.254037	1.1740114	0.595103019
IKZF4	5.85548238	5.40377039	2.9513001	1.83097964	0.06710358	0.25424869	1.1732543	0.594741269
KLHDC2	53.1283179	4.9028211	2.67978905	1.82955486	0.06731653	0.25459221	1.17187829	0.594154889
NUP62CL	25.9783613	4.70138346	2.57226476	1.82772144	0.06759137	0.25539969	1.17010876	0.592779634

SLF1	3.82727384	4.62386634	2.53110981	1.8268138	0.06772777	0.25568308	1.16923323	0.592298018
FASN	2.99357147	3.41585145	1.87168998	1.82500921	0.06799964	0.2564769	1.16749339	0.590951744
NFIX	31.9671151	3.76986174	2.06864255	1.82238431	0.06839669	0.25750798	1.1649649	0.589209303
ASF1A	2.7286844	5.40077701	2.96433037	1.82192142	0.06846691	0.25753949	1.16451927	0.589156175
B9D2	3.54956674	5.35107591	2.9395004	1.82040319	0.06869763	0.25817411	1.16305827	0.588087306
ZNF638	153.196969	3.26300388	1.79330467	1.81954797	0.06882787	0.25843035	1.16223566	0.587656489
FZD5	155.66821	2.87434772	1.58119104	1.81783709	0.06908904	0.25909445	1.16059087	0.586541894
MTRNR2L6	3.9289906	3.48765988	1.91980504	1.81667399	0.06926705	0.25913749	1.15947333	0.586469758
NCL	102.163626	3.69196323	2.03270552	1.81628042	0.06932737	0.25913749	1.15909529	0.586469758
DNAH1	15.7802527	3.10769604	1.71264787	1.81455634	0.06959212	0.25966091	1.15743995	0.585593424
SLC7A5	381.817805	3.24917687	1.794111	1.81102333	0.07013724	0.26146058	1.15405132	0.582593775
FAM222B	51.1663306	5.23902076	2.89538238	1.80944002	0.07038267	0.26214082	1.15253426	0.581465342
BEX3	5.3297757	5.10575197	2.82249221	1.80895166	0.07045851	0.26218109	1.15206652	0.581398636
TUBD1	3.14995129	5.32826463	2.94613673	1.80855986	0.07051941	0.26218109	1.15169133	0.581398636
COX7B	46.8442528	5.1827518	2.86773665	1.80726212	0.07072142	0.26246861	1.15044903	0.580922632
CANX	3.79104857	4.4044715	2.43710815	1.8072532	0.07072281	0.26246861	1.1504405	0.580922632
EAF2	31.2348832	4.38730569	2.43234676	1.80373365	0.07127307	0.26367375	1.14707452	0.578933111
RPL23A	24.2979391	4.45430637	2.46973456	1.80355672	0.07130083	0.26367375	1.14690544	0.578933111
RAB5A	27.3345822	4.74364191	2.63521525	1.80009656	0.07184539	0.26498159	1.14360108	0.576784299
GLS	85.3638992	4.10369681	2.28595132	1.79518119	0.07262484	0.26662048	1.13891483	0.574106502
ZCCHC6	254.816305	2.77530642	1.54616749	1.79495846	0.07266032	0.26662048	1.1387027	0.574106502
SLC22A17	3.07555021	4.92447776	2.74363569	1.79487305	0.07267393	0.26662048	1.13862136	0.574106502
NPL	23.2712801	4.76652435	2.65917652	1.79248136	0.07305589	0.26755034	1.13634475	0.572594494
FAM193A	3.99428425	4.04660503	2.26265991	1.7884283	0.07370693	0.26946064	1.13249166	0.569504661
LRMP	42.3357401	4.70309086	2.63303568	1.78618577	0.07406918	0.27031033	1.13036243	0.568137358
PLEKHA5	79.502707	3.48394731	1.95647058	1.78073075	0.07495645	0.2733088	1.125191	0.563346386
AURKAIP1	2.92234382	5.2263883	2.93880578	1.77840548	0.07533728	0.27445708	1.12299005	0.561525556
RNASEH1	75.545	4.07601514	2.29273028	1.77779967	0.07543676	0.27452333	1.12241696	0.561420736
AKAP11	4.99746459	4.97791225	2.80440229	1.77503501	0.07589211	0.27575456	1.11980339	0.55947729
MED4	56.740295	5.0345218	2.84172138	1.77164512	0.07645349	0.27755215	1.11660271	0.556655408
RELB	6.58154734	4.72653676	2.67368311	1.76779991	0.07709436	0.27939157	1.11297739	0.553786703
MTA3	6.95646757	4.9669374	2.8136272	1.76531468	0.07751089	0.28016959	1.11063725	0.55257901
SZT2	59.4957157	3.85888605	2.18837326	1.76335825	0.07784009	0.28076494	1.10879669	0.551657131
TCP11L2	8.36882558	3.62695382	2.057254	1.7630073	0.07789926	0.28076494	1.10846668	0.551657131
REC8	17.2362311	4.2827996	2.42978123	1.76262766	0.07796331	0.28076494	1.10810974	0.551657131
LRRC9	3.23398582	4.77812211	2.71124529	1.76233487	0.07801274	0.28076494	1.10783449	0.551657131
ATP5B	232.881162	5.05691353	2.87663328	1.75792777	0.0787598	0.28247701	1.1036954	0.549016895
PCMT1	54.7929658	4.85794606	2.76546935	1.75664433	0.07897845	0.28301745	1.10249139	0.548186793
ANK1	408.707671	2.36437394	1.34731676	1.75487607	0.07928051	0.28380305	1.10083357	0.546982946
URB1	2.73896805	5.10749673	2.9109785	1.75456354	0.07933399	0.28380305	1.10054069	0.546982946
NKD2	3.36762454	4.78920772	2.73019551	1.75416292	0.0794026	0.28380464	1.1001653	0.546980506
TMEM266	8.62014782	3.83466248	2.1895034	1.75138458	0.07987969	0.28515606	1.09756364	0.544917396
HEATR9	30.4607213	4.85914577	2.77787961	1.74922835	0.08025156	0.28585755	1.09554654	0.543850333
PAFAH1B2	245.961252	3.69021772	2.11118845	1.74793383	0.08047549	0.28641019	1.09433639	0.543011536
FAM83D	4.36712799	4.53760841	2.59757759	1.74686155	0.08066136	0.28658182	1.09333448	0.542751367
USP32	170.020403	3.84514406	2.20200628	1.74620031	0.08077615	0.286745	1.09271685	0.54250414
ETV1	16.8990303	4.52394722	2.59716105	1.74188166	0.08152915	0.28917154	1.08868707	0.538844445
AKT2	2.9179206	4.03482869	2.32063865	1.73867168	0.08209253	0.29067457	1.08569636	0.536592957
WDR48	5.63469353	4.86038404	2.79645074	1.73805459	0.0822012	0.29081205	1.08512187	0.536387608
TEP1	4.51217522	4.53623534	2.61932198	1.73183571	0.08330282	0.29346272	1.07934031	0.532447065
MYL12B	157.445411	3.78737273	2.18857378	1.73052092	0.08353725	0.29379907	1.07811985	0.531949584
KLHL5	22.4310079	3.99198301	2.30682617	1.73050881	0.08353941	0.29379907	1.0781086	0.531949584
FBXW2	4.57319381	4.14629044	2.39793222	1.72911077	0.08378927	0.29418088	1.07681159	0.531385555
ARL6IP1	499.75653	4.65300367	2.69515988	1.72642956	0.08427016	0.29512276	1.07432617	0.529997303
SMARCE1	24.5963232	4.06345736	2.35523082	1.7252905	0.08447513	0.29527042	1.07327112	0.529780052
INSL6	7.35649669	4.80507087	2.78566647	1.72492684	0.08454066	0.29527042	1.07293438	0.529780052
TMED10	78.1171902	4.80975563	2.78887843	1.72462004	0.08459597	0.29527042	1.07265033	0.529780052
SEPSECS	9.59701634	5.02612887	2.91997359	1.72129258	0.08519775	0.29691596	1.0695719	0.527366454
TBPL1	112.44666	4.04550579	2.3521339	1.71993006	0.08544516	0.29748631	1.06831255	0.526533013
TSG101	282.600125	4.24239009	2.46825581	1.71878056	0.08565434	0.29772878	1.06725064	0.526179179
NR1D2	30.1232142	4.98034429	2.89840225	1.7183068	0.08574067	0.29776827	1.06681313	0.526121589
PLEC	138.554335	3.31170084	1.93416837	1.71220918	0.08685813	0.3008962	1.06118954	0.52158329

USP15	1139.48538	2.10927437	1.23309131	1.71055813	0.08716271	0.30115376	1.05966926	0.521211704
MFSD1	16.5834729	3.62132259	2.11717609	1.71044941	0.0871828	0.30115376	1.05956918	0.521211704
HNRNPH2	4.95559612	5.02716486	2.94881448	1.70480879	0.08823009	0.30387594	1.05438327	0.517303688
ELOB	15.1614334	4.12566244	2.42230608	1.70319617	0.08853136	0.30437093	1.05290285	0.516596834
TMEM214	9.35011248	4.30810465	2.52990586	1.70287152	0.08859211	0.30437093	1.05260494	0.516596834
NOP58	17.0631677	3.81407701	2.2412558	1.7017589	0.08880057	0.30473567	1.05158423	0.516076701
SDF4	5.76713765	4.76800987	2.80348427	1.70074429	0.08899101	0.30473567	1.05065385	0.516076701
GTF3A	6.18483636	3.12310727	1.83844192	1.6987794	0.08936076	0.30549934	1.04885316	0.51498973
DDX10	2.9781289	4.23805239	2.49737638	1.69700187	0.08969631	0.30614379	1.04722544	0.514074547
TMF1	77.3002553	3.70516579	2.18507143	1.69567262	0.0899479	0.30650003	1.04600899	0.513569473
MED17	11.5629695	4.00080727	2.35997766	1.69527337	0.09002357	0.30650708	1.04564375	0.513559483
PTPRA	34.3720957	3.59856621	2.12583621	1.6927768	0.09049796	0.30748458	1.04336121	0.512176656
ZBTB49	14.0084374	4.25258665	2.51361331	1.69182214	0.09067989	0.30748458	1.04248901	0.512176656
CHCHD2	23.4280116	4.90292123	2.90062486	1.69029829	0.0909709	0.30822037	1.04109749	0.511138656
PUS10	4.05427547	4.66219835	2.76137459	1.68836143	0.09134187	0.30897445	1.0393301	0.510077435
USPL1	26.988287	3.96235448	2.35229499	1.68446326	0.09209217	0.31100715	1.03577727	0.507229629
CDC37L1	37.7391376	3.47984639	2.07335502	1.67836495	0.09327588	0.31395672	1.03023066	0.50313021
RPS3A	20.9324999	4.09356052	2.43956855	1.67798544	0.09334994	0.31395672	1.02988595	0.50313021
NOL11	3.46291664	4.29708708	2.56127803	1.67771208	0.09340332	0.31395672	1.02963768	0.50313021
GATC	3.43694855	4.35086719	2.5934495	1.67763713	0.09341796	0.31395672	1.02956962	0.50313021
PELI2	43.9300326	3.81985652	2.27902694	1.67609099	0.09372037	0.31421226	1.028166	0.502776875
BICD1	96.3490019	4.67611025	2.79114865	1.67533544	0.09386844	0.31426008	1.02748042	0.50271078
LETM1	41.3612496	4.23262507	2.52910867	1.67356394	0.09421633	0.31511389	1.02587382	0.501532449
RDX	57.7508566	3.82220124	2.28841646	1.67023849	0.09487219	0.31679855	1.02286108	0.499216816
ZFP36L2	6.18704351	3.7830615	2.26632164	1.66925181	0.09506749	0.31719633	1.02196799	0.498671852
PIK3CA	50.4334873	3.74358297	2.2438438	1.66837948	0.09524042	0.3173776	1.02117871	0.498423725
PKN2	37.9231523	3.20854386	1.92349336	1.66808159	0.09529953	0.3173776	1.02090923	0.498423725
COX6A1	10.7773927	4.85602047	2.91388784	1.66650906	0.09561207	0.31799412	1.0194873	0.497580906
HCFC1	45.6704996	3.29037943	1.97516217	1.66587812	0.09573769	0.31815783	1.01891704	0.497357391
UBE2D3	233.023111	2.53203241	1.52509901	1.66024133	0.09686591	0.32087621	1.01382903	0.493662477
YARS	2.90351466	4.9068608	2.96266535	1.65623188	0.09767487	0.32304857	1.01021716	0.490732171
ZNF595	4.79171603	3.6695573	2.22017783	1.65282134	0.09836723	0.32430811	1.00714956	0.489042189
FAM149B1	62.5516664	3.0950188	1.87618713	1.64963225	0.09901818	0.32619595	1.00428508	0.486521434
THRAP3	255.506026	3.82481673	2.32159063	1.64749835	0.09945566	0.32711955	1.00237051	0.485293499
PPIG	78.2726799	3.43639797	2.08870032	1.64523265	0.09992184	0.3276686	1.00033957	0.48456518
FHL1	3.57476964	4.86688085	2.95830705	1.64515744	0.09993735	0.3276686	1.00027218	0.48456518
EIF1AY	54.7666161	4.47538326	2.72616468	1.64164083	0.10066446	0.32945095	0.99712383	0.482209239
SULF2	3.42394061	2.92481746	1.78192457	1.64138118	0.10071831	0.32945095	0.99689156	0.482209239
ASAH1	274.154816	3.2059042	1.95498544	1.63986091	0.10103409	0.33022445	0.99553206	0.481190771
RIC3	8.77628869	4.7818472	2.91828515	1.63858121	0.10130051	0.3307906	0.99438837	0.480446843
HERC4	19.2688116	3.41584031	2.08503382	1.63826614	0.10136619	0.3307906	0.99410689	0.480446843
CCNDBP1	142.465979	3.19567408	1.95253336	1.63668091	0.10169716	0.33161079	0.99269118	0.479371352
PRDX5	6.94811666	4.78256911	2.92603103	1.63449022	0.10215596	0.33255848	0.9907363	0.47813197
SYK	7.51116609	4.76968095	2.92295079	1.63180337	0.10272091	0.33390184	0.98834112	0.476381185
BTBD19	9.01447034	4.29544762	2.63294243	1.63142482	0.10280071	0.33390184	0.98800388	0.476381185
AP3D1	59.2166925	3.06568889	1.88177918	1.62914381	0.10328258	0.33478222	0.98597293	0.475237616
YWHAG	8.51349438	4.17137463	2.56074683	1.628968	0.10331979	0.33478222	0.98581647	0.475237616
RRBP1	11.7798485	4.11661125	2.5276674	1.62862062	0.10339336	0.33478222	0.98550737	0.475237616
LTV1	3.77074112	4.17302021	2.57050832	1.62342217	0.10449919	0.33724743	0.98088709	0.472051351
IRAK1	4.773361	4.77148971	2.94228193	1.62169698	0.10486825	0.33724743	0.97935599	0.472051351
MKLN1	271.587788	3.09246001	1.90743635	1.62126511	0.10496079	0.33724743	0.97897289	0.472051351
MAPK1	75.1986325	4.00435739	2.47170485	1.62007911	0.10521529	0.33734932	0.97792116	0.471920159
ZFAND6	13.5468846	3.95465286	2.44119355	1.61996695	0.10523938	0.33734932	0.97782173	0.471920159
CDK7	2.82329603	4.09908151	2.53561214	1.61660431	0.10596372	0.33866888	0.9748428	0.470224706
ZNF706	3.36739289	4.15154331	2.56815692	1.61654581	0.10597636	0.33866888	0.97479101	0.470224706
NCAPD3	10.698989	4.69595791	2.90732541	1.61521579	0.10626397	0.33932759	0.97361396	0.469380826
PHLPP2	5.46293397	3.71744568	2.30337005	1.61391596	0.10654566	0.33996637	0.97246425	0.46856404
SRP9	5.41646058	3.97885712	2.46969446	1.61107262	0.1071639	0.34167723	0.9699515	0.466383961
HGF	35.7853808	3.98740233	2.48031423	1.60761982	0.10791847	0.34381982	0.96690423	0.463669096
PAG1	16.9570757	4.6464836	2.90288087	1.60064564	0.10945543	0.34844985	0.9607627	0.45785972
GNB5	5.07064369	4.51787038	2.82659885	1.59834155	0.10996699	0.34897083	0.95873768	0.457210869
MSH6	16.8705947	2.43713296	1.52497633	1.59814478	0.11001076	0.34897083	0.95856483	0.457210869

ARIH1	120.527741	3.33660671	2.08975315	1.59665112	0.1103435	0.34907903	0.95725326	0.457076238
CLU	53.4755032	3.51278422	2.2019562	1.59530159	0.11064481	0.34956373	0.95606895	0.456473639
MGLL	2.91632707	3.58660554	2.24981815	1.59417575	0.11089668	0.34980935	0.95508147	0.45616859
STK24	12.8790783	3.1346246	1.96848477	1.59240481	0.11129378	0.35018806	0.95352912	0.455698671
TNFSF4	86.2687955	2.87998118	1.80918512	1.59186649	0.11141471	0.35018806	0.95305748	0.455698671
RPL8	255.97008	3.9306365	2.46938123	1.59174956	0.11144099	0.35018806	0.95295505	0.455698671
PICALM	107.366389	3.98126274	2.50189165	1.59130102	0.11154185	0.35018806	0.95256217	0.455698671
GABRE	6.11642174	4.65948206	2.92819583	1.59124674	0.11155406	0.35018806	0.95251463	0.455698671
KCNE3	10.3364324	4.03513698	2.53795543	1.58991641	0.11185365	0.35072183	0.95134984	0.455037199
NONO	29.4954826	3.78473278	2.38596202	1.58625022	0.11268256	0.35261401	0.9481433	0.452700442
STAU1	30.6893011	3.00567053	1.89497812	1.58612413	0.11271115	0.35261401	0.94803311	0.452700442
MYH10	3.07107896	3.74886985	2.36427395	1.5856326	0.11282268	0.35269792	0.9476036	0.452597107
TNPO2	6.98491407	3.55192414	2.2415867	1.584558	0.11306679	0.35272191	0.94666492	0.452567568
CPNE1	186.765704	4.24407593	2.68325376	1.58169011	0.11372033	0.35417462	0.94416188	0.450782566
RPLP1	164.693583	4.23798083	2.6825546	1.57983022	0.11414575	0.35485907	0.94254024	0.449944094
RBM12	26.6475405	3.67819009	2.32824108	1.57981496	0.11414925	0.35485907	0.94252694	0.449944094
ENDOD1	188.483426	3.07743885	1.9513359	1.57709334	0.11477405	0.35533022	0.94015629	0.449367857
MFSD12	7.54614265	3.04658384	1.93271823	1.57632074	0.1149519	0.35556366	0.93948383	0.449082637
FES	3.47462427	4.65396543	2.95528594	1.57479361	0.11530409	0.35617673	0.93815528	0.448334456
RPL13	64.45229	3.21060197	2.03937261	1.57430867	0.11541611	0.35625847	0.93773358	0.448234806
C1orf198	42.4590237	4.23625719	2.6919303	1.57368755	0.1155597	0.35643748	0.93719359	0.448016633
RPS15	144.395922	2.65139897	1.68532012	1.57323166	0.11566519	0.35649877	0.93679734	0.447941968
PSMA2	94.0979146	3.60651744	2.30115188	1.56726615	0.11705249	0.35997653	0.93161933	0.443725814
GPBP1	168.861978	3.17727148	2.03200788	1.56361179	0.11790876	0.36127454	0.92845391	0.442162648
USP7	160.903729	3.00157698	1.92771748	1.5570627	0.1194556	0.36547621	0.92279349	0.437140889
RPL19	207.289252	4.21672477	2.71196727	1.55485828	0.11997982	0.36627272	0.92089178	0.436195428
BEX4	64.699385	4.30202637	2.76837235	1.55399123	0.12018651	0.36660182	0.92014428	0.435805388
ATP9A	55.5152693	3.47295931	2.23532958	1.55366767	0.12026371	0.36660182	0.91986541	0.435805388
NARF	20.1218846	2.96332371	1.90791493	1.55317392	0.12038159	0.36665882	0.91943993	0.435737864
YAF2	5.97172675	4.13462195	2.662599	1.55285191	0.12045852	0.36665882	0.91916249	0.435737864
DNMT1	6.10239631	2.89811086	1.86998733	1.54980241	0.12118895	0.3675437	0.91653698	0.43469102
RXRA	3.46551968	4.57633895	2.95356639	1.54942816	0.12127883	0.3675437	0.91621501	0.43469102
SWT1	5.91046535	4.37954579	2.82941616	1.54786201	0.12165552	0.3676916	0.91486817	0.434516297
RAB6B	36.8590936	3.96222726	2.55998038	1.54775689	0.12168084	0.3676916	0.9147778	0.434516297
TAX1BP1	32.1739738	3.58939315	2.32176552	1.54597573	0.12211043	0.36845494	0.91324725	0.433615611
NAP1L4	297.944316	2.80336667	1.81431898	1.5451344	0.12231376	0.36858832	0.91252469	0.433458436
EEF1G	22.9016225	4.47795879	2.89824198	1.54506036	0.12233166	0.36858832	0.91246111	0.433458436
FAM96B	10.523604	4.45370824	2.88710206	1.54262238	0.12292243	0.37010049	0.91036888	0.431680337
RCOR3	71.614042	3.75801375	2.43871122	1.5409835	0.1233208	0.37085204	0.90896366	0.430799332
DYNLRB1	3.9384353	3.06626598	1.99482683	1.53710885	0.12426666	0.3722413	0.90564539	0.429175449
MAP1LC3B	30.3120939	3.4163708	2.22307925	1.53677418	0.12434862	0.3722413	0.90535904	0.429175449
LRRC7	3.50076623	2.87146033	1.87458591	1.5317838	0.12557579	0.37480141	0.90109406	0.426198781
PIGC	121.219437	3.42322188	2.23664461	1.53051668	0.12588889	0.37480141	0.9000126	0.426198781
TGFBR2	9.61492044	4.45526679	2.91110213	1.53043988	0.12590788	0.37480141	0.89994707	0.426198781
ZEB2	209.594372	2.55058484	1.66664034	1.53037508	0.12592391	0.37480141	0.89989179	0.426198781
KPNA3	10.8409863	3.59755485	2.3546496	1.52785147	0.12654942	0.37612547	0.89773984	0.424667254
BECN1	31.2294078	4.15014554	2.72158323	1.5249012	0.12728374	0.37776871	0.89522707	0.422774018
PATL2	98.9328163	4.28437094	2.81164045	1.52379759	0.12755928	0.37816329	0.89428794	0.422320628
SYF2	70.8227904	3.38349933	2.22509973	1.5206057	0.12835882	0.37978873	0.8915743	0.420457923
PDE5A	179.731546	2.56982946	1.69118415	1.51954443	0.12862551	0.38012536	0.89067288	0.420073152
GNL1	4.03339122	3.75207793	2.47219533	1.51771095	0.12908728	0.38121946	0.88911655	0.418824938
CDC37	56.4933328	4.22349157	2.78663581	1.51562381	0.1296145	0.38223427	0.8873464	0.417670375
PIP5K1B	264.160592	3.64977139	2.4094894	1.51474889	0.12983601	0.38249412	0.88660485	0.417375233
UBAC1	147.771854	3.47697865	2.2962663	1.5141879	0.12997819	0.38249412	0.88612951	0.417375233
RANBP10	20.1423457	3.55052296	2.34623363	1.51328619	0.13020698	0.382772	0.88536574	0.417059841
YBX1	27.4915488	4.03677054	2.66885176	1.51254956	0.13039412	0.382772	0.88474201	0.417059841
SP2	37.3867118	3.14623907	2.08083716	1.51200639	0.13053224	0.382772	0.88428222	0.417059841
PPP2CA	94.3371902	4.21040588	2.78785423	1.51026759	0.13097516	0.38380053	0.88281107	0.415894425
CLIP2	37.7488782	2.97712118	1.97413616	1.50806274	0.13153847	0.3840892	0.88094721	0.4155679
AASDHPPT	4.36802693	4.4295758	2.93729127	1.50804786	0.13154228	0.3840892	0.88093464	0.4155679
G3BP1	4.43224151	4.22915267	2.80452079	1.50797694	0.13156043	0.3840892	0.88087472	0.4155679
EEF1A1	335.149036	3.14229925	2.08414469	1.50771646	0.13162711	0.3840892	0.88065465	0.4155679

ARF1	186.208259	3.02625373	2.01000956	1.50559171	0.13217202	0.38487013	0.87886048	0.414685797
TRAM1	22.2573593	3.02986447	2.01479916	1.50380471	0.13263166	0.38517967	0.8773528	0.414336638
HECTD4	431.43693	2.08739471	1.38813541	1.50373998	0.13264833	0.38517967	0.87729821	0.414336638
CD63	60.9228752	2.54998219	1.69927513	1.50062938	0.13345145	0.3858971	0.87467671	0.413528485
DCAF17	15.9628175	3.98265512	2.6556521	1.49969008	0.1336947	0.38613945	0.87388581	0.413255832
NUDT3	15.6587463	2.7093403	1.80672108	1.49958969	0.13372072	0.38613945	0.87380129	0.413255832
PRDM5	3.74425979	3.91446136	2.61257563	1.49831504	0.13405142	0.3865927	0.87272859	0.41274635
TRNT1	42.3403882	3.52252185	2.35106097	1.49826903	0.13406337	0.3865927	0.87268988	0.41274635
CHMP3	6.11150563	3.5322763	2.36694597	1.492335	0.13561135	0.38967671	0.86770397	0.409295547
NCKAP1	109.981705	2.60321301	1.74624209	1.4907515	0.13602675	0.38967671	0.86637568	0.409295547
USP43	3.83769085	3.86929307	2.59564648	1.49068569	0.13604403	0.38967671	0.8663205	0.409295547
GAPDH	16.7923827	3.23628866	2.17127052	1.49050458	0.13609161	0.38967671	0.86616864	0.409295547
YIPF4	21.3925878	2.86959867	1.92560044	1.49023578	0.13616225	0.38967671	0.86594327	0.409295547
UBE2H	44.2047071	3.02872868	2.03467978	1.488553	0.13660512	0.39037173	0.86453303	0.408521645
MSI2	146.250397	3.78824347	2.54564321	1.48812821	0.13671709	0.39037173	0.86417721	0.408521645
RBM18	10.444361	3.60082142	2.42008699	1.48788925	0.1367801	0.39037173	0.86397707	0.408521645
FAM134C	3.83909141	4.13348207	2.78674804	1.48326365	0.13800438	0.39332664	0.86010714	0.405246634
IFT20	3.21641226	4.37344991	2.9517654	1.48163872	0.13843645	0.39400838	0.85874954	0.40449454
SPTLC2	49.2712687	2.96629814	2.00250058	1.48129702	0.13852744	0.39400838	0.85846418	0.40449454
H1F0	82.8144652	3.04748255	2.05972534	1.47955773	0.13899131	0.39447013	0.85701234	0.403985876
TAPBP	46.3469647	3.08464821	2.08525322	1.47926793	0.13906872	0.39447013	0.85677054	0.403985876
TCF23	4.14603366	4.34266216	2.93715473	1.47852686	0.13926681	0.39449456	0.85615236	0.403958977
DHX34	36.4733938	3.74724934	2.53988664	1.47536086	0.14011555	0.39662893	0.85351366	0.40161561
MAP3K7CL	90.5496319	3.27205754	2.22196467	1.47259656	0.14085985	0.39819445	0.85121277	0.399904796
CHMP2A	131.347558	2.81986899	1.92219907	1.46700154	0.14237564	0.40111783	0.84656432	0.396728035
LRP1B	5.60072561	3.10482308	2.12160115	1.46343391	0.14334868	0.40330025	0.84360629	0.39437151
CDV3	14.6993275	3.34616777	2.28744562	1.46284036	0.14351107	0.40330025	0.84311461	0.39437151
HOOK1	4.72867077	3.86189018	2.6408085	1.46238933	0.14363455	0.40330025	0.84274108	0.39437151
TOR1A	8.82954816	4.23936857	2.90066825	1.46151446	0.14387431	0.40352152	0.84201674	0.394133299
MGAT3	4.93366752	3.3150218	2.26839603	1.46139464	0.14390717	0.40352152	0.84191757	0.394133299
PPP3CB	13.8116012	3.49549998	2.39839531	1.45743279	0.14499695	0.40577775	0.83864113	0.391711772
ARID2	57.145914	3.1276246	2.14638834	1.45715691	0.14507307	0.40577775	0.83841319	0.391711772
PCM1	266.472792	2.16211957	1.4838985	1.45705354	0.1451016	0.40577775	0.8383278	0.391711772
ATP11B	79.7051133	2.45027962	1.68386297	1.45515381	0.14562668	0.40697283	0.83675904	0.390434588
TMSB10	17.5715924	3.76928845	2.5943576	1.4528793	0.14625727	0.40838797	0.83488254	0.388927062
PSMA7	111.855151	3.04585069	2.0974426	1.45217356	0.14645335	0.40838797	0.83430068	0.388927062
FAM114A2	3.42325832	4.27492231	2.94511257	1.45153104	0.14663205	0.40841193	0.83377111	0.38890158
RPL10A	57.6926109	3.71623461	2.56355501	1.44964106	0.14715864	0.40960466	0.83221424	0.387635111
GAPVD1	525.878018	2.90028947	2.00670529	1.44529916	0.14837387	0.41221601	0.82864256	0.384875148
PKIG	5.70588707	4.22267075	2.9218021	1.44522818	0.1483938	0.41221601	0.82858424	0.384875148
CDC42	20.5383633	2.77394295	1.92248789	1.44289229	0.14905081	0.4134894	0.82666565	0.383535617
AK3	3.33123218	4.23709734	2.94535135	1.4385711	0.15027208	0.41586529	0.8231217	0.381047322
RPL37A	57.2371202	2.88647563	2.00667912	1.43843408	0.15031093	0.41586529	0.82300944	0.381047322
DR1	75.3750839	3.31699462	2.30651706	1.43809672	0.15040661	0.41586529	0.82273307	0.381047322
METAP1	35.5699749	3.40363745	2.36833379	1.43714432	0.15067699	0.41633642	0.82195306	0.380555596
FAM228B	4.90455043	4.19412866	2.9266522	1.43308066	0.15183479	0.41842492	0.81862871	0.378382455
SLC25A38	4.65689068	4.05678433	2.83579202	1.43056483	0.15255497	0.41985387	0.81657363	0.376901839
CDC5L	31.800469	3.29074758	2.30171188	1.4296957	0.15280437	0.42021041	0.81586421	0.376533188
CPEB4	488.556968	1.95014568	1.36448113	1.42922143	0.1529406	0.42021041	0.81547721	0.376533188
BBOF1	47.5067139	3.47813341	2.43390539	1.42903394	0.15299448	0.42021041	0.81532425	0.376533188
CBX4	15.3649485	3.93234305	2.75285214	1.42846141	0.1531591	0.42021041	0.81485721	0.376533188
FAM175A	5.94665336	3.78003663	2.64669338	1.42821101	0.15323113	0.42021041	0.81465299	0.376533188
CAMK1D	5.21371062	3.5822969	2.50860002	1.42800641	0.15329001	0.42021041	0.81448614	0.376533188
AMD1	77.4858723	1.69613312	1.18810594	1.42759418	0.1534087	0.4202591	0.81415001	0.376482875
KARS	33.041993	3.63483486	2.55269326	1.42392152	0.15446919	0.42260822	0.81115813	0.374062057
VPS26A	5.79004195	4.15713027	2.92039876	1.42348035	0.15459695	0.42268005	0.81079907	0.373988252
PDZK1IP1	414.584857	3.00622071	2.11318586	1.42260119	0.1548518	0.42309726	0.81008374	0.373559786
CPSF7	3.20573016	4.19009895	2.94609921	1.42225317	0.15495277	0.42309726	0.80980066	0.373559786
ANAPC2	3.02777875	4.19213607	2.94965001	1.42123169	0.15524942	0.42362947	0.80897003	0.37301384
DHX38	7.72372887	3.1352918	2.20814998	1.41987267	0.15564475	0.42436938	0.80786551	0.37225596
X6.Mar	544.325767	1.70499605	1.20126226	1.41933706	0.15580077	0.42436938	0.8074304	0.37225596
RAD50	20.4776483	3.29489165	2.32157338	1.41924941	0.15582632	0.42436938	0.8073592	0.37225596

GUCY1A3	72.7873512	3.4322316	2.42795285	1.4136319	0.15747001	0.42828551	0.80280215	0.368266615
TADA1	41.5342389	4.08781957	2.89293004	1.41303782	0.1576446	0.4284805	0.80232089	0.368068937
NOL4L	660.008254	1.92023853	1.3609175	1.41098819	0.15824809	0.42975559	0.80066151	0.366778471
PHACTR2	5.99389844	3.55136681	2.51737173	1.4107439	0.15832014	0.42975559	0.80046383	0.366778471
UBE2G2	2.80218559	4.16429152	2.95324692	1.41007225	0.15851835	0.4300133	0.79992045	0.366518114
TBL1X	7.16205396	3.24681955	2.30376608	1.40935296	0.15873082	0.43030935	0.79933873	0.366219222
MYO10	16.8832979	3.25832341	2.31449078	1.40779278	0.15919244	0.43127997	0.79807756	0.365240709
NDUFB2	3.12545389	2.97916909	2.11986563	1.40535751	0.15991499	0.4325124	0.79611081	0.364001437
PAFAH1B1	72.2265139	2.54283139	1.80977633	1.40505285	0.16000556	0.4325124	0.79586491	0.364001437
FOXK1	6.57456422	2.80185162	1.99439876	1.40486029	0.16006283	0.4325124	0.79570952	0.364001437
TBC1D17	22.2191457	2.89309279	2.0605303	1.40405254	0.16030321	0.43276816	0.79505779	0.363744699
ELAC2	10.0311891	4.07990528	2.90623857	1.4038439	0.16036534	0.43276816	0.79488949	0.363744699
MEGF8	3.94748242	2.72452348	1.94231194	1.40272189	0.16069979	0.43311119	0.7939847	0.363400593
XPR1	11.9743183	3.17209962	2.26319766	1.40160079	0.16103449	0.4331703	0.79308109	0.363341328
RSU1	504.902883	2.79339838	1.99370583	1.4011086	0.1611816	0.43317671	0.79268453	0.363334906
INPP5F	3.11883828	1.85839185	1.32657297	1.40089682	0.16124493	0.43317671	0.79251393	0.363334906
IGSF9B	335.290839	2.87083191	2.0511334	1.39963199	0.16162355	0.43363432	0.79149536	0.362876353
SUPT16H	2.84775306	4.12655606	2.95150016	1.39812158	0.16207656	0.43456974	0.79027979	0.36194052
RDH13	3.53059826	3.18830198	2.28345273	1.39626362	0.16263512	0.43494712	0.78878565	0.361563544
PPP1R14A	18.009917	3.1670632	2.26997151	1.39519954	0.16295568	0.43524531	0.78793051	0.361265903
B4GALT3	28.6651105	3.67618286	2.6372373	1.39395225	0.16333203	0.43590585	0.78692865	0.360607307
VPS13B	91.642796	2.82445852	2.02660992	1.39368632	0.16341235	0.43590585	0.78671512	0.360607307
DEDD	10.2859133	4.04224383	2.90546388	1.39125592	0.16414784	0.43702787	0.78476484	0.359490866
HSPA9	127.672274	3.02453571	2.17519114	1.39046894	0.16438653	0.43714478	0.78413378	0.359374699
USMG5	13.0897442	3.52106723	2.53820035	1.38722983	0.16537169	0.43888319	0.78153883	0.357651051
ADGRE5	5.6799169	3.63363959	2.62336057	1.38510871	0.16601923	0.4398008	0.77984161	0.356743984
EXOSC6	4.00535605	3.62891108	2.62057642	1.38477591	0.166121	0.4398008	0.77957546	0.356743984
LTB4R	2.73902477	3.56995225	2.57811517	1.38471403	0.16613993	0.4398008	0.77952598	0.356743984
FEM1A	21.9752393	2.29678551	1.65956099	1.38397174	0.16636711	0.44012239	0.77893253	0.35642654
MFSD2B	418.421365	2.35838869	1.70492228	1.38328223	0.16657834	0.44040141	0.77838146	0.356151297
ATP5L	12.8414968	3.60245621	2.61077812	1.37984005	0.1676359	0.44231103	0.77563297	0.354272228
CMC2	51.2102884	2.93760966	2.13070777	1.37870134	0.16798686	0.4426666	0.7747247	0.35392324
ATF4	66.7007747	2.54519453	1.84737804	1.37773345	0.1682856	0.4426666	0.77395305	0.35392324
UBC	131.1669	2.52472906	1.83309899	1.377301	0.16841921	0.44273837	0.77360838	0.353852837
EHBP1L1	14.8314698	2.35935574	1.71468484	1.37597049	0.16883077	0.44314141	0.7725484	0.353457667
FAM65A	4.47923002	3.40884692	2.47776767	1.37577343	0.16889179	0.44314141	0.77239146	0.353457667
MPHOSPH8	54.5765903	3.0590997	2.23161354	1.37080173	0.17043677	0.44691355	0.7684367	0.349776477
LAMTOR3	83.5059195	3.6361529	2.65654303	1.36875363	0.1710763	0.44774464	0.76681015	0.348969606
HGSNAT	2.97433806	2.86050117	2.0923384	1.36713123	0.17158418	0.44852856	0.76552277	0.348209901
NPC1	55.0757938	2.91114939	2.12942067	1.36710864	0.17159126	0.44852856	0.76550485	0.348209901
GGNBP2	150.649427	3.10647624	2.27396087	1.36610805	0.17190505	0.4490669	0.76471136	0.347688953
SEC24B	12.1144369	3.96023109	2.90251994	1.36441133	0.17243814	0.44992625	0.76336666	0.34685867
EZH1	38.0508744	3.68848213	2.70407132	1.36404765	0.17255257	0.44992625	0.76307857	0.34685867
QSOX1	5.73769134	3.63995336	2.68312257	1.35661091	0.17490487	0.45524277	0.75719809	0.341756938
FCF1	10.257076	2.31990658	1.71015564	1.35654704	0.17492518	0.45524277	0.75714768	0.341756938
KANSL1L	3.16508759	3.21293027	2.36999493	1.35566968	0.17520429	0.45568436	0.75645526	0.341335876
KIAA1468	31.7795198	2.85782968	2.11001783	1.3544102	0.17560554	0.4562459	0.75546178	0.340801022
RPS23	64.9154484	3.14675385	2.32352063	1.35430424	0.17563933	0.4562459	0.75537822	0.340801022
MTCL1	6.87006578	3.18871652	2.37314144	1.34366897	0.17905545	0.46309746	0.74701244	0.334327599
C17orf62	4.16480266	3.92681369	2.92755191	1.34133017	0.17981328	0.46432879	0.74517824	0.33317439
ARPP19	120.372721	2.71486121	2.02484599	1.34077418	0.17999378	0.46437058	0.7447425	0.333135305
PITPNM2	161.028323	1.61803474	1.20738113	1.34011929	0.18020657	0.46463167	0.74422939	0.332891196
TMEM2	5.89949037	2.40970455	1.79961949	1.33900781	0.18056813	0.4652758	0.7433589	0.332289532
CDS2	374.265419	2.21355931	1.65737909	1.33557815	0.18168719	0.46786981	0.74067568	0.32987498
RPL5	93.5791066	2.78594973	2.08671696	1.3350875	0.18184771	0.46799374	0.74029216	0.329759959
NCBP3	6.55383521	2.93141705	2.19747933	1.33399073	0.1822069	0.46862848	0.73943519	0.329171318
ATP5EP2	7.84001566	3.10317182	2.32705665	1.33351795	0.18236189	0.46873759	0.73906592	0.329070215
FAM134A	3.84984548	3.0226384	2.2678361	1.3328293	0.18258783	0.46902882	0.73852818	0.32880047
TOLLIP	49.2174221	2.4618573	1.84944875	1.33113032	0.18314613	0.47017294	0.73720224	0.327742371
RAB5C	4.19081604	2.5604743	1.92741078	1.32845283	0.18402856	0.47214721	0.73511478	0.325922577
PDIA3	4.46970979	2.81910868	2.12320219	1.3277627	0.18425651	0.47244096	0.73457715	0.325652458
ATAD2	3.99443589	3.88305264	2.92824453	1.32606844	0.18481703	0.47300437	0.73325801	0.325134845

RAB27B	185.230539	2.23754852	1.69023624	1.32380815	0.18556677	0.47461981	0.7314998	0.32365414
GANAB	103.476677	3.10190505	2.34431141	1.32316254	0.18578133	0.47461981	0.73099793	0.32365414
FAM214B	118.086731	2.20621857	1.6674164	1.32313594	0.18579017	0.47461981	0.73097726	0.32365414
DCTN6	4.86601841	3.75852235	2.84172201	1.32262141	0.18596131	0.47476573	0.7305774	0.323520638
CASC3	59.1342486	3.10954678	2.35253457	1.3217858	0.18623949	0.47518458	0.72992823	0.323137663
ACIN1	24.7370602	2.52173167	1.91390207	1.31758657	0.18764208	0.47817725	0.72666976	0.320411086
MPL	122.195304	2.47018952	1.87597563	1.31674926	0.18792269	0.47859943	0.72602079	0.320027823
NUP98	12.8471835	3.42533339	2.60414263	1.31534016	0.18839561	0.47930492	0.72492923	0.319388116
TUB	3.89043796	3.85176759	2.92857068	1.31523805	0.18842991	0.47930492	0.72485016	0.319388116
RBMS2	2.80399875	2.8036005	2.13651374	1.31223144	0.18944206	0.48070489	0.7225236	0.318121456
UBXN1	39.3510739	3.61520093	2.76000296	1.30985401	0.19024523	0.48244892	0.72068623	0.316548657
SUPV3L1	31.9531282	3.73307142	2.85354411	1.30822278	0.19079776	0.48341398	0.71942674	0.315680797
SF3B4	3.25809362	3.84084036	2.93632075	1.30804523	0.19085797	0.48341398	0.7192897	0.315680797
PPP1CB	161.91924	3.12503378	2.3971057	1.30366958	0.19234627	0.48618361	0.71591624	0.313199686
WASF1	50.768554	3.76911235	2.89162575	1.30345787	0.19241849	0.48618361	0.7157532	0.313199686
PRDX6	39.0026566	3.32983835	2.55582963	1.3028405	0.19262922	0.48642091	0.71527783	0.312987764
EDEM2	2.72197263	3.59941876	2.7635759	1.30244975	0.19276268	0.48646292	0.71497704	0.31295026
RGS6	74.4229036	3.02464134	2.3231027	1.30198348	0.19292203	0.48657016	0.71461817	0.312854525
HNRNPF	11.3155924	3.57545117	2.7470519	1.30155938	0.19306705	0.48664117	0.71429183	0.312791153
DPP9	86.9118321	3.24487127	2.49401167	1.30106499	0.19323621	0.48677289	0.71391149	0.312673621
SH2B3	174.830619	2.63660691	2.02988273	1.29889618	0.19397956	0.48835	0.71224403	0.31126881
CCT6A	6.99071246	3.47452723	2.6807146	1.29611978	0.19493422	0.49016068	0.71011192	0.309661527
GUCY2C	3.11566606	2.73656916	2.11257349	1.29537229	0.19519183	0.49042301	0.70953836	0.30942916
RPL39	5.88728826	3.00098675	2.31749601	1.29492639	0.19534562	0.49042301	0.70919632	0.30942916
GOLGA3	33.3521669	3.27821395	2.53347404	1.29395995	0.19567925	0.49059367	0.70845522	0.309278062
ZNF646	2.87151129	2.52969916	1.95608031	1.29324913	0.19592491	0.49065406	0.70791035	0.309224606
AGPAT1	19.740814	2.49522881	1.9295572	1.29316135	0.19595526	0.49065406	0.70784308	0.309224606
GUK1	124.928034	2.74272891	2.1319177	1.28650788	0.19826587	0.49612098	0.70275204	0.304412404
MAP3K13	45.090927	2.85406562	2.21947829	1.28591734	0.19847191	0.49612098	0.70230095	0.304412404
ANO6	205.721629	1.95701512	1.52196437	1.28584818	0.19849605	0.49612098	0.70224813	0.304412404
CXCL2	33.4302537	3.69581831	2.87810338	1.2841159	0.19910141	0.49733549	0.70092567	0.303350552
ITGA9	113.600662	2.20974473	1.72399886	1.28175533	0.19992849	0.49850433	0.69912532	0.302331069
METTL15	7.40504685	2.63430323	2.06192102	1.27759658	0.20139171	0.50140647	0.69595841	0.299810063
TUBGCP2	5.04990863	3.72721691	2.91778987	1.27741101	0.20145718	0.50140647	0.69581725	0.299810063
SNRPD2	3.77596143	3.59889171	2.81806328	1.27707981	0.20157407	0.50140647	0.69556533	0.299810063
ATG4B	81.5674188	3.14173064	2.46251437	1.27582225	0.20201836	0.50221161	0.69460916	0.299113251
PSMD2	13.7668463	2.62336693	2.05790725	1.27477413	0.2023892	0.50270612	0.69381267	0.298685826
KIF5A	3.84109764	3.60830774	2.83098282	1.27457776	0.20245873	0.50270612	0.69366348	0.298685826
ZNF791	13.3065556	1.05094485	0.82539837	1.27325772	0.2029266	0.50306347	0.69266101	0.298377216
BTG3	15.8865105	3.50731561	2.75497427	1.27308471	0.20298799	0.50306347	0.69252967	0.298377216
FUBP3	4.91804961	3.59962822	2.82809818	1.27280879	0.2030859	0.50306347	0.69232022	0.298377216
USP33	97.0004898	2.09992436	1.65213262	1.27103861	0.20371491	0.50432158	0.69097718	0.297292448
RTCB	50.0463735	3.67181423	2.89152955	1.26985188	0.2041374	0.50506721	0.69007743	0.296650822
RPS20	25.7509644	3.25981002	2.57058895	1.26811796	0.20475583	0.50629648	0.68876373	0.295595093
RPS25	139.038706	2.57938199	2.03841469	1.26538628	0.20573289	0.50841054	0.68669627	0.293785457
RACK1	26.7509816	2.61538882	2.07111893	1.26279026	0.20666456	0.50994925	0.68473398	0.292473042
RPL36	48.2425791	3.05572032	2.42121174	1.26206241	0.20692633	0.51014875	0.68418425	0.292303174
TXNL1	28.3723923	2.1064237	1.67019513	1.2611842	0.20724249	0.51048562	0.6835212	0.292016484
AP2S1	37.3508109	3.18524241	2.52664327	1.2606617	0.20743076	0.51048562	0.68312685	0.292016484
KBTBD2	61.1595293	3.06390094	2.43338746	1.25910937	0.20799083	0.51125963	0.68195581	0.291358494
SLC16A1	163.501145	3.44820134	2.74223366	1.25744257	0.20859342	0.51213621	0.68069939	0.290614518
TRPM7	4.01018664	2.04331656	1.62550288	1.25703657	0.20874039	0.51219505	0.6803935	0.290564622
EIF4EBP2	25.6822236	2.48898519	1.98490936	1.25395408	0.2098587	0.51430863	0.67807303	0.288776191
HGS	210.700846	2.76061756	2.20207876	1.2536416	0.2099723	0.51430863	0.67783799	0.288776191
CNPPD1	29.3256624	2.58923452	2.06625258	1.25310649	0.21016695	0.51448277	0.67743557	0.288629163
MAN1A1	5.82688607	2.9211606	2.33228119	1.25249074	0.2103911	0.51472887	0.67697264	0.288421472
SFMBT2	46.7788837	2.69036369	2.14889412	1.25197592	0.21057864	0.51476196	0.67658569	0.288393553
CTR9	172.315839	2.63443918	2.10535228	1.25130564	0.21082299	0.51487796	0.67608203	0.288295695
KDM6B	53.6993373	2.65666902	2.12564114	1.2498201	0.21136527	0.51552024	0.67496637	0.287754279
RAB21	33.3434662	2.38094097	1.90541124	1.24956803	0.21145739	0.51552024	0.67477713	0.287754279
RALGAPB	7.16202772	3.09336732	2.47630754	1.24918544	0.21159726	0.51555938	0.67448997	0.28772131
VCPIP1	69.2221788	3.07256042	2.46118866	1.24840508	0.21188275	0.51595308	0.6739044	0.287389787

FAM204A	77.3245182	2.73740905	2.19603652	1.24652255	0.21257261	0.51716619	0.67249269	0.286369879
GPATCH2L	3.1842027	2.09314355	1.68004857	1.24588276	0.21280744	0.51729725	0.67201319	0.286259832
PRICKLE2	36.9788867	3.56321135	2.86243174	1.24481967	0.21319804	0.51794437	0.67121679	0.285716883
C2CD5	11.0552797	3.60373425	2.90126315	1.24212595	0.2141901	0.51919659	0.66920061	0.284668171
DAPK1	66.1861939	2.47767648	1.99479349	1.24207167	0.21421012	0.51919659	0.66916002	0.284668171
GOSR2	3.15409206	2.65022731	2.13607665	1.2406986	0.21471711	0.51922423	0.66813335	0.28464505
NDUFAF3	56.4505857	3.21092058	2.58910743	1.24016507	0.21491435	0.51922423	0.66773459	0.28464505
ATXN3	42.7354351	2.43382586	1.9626526	1.24006962	0.21494965	0.51922423	0.66766327	0.28464505
GNB2	32.9186264	2.67216362	2.15495377	1.24000972	0.2149718	0.51922423	0.66761851	0.28464505
NUDT4	12.0198814	2.05435163	1.65871398	1.23852071	0.21552305	0.51935067	0.66650628	0.2845393
MOB3C	223.424103	2.16530639	1.75271708	1.23539983	0.21668174	0.5218408	0.6641777	0.282461968
SAFB	159.677638	2.19160265	1.7758976	1.23408166	0.21717248	0.52272034	0.66319521	0.281730597
TP53INP1	160.017941	2.32231183	1.88401559	1.23263939	0.21771033	0.52363127	0.66212096	0.280974428
METTL12	12.9421962	2.97340479	2.41303953	1.23222382	0.21786549	0.52363127	0.66181156	0.280974428
TIAL1	42.4190353	2.80586408	2.27820137	1.23161373	0.21809341	0.52363127	0.66135746	0.280974428
CDK5RAP2	13.9972798	3.04472623	2.47294721	1.2312136	0.21824299	0.52363127	0.6610597	0.280974428
MINDY2	87.2958918	1.99161635	1.617622	1.23120009	0.21824804	0.52363127	0.66104965	0.280974428
SLBP	4.70668091	2.8538114	2.31819938	1.23104657	0.21830545	0.52363127	0.66093543	0.280974428
ABCD3	55.4319633	2.99040776	2.43507146	1.2280575	0.21942538	0.5254096	0.65871313	0.279501993
RCOR1	92.331696	3.01767872	2.47386309	1.21982446	0.22253143	0.53113334	0.65260865	0.274796438
ANKLE1	109.137634	3.51989801	2.88987306	1.21801129	0.22321968	0.53204737	0.65126751	0.274049699
EFHC2	48.7617064	2.93635057	2.41246994	1.2171553	0.22354513	0.53251827	0.65063478	0.273665485
GRIPAP1	3.77883821	2.96953868	2.45412385	1.21001989	0.22627126	0.53760924	0.64537061	0.269533279
NLRC5	1479.15791	2.4183854	1.99887619	1.20987253	0.22632781	0.53760924	0.64526208	0.269533279
ADGRL3	7.88612225	2.41007114	1.99295838	1.20929326	0.2265502	0.53783069	0.64483555	0.269354423
EXOC4	4.97577585	2.67092942	2.22007269	1.20308197	0.2289446	0.54289597	0.64026959	0.26528338
VASP	24.100517	2.62425323	2.18217106	1.20258823	0.22913571	0.54303988	0.63990723	0.265168272
LEMD3	33.9022393	2.513669	2.09241872	1.20132218	0.22962625	0.5438929	0.63897846	0.264486613
TMA7	28.47971	2.35755558	1.96423285	1.20024242	0.23004521	0.54423588	0.63818681	0.264212826
MORF4L1	88.0854418	2.58111138	2.15133961	1.19976937	0.23022892	0.54423588	0.63784012	0.264212826
ATP8B2	58.4885267	2.7391842	2.28321198	1.19970647	0.23025336	0.54423588	0.63779403	0.264212826
SLC2A11	24.1002724	2.84310883	2.37373676	1.19773552	0.23102	0.54564224	0.63635042	0.263092016
GTPBP1	82.0754091	2.34699607	1.96143518	1.19657081	0.23147389	0.54640434	0.635498	0.262485859
ANKRD33B	140.015314	2.46284063	2.05975065	1.19569844	0.23181426	0.54689778	0.63485985	0.262093837
YY1AP1	8.77702937	3.46524027	2.90351578	1.19346356	0.23268788	0.54833747	0.63322624	0.260952072
THAP5	51.551123	3.44156407	2.89116527	1.19037265	0.23389996	0.55057062	0.63096986	0.259186968
MTRNR2L10	2.76288249	1.86433478	1.56673538	1.18994873	0.23406654	0.55065146	0.63066066	0.259123206
STIP1	6.9825299	2.54410891	2.14374757	1.18675769	0.23532321	0.55329524	0.62833523	0.257043069
PCMTD1	99.71954	2.33803227	1.97202246	1.18560124	0.23577981	0.55405595	0.62749338	0.256446377
EXOC7	5.98125537	3.12577492	2.63751463	1.18512136	0.23596947	0.55418887	0.62714419	0.256342203
TMEM183A	25.6174221	3.36584682	2.84396815	1.1835037	0.23660958	0.55457443	0.62596767	0.256040155
CKS2	21.5435371	3.42542385	2.89465834	1.18336033	0.23666637	0.55457443	0.62586345	0.256040155
SDHA	10.458625	3.09420427	2.6156444	1.1829606	0.23682476	0.55463347	0.62557289	0.255993924
JRKL	4.5289338	2.72703499	2.30765247	1.18173556	0.23731064	0.55545898	0.62468278	0.255348011
PYROXD2	5.29698008	3.14864663	2.66519361	1.18139508	0.23744581	0.55546312	0.62443549	0.255344771
RASA2	3.91046577	2.37638204	2.01415309	1.17984182	0.23806313	0.55628221	0.62330785	0.254704831
WAC	227.435029	2.17773552	1.84676407	1.17921697	0.23831179	0.55655093	0.62285447	0.25449509
PAQR7	37.7047601	2.76814754	2.35037626	1.17774655	0.23889766	0.55760642	0.6217881	0.253672231
ZFAND5	113.788951	2.26658462	1.92524535	1.17729651	0.23907718	0.55771282	0.62146187	0.253589374
IRS1	56.7826633	2.56247379	2.17933875	1.17580334	0.23967347	0.55840241	0.62038004	0.253052718
PIAS1	105.540621	2.73272079	2.32463343	1.17554912	0.2397751	0.55840241	0.62019592	0.253052718
TAOK1	280.15375	1.07366702	0.91701242	1.17083149	0.24166652	0.56186453	0.61678352	0.250368385
SORBS1	334.429066	2.17588424	1.8620234	1.16855902	0.24258135	0.56363125	0.6151426	0.249004933
IL6ST	231.680112	1.91615708	1.64107061	1.16762622	0.24295757	0.56392158	0.61446957	0.248781287
PSIP1	30.1434715	2.21529142	1.90154165	1.16499758	0.24401997	0.56607196	0.61257463	0.247128357
PSMD6	9.32613185	3.1839503	2.74396393	1.16034699	0.24590756	0.56949893	0.60922812	0.244507088
MYL6	246.901952	2.13637144	1.8427006	1.15936981	0.24630548	0.5700645	0.60852593	0.244076001
USP21	13.5738078	3.05298367	2.63398264	1.15907509	0.24642558	0.5700645	0.60831422	0.244076001
UQCR10	65.475647	3.00572619	2.60027291	1.1559272	0.24771094	0.57200565	0.60605482	0.24259968
ARMC7	11.0684477	2.23332081	1.93302785	1.15534849	0.24794775	0.57200565	0.60563984	0.24259968
SMC5	6.29205545	2.89778642	2.51086322	1.15409967	0.24845931	0.57200565	0.60474473	0.24259968
EBF1	13.6437735	2.66874252	2.31246121	1.15407018	0.2484714	0.57200565	0.6047236	0.24259968

TNRC6C	71.1713534	2.71765217	2.35583176	1.153585	0.24867035	0.5720792	0.60437599	0.242543845
SMARCA2	66.6687132	2.17109252	1.88395912	1.15240957	0.24915281	0.57287261	0.60353421	0.241941939
CELF2	112.476332	1.97578905	1.71579888	1.15152718	0.24951542	0.57307347	0.60290261	0.241789695
FOXJ3	27.6802056	2.76290136	2.40023898	1.15109428	0.24969345	0.57316622	0.60259285	0.241719411
PPP6R1	108.574587	2.51248536	2.18491598	1.14992311	0.25017554	0.5736404	0.60175515	0.241360272
C17orf97	5.5210333	2.50381086	2.17914141	1.14898962	0.25056026	0.5738905	0.6010878	0.241170963
MARK2	12.9973436	2.57747052	2.24494802	1.14812035	0.25091888	0.5741526	0.60046666	0.240972665
MYCBP2	122.030949	1.85878829	1.61950503	1.14775086	0.25107143	0.5741526	0.60020271	0.240972665
EIF1AX	16.3671817	2.07423257	1.80727952	1.14770989	0.25108835	0.5741526	0.60017344	0.240972665
TPCN1	98.6016527	2.50565425	2.185981	1.14623789	0.25169673	0.57465082	0.59912242	0.24059597
ITGA6	2.71317611	2.57397269	2.24647192	1.1457845	0.25188433	0.57465082	0.59879885	0.24059597
ARNTL2	2.92882861	2.72973689	2.38339493	1.14531455	0.25207888	0.57465082	0.59846354	0.24059597
SI	3.13563921	3.17753176	2.774629	1.1452096	0.25212234	0.57465082	0.59838866	0.24059597
CDC26	6.55373888	3.3294976	2.90769707	1.14506344	0.25218288	0.57465082	0.5982844	0.24059597
COX16	2.95081431	2.73688096	2.39060707	1.14484768	0.25227226	0.57465082	0.5981305	0.24059597
STIM1	162.259791	2.17885167	1.90447469	1.14406964	0.25259476	0.57475659	0.59757567	0.240516037
ATP5E	965.388707	2.28298854	1.99789308	1.14269806	0.25316398	0.57573719	0.59659809	0.239775717
ITGA4	3.8323294	3.33519624	2.920565	1.14196953	0.25346669	0.57611096	0.59607911	0.239493863
LSM10	3.37633914	3.33890768	2.92473681	1.14160962	0.25361632	0.57613659	0.5958228	0.239474543
AFF1	418.227003	1.60961976	1.4176643	1.13540262	0.25620666	0.58170368	0.59140958	0.235298188
ITGB1	52.4779346	2.26263978	1.99615358	1.13349984	0.25700441	0.58271768	0.59005943	0.234541804
LDHB	19.1173438	2.98521054	2.633771	1.13343587	0.25703126	0.58271768	0.59001406	0.234541804
PPP4C	69.6077537	2.8060236	2.47645526	1.13308067	0.25718038	0.58271768	0.58976217	0.234541804
MAP3K7	6.68573501	2.64526431	2.33473748	1.13300289	0.25721304	0.58271768	0.58970702	0.234541804
DNAJB2	8.67073947	2.10912548	1.86453342	1.13118138	0.25797876	0.58413461	0.58841605	0.233487059
LCOR	575.457701	1.62091128	1.43414658	1.13022706	0.25838056	0.58472644	0.58774016	0.233047265
POMP	135.470571	3.226769	2.85745513	1.12924573	0.25879419	0.58510876	0.58704548	0.2327634
SEC22B	8.09206335	3.1148081	2.75972614	1.12866565	0.2590389	0.58510876	0.58663501	0.2327634
UBXN6	248.934092	1.77708672	1.57520715	1.12816065	0.25925208	0.58510876	0.58627775	0.2327634
PDK1	199.778888	1.68577342	1.49474422	1.12780059	0.25940414	0.58513481	0.58602309	0.232744068
GABARAPL2	135.798846	2.87761154	2.5575217	1.12515626	0.26052283	0.58691746	0.58415422	0.231422972
CHUK	4.26822461	3.17440063	2.82436121	1.12393578	0.26104027	0.58691746	0.58329249	0.231422972
ZNF273	21.9530709	2.25816942	2.00957871	1.1237029	0.26113909	0.58691746	0.58312812	0.231422972
DDX58	17.3486613	2.2629413	2.01450783	1.12332217	0.26130069	0.58691746	0.58285944	0.231422972
NPIPB5	17.3558642	1.08603535	0.96698699	1.12311269	0.26138964	0.58691746	0.58271163	0.231422972
ST3GAL1	354.712473	1.81586631	1.62049204	1.12056478	0.26247316	0.5880053	0.58091509	0.23061876
KPNA4	94.3638173	2.67414727	2.38733086	1.12014104	0.26265366	0.5880053	0.58061654	0.23061876
LSM14A	170.740931	2.73613353	2.44444995	1.11932483	0.26300159	0.58846782	0.58004163	0.230277285
TOP1	321.735582	1.37095422	1.22605031	1.11818757	0.26348689	0.58916216	0.57924099	0.229765154
RBFA	16.2803722	2.58378157	2.31407276	1.11655157	0.26418611	0.59016682	0.57809002	0.22902521
C12orf10	11.8478692	3.23455721	2.89863813	1.11588859	0.26446983	0.59023976	0.57762387	0.228971536
EFCAB7	6.28027841	3.24372843	2.90755594	1.1156203	0.2645847	0.59023976	0.57743527	0.228971536
PRKCB	115.372058	2.1046135	1.88877484	1.11427442	0.26516148	0.59044513	0.57648957	0.228820458
PGAP1	5.76112487	3.24064154	2.90927087	1.11390162	0.26532139	0.59048545	0.57622773	0.228790797
TPP1	108.181707	2.81446155	2.53117001	1.11192118	0.26617204	0.5916336	0.57483757	0.22794717
PCSK6	40.1753317	2.33609256	2.10106031	1.11186364	0.26619679	0.5916336	0.57479719	0.22794717
PHF1	27.0287599	2.41585089	2.17309758	1.11170842	0.26626354	0.5916336	0.5746883	0.22794717
MAP3K1	7.27975486	3.22435394	2.90486706	1.1099833	0.26700622	0.59296742	0.57347862	0.22696917
SBF1	28.6324797	2.54986723	2.29998017	1.10864749	0.26758228	0.593199	0.57254264	0.226799592
LIPH	9.89752993	2.31290594	2.08686025	1.10831855	0.26772427	0.593199	0.57231226	0.226799592
GPR22	10.5786906	3.21299761	2.899719	1.10803757	0.26784559	0.593199	0.5721155	0.226799592
HNRNPA3	62.8527152	2.28151716	2.05957616	1.10776052	0.26796525	0.593199	0.57192152	0.226799592
NF1	21.7573008	1.90133699	1.71746206	1.107062	0.26826712	0.59329083	0.57143255	0.226732366
PPHLN1	16.0852281	2.54665787	2.30117815	1.10667567	0.26843418	0.59329083	0.57116219	0.226732366
SMARCD2	8.15198207	3.21156575	2.90300149	1.10629145	0.26860039	0.59334325	0.57089336	0.226693996
ABCC4	510.05883	1.38987403	1.25751137	1.10525762	0.26904797	0.59374846	0.57017027	0.226397506
HNRNPUL1	32.0435731	2.22179459	2.01029347	1.10520908	0.26906901	0.59374846	0.57013633	0.226397506
KPNA6	51.7619362	2.58230152	2.3383845	1.10431006	0.26945866	0.59399474	0.56950785	0.226217399
COPA	69.315689	2.29458273	2.0778743	1.10429333	0.26946591	0.59399474	0.56949616	0.226217399
CFAP45	48.847726	2.25977633	2.0489342	1.10290332	0.27006915	0.5950095	0.56852502	0.225476102
OGT	213.030041	2.01519736	1.82853631	1.10208222	0.27042593	0.59548047	0.56795167	0.22513248
SMC3	112.258955	2.18675137	1.98719136	1.10042314	0.2711478	0.59643922	0.56679392	0.22443381

TNRC6B	441.557683	1.363123	1.24057869	1.09877996	0.27186406	0.59769901	0.56564821	0.223517462
NT5M	7.95260405	2.82022873	2.56927422	1.09767526	0.27234632	0.59844331	0.56487849	0.222976985
ZNF175	318.811618	2.36950467	2.16083074	1.09657116	0.2728289	0.59865902	0.56410963	0.222820472
PPP1R3B	34.6960864	2.93886186	2.6811123	1.09613531	0.27301957	0.59865902	0.56380623	0.222820472
FOXK2	11.1910927	2.29177886	2.09173148	1.09563722	0.27323757	0.5988217	0.56345959	0.222702473
UBE2W	12.5481535	3.05304313	2.79264745	1.0932433	0.27428699	0.60050147	0.56179479	0.221485927
RPL24	103.082481	2.41932979	2.21319371	1.09313965	0.27433249	0.60050147	0.56172275	0.221485927
BCAT1	121.54026	3.15756516	2.8895851	1.09273998	0.27450798	0.60050147	0.56144503	0.221485927
H2AFV	77.9880351	2.55207319	2.33583554	1.092574	0.27458088	0.60050147	0.5613297	0.221485927
XPO7	54.9183826	1.78107116	1.63214318	1.09124688	0.27516426	0.60056045	0.56040797	0.221443272
COMMD3	11.0735549	2.78101913	2.55008839	1.09055794	0.27546745	0.60086247	0.55992972	0.221224924
DPH6	3.21024604	3.0371159	2.78876711	1.08905325	0.2761304	0.60199319	0.55888577	0.220408421
OXSR1	5.88061477	3.15439828	2.90002587	1.08771384	0.27672146	0.60238624	0.55795717	0.220124957
LEPR	34.254505	2.60315545	2.39334818	1.08766266	0.27674406	0.60238624	0.55792169	0.220124957
TPT1	32.6031256	1.67550584	1.54272816	1.0860668	0.27744944	0.60360474	0.55681615	0.219247361
MTMR2	6.52094802	2.68563393	2.47586787	1.08472425	0.2780438	0.60458193	0.55588678	0.218544839
ZNF148	13.0222443	2.29210131	2.11434071	1.08407377	0.27833209	0.60489291	0.55543672	0.218321502
CCDC141	4.70228367	3.11996103	2.88575809	1.0811582	0.27962675	0.60693767	0.55342129	0.216855905
SMC4	28.558612	2.25554992	2.08659707	1.08097052	0.27971023	0.60693767	0.55329165	0.216855905
TXLNA	68.0210893	2.46239718	2.28097019	1.07953939	0.28034734	0.60768679	0.55230356	0.216320201
CREB1	97.3684501	2.70445787	2.50706697	1.0787338	0.28070641	0.60794485	0.55174766	0.216135816
CFAP161	46.5563682	2.39535299	2.22076275	1.07861724	0.28075839	0.60794485	0.55166725	0.216135816
JMY	32.4101517	2.76833253	2.57014645	1.07711081	0.28143076	0.60896467	0.55062843	0.215407903
STK38	42.0127731	2.3020316	2.13763284	1.07690692	0.28152185	0.60896467	0.55048789	0.215407903
POU2F1	47.1664728	2.34169725	2.17564007	1.07632567	0.28178163	0.60921014	0.55008732	0.215232879
PCNT	493.732128	1.71457446	1.59376661	1.07580021	0.28201662	0.60940176	0.5497253	0.215096295
PDZD2	8.80776793	2.4555164	2.28333789	1.0754065	0.28219277	0.60946614	0.54945411	0.215050419
SLC25A3	99.0684256	2.57861943	2.40057296	1.07416832	0.28274725	0.61018605	0.54860161	0.214537726
TADA3	6.80816022	3.11984197	2.90522052	1.07387441	0.28287897	0.61018605	0.54839933	0.214537726
NBAS	6.332124	2.88527353	2.6919986	1.07179607	0.28381164	0.61071548	0.5469698	0.214161073
CNOT11	6.66333082	2.53123105	2.36233474	1.0714955	0.28394669	0.61071548	0.54676319	0.214161073
TFIP11	196.834682	2.92840379	2.73369773	1.07122443	0.28406853	0.61071548	0.54657688	0.214161073
DEPDC1B	5.25913986	2.29323205	2.14153236	1.07083698	0.28424273	0.61071548	0.54631063	0.214161073
ZDHHC2	10.1462365	2.75768963	2.57576	1.07063144	0.28433518	0.61071548	0.5461694	0.214161073
DNAJA1	3.45448743	3.12658225	2.92094638	1.07040042	0.28443911	0.61071548	0.54601069	0.214161073
CMYA5	5.23084629	1.89569653	1.77135837	1.07019368	0.28453214	0.61071548	0.54586867	0.214161073
ACTN4	83.9185557	1.8812436	1.75838546	1.06986986	0.28467789	0.61071548	0.54564626	0.214161073
ATP6V0A1	91.8885244	2.55751634	2.39185918	1.06925874	0.28495309	0.61099109	0.54522662	0.213965126
CNNM2	2.75904236	1.7625333	1.64956377	1.06848449	0.28530202	0.61110989	0.54469515	0.213880687
RAB31	40.0484469	2.3645463	2.21586873	1.06709674	0.28592815	0.6118216	0.54374308	0.213375194
TALDO1	266.543272	2.40836016	2.25964494	1.06581353	0.28650794	0.61274734	0.54286333	0.212718565
STAT5A	37.5518762	2.90423105	2.7385259	1.06050889	0.28891315	0.61666615	0.53923269	0.209949891
YTHDC1	96.355333	1.93579516	1.82541945	1.06046595	0.28893267	0.61666615	0.53920334	0.209949891
ZBTB16	168.522973	2.02602693	1.91534082	1.05778925	0.29015153	0.61859387	0.53737513	0.208594388
CCDC12	50.9081881	2.57360378	2.43365832	1.05750415	0.29028156	0.61859387	0.53718055	0.208594388
CNOT3	10.3112335	2.37503056	2.24743614	1.05677333	0.29061505	0.61898776	0.5366819	0.20831794
FKBP1A	82.0275012	2.83898562	2.6880124	1.05616537	0.29089267	0.6192623	0.53626723	0.208125355
MZF1	3.80925811	3.07251601	2.91748752	1.05313767	0.2922779	0.62113354	0.53420402	0.206815016
ZNF12	5.25293581	1.65921569	1.57568631	1.05301143	0.29233575	0.62113354	0.53411806	0.206815016
PARK7	4.48896793	2.61946747	2.48982047	1.05207082	0.29276706	0.62154437	0.53347779	0.206527864
RTFDC1	4.22910524	2.61014605	2.48143943	1.05186772	0.29286024	0.62154437	0.53333958	0.206527864
ING1	36.169176	2.10330713	2.00282226	1.05017164	0.29363921	0.62288011	0.53218596	0.205595536
ATF7IP	1518.08896	1.06618505	1.01814222	1.04718675	0.29501345	0.62510342	0.53015818	0.204048128
DCAF5	59.7967704	2.22092471	2.12139443	1.04691738	0.29513768	0.62510342	0.52997534	0.204048128
SOD2	87.2457038	1.8300817	1.74865165	1.04656733	0.29529917	0.62512748	0.52973777	0.204031406
SLC8A3	33.1282137	1.99061424	1.90736908	1.04364397	0.29665014	0.62671293	0.52775544	0.202931344
HIST1H2BF	146.802964	2.11863603	2.03245839	1.04240069	0.29722595	0.62733402	0.52691328	0.202501163
GNAI3	39.0472544	1.99973658	1.91901482	1.04206417	0.29738193	0.62733402	0.52668542	0.202501163
EPM2AIP1	78.6153381	2.43855737	2.34049498	1.04189814	0.2974589	0.62733402	0.52657303	0.202501163
CHIC2	4.479041	2.21425702	2.12885615	1.04011585	0.29828608	0.62847558	0.52536701	0.201711594
X11.Sep	166.727746	1.39329081	1.3400664	1.03971774	0.29847105	0.62847558	0.52509778	0.201711594
TARS	16.1940857	2.97199561	2.85972417	1.03925953	0.29868405	0.62847558	0.52478797	0.201711594

USP12	53.0122116	2.27824534	2.19268257	1.03902196	0.29879452	0.62847558	0.52462737	0.201711594
NDUFA11	62.6775571	2.44883206	2.36288063	1.0363757	0.3000269	0.63032898	0.5228398	0.200432729
CYTH2	17.2698469	2.28546257	2.20644646	1.03581148	0.3002901	0.63038016	0.52245898	0.200397462
KIAA1551	477.965155	2.59145993	2.50219547	1.03567445	0.30035404	0.63038016	0.52236652	0.200397462
FLAD1	4.81785731	3.01346406	2.91105666	1.03517877	0.30058543	0.63054798	0.52203207	0.200281863
L3MBTL1	92.07595	2.28104392	2.20622559	1.03391237	0.30117713	0.63131663	0.521178	0.199752774
ZNF460	6.06673943	3.00291508	2.90488769	1.03374567	0.30125508	0.63131663	0.52106563	0.199752774
SKP1	122.13653	1.73026678	1.67665885	1.03197307	0.30208473	0.63273683	0.51987122	0.198776883
RPS6KA5	6.22392792	1.5234095	1.4777308	1.03091138	0.30258238	0.63346055	0.51915637	0.198280428
ARPC4	35.8309336	2.63110022	2.55585895	1.02943874	0.30327355	0.63416689	0.51816546	0.197796439
MPP5	30.6029797	1.98959379	1.9331098	1.02921923	0.30337667	0.63416689	0.51801783	0.197796439
AKT3	18.1650558	2.26519609	2.20195547	1.02872021	0.30361117	0.63433865	0.51768225	0.197678829
CNTD1	6.80918189	2.7694807	2.69537258	1.02749457	0.30418764	0.63522434	0.51685844	0.197072871
CLOCK	62.2084829	2.56529586	2.49956391	1.02629737	0.30475144	0.63579097	0.51605424	0.196685647
HMGXB3	13.3853531	2.86444425	2.79139289	1.02617022	0.30481136	0.63579097	0.51596886	0.196685647
SELENOK	34.8456621	2.63509278	2.5695463	1.02550897	0.30512309	0.63590219	0.51552492	0.196609682
MRPL9	13.6192104	2.49257533	2.43227469	1.02479187	0.3054614	0.63628879	0.51504366	0.196345731
CLK3	179.828932	1.88017854	1.83555093	1.02431293	0.30568749	0.63644136	0.51472233	0.196241602
RPS16	92.0271566	2.54436464	2.49369488	1.02031915	0.30757712	0.63973584	0.51204597	0.193999321
EPG5	131.251541	1.98025275	1.94324634	1.0190436	0.30818227	0.64035477	0.51119236	0.193579352
RBMXL1	30.0394999	2.57001859	2.52542564	1.0176576	0.30884071	0.6412236	0.51026546	0.192990504
NUDT22	14.9715863	2.64909462	2.60349382	1.01751523	0.30890839	0.6412236	0.5101703	0.192990504
DCAF6	4.03624425	2.96061199	2.91462397	1.01577837	0.30973494	0.64202163	0.5090098	0.192450343
IGF2BP2	177.420858	1.65955073	1.63851003	1.01284137	0.31113594	0.64409807	0.50704982	0.191048003
ICE2	8.48519819	2.7660222	2.73143462	1.01266279	0.31122126	0.64409807	0.50693075	0.191048003
SRSF5	380.520538	1.69230674	1.67446329	1.01065622	0.312181	0.64544274	0.50559354	0.190142282
RBX1	362.004685	2.24101437	2.22381061	1.00773616	0.31358114	0.64801582	0.50365006	0.188414393
RPS26	14.5307836	2.14257361	2.12823573	1.00673698	0.31406119	0.64864227	0.50298573	0.187994756
PPP1R21	3.51845667	2.65170939	2.6346974	1.0064569	0.31419583	0.64864227	0.50279958	0.187994756
TRIM69	3.53712011	2.00237178	1.99478973	1.00380092	0.31547458	0.65055695	0.50103563	0.186714682
GLYR1	24.313355	2.53106129	2.52373265	1.00290389	0.31590723	0.65055695	0.50044043	0.186714682
AGBL5	70.7519962	2.28580629	2.27922922	1.00288566	0.31591603	0.65055695	0.50042833	0.186714682
CCDC93	6.53042563	2.74867832	2.74147031	1.00262925	0.31603978	0.65055695	0.50025825	0.186714682
FEM1B	7.97390766	2.4695283	2.46315874	1.00258593	0.31606069	0.65055695	0.50022952	0.186714682
TUBGCP4	32.5874177	2.11464806	2.11797637	0.99842854	0.3180716	0.65323631	0.49747511	0.184929683
NDUFC1	4.474874	2.85983009	2.87605888	0.99435728	0.32004896	0.65540293	0.49478358	0.183491619
EPRS	6.35069321	2.8873746	2.90491952	0.99396027	0.32024221	0.65540293	0.49452143	0.183491619
LARP1	253.68326	1.67051957	1.68159072	0.99341626	0.32050714	0.65540293	0.49416229	0.183491619
PCTP	6.91529565	2.88433615	2.90357244	0.99337496	0.32052726	0.65540293	0.49413503	0.183491619
UNC13D	1016.396	1.02513829	1.03245859	0.99290984	0.3207539	0.65540293	0.49382806	0.183491619
LRCH4	8.38358319	1.35529451	1.36551093	0.99251825	0.32094478	0.65540293	0.49356968	0.183491619
ATP6V0B	241.706243	2.61117321	2.63198618	0.9920923	0.3211525	0.65540293	0.49328869	0.183491619
NRXN2	3.36401106	1.84497403	1.85997395	0.99193542	0.32122903	0.65540293	0.49318521	0.183491619
DAPK3	35.2637897	1.58028886	1.59480152	0.99090002	0.3217344	0.65607348	0.4925025	0.183047516
UGGT2	9.95910675	2.00336062	2.02588596	0.98888124	0.32272125	0.65744192	0.49117243	0.182142605
RPL32	75.5069014	2.45903503	2.48774171	0.98846075	0.32292705	0.65753948	0.49089558	0.182078167
RICTOR	121.410171	1.9044452	1.93070469	0.98639901	0.32393735	0.65927425	0.48953898	0.180933885
DCAF11	191.081661	2.53357806	2.56976832	0.98591692	0.32417388	0.65943334	0.48922197	0.180829097
BPTF	215.187081	1.44257311	1.4641654	0.98525283	0.32449989	0.6594522	0.48878544	0.180816678
NAA35	5.78037396	2.85792203	2.90628246	0.98336004	0.32543027	0.66069802	0.48754205	0.179996992
NBR1	43.2521774	2.26809923	2.30782903	0.98278477	0.32571338	0.66095054	0.4871644	0.179831039
RPS2	7.79929456	2.8486671	2.90168371	0.98172902	0.32623336	0.66168326	0.48647163	0.179349854
PFDN5	78.6097059	2.60834	2.65780235	0.98138975	0.32640058	0.6617001	0.48624908	0.179338801
HIBCH	8.64974125	2.17354339	2.22018076	0.97899388	0.327583	0.66280642	0.48467864	0.178613292
EIF2AK1	239.153893	1.69201496	1.73050909	0.9777556	0.32819521	0.66372262	0.48386776	0.178013383
BCL11A	19.3700114	2.79013573	2.85776703	0.97633421	0.32889887	0.66425587	0.48293762	0.177664599
ASTN2	2.95700836	2.22626039	2.28496708	0.97430742	0.32990392	0.66556198	0.48161252	0.176811496
KLHL24	207.929171	1.45793803	1.49748953	0.97358814	0.33026108	0.66579639	0.4811426	0.176658567
RPS3	97.4452175	2.14845045	2.20737716	0.97330465	0.33040191	0.66579639	0.48095745	0.176658567
DAZAP1	5.85462999	2.13839703	2.19749272	0.97310767	0.33049979	0.66579639	0.48082881	0.176658567
PRKD3	3.97595068	2.75634083	2.83774934	0.9713123	0.33139279	0.66644576	0.47965695	0.176235189
LSM12	8.67623353	2.48264241	2.55633491	0.9711726	0.33146234	0.66644576	0.47956581	0.176235189

TTC3	41.1334741	2.097754	2.16099912	0.97073339	0.33168106	0.66656367	0.47927933	0.17615836
MAPK14	26.6453969	1.94087375	2.00021815	0.97033104	0.33188151	0.66664477	0.47901694	0.176105523
SOX6	6.67593036	1.95363878	2.01458123	0.96974932	0.33217146	0.66690547	0.47863769	0.175935717
CABLES1	48.5982823	2.41345472	2.49453161	0.96749815	0.33329506	0.66851669	0.47717112	0.174887745
RPS19	149.202654	1.95408213	2.02263639	0.96610649	0.3339909	0.66958984	0.47626537	0.174191142
RPS13	197.866095	2.31351005	2.39883301	0.96443147	0.33482965	0.67062562	0.47517609	0.173519858
NCOA6	108.438802	1.75694824	1.82925419	0.96047244	0.33681749	0.67368063	0.47260536	0.171545938
GATAD2A	27.6547513	1.73492042	1.80711787	0.96004829	0.33703091	0.67368063	0.47233026	0.171545938
EIF5A	241.264269	2.11449188	2.20309119	0.95978409	0.33716389	0.67368063	0.47215894	0.171545938
UBE3C	4.7231031	2.08228714	2.17254405	0.95845566	0.33783304	0.67424574	0.47129788	0.171181786
NBPF14	19.3515243	1.81441017	1.89340897	0.95827695	0.33792312	0.67424574	0.47118209	0.171181786
ABCC5	57.189173	2.37982262	2.48348741	0.95825838	0.33793249	0.67424574	0.47117006	0.171181786
LTB	12.9662391	2.05178491	2.14766423	0.95535647	0.33939746	0.67684436	0.46929141	0.169511185
KLHL18	3.61024158	2.78252712	2.91584062	0.95427957	0.33994215	0.67729589	0.46859499	0.169221558
NAPA	12.2094908	2.00855199	2.10481429	0.95426566	0.33994919	0.67729589	0.46858599	0.169221558
CAPNS1	4.16404569	2.22438893	2.33428946	0.95291906	0.34063108	0.6783299	0.46771573	0.16855904
WDSUB1	8.90897785	2.76037968	2.89969604	0.95195484	0.34111989	0.67846037	0.46709296	0.168475513
USP3	77.1250132	2.05308031	2.15845827	0.95117906	0.34151349	0.67846037	0.46659214	0.168475513
SPICE1	7.89870234	1.98861552	2.09090141	0.95108048	0.34156352	0.67846037	0.46652852	0.168475513
GLA	29.7102889	2.44345925	2.57135084	0.95026288	0.3419787	0.67846037	0.46600095	0.168475513
NPEPPS	25.4500806	1.33749025	1.40755735	0.95022079	0.34200008	0.67846037	0.4659738	0.168475513
HOOK2	11.6466475	2.46730629	2.5974629	0.94989087	0.34216771	0.67846968	0.46576098	0.168469556
PMPCB	3.317694	2.76718879	2.91828176	0.94822537	0.34301474	0.6795764	0.46468722	0.16776171
PCNP	229.491398	2.22915002	2.3549115	0.94659609	0.34384464	0.68082219	0.46363774	0.166966297
H2AFJ	454.033007	1.41055959	1.49073236	0.9462192	0.34403679	0.6808789	0.46339511	0.166930122
CTDSP2	247.879721	1.75253688	1.85326171	0.94564997	0.34432715	0.68112981	0.46302874	0.166770115
ROCK1	70.0307339	2.20129712	2.33027788	0.94465005	0.34483757	0.68181558	0.46238543	0.166333076
AVL9	69.5204502	2.25284153	2.38683172	0.94386274	0.3452398	0.68196324	0.46187915	0.166239035
HK1	402.24926	1.30946908	1.38791654	0.94347826	0.34543633	0.68202792	0.46163199	0.166197848
RPL35	8.89465049	2.08248393	2.2118717	0.94150304	0.34644713	0.68337558	0.46036303	0.165340544
HECTD1	5.71823397	2.7140048	2.88964065	0.93921879	0.34761842	0.6847084	0.45889722	0.164494347
JAK3	207.949239	1.89887905	2.02246371	0.93889401	0.34778517	0.6847084	0.45868895	0.164494347
PLEKHG2	26.6712628	2.11726776	2.25720153	0.93800564	0.34824151	0.68496819	0.45811946	0.164329598
SYMPK	10.6944889	1.46087693	1.55839839	0.937422	0.34854153	0.68523462	0.45774547	0.164160704
SMAD2	59.2831535	1.43119565	1.52780577	0.93676544	0.34887923	0.68537114	0.45732489	0.16407419
PPOX	18.7440664	2.64965151	2.82931332	0.93649986	0.34901589	0.68537114	0.4571548	0.16407419
DNAJA2	9.07999847	2.14080101	2.28638131	0.9363272	0.34910475	0.68537114	0.45704424	0.16407419
PDLIM1	124.814645	1.27651994	1.3653983	0.93490664	0.34983641	0.68641145	0.45613499	0.163415483
SENP6	3.53182156	2.45603411	2.63286236	0.93283802	0.35090361	0.68760594	0.45481217	0.162660379
CRKL	226.821987	2.14309259	2.3001177	0.93173171	0.3514752	0.68820484	0.45410532	0.162282276
PRKDC	2.93551984	2.6283749	2.82187164	0.93142965	0.35163136	0.68820484	0.4539124	0.162282276
NDUFV2	132.462558	2.08264486	2.23885382	0.93022816	0.35225297	0.68820484	0.45314534	0.162282276
FRYL	213.410964	1.41454688	1.52142239	0.9297529	0.35249904	0.68820484	0.45284206	0.162282276
VPS53	22.7573229	1.81793881	1.95556573	0.92962296	0.35256633	0.68820484	0.45275916	0.162282276
SUPT5H	100.759913	1.68089075	1.80825781	0.92956366	0.35259705	0.68820484	0.45272133	0.162282276
PIGG	3.37700556	2.47810781	2.6663234	0.92941007	0.35267661	0.68820484	0.45262334	0.162282276
HIST1H4J	6.44288244	1.67056199	1.79813561	0.92905228	0.35286199	0.68820484	0.45239512	0.162282276
CD47	457.464418	1.18011097	1.27171019	0.92797162	0.35342229	0.68865251	0.45170606	0.161999866
IFNGR1	85.6125931	2.0661961	2.22923912	0.92686158	0.3539984	0.68940283	0.45099869	0.161526938
ARPC2	190.348571	1.69608885	1.83045949	0.92659185	0.35413849	0.68940283	0.45082687	0.161526938
NFKBIB	3.41920617	1.83187108	1.98002649	0.92517504	0.35487487	0.69051352	0.44992476	0.160827811
LPCAT4	42.9844424	2.05508282	2.22215588	0.92481488	0.35506221	0.69055537	0.44969555	0.160801494
RUFY2	17.633231	1.79149152	1.93886567	0.9239895	0.35549178	0.69106806	0.44917043	0.160479178
STK17A	14.2642667	2.67310198	2.89535521	0.92323801	0.35588318	0.69116528	0.44869253	0.160418087
CPT2	17.2235201	2.30361497	2.49745747	0.92238407	0.35632828	0.69116528	0.44814971	0.160418087
RALGDS	190.964838	1.73433016	1.88084078	0.92210366	0.35647451	0.69116528	0.44797152	0.160418087
ECH1	75.2392208	2.36609396	2.56766829	0.92149518	0.35679196	0.69116528	0.44758495	0.160418087
HUWE1	332.093239	1.585089	1.7203598	0.92137064	0.35685695	0.69116528	0.44750584	0.160418087
FOXO3	109.130377	1.59733052	1.73369189	0.92134625	0.35686968	0.69116528	0.44749035	0.160418087
TCP1	14.0542912	2.42966279	2.63950284	0.92050016	0.35731145	0.69169915	0.44695307	0.160082757
ARIH2	271.439072	2.15097787	2.33855816	0.91978806	0.35768353	0.69209768	0.44650106	0.159832608
WASF2	109.710754	1.18962301	1.29573376	0.9181076	0.35856254	0.69347627	0.44543509	0.158968396

CCDC82	4.12209182	1.8294143	1.99473438	0.91712176	0.35907884	0.6938303	0.44481018	0.158746738
C3orf58	26.1099807	2.03707383	2.22225646	0.9166691	0.35931606	0.69385535	0.44452337	0.158731056
HARBI1	3.16640613	2.22767695	2.43073799	0.91646116	0.35942507	0.69385535	0.44439163	0.158731056
TSC2	18.5508112	1.40049846	1.52876506	0.9160979	0.35961555	0.69390136	0.44416154	0.158702261
CTNNBL1	142.417313	2.21089445	2.41623426	0.9150166	0.36018292	0.69437058	0.44347689	0.158408691
PHF3	42.2868294	1.97118991	2.15497268	0.91471689	0.36034028	0.69437058	0.44328719	0.158408691
FAM120B	179.786384	1.91745429	2.09703663	0.91436375	0.36052574	0.69437058	0.44306372	0.158408691
TRAK2	68.7651843	1.38278671	1.51427992	0.91316453	0.36115602	0.6952629	0.44230515	0.157850945
KIF11	10.0287895	1.91545493	2.10003104	0.91210791	0.36171191	0.69573739	0.44163719	0.157554658
CSNK1A1	76.7983651	1.65154378	1.81246643	0.91121344	0.36218292	0.69595279	0.44107203	0.157420222
HMGA1	3.86631648	2.27774536	2.50198968	0.9103736	0.36262551	0.69595747	0.44054164	0.1574173
TRPM4	9.19725075	2.41044884	2.6477839	0.91036464	0.36263024	0.69595747	0.44053598	0.1574173
ARHGEF12	838.854159	1.01120524	1.1109003	0.91025742	0.36268677	0.69595747	0.44046829	0.1574173
UGGT1	3.32211358	1.84006642	2.02499148	0.9086786	0.3635198	0.69691366	0.43947192	0.156821025
FAM213B	35.8696906	1.95437559	2.15263834	0.90789779	0.36393223	0.69706246	0.43897948	0.156728303
NCOA3	117.692545	1.70900427	1.88507371	0.90659811	0.36461936	0.69800894	0.43816028	0.156139018
NADK2	3.88738299	1.82782454	2.01673359	0.9063292	0.36476163	0.69800894	0.43799085	0.156139018
TRIM33	68.6763138	1.97166042	2.17657681	0.90585382	0.36501323	0.69816954	0.4376914	0.156039104
WDFY2	39.8552035	2.20287156	2.43342139	0.90525692	0.36532929	0.6982144	0.4373155	0.156011197
SET	83.1215338	1.98146222	2.18903479	0.90517621	0.36537204	0.6982144	0.43726469	0.156011197
CMAS	325.768382	2.00062876	2.2126515	0.90417707	0.36590151	0.69890546	0.43663579	0.155581566
INPP5K	214.294273	2.07867125	2.3025928	0.90275243	0.3666573	0.70002797	0.43573966	0.154884606
SEC61A1	36.2128103	2.30620546	2.55734037	0.9017984	0.36716397	0.70035792	0.43513994	0.154679954
ASXL1	34.1996661	2.3543867	2.61078217	0.90179362	0.36716651	0.70035792	0.43513694	0.154679954
RPS28	67.4576906	1.96377715	2.18198256	0.89999672	0.368122	0.70147019	0.43400823	0.153990783
BCL2L1	95.5350227	1.92062997	2.1346338	0.89974682	0.368255	0.70147019	0.43385135	0.153990783
DYNLL1	37.696679	1.9965734	2.22171251	0.89866416	0.36883158	0.70224722	0.43317191	0.153509969
FER	26.3777506	1.98987535	2.22807286	0.89309258	0.37180759	0.7060767	0.42968174	0.151148121
C11orf24	6.30953014	2.09985975	2.35148196	0.8929942	0.37186028	0.7060767	0.42962021	0.151148121
LANCL3	64.3805144	1.89845917	2.13538579	0.8890474	0.37397761	0.70945	0.4271544	0.149078204
ARL8B	252.592503	2.15707784	2.43165613	0.88708178	0.37503487	0.71080802	0.42592835	0.148247682
CEP85L	5.65445769	2.10736287	2.37664716	0.88669572	0.37524274	0.71087843	0.4256877	0.148204664
SP3	22.2650704	2.04383192	2.30585578	0.8863659	0.37542039	0.71089154	0.42548214	0.148196652
WDR44	70.4314014	1.48062002	1.67247902	0.88528466	0.37600312	0.71098124	0.42480855	0.148141857
ELK4	3.51181412	2.17992295	2.46429524	0.88460299	0.37637079	0.7110744	0.42438409	0.148084957
FHL2	10.2736725	2.1735443	2.45937125	0.88378048	0.37681473	0.71129822	0.42387213	0.147948281
GLUL	36.94679	1.75377258	1.98517623	0.8834342	0.37700172	0.71129822	0.42365667	0.147948281
INTS11	5.80637882	2.14537268	2.43512851	0.88101004	0.37831238	0.71300883	0.42214944	0.146905089
EIF5B	16.2655213	2.32758952	2.64256355	0.8808074	0.37842208	0.71300883	0.42202354	0.146905089
CCNT2	134.71328	2.11230481	2.40464856	0.87842558	0.37971281	0.71479391	0.42054475	0.145819156
CHD6	5.91332301	1.07035518	1.2190756	0.87800558	0.37994069	0.7148997	0.42028419	0.145754883
DIDO1	213.993192	1.70684717	1.95111096	0.87480784	0.38167847	0.71719727	0.41830234	0.144361372
PCYT1B	3.13923168	1.88495099	2.15840091	0.87330902	0.38249466	0.71840676	0.41737462	0.143629592
CDKN1B	3.18552428	2.02503688	2.32071249	0.87259274	0.3828851	0.71881584	0.41693154	0.143382359
VMP1	97.6077632	1.55107417	1.77856786	0.87209165	0.38315838	0.71900474	0.41662167	0.143268249
SBNO2	2.90515725	1.45631267	1.67061971	0.87172003	0.38336113	0.71906115	0.41639192	0.143234177
NBEA	4.39662807	1.86709469	2.15018779	0.86834029	0.38520807	0.72155034	0.41430462	0.141733363
RAP1A	28.5951592	1.41150304	1.62924032	0.86635656	0.38629466	0.72326032	0.4130813	0.140705359
PIGN	14.6517007	1.79512133	2.07343598	0.86577128	0.3866156	0.72353589	0.41272063	0.14053992
FOXP1	177.219741	1.35592784	1.5689033	0.86425202	0.38744946	0.72477069	0.41178494	0.139799378
RPL9	15.9478584	2.11758889	2.45457945	0.86270945	0.38829724	0.72570453	0.4108357	0.139240167
LRP10	22.4611911	2.18241526	2.53780682	0.85996114	0.38981046	0.7278957	0.40914651	0.137930848
JAK2	325.785543	1.49695468	1.74196072	0.85935042	0.39014721	0.7278957	0.40877149	0.137930848
FAU	148.32562	1.95959551	2.28042603	0.85931115	0.39016888	0.7278957	0.40874738	0.137930848
INO80D	235.679361	1.78604207	2.07940163	0.85892117	0.39038401	0.7279709	0.40850798	0.137885981
SKIL	4.29058959	1.38848878	1.61900478	0.8576187	0.39110304	0.72872268	0.4077088	0.137437715
CFAP44	3.31954012	1.73353855	2.02297944	0.85692347	0.39148718	0.72872268	0.40728245	0.137437715
HSPB1	92.831356	1.82627331	2.13492528	0.85542727	0.39231465	0.72961063	0.40636547	0.136908845
MAEA	85.4142184	2.11123899	2.48315432	0.85022464	0.3952002	0.73366636	0.40318284	0.134501393
ABR	68.8528883	1.7641853	2.07702205	0.84938208	0.39566872	0.73388523	0.40266828	0.134371855
CALCOCO1	272.82305	1.59324075	1.87577192	0.84937872	0.39567059	0.73388523	0.40266623	0.134371855
ZRANB2	41.9442738	2.3085227	2.72077421	0.84848007	0.39617067	0.73424563	0.40211768	0.134158629

DNAJC24	2.98749341	2.22083615	2.61768866	0.84839583	0.39621756	0.73424563	0.40206628	0.134158629
AHSA1	24.6466446	2.45253098	2.8922742	0.84795936	0.3964606	0.7343692	0.40179996	0.134085549
NDUFB4	67.8760025	1.84888594	2.18368414	0.84668195	0.39717242	0.73484017	0.40102091	0.133807108
CHD9	233.392399	1.14092381	1.34823264	0.8462366	0.39742077	0.73484017	0.40074944	0.133807108
RNF10	68.6291911	1.70792362	2.02075896	0.84518918	0.39800522	0.73536075	0.40011123	0.133499554
EPOR	320.095038	1.66922319	1.9763503	0.84459885	0.39833485	0.73536075	0.3997517	0.133499554
GTPBP8	14.4832326	1.9260601	2.28161448	0.84416545	0.39857696	0.73536075	0.39948781	0.133499554
RBM33	142.808347	1.64112111	1.94556616	0.84351853	0.39893851	0.73536075	0.39909404	0.133499554
NOTCH2NL	2.82540271	1.6235962	1.92614182	0.84292661	0.39926949	0.7356452	0.39873388	0.133331594
NAALADL2	3.7112315	2.23967864	2.6619534	0.84136658	0.4001426	0.73660203	0.39778521	0.132767089
FAXDC2	366.982367	1.66178442	1.97824987	0.84002756	0.40089293	0.73749037	0.3969716	0.132243647
BCCIP	57.6976339	2.42287503	2.89018159	0.83831239	0.40185528	0.73846165	0.39593032	0.131672056
TMEM164	74.538566	1.70768658	2.03832144	0.83779062	0.40214831	0.73846165	0.39561375	0.131672056
BTF3	45.9493267	1.80497867	2.15476466	0.83766859	0.40221686	0.73846165	0.39553973	0.131672056
SAR1A	132.806408	1.90277651	2.28121218	0.83410764	0.40422033	0.74087059	0.39338185	0.130257647
LRIG2	39.1404663	1.82476198	2.19013994	0.83317141	0.40474807	0.74087059	0.39281522	0.130257647
PIP4K2A	336.047988	1.15495412	1.38629198	0.83312472	0.4047744	0.74087059	0.39278697	0.130257647
C2orf68	138.970786	1.30863724	1.57211396	0.8324061	0.40517976	0.7412867	0.39235225	0.130013791
MKNK2	52.0185222	1.80839476	2.17627958	0.830957	0.40599792	0.74237058	0.39147619	0.129379245
MINDY1	17.5242968	1.97849981	2.38165318	0.83072541	0.40612877	0.74237058	0.39133625	0.129379245
MYZAP	129.402941	1.72601575	2.07958543	0.82998069	0.4065497	0.74262799	0.39088635	0.129228684
ZFX	58.8390355	2.17044452	2.6159347	0.82970134	0.40670767	0.74262799	0.39071764	0.129228684
DPCD	5.65834439	2.2526555	2.7180994	0.82876126	0.40723951	0.74309611	0.39015009	0.128955012
DUSP28	3.50357732	2.40624642	2.91354841	0.82588174	0.40887118	0.74574665	0.3884135	0.127408691
CD58	81.3092001	1.93084919	2.34006859	0.82512504	0.40930061	0.74620303	0.38795761	0.127142992
NEXN	122.558527	1.38703985	1.68256094	0.82436233	0.40973371	0.74666573	0.3874983	0.126873783
NCOR2	44.8731768	1.38054309	1.67838541	0.82254236	0.41076829	0.74789644	0.38640309	0.126158534
ZNF100	9.61222414	2.15227223	2.61945917	0.82164756	0.41127751	0.74816931	0.38586504	0.126000108
SMNDC1	5.18361856	1.82147408	2.22176117	0.81983343	0.41231107	0.74923246	0.384775	0.125383415
DMPK	29.7912907	2.11609635	2.58348476	0.81908606	0.41273731	0.74951512	0.38432627	0.1252196
ACTR1A	73.1233855	1.92059155	2.34593891	0.81868779	0.41296456	0.74960089	0.38408721	0.125169907
INTS6L	14.8986979	2.19313558	2.68048588	0.81818584	0.41325108	0.74979412	0.383786	0.12505797
DPY19L1	3.15106582	2.31512031	2.83106278	0.81775661	0.41349618	0.74991205	0.3835285	0.124989665
F2R	91.1546647	1.52054026	1.86154867	0.81681467	0.41403435	0.75056118	0.38296363	0.124613903
CRK	49.3631467	2.24795175	2.75370792	0.81633631	0.41430781	0.75073009	0.38267687	0.124516178
C19orf25	5.98585775	1.69462133	2.07948383	0.81492402	0.4151158	0.75135354	0.38183074	0.124155664
DIMT1	5.17786827	1.67268219	2.05321684	0.81466417	0.41526457	0.75135354	0.38167512	0.124155664
ECD	39.3546136	1.50527535	1.85100491	0.81322062	0.41609158	0.75205759	0.38081107	0.123748902
RPL18	135.88415	1.71385016	2.10844864	0.8128489	0.41630469	0.75205759	0.38058869	0.123748902
DAGLB	165.272675	1.57895162	1.94597645	0.81139297	0.41714003	0.75291335	0.37971813	0.123255003
ZNF445	3.42010967	1.26810582	1.56641606	0.80955875	0.41819383	0.75447922	0.37862238	0.12235272
LEPROT	125.725103	1.61446169	1.99758526	0.80820665	0.41897163	0.75447922	0.37781538	0.12235272
CCDC85B	3.83213478	2.15703954	2.66969759	0.80797149	0.41910699	0.75447922	0.37767509	0.12235272
ELMO1	51.8370736	1.49813026	1.85486056	0.8076781	0.41927591	0.75447922	0.37750009	0.12235272
ARHGAP4	2.77697813	1.9915471	2.46989984	0.80632707	0.42005429	0.75555337	0.37669458	0.121734854
SF3B6	44.3396267	2.32841399	2.89059049	0.805515	0.42052256	0.75606905	0.37621071	0.121438539
RAB1A	110.409228	1.78721626	2.22356835	0.80376043	0.42153534	0.75756287	0.37516601	0.120581318
SSR4	243.776446	1.60183441	1.99487154	0.80297622	0.42198848	0.75805005	0.37469941	0.120302118
OST4	252.056515	1.40556476	1.75256681	0.80200353	0.42255092	0.75815071	0.37412095	0.120244456
PRELID3B	59.8938881	2.10645332	2.62679562	0.80190987	0.4226051	0.75815071	0.37406527	0.120244456
NR4A2	3.43584522	1.52666994	1.90767728	0.80027684	0.42355042	0.75921823	0.37309488	0.119633373
RBM4B	70.2711763	2.15410308	2.69420315	0.79953254	0.42398169	0.75966426	0.3726529	0.119378303
STIM2	47.9276505	2.0793798	2.60647252	0.79777545	0.42500081	0.76116274	0.37161024	0.118522481
STAMBP	14.0887267	2.12537741	2.66779196	0.79668034	0.4256367	0.76134874	0.37096093	0.118416365
PAQR3	7.28761786	2.02917505	2.55044988	0.79561456	0.4262561	0.76185993	0.37032939	0.118124865
MBNL1	480.588973	0.93497794	1.17621201	0.79490596	0.42666821	0.76185993	0.36990972	0.118124865
GRAMD1A	42.9977735	1.93100602	2.43024268	0.79457333	0.42686174	0.7618741	0.36971277	0.118116791
GRK2	107.604608	1.61287648	2.03337166	0.79320299	0.42765956	0.76286978	0.36890181	0.117549587
PGRMC2	20.0130362	2.10596936	2.66168186	0.79121753	0.42881706	0.76306187	0.36772794	0.117440249
SLC12A7	72.3173247	1.93943625	2.45186484	0.79100455	0.42894134	0.76306187	0.3676021	0.117440249
ANAPC5	132.292042	1.83867281	2.32493361	0.7908496	0.42903176	0.76306187	0.36751055	0.117440249
ANKS1A	51.0444528	2.06766965	2.61523073	0.79062609	0.42916222	0.76306187	0.36737852	0.117440249

NPIPB2	3.10009732	0.96734473	1.22733733	0.78816533	0.43060001	0.76379925	0.36592597	0.117020773
CCDC117	4.33043413	1.97629981	2.5076382	0.78811202	0.43063118	0.76379925	0.36589453	0.117020773
CACNB1	109.551508	1.78916273	2.27055745	0.78798391	0.43070612	0.76379925	0.36581896	0.117020773
GPM6B	14.402907	2.06440582	2.62161893	0.78745457	0.43101581	0.76379925	0.3655068	0.117020773
EMSY	81.0641053	1.64485051	2.08884015	0.78744681	0.43102035	0.76379925	0.36550222	0.117020773
ZER1	53.4431195	1.60207649	2.03468937	0.78738136	0.43105865	0.76379925	0.36546363	0.117020773
PPP1R9B	4.86787913	1.62898714	2.0712959	0.78645796	0.43159924	0.76421703	0.36491933	0.116783288
ING3	5.25980457	1.65164169	2.10148137	0.78594163	0.43190169	0.76421703	0.3646151	0.116783288
IRF3	18.5596729	2.24630682	2.85865277	0.78579212	0.43198928	0.76421703	0.36452703	0.116783288
IGF1R	183.090194	1.21206488	1.54260679	0.7857251	0.43202855	0.76421703	0.36448755	0.116783288
WDR43	31.7784629	1.69059611	2.15536735	0.78436565	0.43282559	0.76437796	0.36368707	0.116691844
PUM2	188.482646	1.88492302	2.40326512	0.78431755	0.4328538	0.76437796	0.36365876	0.116691844
GRAP2	369.781308	1.21275155	1.54700612	0.78393455	0.43307852	0.76445059	0.36343336	0.116650581
KLHL6	202.44078	1.11102239	1.42019924	0.78230037	0.43403807	0.76513083	0.36247218	0.1162643
UBE2B	62.4116303	1.41728325	1.81232079	0.7820267	0.43419888	0.76513083	0.3623113	0.1162643
TSTA3	10.3999301	2.1994653	2.8141453	0.78157489	0.43446445	0.76527494	0.36204575	0.116182506
EIF4A1	7.97153509	2.25123504	2.89924516	0.77649006	0.43745969	0.76957421	0.35906196	0.113749497
CTDP1	8.20682062	2.18484369	2.81544939	0.77601952	0.43773746	0.76973766	0.35878629	0.113657265
GATA2	82.4518422	1.16324041	1.50081384	0.77507309	0.43829647	0.77039532	0.35823202	0.113286363
NBEAL2	948.332121	1.04592521	1.35118944	0.7740774	0.43888502	0.77077909	0.35764924	0.113070075
PI4KA	142.768515	1.46236003	1.8921595	0.77285241	0.43960973	0.77140115	0.3569327	0.11271972
NR3C1	8.88921846	1.72404266	2.23412212	0.77168685	0.44029992	0.7722868	0.35625139	0.112221387
TMBIM6	71.0949423	1.95707145	2.53801543	0.77110306	0.44064585	0.77256813	0.35591032	0.11206321
VPS36	8.54475584	2.23290322	2.89847869	0.77037076	0.44108	0.77268095	0.35548264	0.111999794
DNAAF2	10.2659872	1.84512638	2.3995783	0.76893777	0.44193026	0.77268095	0.35464626	0.111999794
DUSP1	25.446439	1.94862789	2.53653415	0.76822458	0.44235377	0.77268095	0.35423027	0.111999794
LUC7L	89.1166419	1.59931767	2.08772306	0.76605834	0.44364159	0.77383467	0.35296775	0.111351817
EPB41	289.129845	1.02750985	1.34171412	0.76581876	0.44378415	0.77383467	0.35282822	0.111351817
CRBN	190.697032	1.44942094	1.89531203	0.76474001	0.44442638	0.77463015	0.35220017	0.110905602
BAZ2A	177.718734	1.0423775	1.36902466	0.76140155	0.44641726	0.77678384	0.35025902	0.10969982
TBC1D32	5.92222766	1.68335234	2.21104203	0.76133892	0.44645466	0.77678384	0.35022264	0.10969982
MPP1	487.975564	1.17855567	1.54984242	0.7604358	0.44699413	0.77678384	0.34969818	0.10969982
YEATS2	43.0201048	1.78680427	2.35172594	0.75978423	0.44738357	0.77678384	0.34931997	0.10969982
HDGF	7.07476979	2.19962009	2.90037325	0.75839208	0.4482163	0.77681423	0.34851235	0.109682828
KDM6A	54.9581041	1.71618406	2.26318948	0.75830331	0.44826943	0.77681423	0.34846088	0.109682828
ANKRD12	167.770059	0.91119241	1.20242412	0.75779619	0.44857301	0.77681423	0.34816686	0.109682828
EEF2	61.4207605	1.79256323	2.36659293	0.75744468	0.4487835	0.77681423	0.34796312	0.109682828
STXBP2	626.99454	0.76842784	1.01511325	0.75698731	0.44905747	0.77681423	0.34769808	0.109682828
LMAN2	7.85603306	1.70285195	2.25033804	0.7567094	0.44922398	0.77681423	0.34753707	0.109682828
SF1	63.6838794	1.58203148	2.09270866	0.75597311	0.44966532	0.7771789	0.34711061	0.109478997
RBM6	72.081992	1.4366752	1.90103064	0.7557349	0.44980815	0.7771789	0.34697268	0.109478997
ETF1	17.9091204	2.15975868	2.86455034	0.7539608	0.45087276	0.77837237	0.345946	0.108812587
NMT1	16.7697372	1.00928211	1.34300647	0.75150949	0.45234609	0.78002882	0.34452916	0.10788935
DDHD1	11.2108148	2.10749375	2.81006561	0.74998027	0.45326659	0.78002882	0.34364629	0.10788935
RAPGEF2	175.509668	1.17565293	1.57001694	0.74881544	0.45396845	0.78076792	0.34297433	0.107478041
KLHL35	27.2635223	1.8373634	2.45460112	0.74853848	0.45413542	0.78076792	0.34281462	0.107478041
OTULIN	3.64855687	1.87417809	2.50840441	0.74715946	0.45496731	0.78155276	0.34201981	0.107041701
DMTN	413.900447	1.0066803	1.34971941	0.74584413	0.45576158	0.78213853	0.34126229	0.106716316
NPM1	46.5432556	1.96088928	2.63019381	0.74553034	0.45595118	0.78213853	0.34108166	0.106716316
KNG1	29.4378407	0.67204275	0.90160016	0.7453889	0.45603665	0.78213853	0.34100025	0.106716316
PDGFB	7.75344913	1.42638053	1.91400592	0.74523308	0.45613083	0.78213853	0.34091057	0.106716316
CDK9	11.9179012	1.82305158	2.44691739	0.7450401	0.45624748	0.78213853	0.34079952	0.106716316
TBL1XR1	446.774097	1.27470352	1.71543976	0.74307682	0.45743517	0.78310586	0.33967044	0.106179525
CALM3	86.7389348	1.07389428	1.44520862	0.74307215	0.457438	0.78310586	0.33966776	0.106179525
PPP3CA	52.4202262	1.82792541	2.46380091	0.74191279	0.45814017	0.78344792	0.33900162	0.105989868
PRPF40A	16.9809666	1.52102053	2.05263727	0.74100795	0.45868862	0.78364581	0.33848203	0.105880185
MOB1B	466.228127	0.98639438	1.33154297	0.74079049	0.45882048	0.78364581	0.3383572	0.105880185
SMPD1	39.1346733	1.49149688	2.01881156	0.73879945	0.46002878	0.78474898	0.33721499	0.105269238
MFN2	212.946771	1.52829583	2.06909958	0.73862845	0.46013264	0.78474898	0.33711696	0.105269238
LNPEP	93.9819931	1.52149734	2.06029757	0.73848427	0.46022023	0.78474898	0.3370343	0.105269238
AKAP9	923.74985	0.96713851	1.31114693	0.73762787	0.46074062	0.78475411	0.3365435	0.1052664
BRD4	95.3974088	1.21796058	1.66071799	0.73339398	0.46331817	0.78595827	0.33412067	0.104600511

TTC19	32.194159	1.45779636	1.98820561	0.73322213	0.46342296	0.78595827	0.33402245	0.104600511
BDP1	4.77962611	1.36558231	1.86524138	0.73212096	0.46409474	0.78606552	0.33339335	0.104541251
FAH	3.38049326	1.80289859	2.4636268	0.7318067	0.46428656	0.78606552	0.33321389	0.104541251
SSB	3.31701229	1.92907624	2.63665838	0.73163678	0.46439029	0.78606552	0.33311687	0.104541251
SLC24A1	2.98503682	2.13123495	2.91580625	0.73092475	0.46482512	0.78648183	0.33271041	0.104311305
MTRNR2L12	22.6053544	0.75572017	1.03799841	0.72805523	0.46657978	0.78825259	0.33107409	0.103334596
HJURP	3.09915076	2.06817154	2.84725585	0.72637362	0.46760976	0.78894937	0.33011643	0.102950868
TXK	23.2361908	1.83824173	2.53507722	0.72512258	0.46837683	0.78992351	0.32940459	0.102414962
USF3	12.016253	1.30445793	1.80027576	0.72458784	0.46870492	0.79000495	0.32910049	0.102370187
NSD3	79.1178052	1.43128164	1.97574728	0.72442546	0.46880457	0.79000495	0.32900816	0.102370187
VPS11	39.1824971	1.77951379	2.46426789	0.72212676	0.47021655	0.79087915	0.32770209	0.101889873
MT.CO1	519.147248	0.97037544	1.34467173	0.72164486	0.47051285	0.79087915	0.32742851	0.101889873
GTF2A2	122.444453	1.8462357	2.55887371	0.72150325	0.47059994	0.79087915	0.32734813	0.101889873
ITCH	5.30376256	1.7358827	2.40778153	0.72094693	0.47094217	0.79087915	0.32703242	0.101889873
SMOX	308.320823	1.02178524	1.41810695	0.72052763	0.47120019	0.79087915	0.32679454	0.101889873
MTRNR2L1	3.57556192	0.94019324	1.30529352	0.72029258	0.47134487	0.79087915	0.32666122	0.101889873
MAGI3	4.553778	1.59049157	2.20872266	0.72009565	0.47146611	0.79087915	0.32654952	0.101889873
C1GALT1	18.9367264	1.98059951	2.75137918	0.71985698	0.47161306	0.79087915	0.32641418	0.101889873
RFX2	55.5200852	1.81657524	2.52905844	0.71828125	0.47258389	0.79087915	0.32552109	0.101889873
ABCA7	415.74055	0.921731	1.28402208	0.71784669	0.47285182	0.79087915	0.32527493	0.101889873
UBA7	321.976383	1.10349043	1.53784037	0.7175585	0.47302955	0.79087915	0.32511173	0.101889873
NPLOC4	8.40813744	1.48394217	2.06842117	0.71742747	0.47311037	0.79087915	0.32503753	0.101889873
X6.Sep	101.069818	1.68645687	2.35078092	0.71740282	0.47312558	0.79087915	0.32502357	0.101889873
CBL	198.985914	0.95217799	1.32800627	0.71699811	0.47337526	0.79087915	0.32479444	0.101889873
PADI4	785.57406	0.99963725	1.39459692	0.71679296	0.47350186	0.79087915	0.32467832	0.101889873
ZNF714	72.5007974	1.63707626	2.29084457	0.71461691	0.47484579	0.79255551	0.3234474	0.100970312
HNRNPR	13.1785349	1.65639603	2.32082297	0.71371063	0.47540613	0.79264224	0.32293522	0.100922785
ASAP2	42.3572822	1.54534162	2.1657272	0.71354399	0.4755092	0.79264224	0.32284107	0.100922785
OR2B6	9.37594391	1.97908594	2.77534251	0.71309611	0.47578629	0.79278676	0.32258808	0.100843614
SUDS3	5.46359181	2.06308882	2.90340354	0.71057598	0.47734703	0.79475132	0.32116577	0.099768745
TCF25	146.551417	1.2308962	1.73404802	0.70983974	0.47780352	0.79482924	0.32075066	0.099726165
MGAT5	2.94381889	2.06896298	2.91576946	0.70957701	0.47796648	0.79482924	0.32060256	0.099726165
MOB1A	109.633826	1.60635264	2.26701132	0.70857725	0.47858686	0.79490853	0.32003923	0.099682842
SUPT4H1	3.02269953	1.87696954	2.6568389	0.7064672	0.47989764	0.79676786	0.31885138	0.098668194
HDAC6	335.38438	1.55244555	2.21032487	0.70236081	0.48245418	0.79936198	0.31654392	0.09725651
SPHK1	85.9442343	1.34765856	1.9194169	0.70211873	0.48260512	0.79936198	0.31640807	0.09725651
KMT2D	136.527897	1.23535883	1.75950013	0.70210783	0.48261192	0.79936198	0.31640196	0.09725651
GTF2A1	33.0087815	1.81116305	2.58486657	0.70067951	0.48350304	0.80051955	0.31560079	0.096628055
CCM2	143.123719	1.26869566	1.81251477	0.69996432	0.48394959	0.80056078	0.31519987	0.096605692
KMT5A	39.2248992	1.51140631	2.15953943	0.69987438	0.48400576	0.80056078	0.31514947	0.096605692
PBX2	22.9172655	1.27744535	1.82638777	0.69943818	0.48427823	0.80056078	0.31490505	0.096605692
INTS8	32.14737	1.82174703	2.60681953	0.69883895	0.48465268	0.80056078	0.31456938	0.096605692
BMP2K	68.2883116	1.8533937	2.65507261	0.69805763	0.48514115	0.80056078	0.31413188	0.096605692
STK40	139.966234	1.41733017	2.03228016	0.69740885	0.48554697	0.80056078	0.31376876	0.096605692
POGK	189.876345	1.63585135	2.34589714	0.69732441	0.48559979	0.80056078	0.31372151	0.096605692
HPS1	14.994141	1.52181088	2.18459114	0.6966113	0.48604608	0.80056078	0.31332255	0.096605692
STRBP	36.5622413	1.71248545	2.45861045	0.69652574	0.48609964	0.80056078	0.3132747	0.096605692
UIMC1	179.757256	1.15990308	1.67118898	0.6940586	0.48764546	0.80227408	0.31189581	0.095677239
ABCA3	51.6582438	1.57073949	2.26646338	0.69303546	0.4882873	0.80269574	0.31132457	0.095449041
CD46	124.568887	1.31165839	1.89683618	0.69149798	0.48925265	0.80396528	0.31046682	0.094762705
PLA2G4C	24.6495598	1.44384689	2.0965869	0.68866542	0.49103384	0.80545268	0.30888857	0.093959971
SYCP2	3.87467348	1.73451998	2.52002814	0.68829389	0.49126773	0.80545268	0.30868177	0.093959971
MOB3A	8.58160936	1.69066747	2.45960635	0.68737319	0.49184759	0.80551302	0.30816946	0.093927437
DMTF1	434.008063	1.51698491	2.20749236	0.68719826	0.4919578	0.80551302	0.30807215	0.093927437
NUMA1	121.199581	1.18497641	1.72504083	0.68692659	0.49212899	0.80551302	0.30792105	0.093927437
CAB39	5.62968871	1.49991933	2.19033645	0.68478947	0.49347678	0.80708457	0.30673327	0.093080953
ZNF792	35.9799125	1.39007751	2.0404133	0.68127252	0.49569907	0.80961116	0.3047819	0.091723516
DNM3	908.562164	0.88931285	1.30567395	0.68111403	0.49579934	0.80961116	0.30469406	0.091723516
TMEM57	86.770211	1.38752954	2.04009338	0.68013041	0.49642189	0.80979945	0.30414907	0.091622522
KHSRP	2.82881803	1.43151925	2.10514416	0.68001008	0.49649808	0.80979945	0.30408243	0.091622522
MIS18BP1	268.526096	1.33174597	1.96156949	0.67891858	0.49718945	0.80994337	0.30347809	0.091545347
MYBL1	3.44994024	1.93776606	2.85635605	0.67840494	0.49751498	0.80994337	0.30319384	0.091545347

RPL26	127.56111	1.55147393	2.28860908	0.67791129	0.49782794	0.81006469	0.30292073	0.091480297
EFNB1	5.04222721	1.83960043	2.7149641	0.67757818	0.49803918	0.81009186	0.30273649	0.091465731
ZNF596	3.3172903	1.90438226	2.82011478	0.67528537	0.49949447	0.81182474	0.30146931	0.090537717
TMEM165	8.51705736	1.73879702	2.57637997	0.6748993	0.49973975	0.81190648	0.30125611	0.090493992
RPL10	97.5473332	1.32081862	1.95874361	0.6743193	0.50010833	0.81218842	0.30093591	0.090343208
PTPRC	8.64047887	1.72316318	2.55795327	0.6736492	0.50053437	0.81256339	0.3005661	0.090142748
HNRNPD	54.1471606	1.48371116	2.20684073	0.67232363	0.50137769	0.81305834	0.29983499	0.089878293
ZNF211	140.479855	1.94237949	2.88920148	0.67228939	0.50139948	0.81305834	0.29981612	0.089878293
KIF5B	70.5521493	1.57145009	2.33762192	0.67224305	0.50142898	0.81305834	0.29979057	0.089878293
PLPP5	52.9970917	1.36748185	2.03511729	0.67194253	0.50162028	0.81305834	0.29962491	0.089878293
LAPTM5	75.0786777	1.92853967	2.87383183	0.67106908	0.50217653	0.81364321	0.29914359	0.089565994
IWS1	16.6017378	1.79200928	2.67795424	0.66917099	0.50338641	0.81465255	0.29809851	0.089027577
RBM12B	3.87376199	1.17362288	1.75906785	0.66718454	0.50465426	0.81623752	0.29700605	0.088183448
TNR	28.139483	0.62898569	0.94309628	0.66693688	0.50481245	0.81623752	0.29686994	0.088183448
TCTE3	203.875401	1.85874847	2.78791833	0.66671554	0.50495385	0.81623752	0.29674831	0.088183448
CUTA	32.7608344	1.9249165	2.89104423	0.66582049	0.50552584	0.81660733	0.29625664	0.087986724
TULP2	13.4634864	1.6446612	2.47099226	0.66558735	0.50567489	0.81660733	0.29612861	0.087986724
FAM76B	26.7319329	1.58301948	2.38700945	0.66318107	0.50721459	0.81766998	0.29480826	0.087421945
DAZAP2	37.389089	1.45409206	2.19999005	0.66095393	0.50864186	0.8189897	0.2935879	0.08672156
MAN1A2	3.27805689	1.4088283	2.13354913	0.66032147	0.50904756	0.8189897	0.29324164	0.08672156
ID2	290.330275	0.68013025	1.03332669	0.6581948	0.51041297	0.81965473	0.2920783	0.086369051
BCL7B	20.4627309	1.89213543	2.89246558	0.65416005	0.5130087	0.82283369	0.28987527	0.084687937
ZFYVE27	60.596539	1.88471884	2.88993023	0.65216759	0.51429306	0.82397727	0.28878933	0.084084771
RPS18	45.2622198	1.59468762	2.45052808	0.65075264	0.51520618	0.82512251	0.28801894	0.083481567
SNRPA1	14.5738304	1.69542779	2.61544092	0.64823785	0.51683113	0.8261362	0.28665134	0.082948346
ADAMTS6	7.19614417	1.69540265	2.63052916	0.64451012	0.5192447	0.82840419	0.28462793	0.081757715
ADAM10	146.812004	1.06407326	1.65537884	0.64279743	0.52035555	0.82865713	0.28369981	0.08162513
EIF4G3	47.5379167	1.19869086	1.86784279	0.64175147	0.52103456	0.82865713	0.28313347	0.08162513
ATP11A	4.81972671	1.8635738	2.90471619	0.64156829	0.52115353	0.82865713	0.28303432	0.08162513
SRCAP	152.343862	0.99280602	1.54806665	0.64131995	0.52131483	0.82865713	0.28289992	0.08162513
CDK17	17.8387947	1.49106016	2.32542437	0.64119916	0.52139329	0.82865713	0.28283456	0.08162513
SBDS	4.42109589	1.85973503	2.90658338	0.63983543	0.5222796	0.82957537	0.28209694	0.08114415
PBX1	207.308098	1.26913651	1.98641034	0.63890954	0.52288178	0.8296494	0.28159649	0.081105396
STK35	50.8981336	1.7993953	2.82526966	0.63689329	0.52419435	0.8300449	0.28050766	0.080898414
RPL11	111.932578	1.53914704	2.42208626	0.63546335	0.52512626	0.8303986	0.27973626	0.080713391
TMEM9	87.1383873	1.83541856	2.88952879	0.6351965	0.52530027	0.8303986	0.27959238	0.080713391
TPST2	756.704528	0.91463489	1.44047071	0.63495556	0.5254574	0.8303986	0.27946249	0.080713391
KDM7A	137.458749	1.25003027	1.96879866	0.63492032	0.52548038	0.8303986	0.27944349	0.080713391
CYB5D2	57.4093392	1.23794112	1.95166202	0.63430098	0.52588442	0.830415	0.2791097	0.080704815
NLK	33.2896469	1.34023513	2.12100086	0.63188807	0.52746002	0.83067637	0.27781045	0.080568142
DCAF8	4.04359728	1.55170529	2.45577534	0.63185963	0.52747861	0.83067637	0.27779515	0.080568142
RYR1	4.36011465	1.10848181	1.75557065	0.63140826	0.52777362	0.83067637	0.27755232	0.080568142
FOSB	23.8843344	1.59751	2.53185507	0.63096424	0.52806391	0.83067637	0.27731351	0.080568142
RBBP5	13.3319156	1.78383366	2.82979592	0.63037538	0.52844902	0.83067637	0.2769969	0.080568142
TCERG1	17.4241439	1.38039433	2.19951571	0.62759012	0.53027248	0.83208464	0.27550091	0.079832494
DBNL	25.122135	1.3167099	2.09853521	0.62744237	0.5303693	0.83208464	0.27542162	0.079832494
ELF1	145.924613	0.90041672	1.43597247	0.62704317	0.53063094	0.83208464	0.27520743	0.079832494
DYRK1B	9.12573016	1.35748728	2.16686701	0.62647466	0.53100365	0.83217885	0.27490249	0.079783326
MED31	6.62730436	1.68105453	2.69799408	0.62307569	0.53323478	0.83396263	0.27308153	0.078853411
GALK2	28.9444472	1.3861917	2.23161348	0.6211612	0.53449357	0.83494149	0.27205752	0.078343957
RPS27A	138.966656	1.3249889	2.13348852	0.62104337	0.53457109	0.83494149	0.27199453	0.078343957
RSRC2	41.1039024	1.04160675	1.68336035	0.61876636	0.53607029	0.83632359	0.27077826	0.077625653
GFOD1	13.4342417	1.78520028	2.88643443	0.61847942	0.53625936	0.83632359	0.27062511	0.077625653
HIST1H3J	9.98016235	1.29610828	2.09861191	0.61760265	0.5368373	0.83691146	0.27015732	0.077320486
GAN	5.73419762	0.85857866	1.39233554	0.61664637	0.537468	0.83758112	0.26964739	0.07697312
RHEB	44.1814303	1.42725942	2.31767158	0.61581608	0.53801592	0.83794059	0.26920488	0.07678677
TRIM15	143.654438	1.44786602	2.35187631	0.61562167	0.53814425	0.83794059	0.2691013	0.07678677
SSU72	78.7893048	1.40763612	2.28741785	0.61538215	0.53830237	0.83794059	0.2689737	0.07678677
SUB1	100.81225	1.41323816	2.30208623	0.61389454	0.53928502	0.83898161	0.26818164	0.076247556
DCUN1D4	7.30180911	1.57508646	2.56629161	0.61375974	0.53937411	0.83898161	0.26810991	0.076247556
HNRNPH3	249.109985	1.05920088	1.73001576	0.61224927	0.54037285	0.84022127	0.26730648	0.07560633
CDC16	74.3356569	1.23362241	2.02045615	0.61056629	0.54148675	0.84083358	0.26641217	0.075289952

FAM110A	237.819381	0.72458625	1.18700183	0.61043398	0.54157437	0.84083358	0.2663419	0.075289952
RPL30	30.1869935	1.2936838	2.12258061	0.6094863	0.54220215	0.8414945	0.26583876	0.074948716
ZBTB7A	30.8373812	0.98094143	1.61774659	0.60636285	0.54427383	0.84335319	0.26418254	0.073990507
PDPR	27.1092639	1.4205676	2.34313631	0.60626759	0.54433708	0.84335319	0.26413208	0.073990507
LSS	92.0520306	1.56782817	2.58669738	0.60611194	0.54444042	0.84335319	0.26404964	0.073990507
SPEN	307.563055	0.85911441	1.41851796	0.60564225	0.54475234	0.84335319	0.26380089	0.073990507
BRD1	52.4395502	1.13249275	1.87093178	0.60530948	0.54497338	0.84335319	0.26362471	0.073990507
C7orf49	29.6686285	1.5549885	2.56921291	0.60523925	0.54502004	0.84335319	0.26358753	0.073990507
AXIN1	192.523276	0.78244346	1.2943071	0.6045269	0.54549339	0.84367472	0.26321051	0.073824963
GP1BA	114.265487	1.1827257	1.96231132	0.60272072	0.54669449	0.84426705	0.2622553	0.073520159
TP53BP2	12.8061332	1.46775926	2.43853234	0.60190273	0.54723889	0.8445896	0.26182305	0.073354271
SPG7	16.495277	1.10765633	1.84579282	0.60009786	0.54844102	0.84581792	0.26087007	0.072723117
CARMIL1	101.644438	1.19281493	2.00269049	0.59560623	0.55143831	0.84898418	0.25850306	0.071100401
EIF5	564.295909	1.2172605	2.04461441	0.59534966	0.55160977	0.84898418	0.25836805	0.071100401
VPS13C	75.4981409	1.16950854	1.96714854	0.59451969	0.55216458	0.84898418	0.25793146	0.071100401
SPOP	69.540226	1.40594349	2.36724706	0.59391498	0.55256898	0.84898418	0.2576135	0.071100401
MGAT4C	7.05371427	1.05615289	1.77934875	0.59356148	0.55280545	0.84898418	0.25742769	0.071100401
TBC1D22B	26.7523388	1.62794493	2.74501682	0.59305463	0.55314459	0.84898418	0.25716133	0.071100401
CHFR	51.8893963	1.06225124	1.79523748	0.59170513	0.55404806	0.84943156	0.25645256	0.070871608
BCOR	124.392002	1.07708416	1.82307886	0.59080503	0.55465106	0.85004307	0.25598015	0.070559067
SIN3B	157.736117	1.26142597	2.14529576	0.5879963	0.55653478	0.85155593	0.25450769	0.069786822
PAOX	6.95498847	1.61092624	2.74223105	0.58745095	0.55690088	0.85155593	0.25422209	0.069786822
CASS4	4.80261518	1.383317	2.35522803	0.58733888	0.55697613	0.85155593	0.25416342	0.069786822
AFAP1L1	40.3042812	1.53600374	2.61831747	0.5866377	0.55744706	0.85156494	0.25379637	0.069782228
TMOD3	6.35029078	1.20933367	2.06166259	0.58658176	0.55748464	0.85156494	0.2537671	0.069782228
WDR83OS	7.26818047	1.38041425	2.35910401	0.58514345	0.55845127	0.85189838	0.25301472	0.069612207
SERINC3	184.687939	0.89158464	1.52423852	0.58493774	0.55858958	0.85189838	0.25290717	0.069612207
DTWD1	4.27618442	1.37700292	2.35459828	0.58481437	0.55867254	0.85189838	0.25284268	0.069612207
KIAA2026	65.0312449	1.03151376	1.76479362	0.5844954	0.55888706	0.85189838	0.25267595	0.069612207
WBP2	116.295851	0.88514763	1.51619562	0.58379514	0.55935815	0.85189838	0.25231003	0.069612207
PNPLA2	12.1174984	1.36759121	2.34270327	0.5837663	0.55937756	0.85189838	0.25229496	0.069612207
CD2AP	21.7314276	1.68535697	2.89212427	0.58274016	0.56006823	0.85238455	0.25175906	0.069364432
SMYD3	7.37079152	1.38619599	2.39149429	0.57963592	0.56216017	0.85463123	0.25013993	0.068221243
RPN2	66.7677028	1.4070125	2.43072153	0.57884562	0.56269335	0.8551296	0.24972822	0.067968061
MLXIP	722.522655	0.69184342	1.19942167	0.57681418	0.56406499	0.85658885	0.24867085	0.067227583
RC3H1	159.21581	0.88538907	1.54534295	0.57294018	0.5666852	0.85853849	0.24665813	0.066240228
IL16	226.74532	1.05414276	1.84536077	0.57123939	0.56783738	0.85949649	0.24577602	0.065755892
LEKR1	9.54246997	1.45074415	2.56925476	0.56465562	0.57230803	0.86393474	0.24237016	0.063519062
TXNL4B	35.4918368	1.20639276	2.13718581	0.56447724	0.57242938	0.86393474	0.24227808	0.063519062
CERS2	54.841609	1.33182217	2.36414419	0.56334219	0.57320189	0.86441576	0.24169238	0.063277322
KMT2B	30.8410663	0.83025816	1.47992576	0.56101338	0.57478841	0.86529969	0.240492	0.06283345
STX10	110.749919	1.61943775	2.88931656	0.56049163	0.57514415	0.86547106	0.2402233	0.06274745
MDM4	51.1743473	1.0756946	1.92663104	0.55832932	0.57661953	0.86628875	0.23911065	0.062337328
TGOLN2	285.475921	0.72972262	1.30941014	0.5572911	0.57732855	0.86628875	0.23857696	0.062337328
SREK1	85.8029024	1.22263624	2.19476303	0.55706982	0.57747973	0.86628875	0.23846326	0.062337328
DNAH10	19.7547177	1.05023357	1.88933082	0.55587595	0.57829566	0.86628875	0.23785007	0.062337328
TRAPPC2	27.311481	1.45398919	2.61620909	0.55576184	0.57837367	0.86628875	0.23779148	0.062337328
COX4I1	11.3281766	1.29984557	2.34646127	0.55395995	0.57960625	0.86628875	0.23686694	0.062337328
STAG1	11.6467007	1.28598325	2.32506535	0.55309553	0.580198	0.86686202	0.23642377	0.062050023
COPE	5.34921723	1.60421194	2.90252008	0.55269624	0.58047143	0.86695948	0.23621915	0.062001201
STRN3	14.5486757	1.46149494	2.64924431	0.55166484	0.581178	0.86749483	0.23569083	0.061733103
RIC1	22.525629	1.09495786	1.99344788	0.5492784	0.58281441	0.86890053	0.23446972	0.061029936
ZNF117	6.16423687	1.15157219	2.10289103	0.54761382	0.5839571	0.8700228	0.23361906	0.060469367
ZC3H12C	18.7830897	1.36335977	2.48982226	0.54757313	0.58398504	0.8700228	0.23359828	0.060469367
EIF1AD	6.7273127	1.08264647	1.97964636	0.54688882	0.58445512	0.8701005	0.23324884	0.060430581
PTK2B	57.0300795	1.33676586	2.44726399	0.54622871	0.58490873	0.8702528	0.2329119	0.06035457
SMARCC2	12.7433919	1.2726676	2.3303309	0.54613171	0.58497541	0.8702528	0.23286239	0.06035457
FOXN2	114.828674	1.20707167	2.21222064	0.54563801	0.58531479	0.87044596	0.2326105	0.060258184
CSDE1	29.5235401	1.24748717	2.28756234	0.54533472	0.58552333	0.87044596	0.2324558	0.060258184
C14orf2	35.1692621	1.17302248	2.16411824	0.54203253	0.5877961	0.87196444	0.2307733	0.059501226
FAM192A	27.4444409	1.08590688	2.0038019	0.54192327	0.58787136	0.87196444	0.2307177	0.059501226
CLCN3	69.0992051	0.82776909	1.53504903	0.53924603	0.5897171	0.87387261	0.22935628	0.058551875

RPL15	142.570277	1.02634299	1.90674481	0.53826972	0.59039085	0.87412711	0.22886038	0.058425411
RTN2	7.15366032	1.37642482	2.56717283	0.53616367	0.59184544	0.87412711	0.2277917	0.058425411
CLIC1	219.0487	0.97056143	1.8109765	0.53593265	0.5920051	0.87412711	0.22767455	0.058425411
HEATR5B	3.23837608	1.24262892	2.31866929	0.53592331	0.59201155	0.87412711	0.22766982	0.058425411
RPL23	25.5571107	1.466788	2.73803642	0.53570799	0.59216038	0.87412711	0.22756066	0.058425411
MTA2	24.7156108	0.95440085	1.78231885	0.53548267	0.59231614	0.87412711	0.22744643	0.058425411
FOXJ2	106.370324	0.82514868	1.54132215	0.53535121	0.59240702	0.87412711	0.2273798	0.058425411
HECA	24.3007417	1.22995884	2.29748603	0.53534987	0.59240795	0.87412711	0.22737912	0.058425411
ZNF207	294.786164	0.90487395	1.69517482	0.53379389	0.59348414	0.87535849	0.22659088	0.057814051
STAT5B	137.456653	1.21679605	2.2820823	0.53319552	0.59389824	0.8753955	0.22628796	0.05779569
ACCS	669.601805	0.66133153	1.24214136	0.53241245	0.59444036	0.87539959	0.22589171	0.057793661
P2RY1	131.340002	1.10500716	2.0759864	0.53228054	0.59453171	0.87539959	0.22582498	0.057793661
ELMO2	3.19761557	1.32550022	2.49357782	0.53156561	0.59502689	0.8755357	0.22546341	0.057726142
SOX12	25.892922	1.19079275	2.24036007	0.53151847	0.59505955	0.8755357	0.22543957	0.057726142
CELF1	285.827357	1.3276285	2.49946861	0.5311643	0.59530493	0.8755357	0.22526052	0.057726142
GNA13	136.320807	1.28479311	2.42214181	0.53043678	0.59580913	0.87573216	0.22489285	0.0576287
RNASEK	27.85416	1.52553943	2.89130374	0.52763029	0.59775597	0.87728518	0.22347608	0.056859209
MIER1	95.6331479	1.09579577	2.07896494	0.52708718	0.59813305	0.87728518	0.2232022	0.056859209
NRDC	554.330102	0.67124755	1.27370635	0.52700338	0.59819124	0.87728518	0.22315995	0.056859209
CORO7	235.69554	0.91910589	1.74473442	0.52678842	0.59834052	0.87728518	0.22305158	0.056859209
ULK2	11.1410243	1.21861296	2.31964785	0.52534395	0.59934411	0.87799689	0.22232376	0.056507022
PKP4	8.02861205	0.93822034	1.78710642	0.52499411	0.59958729	0.87799689	0.22214759	0.056507022
ATXN1L	4.24855062	1.50656997	2.87184219	0.52460054	0.59986091	0.87799689	0.22194944	0.056507022
MED16	305.649793	0.69590901	1.32662046	0.5245728	0.5998802	0.87799689	0.22193547	0.056507022
ZC3H4	25.6084423	0.98005767	1.87282199	0.5233053	0.60076181	0.87838182	0.22129768	0.05631666
DAD1	104.175775	1.40014814	2.68241533	0.52197291	0.60168919	0.87857115	0.22062779	0.05622306
TMED2	2.78240063	1.38848412	2.66051081	0.52188629	0.6017495	0.87857115	0.22058426	0.05622306
NBPF10	10.728376	1.16313743	2.23834009	0.51964285	0.60331253	0.88023797	0.21945766	0.055399903
LRBA	413.100501	0.73743212	1.41911883	0.51964085	0.60331392	0.88023797	0.21945666	0.055399903
ZFPL1	6.31712389	1.34013922	2.58062585	0.51930783	0.60354609	0.88026828	0.21928956	0.05538495
C10orf10	123.79637	1.10611013	2.13217538	0.51877071	0.60392065	0.88050615	0.21902012	0.055267605
ZBED5	50.3241604	1.28704994	2.48719546	0.51747036	0.60482785	0.88152019	0.21836822	0.054767737
GPX4	202.643009	0.47260927	0.91554915	0.51620306	0.6057126	0.88179148	0.21773339	0.0546341
FKBP8	18.2591799	1.03806229	2.0116315	0.51603004	0.60583343	0.88179148	0.21764677	0.0546341
ZNF431	157.466474	0.74206259	1.44014011	0.51527111	0.60636359	0.88195585	0.21726689	0.054553152
LUC7L3	693.851284	0.6294678	1.22173992	0.51522242	0.6063976	0.88195585	0.21724253	0.054553152
CA2	136.423382	0.7019693	1.36436076	0.51450417	0.60689954	0.88202542	0.21688319	0.0545189
NT5C3A	112.894082	0.77687766	1.51019314	0.51442272	0.60695648	0.88202542	0.21684245	0.0545189
JTB	33.5279994	1.48660243	2.89084617	0.51424474	0.60708089	0.88202542	0.21675344	0.0545189
SH3BGRL3	36.6236294	1.00599138	1.96045351	0.51314218	0.60785187	0.88237111	0.21620224	0.054348718
ITPR2	298.047722	0.76059384	1.48348015	0.51270915	0.60815479	0.88237111	0.21598587	0.054348718
ARRB1	58.1772823	0.78741017	1.53583327	0.51269248	0.60816645	0.88237111	0.21597754	0.054348718
CNOT2	76.6038744	1.21257405	2.3702131	0.51158862	0.60893895	0.88318419	0.21542624	0.053948716
PACS1	101.850103	0.75114454	1.47007723	0.51095583	0.60938198	0.88347601	0.21511039	0.053805238
UQCRC2	10.8944561	1.418405	2.7867094	0.5089892	0.61075979	0.88347601	0.21412956	0.053805238
UBXN2A	49.1431529	1.08223203	2.12888048	0.50835735	0.61120276	0.88347601	0.21381469	0.053805238
AP2M1	194.956953	0.89572409	1.76236783	0.50825036	0.61127778	0.88347601	0.21376139	0.053805238
RPL18A	12.9199165	1.46876592	2.89439493	0.5074518	0.61183785	0.88347601	0.21336366	0.053805238
HNRNPA0	44.2881289	1.4196155	2.79849978	0.50727733	0.61196024	0.88347601	0.21327679	0.053805238
PABPN1	123.65361	0.71837153	1.41962212	0.5060301	0.61283552	0.88347601	0.21265607	0.053805238
CXXC4	10.2176976	0.66913058	1.32327635	0.50566201	0.61309394	0.88347601	0.21247298	0.053805238
RPS21	15.7325616	1.34374679	2.65760655	0.50562292	0.61312139	0.88347601	0.21245354	0.053805238
DDX20	48.6056631	1.31224567	2.59669602	0.50535206	0.61331159	0.88347601	0.21231883	0.053805238
LRRC8B	26.2641667	0.87866447	1.73974008	0.50505503	0.61352019	0.88347601	0.21217114	0.053805238
SPECC1	378.97086	0.75488878	1.49543909	0.50479407	0.61370348	0.88347601	0.21204141	0.053805238
TPRKB	4.25784187	1.44641152	2.86619368	0.50464542	0.6138079	0.88347601	0.21196753	0.053805238
KLHDC3	62.6922606	1.44178946	2.85930473	0.50424477	0.61408938	0.88357574	0.21176841	0.053756217
PSEN1	4.22029153	1.02694982	2.04140239	0.50306095	0.61492142	0.88385668	0.21118038	0.053618152
CD109	100.752855	1.45110333	2.88937048	0.50222128	0.61551188	0.88440009	0.21076356	0.053351223
ISCU	32.0948954	1.40259617	2.79454785	0.50190451	0.61573469	0.88441506	0.21060638	0.053343871
GUCY1B3	124.707637	0.68132027	1.36234967	0.50010676	0.61699991	0.88501124	0.2097149	0.053051215
HACD4	107.875824	1.19470218	2.39446748	0.49894275	0.61781972	0.88548951	0.20913823	0.052816577

ATP6V0C	9.17576227	1.10678386	2.23237015	0.49578869	0.62004351	0.88662815	0.20757783	0.052258485
CCDC88C	699.477565	1.04130254	2.10829622	0.49390713	0.62137177	0.88732954	0.20664848	0.051915059
TSC22D4	100.495958	0.9326832	1.89330867	0.49262078	0.62228056	0.88789127	0.20601377	0.051640216
RBM28	2.75593121	1.31759064	2.67927702	0.49177096	0.62288127	0.88794166	0.20559473	0.051615567
DNMT3A	3.41510166	1.14551658	2.33471343	0.49064548	0.62367722	0.88876799	0.20504012	0.051211594
CYP20A1	7.108052	1.08634774	2.21546368	0.4903478	0.62388781	0.88876799	0.2048935	0.051211594
MT.ND6	49.567	0.74153085	1.51573406	0.48922226	0.62468435	0.88895755	0.20433937	0.051118978
HIST1H4C	519.854287	0.91822238	1.87798277	0.48894079	0.62488361	0.88895755	0.20420086	0.051118978
MBP	66.5515236	1.01243017	2.08166677	0.48635554	0.62671511	0.88895755	0.20292984	0.051118978
N4BP2L2	144.307677	0.89703209	1.84582314	0.48597944	0.62698174	0.88895755	0.2027451	0.051118978
MLLT3	3.74862471	0.97150136	2.00599355	0.48429934	0.62817344	0.88895755	0.20192043	0.051118978
LRWD1	120.929631	0.98047964	2.02499868	0.48418779	0.6282526	0.88895755	0.20186571	0.051118978
SRSF2	81.1435405	1.39446274	2.88954294	0.48258938	0.62938732	0.88945902	0.20108201	0.050874055
RPL4	287.555685	0.97321144	2.01985227	0.48182308	0.62993163	0.88945902	0.20070658	0.050874055
TTC7B	117.764175	0.88972028	1.84974813	0.48099537	0.63051979	0.88945902	0.20030128	0.050874055
HIST1H4E	196.808172	1.09316866	2.27349767	0.48083122	0.63063646	0.88945902	0.20022093	0.050874055
KIAA1109	145.219945	0.83935483	1.74654872	0.48057911	0.63081567	0.88945902	0.20009753	0.050874055
KLHDC10	52.1015965	1.38816883	2.89004797	0.48032726	0.63099471	0.88945902	0.19997428	0.050874055
KIAA1191	150.809167	1.35794944	2.83462301	0.47905822	0.63189721	0.89024502	0.19935356	0.050490449
FLT1	184.306674	1.27935487	2.67575138	0.4781292	0.63255825	0.89075839	0.19889948	0.050240079
ACTR3	38.3274391	1.16551362	2.44030314	0.47761018	0.63292769	0.89089693	0.19864591	0.050172538
STXBP5	13.2134849	0.9512061	1.99251427	0.47738985	0.63308454	0.89089693	0.19853829	0.050172538
GPBP1L1	25.2869428	1.02631735	2.1551524	0.47621567	0.63392074	0.89109937	0.19796504	0.050073864
F13A1	681.581911	0.75817531	1.59285233	0.47598594	0.6340844	0.89109937	0.19785293	0.050073864
MICALL2	31.7806561	0.91818169	1.93352834	0.47487366	0.63487703	0.89191226	0.19731039	0.049677867
BAG1	7.60227671	1.07875112	2.28109382	0.47290958	0.63627769	0.89271602	0.1963533	0.049286673
HNRNPA1	78.2968668	1.03400254	2.18651462	0.4728999	0.6362846	0.89271602	0.19634859	0.049286673
ILK	120.718835	0.82576934	1.74716953	0.47263263	0.6364753	0.89271602	0.19621845	0.049286673
ARPC5	23.3245712	1.0584029	2.23968169	0.47256845	0.6365211	0.89271602	0.1961872	0.049286673
NFATC1	356.259588	0.5646301	1.20058034	0.47029764	0.63814238	0.89368285	0.19508241	0.048816574
HINT1	12.4469082	1.25009804	2.66023232	0.46992063	0.63841173	0.89368285	0.19489914	0.048816574
FUS	61.0731636	0.81504888	1.73714601	0.46918847	0.63893492	0.89368285	0.19454337	0.048816574
MAPK6	24.1115827	1.07803804	2.29870323	0.46897661	0.63908636	0.89368285	0.19444045	0.048816574
EXOC6	31.842205	1.0334106	2.20773459	0.46808643	0.63972278	0.89368285	0.19400818	0.048816574
HSPA8	31.8573052	1.01544165	2.16962021	0.46802738	0.63976501	0.89368285	0.19397951	0.048816574
SSR1	27.6932743	0.42920107	0.91818592	0.46744462	0.6401818	0.89368285	0.19369667	0.048816574
ARF6	6.292256	0.85376139	1.82922398	0.4667342	0.64069006	0.89404605	0.19335201	0.048640112
BROX	417.719921	1.05429019	2.26262919	0.46595801	0.64124556	0.89426501	0.19297563	0.048533761
HIST2H4A	27.8308102	0.90197985	1.93593308	0.46591479	0.64127649	0.89426501	0.19295468	0.048533761
MFSD14A	15.2895282	1.23663676	2.66181523	0.464584	0.64222939	0.89499438	0.19230982	0.048179694
GAK	235.035663	0.79333794	1.71023092	0.46387767	0.64273539	0.89510039	0.19196779	0.048128254
KIAA0232	144.412407	0.91178725	1.96909119	0.46304978	0.64332868	0.89562709	0.19156709	0.047872777
LIMD1	32.499176	1.13922269	2.46732163	0.46172444	0.64427895	0.89635066	0.19092606	0.047522057
RAB32	185.377842	1.03911493	2.25909272	0.45997002	0.64553774	0.89660412	0.19007836	0.047399272
SPDYC	4.55542147	1.16670435	2.54642614	0.45817325	0.64682797	0.89748697	0.18921121	0.046971849
PRMT2	45.9035934	0.89305546	1.95172197	0.45757309	0.64725918	0.89748697	0.18892178	0.046971849
GPCPD1	325.425676	0.83548975	1.82840763	0.45694939	0.64770742	0.89752203	0.18862113	0.046954881
NHLRC3	7.15367248	1.10690106	2.42878547	0.45574262	0.64857507	0.89801949	0.18803975	0.046714239
TRIM10	543.53211	1.31488299	2.888772	0.45517022	0.64898678	0.89801949	0.18776415	0.046714239
PRKAB1	43.9722415	0.76155825	1.67354682	0.45505643	0.64906864	0.89801949	0.18770937	0.046714239
UBE2F	73.2419561	0.98551011	2.16752738	0.4546702	0.64934652	0.89801949	0.18752348	0.046714239
SLC12A2	2.75245608	1.28850765	2.8378003	0.45405156	0.64979172	0.89801949	0.18722583	0.046714239
TCF4	22.8856773	0.86647587	1.91989102	0.45131513	0.65176244	0.90014554	0.18591067	0.045687265
SMAD1	4.15380382	1.20711903	2.68125539	0.45020666	0.65256144	0.90049229	0.18537859	0.045520003
PPP6R3	250.240155	0.82031126	1.82300659	0.44997712	0.65272694	0.90049229	0.18526847	0.045520003
AGTPBP1	25.3933582	0.88763947	1.97326528	0.44983281	0.652831	0.90049229	0.18519923	0.045520003
COL4A3BP	15.495301	1.05330821	2.34189806	0.44976689	0.65287853	0.90049229	0.18516761	0.045520003
BTK	487.453189	0.59323782	1.32048056	0.44925904	0.6532448	0.90069913	0.18492404	0.045420258
RPL34	19.1317977	1.10594315	2.46561171	0.44854717	0.65375835	0.90110883	0.18458275	0.045222754
NOL8	55.7624167	1.12647717	2.51334323	0.4481987	0.6540098	0.90115712	0.18441574	0.045199483
OBSCN	6.71271091	0.80609077	1.80035183	0.44774069	0.65434035	0.90131297	0.1841963	0.045124378
PPP2R5C	10.3214909	1.04228638	2.32943251	0.44744219	0.65455582	0.90131297	0.18405331	0.045124378

DCAKD	11.2916441	0.96295248	2.1591313	0.4459907	0.65560396	0.90245782	0.18335843	0.044573088
ZZEF1	408.240299	0.72969815	1.63852315	0.44533893	0.65607484	0.90280754	0.18304662	0.044404821
SLC35A3	24.605301	0.98443627	2.22979309	0.44149221	0.6588567	0.90484146	0.18120903	0.043427509
STXBP3	136.782981	0.78929848	1.79566541	0.43955765	0.66025752	0.90535672	0.18028665	0.043180272
PGGT1B	30.3408449	1.13374819	2.58695696	0.43825553	0.66120106	0.90535672	0.17966646	0.043180272
C4orf22	17.7694539	1.03828774	2.37188413	0.43774809	0.66156891	0.90535672	0.17942492	0.043180272
DDRGK1	7.9694234	1.26722135	2.89769462	0.43732053	0.66187891	0.90535672	0.17922146	0.043180272
CEBPZOS	2.80182272	1.27363394	2.91413061	0.43705451	0.66207181	0.90535672	0.1790949	0.043180272
NLGN3	4.61878244	0.9708499	2.22326817	0.43667692	0.66234567	0.90535672	0.1789153	0.043180272
HLA.B	590.149568	0.43128447	0.98849231	0.43630533	0.66261521	0.90535672	0.1787386	0.043180272
CKB	3.67233133	1.26781961	2.90834316	0.43592504	0.66289112	0.90535672	0.1785578	0.043180272
CAND1	31.5770218	1.13741299	2.61014494	0.43576622	0.66300636	0.90535672	0.17848231	0.043180272
ETFA	29.8440702	0.65137507	1.4963336	0.43531407	0.66333447	0.90535672	0.17826743	0.043180272
COL24A1	949.988698	0.67042524	1.54171705	0.43485621	0.6636668	0.90535672	0.17804991	0.043180272
TRPS1	90.9327664	0.96935247	2.23313739	0.4340765	0.66423289	0.90535672	0.17767963	0.043180272
USP24	95.6915951	0.92382105	2.1318468	0.43334308	0.66476555	0.90535672	0.1773315	0.043180272
UPF2	52.3508324	0.6363836	1.47288025	0.43206744	0.6656924	0.90586377	0.1767264	0.042937109
DOK6	9.21152733	0.95543892	2.21735259	0.43089174	0.66654709	0.90665545	0.17616916	0.042557724
CEP44	4.53248897	0.71740352	1.66579175	0.43066819	0.66670965	0.90665545	0.17606326	0.042557724
ERG	54.7593776	1.24313971	2.88996577	0.43015725	0.66708126	0.90686463	0.17582126	0.042457535
NACA	353.828319	0.97944144	2.28211606	0.42918126	0.66779132	0.90750028	0.17535923	0.042153231
VPS13A	211.428111	0.66703352	1.5552383	0.42889474	0.66799983	0.90750028	0.17522365	0.042153231
UBE2L3	4.15871268	0.93301832	2.17681607	0.42861606	0.66820266	0.90750028	0.1750918	0.042153231
GRSF1	111.092296	0.88321793	2.06713151	0.42726741	0.66918457	0.90781038	0.17445408	0.042004858
E2F1	101.399421	1.15154143	2.70430932	0.4258172	0.67024105	0.90817761	0.17376898	0.041829208
ZNF33A	4.89719921	0.7676257	1.80554971	0.42514792	0.67072885	0.90817761	0.17345301	0.041829208
SUZ12	26.7027695	1.14913535	2.70669269	0.42455332	0.67116233	0.90817761	0.17317243	0.041829208
IDH3B	51.9930951	1.18905885	2.80154875	0.42442911	0.67125289	0.90817761	0.17311383	0.041829208
RFX1	8.93195419	0.78436234	1.86112931	0.4214443	0.67343068	0.90929758	0.1717071	0.041293964
C18orf8	317.489835	0.6762643	1.60477085	0.42140864	0.67345671	0.90929758	0.17169032	0.041293964
PIK3CB	39.5601702	0.96565686	2.29645883	0.42049822	0.67412153	0.90953915	0.1712618	0.041178602
NDUFA4	4.22394567	1.22113081	2.90553076	0.42027805	0.67428234	0.90953915	0.17115821	0.041178602
RPS24	28.6950306	0.70824328	1.68522493	0.42026632	0.67429091	0.90953915	0.17115269	0.041178602
TGFBRAP1	8.29100738	1.03594998	2.47303841	0.41889765	0.67529093	0.9102983	0.17050908	0.040816269
ADAM12	12.8397871	1.21123136	2.89427328	0.4184924	0.67558714	0.91040287	0.17031862	0.040766381
PCNX4	13.8790301	0.89853335	2.15621728	0.41671744	0.67688509	0.91141267	0.16948505	0.040284938
SLC3A2	255.039682	0.67177979	1.61224655	0.41667311	0.67691752	0.91141267	0.16946424	0.040284938
TMEM175	56.0769335	1.11137209	2.67508669	0.41545274	0.67781051	0.91168513	0.1688917	0.040155129
MTPN	29.8041673	0.87265036	2.10394328	0.41476896	0.67831105	0.91196113	0.16857111	0.040023672
RPS8	4.66414552	1.19906484	2.90404599	0.41289458	0.67968385	0.91304584	0.16769305	0.039507416
STX4	65.891333	0.76390641	1.85047501	0.41281639	0.67974114	0.91304584	0.16765644	0.039507416
RFFL	9.7356166	1.17410426	2.84916075	0.41208776	0.6802751	0.9132834	0.16731542	0.039394434
DDX6	346.673268	0.69873012	1.70343148	0.41018974	0.68166677	0.91415794	0.16642788	0.038978763
HNRNPK	969.339686	0.84727583	2.07760226	0.40781426	0.68341004	0.91475714	0.16531864	0.038694192
AVPR1A	26.0961524	1.05195897	2.57969141	0.40778481	0.68343167	0.91475714	0.1653049	0.038694192
FBXO9	32.3973508	0.9159351	2.25086957	0.406925	0.68406307	0.91482038	0.16490385	0.038664168
MGRN1	3.30264067	0.78444934	1.9291195	0.40663595	0.68427539	0.91482038	0.16476908	0.038664168
RBM41	17.2384802	0.97036445	2.38873736	0.40622484	0.6845774	0.91482038	0.16457744	0.038664168
RPL41	161.965106	0.75198221	1.85364447	0.40567769	0.68497944	0.91483828	0.16432247	0.03865567
SRSF1	230.634492	0.83409617	2.05640648	0.40560861	0.6850302	0.91483828	0.16429028	0.03865567
CUX1	68.0365506	0.67788119	1.67587905	0.40449291	0.68585029	0.91497664	0.16377067	0.038589993
CDKN1C	3.68136275	0.71864877	1.77864503	0.40404283	0.68618122	0.91497664	0.16356117	0.038589993
ZNF737	2.71139414	1.0538067	2.60953236	0.40382971	0.68633795	0.91497664	0.16346199	0.038589993
X2.Mar	252.769129	0.48594684	1.20646142	0.40278689	0.68710501	0.91503075	0.16297689	0.038564309
SERAC1	13.3183306	0.4913485	1.22485637	0.40114785	0.68831127	0.91503075	0.16221512	0.038564309
GOLGB1	19.532507	0.96842917	2.41651277	0.40075483	0.68860064	0.91503075	0.16203258	0.038564309
CLK2	4.79540919	1.16343073	2.90357687	0.4006888	0.68864926	0.91503075	0.16200192	0.038564309
C1orf174	38.9390738	0.73684485	1.83920128	0.40063307	0.68869029	0.91503075	0.16197604	0.038564309
CEP97	35.8294953	0.93338483	2.33982916	0.39891153	0.68995839	0.91593796	0.1611771	0.038133943
NPR3	12.1999233	0.99722953	2.50085145	0.398756	0.690073	0.91593796	0.16110497	0.038133943
YLPM1	4.48700392	0.9500669	2.38259212	0.39875348	0.69007486	0.91593796	0.1611038	0.038133943
FAM118A	8.0497947	1.12639669	2.83617841	0.39715297	0.69125466	0.91656022	0.16036193	0.037838996

CBX5	346.977188	1.08509204	2.73396065	0.3968938	0.69144577	0.91656022	0.16024187	0.037838996
LTN1	6.72533754	1.15011641	2.89934483	0.39668148	0.69160236	0.91656022	0.16014354	0.037838996
CCDC90B	12.4560322	0.86595969	2.18626197	0.39609146	0.69203756	0.91684518	0.15987033	0.037703994
MBNL2	27.2889776	0.95085957	2.40286291	0.39571944	0.69231201	0.91690093	0.15969813	0.037677585
SERTAD2	60.9217637	1.07051365	2.70716269	0.3954375	0.69252004	0.91690093	0.15956766	0.037677585
PKHD1L1	616.029699	0.61986291	1.56914025	0.39503347	0.69281819	0.91700411	0.15938072	0.037628716
LY6G5C	29.4666077	0.68047068	1.72573766	0.39430714	0.69335429	0.91742208	0.15904479	0.037430812
YIPF3	109.593038	1.00799269	2.56016101	0.39372238	0.69378601	0.91747195	0.15877446	0.037407203
HSPD1	8.84772997	1.02734604	2.60973451	0.39365922	0.69383266	0.91747195	0.15874526	0.037407203
IL7	3.20712869	1.03968716	2.65040154	0.39227534	0.69485478	0.91771542	0.15810595	0.037291969
CSNK1D	10.7777436	0.80385937	2.05584473	0.39101171	0.69578858	0.918082	0.1575227	0.037118527
ATF1	4.7510157	1.1336459	2.90370253	0.39041393	0.69623049	0.918082	0.15724696	0.037118527
SNRPC	11.3696693	1.02034883	2.61547916	0.39011927	0.69644835	0.918082	0.15711109	0.037118527
DNAH17	8.40052477	0.51033073	1.30903742	0.3898519	0.69664606	0.918082	0.15698781	0.037118527
TMEM87B	90.7810486	0.65965603	1.69436103	0.38932436	0.69703622	0.918082	0.15674466	0.037118527
SAYSD1	24.2566242	1.04926604	2.69747518	0.3889808	0.69729035	0.918082	0.15658634	0.037118527
BAZ1A	49.9760594	0.82111711	2.11878953	0.38754067	0.69835599	0.91907532	0.15592314	0.036648894
SRRM1	33.2437778	0.85638556	2.21762482	0.38617243	0.69936897	0.91944336	0.15529364	0.036475019
RPL12	164.881426	0.84273252	2.18341442	0.38597003	0.69951887	0.91944336	0.15520057	0.036475019
PLEKHA6	95.6505681	1.03225163	2.6841455	0.38457365	0.70055332	0.91996895	0.1545588	0.036226832
ICAM2	138.128538	0.476705	1.23969027	0.38453557	0.70058154	0.91996895	0.15454131	0.036226832
ARRDC2	43.8739041	0.99437264	2.59103041	0.38377498	0.70114523	0.92012882	0.15419201	0.036151365
GALNT2	8.71467022	1.06193585	2.76994454	0.38337802	0.7014395	0.92013258	0.15400978	0.03614959
NADSYN1	119.07807	0.7692728	2.00782963	0.38313649	0.70161857	0.92013258	0.15389893	0.03614959
SP4	11.4038021	1.10410582	2.89495843	0.38138918	0.70291449	0.92066086	0.1530975	0.035900321
KIF2C	3.88314294	1.0119033	2.66553079	0.37962544	0.70422347	0.92066086	0.1522895	0.035900321
IRF9	7.287284	0.97395004	2.56637049	0.37950485	0.70431301	0.92066086	0.15223429	0.035900321
RAB24	27.397033	1.09676257	2.89127692	0.37933501	0.7044391	0.92066086	0.15215654	0.035900321
LYPLA1	7.24515872	1.09569083	2.89853704	0.37801512	0.70541936	0.92066086	0.15155263	0.035900321
SPIN3	4.13080043	0.40065167	1.06014241	0.3779225	0.70548817	0.92066086	0.15151027	0.035900321
MT.CO3	209.727567	0.57356416	1.51840925	0.37774017	0.70562363	0.92066086	0.15142689	0.035900321
RPL36AL	149.63362	1.03213223	2.73627994	0.37720272	0.70602296	0.92066086	0.15118118	0.035900321
CTDSPL	127.551877	0.73870456	1.96083702	0.3767292	0.70637486	0.92066086	0.15096476	0.035900321
EIF4A2	212.115033	0.93068468	2.47135122	0.3765894	0.70647876	0.92066086	0.15090089	0.035900321
MAP4K2	426.573893	0.71787049	1.9064723	0.37654389	0.70651259	0.92066086	0.15088009	0.035900321
GIGYF2	5.45948288	0.70995885	1.88683362	0.37626998	0.70671619	0.92066086	0.15075496	0.035900321
CORO1C	478.700853	0.61611878	1.63767788	0.37621487	0.70675716	0.92066086	0.15072978	0.035900321
THBS1	473.071008	0.54472601	1.44951269	0.37579941	0.70706603	0.92066086	0.15054003	0.035900321
AQR	4.62774371	0.88454915	2.35455002	0.37567652	0.7071574	0.92066086	0.15048391	0.035900321
KIF14	7.76300216	0.9720061	2.59306785	0.37484793	0.70777357	0.92066086	0.15010566	0.035900321
POLR3E	34.0922565	1.03395785	2.75894259	0.37476599	0.70783451	0.92066086	0.15006827	0.035900321
HNRNPL	67.8251279	0.46851132	1.2509629	0.37452056	0.70801707	0.92066086	0.14995627	0.035900321
MAT2A	468.24232	1.08100083	2.88879231	0.3742051	0.70825173	0.92066086	0.14981236	0.035900321
SLC20A1	3.47182462	0.86623045	2.31752957	0.3737732	0.70857306	0.92066086	0.14961537	0.035900321
SLC2A3	403.787514	0.43091915	1.15428668	0.37332073	0.70890975	0.92066086	0.14940905	0.035900321
H2AFZ	124.606673	0.94694419	2.53924677	0.37292326	0.70920556	0.92066086	0.14922787	0.035900321
HERC2	550.854579	0.53133396	1.42846635	0.37196113	0.70992179	0.92119487	0.14878949	0.035648489
NELFCD	58.5963852	0.82544412	2.22866698	0.37037571	0.71110257	0.92186522	0.14806775	0.035332569
RAB1B	13.7792335	0.81213965	2.20451033	0.36839911	0.71257566	0.92269976	0.14716902	0.034939592
MYCT1	58.3894467	0.90723049	2.46291846	0.36835588	0.71260789	0.92269976	0.14714937	0.034939592
ORAI1	4.66992158	0.74439623	2.02104387	0.36832265	0.71263267	0.92269976	0.14713427	0.034939592
ING5	767.521938	0.57834913	1.57294449	0.36768566	0.71310764	0.92280709	0.14684491	0.034889078
UPF3A	48.3889218	0.70936611	1.93233007	0.36710401	0.71354144	0.92301539	0.1465808	0.034791057
SPTBN1	316.228757	0.85100743	2.32291615	0.36635306	0.71410164	0.92312855	0.14623997	0.034737817
ANKFY1	8.1824983	0.93580149	2.55824543	0.36579817	0.71451569	0.92312855	0.14598823	0.034737817
TBC1D9B	76.9255526	0.73561319	2.01584132	0.36491622	0.71517395	0.92369241	0.14558831	0.034472626
HIST1H2BJ	201.72231	0.5104387	1.40488995	0.36333002	0.71635837	0.92394204	0.14486966	0.034355271
RHBDL1	13.7480717	0.97721642	2.69298652	0.3628746	0.71669856	0.92394204	0.14466347	0.034355271
ZNF33B	3.77164003	0.71864698	1.98991761	0.36114409	0.71799174	0.92471229	0.14388055	0.033993369
IRF2BP2	24.2321802	0.80281065	2.22638439	0.36058941	0.7184064	0.92471229	0.14362981	0.033993369
EIF1	382.843131	0.65103319	1.81211293	0.35926745	0.71939502	0.92484127	0.14303257	0.033932798
HIST2H2BF	204.533081	0.81149195	2.26424499	0.35839406	0.72004844	0.92496375	0.14263829	0.033875285

RB1CC1	26.0343559	0.90800551	2.53843152	0.35770337	0.72056531	0.92520319	0.14232665	0.033762878
UGP2	12.9336797	1.0309263	2.88495589	0.3573456	0.72083309	0.92522413	0.14216528	0.033753047
TOPORS.AS1	15.9328469	0.89095805	2.49506656	0.35708789	0.72102601	0.92522413	0.14204907	0.033753047
SLC6A6	414.19887	0.70675842	1.99397317	0.35444731	0.72300369	0.92637625	0.14085949	0.03321259
NPEPL1	59.9409113	0.74038451	2.09029479	0.354201	0.72318825	0.92637625	0.14074864	0.03321259
ITM2A	80.864348	1.02136423	2.88953294	0.35347035	0.72373585	0.92637625	0.14041991	0.03321259
MYO1C	157.273388	0.71965272	2.03841885	0.35304458	0.72405502	0.92637625	0.14022843	0.03321259
ZC3H7A	77.7414227	0.69277193	1.96246438	0.35301121	0.72408004	0.92637625	0.14021343	0.03321259
TEX2	5.9421956	0.64640769	1.83159948	0.35291978	0.72414858	0.92637625	0.14017232	0.03321259
ARHGAP18	296.715476	0.51786399	1.47453411	0.35120516	0.72543444	0.92688217	0.13940183	0.032975471
NSD1	47.416785	0.60782088	1.74043784	0.34923446	0.72691329	0.92727323	0.13851739	0.032792278
NAA15	6.90823437	0.77751497	2.23031144	0.34861274	0.72738006	0.92727323	0.13823861	0.032792278
WASHC4	20.3826881	0.8798971	2.52658153	0.34825597	0.72764796	0.92727323	0.13807868	0.032792278
MAML3	54.3991386	0.58012036	1.66627922	0.34815315	0.72772517	0.92727323	0.1380326	0.032792278
TNXB	39.3874312	0.63378565	1.82167962	0.3479128	0.72790567	0.92727323	0.1379249	0.032792278
MTR	55.3616585	0.78904776	2.26897588	0.34775502	0.72802417	0.92727323	0.1378542	0.032792278
MITF	16.8691289	0.80394637	2.31290825	0.34759112	0.72814728	0.92727323	0.13778077	0.032792278
DNMT3B	34.0337108	0.84270299	2.42471271	0.34754756	0.72817999	0.92727323	0.13776126	0.032792278
HEPH	7.39383343	0.79949519	2.30047957	0.34753414	0.72819007	0.92727323	0.13775525	0.032792278
MDGA2	15.0761583	0.8580016	2.47281838	0.34697316	0.72861148	0.92752621	0.13750399	0.032673811
TBXA2R	29.1477544	0.59402986	1.71819767	0.34572848	0.72954678	0.92843301	0.13694685	0.032249429
SPG11	88.5869023	0.73529377	2.1341705	0.34453375	0.73044492	0.92900813	0.13641253	0.031980484
APPBP2	42.3073738	0.84181113	2.45737201	0.3425656	0.73192528	0.929875	0.13553325	0.031575429
OSER1	14.7252991	0.92532077	2.70192289	0.3424675	0.7319991	0.929875	0.13548945	0.031575429
LIMS1	123.432717	0.50997671	1.48930535	0.34242589	0.73203041	0.929875	0.13547088	0.031575429
PURB	5.52553481	0.80347054	2.34983244	0.34192674	0.73240603	0.929875	0.13524809	0.031575429
TARSL2	20.0198295	0.91307263	2.67100145	0.34184655	0.73246638	0.929875	0.13521231	0.031575429
RABGAP1	11.0050207	0.67993385	1.99833933	0.34024945	0.73366868	0.93044834	0.13450002	0.031307734
SLC6A18	12.4103494	0.46432553	1.36570182	0.33999042	0.73386374	0.93044834	0.13438457	0.031307734
ELMSAN1	590.384887	0.85709399	2.52269962	0.33975269	0.73404278	0.93044834	0.13427863	0.031307734
MFF	32.027856	0.89539133	2.63776264	0.33945106	0.73426996	0.93044834	0.13414424	0.031307734
MPZL3	46.4726222	0.88803547	2.62333668	0.33851372	0.73497609	0.9306315	0.13372679	0.031222252
ARHGAP45	5260.75682	0.39419291	1.1662473	0.33800114	0.73536233	0.9306315	0.13349862	0.031222252
NHLRC2	79.3702817	0.7595874	2.25546081	0.33677703	0.73628499	0.9306315	0.13295405	0.031222252
SGSM3	84.5188641	0.78821098	2.34819057	0.33566738	0.7371217	0.9306315	0.1324608	0.031222252
SERP1	49.3252695	0.94353573	2.81410787	0.33528769	0.73740808	0.9306315	0.13229211	0.031222252
CGGBP1	57.8533098	0.80068303	2.38849544	0.33522485	0.73745547	0.9306315	0.1322642	0.031222252
CAPRIN1	5.49027306	0.92932229	2.77427574	0.33497834	0.73764142	0.9306315	0.1321547	0.031222252
C11orf87	33.2236775	0.96792259	2.89081483	0.33482691	0.73775566	0.9306315	0.13208745	0.031222252
SMAGP	27.2250203	0.29922606	0.8982565	0.33311873	0.73904466	0.93141101	0.13132932	0.030858634
BCR	275.274281	0.55562331	1.6777453	0.33117262	0.7405141	0.93243423	0.13046667	0.030381791
M6PR	97.3857709	0.94750583	2.86122618	0.33115377	0.74052834	0.93243423	0.13045832	0.030381791
BNIP3L	517.793094	0.59270884	1.8085216	0.32773114	0.74311496	0.93387984	0.128944	0.029709002
ANKRD9	35.9014235	0.53104258	1.64680048	0.32246929	0.7470972	0.93628728	0.12662289	0.028590876
CSMD3	9.15933764	0.56448904	1.75108607	0.3223651	0.74717612	0.93628728	0.12657702	0.028590876
SACS	2.75827908	0.46795218	1.45180381	0.32232467	0.74720675	0.93628728	0.12655922	0.028590876
TACC1	68.364839	0.62701778	1.94662447	0.32210515	0.74737303	0.93628728	0.12646258	0.028590876
CCDC88A	2.72529403	0.93776961	2.91455426	0.32175404	0.74763904	0.93629141	0.12630803	0.02858896
CWC25	19.8997052	0.76310768	2.37627105	0.32113663	0.74810685	0.93637834	0.12603637	0.028548641
TMTC3	15.2357076	0.55181621	1.72601304	0.3197057	0.74919144	0.93682677	0.12540719	0.028340707
GCC2	32.0513621	0.61715528	1.9376935	0.31849995	0.75010573	0.93746295	0.12487751	0.028045885
TPP2	332.224884	0.46660122	1.4652676	0.31844096	0.75015047	0.93746295	0.12485161	0.028045885
SPCS3	19.6806454	0.6697225	2.11554735	0.31657174	0.75156858	0.93786002	0.12403138	0.027861979
WIPF2	48.0232473	0.55271097	1.7545761	0.31501111	0.75275322	0.93874346	0.12334738	0.027453078
C11orf58	111.383976	0.76580833	2.43934288	0.31394042	0.75356629	0.93947606	0.12287854	0.027114284
MPIG6B	2875.31898	0.44970034	1.43559282	0.31325062	0.75409026	0.93984791	0.12257667	0.026942422
IST1	120.852106	0.54105002	1.73536508	0.31177879	0.75520865	0.94026788	0.12193305	0.026748401
WDTC1	21.8045772	0.73769348	2.3730595	0.31086177	0.75590571	0.94026788	0.12153237	0.026748401
FTH1	43.726201	0.33037182	1.06284125	0.31083835	0.75592351	0.94026788	0.12152215	0.026748401
EPC1	45.667988	0.76321026	2.45650003	0.31069011	0.75603622	0.94026788	0.1214574	0.026748401
IL18BP	3.33078164	0.77033622	2.49122392	0.30921998	0.75715419	0.94074865	0.12081567	0.026526396
GRB10	29.6968237	0.7540989	2.445274	0.30839035	0.75778533	0.94074865	0.12045381	0.026526396

IQGAP2	326.044181	0.5678276	1.84275935	0.30813985	0.75797592	0.94074865	0.12034459	0.026526396
ARID5A	103.376725	0.87864919	2.88933992	0.30410032	0.76105146	0.94400307	0.11858597	0.025026594
DYNLL2	6.13739632	0.88022175	2.90040354	0.30348251	0.76152217	0.94402451	0.11831745	0.025016729
NDUFB6	70.6787515	0.72809482	2.41476177	0.30151828	0.76301931	0.94419862	0.11746447	0.024936637
CIRBP	347.433043	0.41459917	1.38183342	0.30003556	0.76415003	0.94419862	0.11682137	0.024936637
SUSD1	127.203329	0.64767898	2.16680928	0.29890908	0.76500942	0.94419862	0.11633322	0.024936637
ELOVL7	28.9112616	0.59539435	1.99273555	0.29878242	0.76510606	0.94419862	0.11627836	0.024936637
NET1	113.079601	0.66305883	2.2214598	0.29847888	0.76533769	0.94419862	0.1161469	0.024936637
MGEA5	1416.4136	0.37136332	1.24483709	0.29832283	0.76545678	0.94419862	0.11607932	0.024936637
HNRNPDL	162.166239	0.58743276	1.97278741	0.2977679	0.76588032	0.94436649	0.11583909	0.02485943
RAPGEF6	4.0158843	0.64165604	2.16689295	0.29611801	0.76713996	0.94477133	0.1151254	0.024673293
TET3	64.1223385	0.68067217	2.30546119	0.29524339	0.76780796	0.94506381	0.11474739	0.024538868
PRKG2	4.52260828	0.75153332	2.54810381	0.29493827	0.76804104	0.94506381	0.11461557	0.024538868
CCDC97	30.927159	0.5584251	1.89521722	0.29464965	0.76826153	0.94506381	0.11449092	0.024538868
CLINT1	14.7982604	0.5500698	1.87812082	0.29288308	0.76961153	0.94529196	0.11372844	0.024434034
BRD3	450.419979	0.45042213	1.54192525	0.29211671	0.77019739	0.94529196	0.11339796	0.024434034
NIN	12.2636688	0.64805542	2.2288529	0.29075738	0.77123689	0.9453725	0.11281221	0.024397033
PSMD12	11.6266443	0.84066027	2.89485634	0.29039792	0.77151183	0.94543121	0.11265741	0.024370063
MAPK8IP3	377.861909	0.47550563	1.66224135	0.28606293	0.77482992	0.94753175	0.11079362	0.023406231
TGFB1	22.2394822	0.44049249	1.54154498	0.28574741	0.77507159	0.94753175	0.11065818	0.023406231
FBXW5	4.58363473	0.82886111	2.90431076	0.28538995	0.7753454	0.94753175	0.11050478	0.023406231
TUBGCP6	100.046477	0.67587422	2.36951388	0.2852375	0.77546219	0.94753175	0.11043937	0.023406231
ATF6B	13.8869371	0.6228924	2.18902722	0.28455215	0.77598727	0.94753175	0.1101454	0.023406231
SPINT2	134.791561	0.77792797	2.73435581	0.28450137	0.77602619	0.94753175	0.11012362	0.023406231
CEP83	4.74949998	0.71852183	2.53002176	0.28399828	0.7764117	0.94753175	0.10990793	0.023406231
PRPF39	22.0194946	0.59495336	2.09921531	0.28341702	0.77685718	0.94767396	0.10965882	0.023341054
ZFAND2B	369.754837	0.43221201	1.52589031	0.28325235	0.7769834	0.94767396	0.10958826	0.023341054
FOCAD	11.894419	0.61741172	2.18204948	0.28295038	0.77721488	0.94767869	0.1094589	0.023338883
PCF11	12.5072247	0.59592284	2.11613957	0.28160848	0.77824374	0.94865542	0.10888436	0.022891506
ZNF654	44.9021461	0.74315766	2.64387771	0.28108625	0.77864425	0.94870985	0.10866092	0.022866591
RPLP2	18.9407544	0.61490528	2.18861682	0.28095611	0.77874406	0.94870985	0.10860525	0.022866591
MIA3	14.0908428	0.74636746	2.66555593	0.28000443	0.77947411	0.94893608	0.10819831	0.022763039
ANKRD42	3.90898615	0.65199815	2.33628546	0.2790747	0.7801875	0.94904642	0.10780101	0.022712543
ATG3	61.4445247	0.46315877	1.66117682	0.27881365	0.78038784	0.94904642	0.10768951	0.022712543
CGRRF1	130.602543	0.66577047	2.39854139	0.27757306	0.78134011	0.94954047	0.10715988	0.02248652
NEMF	2.9767455	0.5552447	2.01045215	0.27617902	0.78241057	0.94970815	0.10656529	0.022409836
PTMS	106.596792	0.6612335	2.39601346	0.27597236	0.78256929	0.94970815	0.1064772	0.022409836
RNF24	34.0862228	0.43415877	1.5775936	0.27520318	0.78316015	0.94970815	0.10614942	0.022409836
PIGM	11.8318227	0.77359675	2.82783854	0.27356468	0.78441918	0.95050513	0.1054518	0.022045534
EGR1	9.50533553	0.56388055	2.06295554	0.27333626	0.78459474	0.95050513	0.10535461	0.022045534
RIOK3	263.817302	0.35016354	1.28290355	0.27294611	0.78489464	0.95050513	0.10518864	0.022045534
RHOB	22.2895492	0.78793378	2.89190131	0.2724622	0.78526666	0.95053789	0.10498284	0.022030565
COL6A3	253.938706	0.63517237	2.33488213	0.27203616	0.78559421	0.95065804	0.10480172	0.021975676
ITFG1	114.870425	0.53108056	1.96496139	0.27027532	0.78694845	0.95101119	0.10405371	0.021814374
UNKL	6.81211713	0.59183544	2.19721038	0.26935766	0.78765447	0.95101119	0.10366426	0.021814374
STON1	43.9116538	0.41383983	1.53674289	0.26929673	0.78770135	0.95101119	0.10363841	0.021814374
RNF215	76.477907	0.40770731	1.51405681	0.26928138	0.78771316	0.95101119	0.1036319	0.021814374
IVNS1ABP	70.7454981	0.51374439	1.91411827	0.26839741	0.78839343	0.95132069	0.103257	0.021673059
DYNC1LI1	4.78769302	0.7196613	2.68175578	0.26835453	0.78842644	0.95132069	0.10323882	0.021673059
PLEKHA2	5.01830266	0.38255802	1.43538583	0.2665193	0.7898393	0.95173872	0.10246126	0.021482262
ATXN2	111.982887	0.45233847	1.69783932	0.26642007	0.78991571	0.95173872	0.10241925	0.021482262
AK9	19.2300352	0.61418266	2.32713722	0.26392198	0.79184004	0.95290343	0.10136254	0.020951111
RPS11	15.0405438	0.45641884	1.7312331	0.263638	0.79205887	0.95290343	0.10124254	0.020951111
ZNF286A	2.84336385	0.75418921	2.86347028	0.26338293	0.79225544	0.95290343	0.10113477	0.020951111
ACTG1	18.317514	0.72460159	2.75975844	0.26255979	0.7928899	0.95339115	0.10078712	0.020728886
NFAT5	301.798022	0.40099363	1.53714143	0.2608697	0.79419299	0.95385625	0.10007395	0.020517072
CD226	197.174278	0.33869598	1.30790678	0.25896033	0.79566585	0.95430668	0.09926928	0.020312037
METTL7A	30.6683965	0.50438648	1.95017163	0.25863697	0.79591535	0.95430668	0.09913312	0.020312037
CD69	16.8377872	0.74812241	2.89296017	0.25860101	0.79594311	0.95430668	0.09911797	0.020312037
CLDN5	186.744228	0.48486513	1.87892494	0.25805455	0.79636481	0.95431706	0.09888794	0.020307311
TRAPPC5	46.2983245	0.47275562	1.8324158	0.25799582	0.79641013	0.95431706	0.09886322	0.020307311
HIST1H1E	38.776189	0.57731667	2.25537721	0.25597344	0.79797134	0.955542	0.0980127	0.01975022

UBR3	7.79375727	0.4707583	1.85309898	0.2540384	0.79946589	0.95660229	0.09720006	0.019268583
SF3A1	15.4614504	0.54429611	2.14882696	0.25329918	0.80003703	0.95677892	0.09688991	0.019188401
MAP3K2	8.79635154	0.53283097	2.1103786	0.25248122	0.80066913	0.95677892	0.09654692	0.019188401
OAZ1	917.346412	0.22759407	0.90617148	0.25116004	0.80169038	0.95677892	0.09599333	0.019188401
PIP4K2C	27.7482669	0.68520485	2.733284	0.25068922	0.80205439	0.95677892	0.09579618	0.019188401
HIST1H3C	34.7324856	0.56369733	2.25074484	0.25044924	0.80223996	0.95677892	0.09569571	0.019188401
CEP162	90.8366841	0.55725884	2.23499058	0.24933387	0.80310254	0.95677892	0.095229	0.019188401
SNRPB2	25.5581955	0.67770747	2.71814621	0.24932709	0.80310778	0.95677892	0.09522617	0.019188401
PPIA	19.7636082	0.58417821	2.34523405	0.24909164	0.80328989	0.95677892	0.0951277	0.019188401
BNIP3	13.1543191	0.70875796	2.85768283	0.24801841	0.80412016	0.95681946	0.09467905	0.01917
RBM3	18.63427	0.63100153	2.55191733	0.24726566	0.80470263	0.95681946	0.09436458	0.01917
CDC73	57.3107098	0.61403373	2.5085156	0.24477971	0.806627	0.9575803	0.09332724	0.018824795
BRMS1	4.24839146	0.71000463	2.90557214	0.24435966	0.80695228	0.9575803	0.09315215	0.018824795
SMDT1	4.74640381	0.70376693	2.9039154	0.24235105	0.80850816	0.95888009	0.09231559	0.018235699
CREBZF	154.636123	0.55305671	2.28504941	0.24203271	0.80875481	0.9588995	0.09218312	0.018226907
MCM3AP	250.977341	0.39800014	1.65362411	0.24068356	0.80980038	0.95905153	0.09162202	0.018158059
KLF10	323.451812	0.63815863	2.65948833	0.23995542	0.81036482	0.95905153	0.09131942	0.018158059
C11orf65	10.1390627	0.6271047	2.62873371	0.23855771	0.81144856	0.95905153	0.09073901	0.018158059
CBX3	39.0877648	0.38421653	1.61123289	0.2384612	0.8115234	0.95905153	0.09069895	0.018158059
ZBTB2	5.81586911	0.69044206	2.90112338	0.23799128	0.81188786	0.95905153	0.09050395	0.018158059
YPEL5	24.5344517	0.51085826	2.14917044	0.23770021	0.81211362	0.95905153	0.0903832	0.018158059
FAM126A	7.16150687	0.61163941	2.58272659	0.23681926	0.81279701	0.95905153	0.09001791	0.018158059
DIAPH1	251.231688	0.4244783	1.79487866	0.23649415	0.81304925	0.95905153	0.08988315	0.018158059
TSPAN9	53.0280378	0.38395938	1.64049494	0.23405094	0.81494544	0.96018199	0.08887147	0.017646445
BET1	25.5289698	0.53806071	2.30340841	0.23359327	0.81530075	0.96018199	0.08868216	0.017646445
KALRN	104.18849	0.43471091	1.86657623	0.23289213	0.81584517	0.96018199	0.08839225	0.017646445
AP1G1	57.7888221	0.48415345	2.07925548	0.23284943	0.81587833	0.96018199	0.0883746	0.017646445
REV3L	98.8857295	0.5654782	2.43836735	0.23190853	0.81660906	0.96028244	0.08798581	0.017601014
GABPB2	3.65360563	0.45937699	1.99110345	0.23071478	0.81753639	0.96071438	0.08749291	0.017405708
LCN2	28.1327086	0.51898809	2.2600235	0.22963836	0.8183728	0.9607601	0.08704881	0.017385039
KIFAP3	216.479034	0.3629852	1.5834125	0.22924235	0.81868056	0.96081901	0.08688552	0.017358413
SCAF4	196.544677	0.383151	1.68753802	0.22704733	0.82038694	0.96084955	0.08598126	0.017344609
HNRNPM	235.424998	0.43871683	1.93805177	0.22637003	0.82091364	0.96084955	0.08570253	0.017344609
SEC31A	6.41894333	0.39238161	1.73612973	0.22600938	0.82119412	0.96084955	0.08555417	0.017344609
GFI1B	349.28509	0.2967577	1.31325072	0.22597186	0.82122331	0.96084955	0.08553873	0.017344609
MED13	137.833125	0.36478266	1.61776849	0.22548508	0.82160193	0.96084955	0.08533855	0.017344609
LYL1	89.9449084	0.26100938	1.16138445	0.22473986	0.82218165	0.96084955	0.08503222	0.017344609
KIDINS220	11.3877272	0.40340601	1.79936884	0.22419306	0.82260708	0.96084955	0.08480756	0.017344609
CTTN	1673.93022	0.22704785	1.01514279	0.223661	0.8230211	0.96084955	0.08458903	0.017344609
CCNL2	144.745199	0.44726041	2.00561092	0.22300457	0.82353195	0.96084955	0.08431955	0.017344609
EPS15L1	61.0648302	0.34814966	1.57201827	0.22146668	0.82472908	0.96112389	0.08368869	0.017220629
PHC3	74.862183	0.38753318	1.7515173	0.22125569	0.82489335	0.96112389	0.0836022	0.017220629
EMP3	25.2132031	0.27681473	1.25693175	0.22023052	0.82569163	0.96125495	0.08318212	0.017161411
ANKRD10	225.518201	0.53261018	2.42292532	0.21982113	0.82601047	0.96125495	0.08301445	0.017161411
SCAF11	46.5257815	0.38420594	1.75219908	0.21927071	0.82643917	0.96125495	0.08278911	0.017161411
SNX19	45.8396512	0.62994625	2.89024382	0.21795609	0.82746332	0.96188884	0.08225125	0.016875115
RBM26	49.3992982	0.48843467	2.24267381	0.21779122	0.82759178	0.96188884	0.08218383	0.016875115
YME1L1	60.1328989	0.44383207	2.04333784	0.21720934	0.8280452	0.96204805	0.08194595	0.016803235
SIN3A	165.532635	0.39381507	1.82205741	0.21613758	0.82888051	0.96234411	0.08150807	0.016669609
KMT2E	377.396792	0.31555622	1.47048905	0.2145927	0.83008488	0.96234411	0.08087749	0.016669609
NENF	4.62589315	0.62034389	2.90444399	0.21358439	0.83087118	0.96234411	0.08046631	0.016669609
PRRC2A	24.2112794	0.4364879	2.04387347	0.21355916	0.83089085	0.96234411	0.08045602	0.016669609
STOX2	2.7670431	0.38494711	1.80909387	0.21278449	0.83149506	0.96234411	0.08014032	0.016669609
SEC14L2	15.4908985	0.41578198	1.95448207	0.21273256	0.83153557	0.96234411	0.08011917	0.016669609
ARF4	151.157224	0.39773538	1.88955691	0.21049135	0.8332842	0.96356441	0.07920685	0.016119251
TPM2	47.9916318	0.43220623	2.06431027	0.20937077	0.83415881	0.96378283	0.07875126	0.016020814
SERPINE1	54.6789285	0.41218059	1.97103511	0.20911885	0.83435546	0.96378283	0.07864889	0.016020814
GLB1	60.8760134	0.56135112	2.69730383	0.20811564	0.83513867	0.96378283	0.07824141	0.016020814
MBD1	16.5537272	0.51875395	2.50376615	0.20718946	0.8358619	0.96386844	0.07786547	0.015982238
PTEN	146.579746	0.35347293	1.71188388	0.20648184	0.83641455	0.9642387	0.07757842	0.015815442
GPAT4	15.9853761	0.49084964	2.38335866	0.20594871	0.83683098	0.9642395	0.07736225	0.01581508
GADD45GIP1	8.72671136	0.53337699	2.59388487	0.20562863	0.83708101	0.9642395	0.07723251	0.01581508

KIAA1143	6.21596764	0.48269034	2.35138493	0.20527917	0.83735402	0.9642395	0.07709089	0.01581508
C21orf58	416.809084	0.28323715	1.38604676	0.20434891	0.83808085	0.9645574	0.07671408	0.015671925
ADAT2	98.1246744	0.41666062	2.05288233	0.20296372	0.83916339	0.96491364	0.07615347	0.015511554
CS	42.4564669	0.49736756	2.45290409	0.20276682	0.83931729	0.96491364	0.07607383	0.015511554
THRA	21.5064161	0.5824343	2.89206991	0.20139012	0.84039354	0.96561648	0.07551729	0.015195333
AUP1	83.9531186	0.41171339	2.05272926	0.20056877	0.84103578	0.96561648	0.07518553	0.015195333
PDS5B	51.2945679	0.42766001	2.14861029	0.19904029	0.84223123	0.96561648	0.07456866	0.015195333
EIF4G2	483.376462	0.35834795	1.80218427	0.1988409	0.8423872	0.96561648	0.07448824	0.015195333
SUN3	6.0706283	0.29460399	1.48337549	0.19860379	0.84257269	0.96561648	0.07439262	0.015195333
PCIF1	18.6318373	0.5097906	2.59358664	0.19655815	0.84417332	0.96672261	0.07356838	0.014698125
UBE3B	209.273741	0.28945208	1.47489374	0.19625283	0.84441228	0.96672261	0.07344546	0.014698125
TAX1BP3	10.8279498	0.4421843	2.25597214	0.1960061	0.84460539	0.96672261	0.07334615	0.014698125
ACAP2	60.2564009	0.40404413	2.07200564	0.19500146	0.8453918	0.96699668	0.07294197	0.014575016
HIST1H4H	2338.16429	0.46356313	2.38683103	0.19421699	0.84600598	0.96699668	0.07262657	0.014575016
GSK3B	9.33720035	0.27817044	1.44129933	0.19299977	0.84695915	0.96761342	0.07213754	0.014298117
HIST1H3A	45.4306066	0.32130861	1.66680531	0.19276913	0.84713978	0.96761342	0.07204492	0.014298117
LACTB2	3.23126079	0.55077344	2.86090135	0.19251745	0.8473369	0.96761342	0.07194388	0.014298117
PSMD11	120.584001	0.43766342	2.28234113	0.19176074	0.84792963	0.96786704	0.07164019	0.014184299
TNIK	351.456695	0.29111787	1.52088238	0.1914138	0.84820141	0.96791194	0.07150101	0.014164152
COPZ1	63.3460226	0.50979144	2.68637905	0.18976899	0.84949016	0.96858626	0.07084165	0.013861697
INPP5A	82.826387	0.33486052	1.77282677	0.18888508	0.85018289	0.96895794	0.07048764	0.013695074
PUM1	11.3607543	0.5449125	2.89513602	0.18821654	0.8507069	0.96895794	0.07022004	0.013695074
RPS27	51.9291071	0.38667617	2.05497932	0.18816548	0.85074693	0.96895794	0.07019961	0.013695074
THUMPD1	140.531138	0.3382284	1.81103851	0.18675937	0.85184931	0.96994752	0.06963723	0.013251764
UBR4	2861.0917	0.19706046	1.06129756	0.18567881	0.85269665	0.96994752	0.06920544	0.013251764
SNRNP35	9.12334396	0.53774147	2.8967379	0.18563691	0.85272951	0.96994752	0.06918871	0.013251764
PLXNB3	206.481516	0.29768276	1.60450058	0.18552985	0.85281347	0.96994752	0.06914595	0.013251764
GSN	17.9498651	0.41961698	2.29849712	0.18256145	0.85514213	0.97086669	0.0679617	0.012840401
PDCD10	45.2398145	0.32598934	1.78803325	0.18231727	0.85533375	0.97086669	0.06786439	0.012840401
TNF	34.7970334	0.42247079	2.32296426	0.18186711	0.85568702	0.97086669	0.06768506	0.012840401
MAPK8	3.65138547	0.38867646	2.15424506	0.18042351	0.8568201	0.97125306	0.06711036	0.012667598
STAB1	1056.67802	0.27400015	1.52653156	0.17949197	0.85755142	0.97125306	0.06673983	0.012667598
SATB1	23.5308416	0.37610598	2.10221878	0.17890906	0.85800911	0.97125306	0.0665081	0.012667598
PPP1R12A	399.994535	0.27824789	1.56240555	0.17808941	0.85865276	0.97125306	0.06618243	0.012667598
PSMB8	313.577329	0.47713619	2.68771868	0.1775246	0.85909634	0.97125306	0.06595813	0.012667598
TMEM50A	131.946708	0.45941429	2.59479423	0.1770523	0.85946731	0.97125306	0.06577064	0.012667598
DNAH12	7.55370255	0.19387287	1.09675758	0.17676912	0.85968975	0.97125306	0.06565825	0.012667598
SUMO2	18.3397711	0.39029388	2.20796187	0.17676659	0.85969174	0.97125306	0.06565725	0.012667598
BNIP2	133.513138	0.30752388	1.74122902	0.17661311	0.85981229	0.97125306	0.06559635	0.012667598
NRIP1	18.5083132	0.42215352	2.39229187	0.17646405	0.85992939	0.97125306	0.06553721	0.012667598
SDC4	19.2342493	0.41647809	2.36179749	0.17633946	0.86002726	0.97125306	0.06548778	0.012667598
MAST4	737.382375	0.22387919	1.27012934	0.17626487	0.86008586	0.97125306	0.06545819	0.012667598
EIF3E	3.68989643	0.51235578	2.90862862	0.17615029	0.86017587	0.97125306	0.06541274	0.012667598
C19orf33	13.667245	0.43486401	2.48179038	0.17522189	0.86090528	0.97125306	0.06504463	0.012667598
GSE1	413.372057	0.25929648	1.47983259	0.17522015	0.86090666	0.97125306	0.06504393	0.012667598
EXOSC4	7.98827396	0.45099237	2.57421948	0.17519577	0.86092581	0.97125306	0.06503427	0.012667598
KCTD13	30.9614247	0.39072827	2.23843923	0.17455389	0.86143018	0.97155885	0.06477991	0.012530886
PRKCD	388.29264	0.27165523	1.55893468	0.17425697	0.86166352	0.97155886	0.0646623	0.012530882
DGKZ	39.3583929	0.3036347	1.74979598	0.17352577	0.86223817	0.97191788	0.06437275	0.012370428
ESRRB	7.53822649	0.38883318	2.24424553	0.17325786	0.86244874	0.97191788	0.06426671	0.012370428
STK11	133.405896	0.23630937	1.36965134	0.1725325	0.86301891	0.97203652	0.06397969	0.012317416
CUL9	4.77915134	0.16923973	0.99080918	0.17080961	0.86437347	0.97203652	0.06329857	0.012317416
PRUNE1	28.8915848	0.4938288	2.89122055	0.17080288	0.86437876	0.97203652	0.06329591	0.012317416
MAPKAPK2	7.66257338	0.44190742	2.59088128	0.17056259	0.86456772	0.97203652	0.06320099	0.012317416
ASB8	26.5225301	0.39920531	2.34204445	0.17045164	0.86465497	0.97203652	0.06315716	0.012317416
SCN1A	9.46455037	0.44051914	2.60991391	0.16878685	0.86596431	0.97213278	0.06250001	0.012274413
PTBP3	32.0275565	0.41116018	2.43794786	0.16865011	0.86607186	0.97213278	0.06244607	0.012274413
EXT2	100.451967	0.45633958	2.71111948	0.16832146	0.86633039	0.97213278	0.06231645	0.012274413
SOS1	111.354973	0.33977965	2.01931288	0.16826499	0.86637482	0.97213278	0.06229418	0.012274413
MMRN1	6.25472182	0.37023667	2.21466525	0.16717501	0.86723235	0.97267004	0.06186453	0.012034462
ABLIM3	170.065454	0.32295823	1.93831639	0.16661791	0.8676707	0.97267004	0.06164507	0.012034462
SIRT3	15.0207523	0.33543096	2.02948072	0.1652792	0.86872423	0.97267004	0.06111807	0.012034462

MCM7	261.612837	0.38683392	2.37123591	0.16313599	0.87041136	0.97374455	0.06027545	0.01155496
GSTO1	76.1660792	0.27074475	1.66837714	0.16228031	0.87108512	0.97374455	0.0599394	0.01155496
PHB2	111.276656	0.44169015	2.73830393	0.16130063	0.87185663	0.9738238	0.05955493	0.011519615
GSTM2	6.9860771	0.46764798	2.89925485	0.16129937	0.87185762	0.9738238	0.05955443	0.011519615
SDCCAG8	5.61195836	0.31115111	1.94700092	0.15981046	0.87303038	0.97487222	0.05897064	0.011052303
RHOA	57.3773331	0.36867163	2.33109801	0.15815364	0.87433573	0.9750799	0.05832177	0.010959797
FUBP1	151.678371	0.29816553	1.88787133	0.15793742	0.87450611	0.9750799	0.05823715	0.010959797
BSDC1	36.2703469	0.31577781	2.00280506	0.15766777	0.8747186	0.9750799	0.05813164	0.010959797
GALM	3.45823723	0.38530934	2.48009108	0.15536096	0.87653676	0.9750799	0.05722987	0.010959797
VSIR	342.340676	0.24888626	1.60967919	0.15461855	0.87712205	0.9750799	0.05693997	0.010959797
TMBIM1	1029.54348	0.22604589	1.47344855	0.15341282	0.87807274	0.9750799	0.05646951	0.010959797
XRN1	81.1399391	0.29553913	1.92913777	0.15319752	0.87824251	0.9750799	0.05638554	0.010959797
PFN1	68.0560044	0.28631588	1.87483291	0.15271541	0.87862271	0.9750799	0.05619758	0.010959797
EP400	10.8948194	0.31866204	2.08810168	0.15260849	0.87870703	0.9750799	0.0561559	0.010959797
FBXO11	26.9304357	0.36574123	2.40107073	0.15232422	0.87893122	0.9750799	0.05604511	0.010959797
HTATIP2	4.83976549	0.41908867	2.75776775	0.15196663	0.87921326	0.9750799	0.05590577	0.010959797
FARP1	4.5306378	0.28808314	1.89634364	0.15191505	0.87925394	0.9750799	0.05588568	0.010959797
PACSIN2	3.12985493	0.29536541	1.94510514	0.15185062	0.87930476	0.9750799	0.05586058	0.010959797
ARHGAP5	37.4632554	0.39480501	2.612675	0.15111141	0.87988783	0.9754667	0.05557269	0.010787553
ERV3.1	320.664439	0.18907709	1.26895566	0.14900212	0.88155196	0.9762719	0.05475209	0.01042921
MYO15B	257.389411	0.17854298	1.20704058	0.14791796	0.88240751	0.97681203	0.0543308	0.010189
SRC	121.280005	0.31416827	2.12578124	0.14778956	0.88250885	0.97681203	0.05428093	0.010189
RBM14	15.8949004	0.34447	2.34283247	0.14703143	0.88310722	0.97697793	0.05398657	0.010115245
FAM78A	20.3136589	0.34609112	2.36302214	0.14646123	0.8835573	0.97697793	0.05376528	0.010115245
PIH1D1	63.0656417	0.24520138	1.69032989	0.14506126	0.88466251	0.97697793	0.05322238	0.010115245
SFT2D1	54.1907332	0.33691181	2.33195395	0.14447618	0.88512446	0.97697793	0.05299566	0.010115245
SUSD6	65.8985423	0.36401436	2.52212843	0.14432824	0.88524128	0.97697793	0.05293834	0.010115245
CAPZA2	234.163447	0.28463334	1.98489937	0.14339938	0.88597477	0.97697793	0.05257864	0.010115245
DENND2C	57.502408	0.21472227	1.49852697	0.1432889	0.88606203	0.97697793	0.05253588	0.010115245
HIST1H2AG	672.595698	0.18046962	1.26312851	0.14287511	0.88638882	0.97697793	0.05237573	0.010115245
CABIN1	22.1017199	0.2508834	1.76207039	0.1423799	0.88677994	0.97697793	0.05218414	0.010115245
SUN2	18.3200216	0.29329198	2.06421623	0.14208394	0.8870137	0.97697793	0.05206967	0.010115245
TNRC6A	88.3118233	0.23405852	1.65074844	0.14178933	0.88724641	0.97697793	0.05195575	0.010115245
RMND5B	2.88170537	0.4131181	2.91372786	0.14178335	0.88725113	0.97697793	0.05195344	0.010115245
DNAJB6	112.736353	0.20523133	1.45315563	0.14123148	0.88768707	0.97697793	0.0517401	0.010115245
CAPN3	2.7630337	0.34922141	2.48379208	0.1406001	0.88818587	0.97697793	0.05149614	0.010115245
ERBIN	86.4328689	0.27029681	1.92442688	0.14045574	0.88829992	0.97697793	0.05144038	0.010115245
NFKBIA	382.992546	0.25315092	1.81060494	0.13981566	0.88880564	0.97702922	0.0511932	0.010092447
TCEAL9	26.6683087	0.28531714	2.05844602	0.13860802	0.8897599	0.97726004	0.05072717	0.009989859
TNS3	3.45317108	0.30432164	2.19889816	0.13839733	0.88992641	0.97726004	0.05064591	0.009989859
KLF3	6.18817335	0.295447	2.1353201	0.13836192	0.88995439	0.97726004	0.05063225	0.009989859
POLR2E	88.876257	0.23365584	1.70375925	0.13714135	0.89091907	0.97779196	0.05016175	0.00975354
VPS28	257.998291	0.18305842	1.34115935	0.13649267	0.89143182	0.97779196	0.04991187	0.00975354
BCLAF1	91.581933	0.23389559	1.71649333	0.13626362	0.89161289	0.97779196	0.04982366	0.00975354
MPPED2	20.5251573	0.34085452	2.51506393	0.13552519	0.89219665	0.97783927	0.04953941	0.009732526
ARGLU1	280.311434	0.22604241	1.66908391	0.13542903	0.89227268	0.97783927	0.04950241	0.009732526
HTRA2	67.4209567	0.2964196	2.19054221	0.13531791	0.89236052	0.97783927	0.04945965	0.009732526
PNN	54.0762554	0.38409424	2.89003791	0.13290284	0.89427022	0.97890147	0.04853123	0.009261018
IFRD1	233.037584	0.18708745	1.42310491	0.13146427	0.89540805	0.97922207	0.04797901	0.009118806
C20orf96	113.328231	0.2877914	2.18971242	0.13142886	0.89543606	0.97922207	0.04796542	0.009118806
NRBP1	126.480507	0.25881186	1.98113347	0.13063827	0.89606146	0.97922207	0.0476622	0.009118806
SPSB3	20.7937391	0.31380274	2.40931539	0.13024561	0.89637211	0.97922207	0.04751166	0.009118806
RADIL	17.4074379	0.2813923	2.17402954	0.12943352	0.89701462	0.97922207	0.04720048	0.009118806
CREB3L2	100.328791	0.2495189	1.9295673	0.1293134	0.89710967	0.97922207	0.04715446	0.009118806
HMGCR	9.74066316	0.33362911	2.59857294	0.12838936	0.89784085	0.9794289	0.04680064	0.009027085
EIF4ENIF1	11.3470122	0.24471704	1.90781565	0.1282708	0.89793467	0.9794289	0.04675526	0.009027085
CASP6	48.6898256	0.29971634	2.35224599	0.12741709	0.89861029	0.97962318	0.04642861	0.008940948
JMJD6	26.821769	0.27454504	2.15647912	0.12731171	0.89869369	0.97962318	0.04638831	0.008940948
HIST1H2BL	16.7279332	0.28414596	2.25159262	0.12619776	0.89957538	0.98032763	0.04596244	0.008628755
ERP44	44.3502188	0.27549874	2.22117147	0.12403308	0.90128908	0.98168133	0.04513589	0.008029467
IFT88	11.732716	0.30829173	2.52149067	0.12226566	0.90268862	0.98213474	0.04446203	0.007828929
SCYL1	31.7199422	0.27465914	2.2702535	0.1209817	0.90370553	0.98213474	0.04397306	0.007828929

PARP12	17.8674866	0.32919378	2.72374677	0.12086064	0.90380142	0.98213474	0.04392698	0.007828929
RYBP	197.179086	0.22516922	1.86356827	0.12082693	0.90382812	0.98213474	0.04391415	0.007828929
PWWP2A	14.4871841	0.26021148	2.17656521	0.11955143	0.9048385	0.98297613	0.04342893	0.007457028
STOML2	13.2338341	0.30502961	2.5676185	0.11879865	0.90543488	0.98299593	0.04314278	0.007448282
WIPF1	34.1991925	0.2264011	1.91061885	0.11849621	0.9056745	0.98299593	0.04302786	0.007448282
MTMR3	7.52947499	0.33022321	2.79054285	0.11833655	0.905801	0.98299593	0.0429672	0.007448282
ZNF317	14.5572499	0.33900811	2.89385715	0.11714749	0.90674318	0.98324963	0.0425157	0.007336208
VEZF1	88.7520702	0.31889596	2.74531764	0.11615995	0.90752578	0.98335557	0.04214103	0.007289419
CYB5R1	107.212447	0.21703971	1.86900299	0.11612593	0.90755274	0.98335557	0.04212813	0.007289419
STAT1	49.6734992	0.33050929	2.89017359	0.11435621	0.90895543	0.98395758	0.04145741	0.007023623
FAM19A2	3.0610646	0.2627841	2.30034717	0.11423671	0.90905015	0.98395758	0.04141216	0.007023623
RBMX	13.4469332	0.24944552	2.19101806	0.11384914	0.90935738	0.98403434	0.0412654	0.006989745
FHOD1	220.748219	0.16597444	1.47116784	0.11281816	0.91017472	0.98440716	0.04087523	0.006825238
C2orf88	338.23532	0.18821778	1.68521265	0.11168785	0.91107091	0.98482621	0.04044782	0.0066404
ARID4B	183.432821	0.1828551	1.65911224	0.11021261	0.91224076	0.98482621	0.03989053	0.0066404
ANKLE2	34.7006719	0.25621341	2.3251778	0.11019089	0.91225799	0.98482621	0.03988233	0.0066404
RUNX1	7.74226248	0.30927886	2.82768234	0.10937539	0.91290475	0.98482621	0.03957453	0.0066404
TUT1	10.7677156	0.2543622	2.3315021	0.10909799	0.91312477	0.98482621	0.03946988	0.0066404
USP1	2.97226238	0.28746387	2.6443273	0.10870964	0.9134328	0.98482621	0.0393234	0.0066404
C2orf42	3.91434838	0.31139537	2.90784132	0.10708816	0.91471904	0.98482621	0.03871228	0.0066404
KLHL12	29.4496259	0.29538824	2.76899924	0.1066769	0.91504531	0.98482621	0.0385574	0.0066404
CAPZB	29.1197911	0.20461034	1.94618434	0.1051341	0.9162694	0.98482621	0.03797682	0.0066404
C14orf166	19.8925252	0.26721807	2.54259553	0.10509657	0.91629918	0.98482621	0.0379627	0.0066404
NSUN2	6.35563648	0.29074039	2.76838758	0.10502156	0.9163587	0.98482621	0.03793449	0.0066404
JUNB	338.609316	0.15348945	1.47537491	0.1040342	0.91714221	0.98485475	0.03756332	0.006627818
RAP2B	308.942302	0.18917194	1.83305563	0.10320033	0.91780398	0.98485475	0.03725006	0.006627818
SMIM10L1	87.7379204	0.26255933	2.55411825	0.10279842	0.91812296	0.98485475	0.03709915	0.006627818
TTLL3	74.4554891	0.17275621	1.68188071	0.10271609	0.91818831	0.98485475	0.03706824	0.006627818
HMG20B	221.655098	0.15398053	1.51278427	0.10178618	0.91892639	0.98485475	0.03671927	0.006627818
AHDC1	6.35125813	0.27395666	2.76328296	0.09914173	0.92102574	0.98485475	0.03572823	0.006627818
SRSF10	147.005616	0.27161192	2.75233134	0.09868431	0.92138893	0.98485475	0.03555701	0.006627818
ATP6V1D	3.2735044	0.26450127	2.70116724	0.0979211	0.92199495	0.98485475	0.03527146	0.006627818
SF3B2	60.4585886	0.22946616	2.34631595	0.09779849	0.92209231	0.98485475	0.0352256	0.006627818
HIST1H2AE	104.586504	0.15700493	1.61009403	0.09751289	0.92231909	0.98485475	0.0351188	0.006627818
PAN3	372.026941	0.17852108	1.83423913	0.09732705	0.92246668	0.98485475	0.03504931	0.006627818
EPHX2	7.67957169	0.20257779	2.08605883	0.09711029	0.92263881	0.98485475	0.03496828	0.006627818
DIP2A	297.410006	0.2028809	2.09597437	0.0967955	0.92288879	0.98485475	0.03485063	0.006627818
TNFRSF14	333.769734	0.14671	1.51843177	0.09661942	0.92302863	0.98485475	0.03478483	0.006627818
TNS1	974.952332	0.13837919	1.44083503	0.09604097	0.92348803	0.98485475	0.03456873	0.006627818
MAPRE2	51.5002046	0.17334331	1.83128842	0.09465648	0.92458769	0.98485475	0.03405189	0.006627818
DAB2	410.010519	0.11297777	1.19450386	0.09458134	0.92464737	0.98485475	0.03402386	0.006627818
NIPBL	179.939415	0.16031664	1.6993176	0.09434177	0.92483767	0.98485475	0.03393449	0.006627818
SEC14L1	560.218805	0.10401342	1.10466961	0.09415795	0.92498369	0.98485475	0.03386593	0.006627818
UQCRB	6.92159992	0.27278352	2.89985078	0.09406812	0.92505504	0.98485475	0.03383243	0.006627818
MECP2	76.7386892	0.17335531	1.85789131	0.09330756	0.92565922	0.98499748	0.03354887	0.006564879
OSBPL2	57.1419607	0.2100821	2.27998867	0.09214173	0.92658543	0.9855009	0.03311453	0.006342973
NXF1	218.294543	0.19583555	2.14634715	0.09124132	0.92730084	0.9855009	0.03277935	0.006342973
JKAMP	60.6637415	0.22478843	2.47835043	0.09070082	0.92773032	0.9855009	0.03257825	0.006342973
ADIPOR1	247.718844	0.13672922	1.50794979	0.09067226	0.92775301	0.9855009	0.03256763	0.006342973
XPO1	368.235656	0.15528431	1.73227452	0.08964186	0.92857182	0.98586768	0.0321845	0.00618137
P2RX1	1085.0344	0.10361658	1.1859488	0.0873702	0.93037726	0.98677812	0.03134091	0.005780489
FLNA	285.756575	0.13042722	1.54977211	0.08415897	0.93293004	0.98851893	0.03015092	0.005015008
TRA2A	87.0551701	0.13913332	1.66947562	0.08333954	0.93358156	0.98851893	0.02984773	0.005015008
HERC5	2.88975968	0.23902956	2.86830562	0.08333476	0.93358536	0.98851893	0.02984597	0.005015008
HIST1H4D	4.79011055	0.18733163	2.24951001	0.08327664	0.93363157	0.98851893	0.02982447	0.005015008
TMEM260	5.93418411	0.21217856	2.55893518	0.08291674	0.93391774	0.98851893	0.02969137	0.005015008
WSB1	350.589092	0.13921368	1.69429852	0.08216597	0.93451473	0.98889946	0.02941385	0.004847862
PLEKHF2	46.267728	0.21388504	2.6261428	0.08144456	0.93508842	0.98909081	0.02914732	0.004763831
UBE2M	8.63076877	0.16803004	2.08965508	0.08041042	0.93591084	0.98918168	0.02876552	0.004723937
UBAP2L	64.7837273	0.18655831	2.37563659	0.07852982	0.93740661	0.99028666	0.02807199	0.004239073
SOX4	53.6485454	0.18039919	2.31702718	0.07785804	0.93794098	0.99028666	0.02782449	0.004239073
DNAJC9	3.29641592	0.15509548	2.03597292	0.07617758	0.93927782	0.99061941	0.02720593	0.004093166

ELP2	5.406694	0.17932919	2.37238352	0.0755903	0.93974505	0.99061941	0.02698995	0.004093166
SOCS2	192.283172	0.16833317	2.23124052	0.07544376	0.93986165	0.99061941	0.02693607	0.004093166
CD55	148.145344	0.15202494	2.02806883	0.07496045	0.94024618	0.99061941	0.02675842	0.004093166
STARD8	297.542232	0.12018577	1.60964402	0.07466605	0.94048042	0.99061941	0.02665024	0.004093166
HIST1H4I	94.0867281	0.13849166	1.90002878	0.07288925	0.94189425	0.99142999	0.02599785	0.003737948
DDX3X	521.852056	0.12363	1.7028109	0.07260348	0.94212166	0.99142999	0.02589301	0.003737948
OSBPL8	44.9785614	0.16776924	2.3152981	0.07246118	0.9422349	0.99142999	0.02584082	0.003737948
EFEMP2	41.5340067	0.17427715	2.4415946	0.07137841	0.94309659	0.99142999	0.02544383	0.003737948
ZBTB44	20.6930635	0.19128451	2.78228398	0.06875089	0.94518791	0.9928765	0.02448184	0.003104768
ZNF335	37.3256169	0.16547199	2.46041995	0.06725356	0.94637985	0.99312644	0.02393451	0.002995458
BNIP1	11.2644322	0.1871395	2.81139574	0.06656462	0.94692831	0.99326607	0.0236829	0.002934402
ARAP1	912.86413	0.11156616	1.67801068	0.06648716	0.94698998	0.99326607	0.02365462	0.002934402
CDKL1	65.4105826	0.10702631	1.63096171	0.0656216	0.94767909	0.99373854	0.0233387	0.002727867
ZCCHC3	18.6468698	0.14188235	2.2413602	0.0633019	0.94952611	0.99492368	0.02249309	0.002210232
BIRC6	192.912801	0.0938615	1.53714012	0.06106243	0.95130949	0.99543999	0.02167817	0.001984915
DENND3	432.636917	0.07876126	1.2938105	0.06087543	0.95145842	0.99543999	0.02161019	0.001984915
NKTR	134.863165	0.11975943	2.05087269	0.05839438	0.95343449	0.99650455	0.02070914	0.001520714
OTOGL	13.3865325	0.13606777	2.34322703	0.05806854	0.95369404	0.99650455	0.02059093	0.001520714
TLN1	1411.69537	0.06085268	1.06572468	0.05709981	0.95446569	0.99658517	0.02023968	0.001485578
RAB11FIP3	59.3515382	0.09809535	1.76540163	0.05556546	0.95568798	0.99658517	0.01968387	0.001485578
COMMD7	35.789669	0.09903225	1.78795515	0.05538856	0.95582891	0.99658517	0.01961984	0.001485578
SDPR	215.669832	0.07732162	1.43669869	0.05381896	0.95707941	0.99731698	0.01905203	0.001166787
FNBP1	7.91250187	0.13304403	2.497644	0.05326781	0.95751853	0.99752493	0.01885281	0.001076242
PROSER2	14.4054913	0.1089251	2.08155213	0.05232879	0.95826671	0.99762923	0.0185136	0.001030833
VTI1B	9.5297526	0.14959007	2.89685879	0.05163872	0.95881657	0.99762923	0.01826447	0.001030833
GTF2B	18.4329031	0.1029467	2.02441383	0.05085259	0.95944298	0.99766907	0.01798083	0.001013492
IGIP	7.69927183	0.14659724	2.89875757	0.05057244	0.95966623	0.99766907	0.01787979	0.001013492
PHIP	349.498169	0.08424187	1.70976475	0.04927103	0.96070331	0.99766907	0.01741071	0.001013492
TROVE2	48.3328083	0.11687897	2.44284199	0.04784549	0.96183938	0.99766907	0.01689744	0.001013492
X7.Mar	57.4781372	0.13239001	2.81359133	0.04705375	0.96247039	0.99766907	0.01661262	0.001013492
ZNF606	7.1857365	0.13589262	2.89949088	0.04686775	0.96261863	0.99766907	0.01654574	0.001013492
C6orf62	362.586036	0.07590914	1.63475841	0.04643447	0.96296396	0.99766907	0.01638997	0.001013492
HES1	5.31056588	0.11434267	2.52006792	0.04537285	0.96381012	0.99766907	0.01600852	0.001013492
MCTP1	73.6944122	0.06876234	1.51810018	0.045295	0.96387218	0.99766907	0.01598056	0.001013492
SRSF6	874.272615	0.08424689	1.90515328	0.04422053	0.96472861	0.99766907	0.01559484	0.001013492
STK4	73.6585122	0.07936466	1.80190745	0.0440448	0.96486869	0.99766907	0.01553179	0.001013492
USP22	160.732765	0.0659591	1.51272164	0.04360294	0.96522091	0.99766907	0.01537328	0.001013492
PDCD6IP	55.8448988	0.09966579	2.28976817	0.04352659	0.96528177	0.99766907	0.0153459	0.001013492
DDX39B	30.3537974	0.08739241	2.01100946	0.04345699	0.96533725	0.99766907	0.01532093	0.001013492
AMPD2	281.567423	0.07761479	1.81806402	0.0426909	0.96594793	0.99766907	0.01504628	0.001013492
RNF44	60.5864404	0.09311842	2.22339012	0.04188128	0.96659334	0.99766907	0.0147562	0.001013492
FNIP1	208.338442	0.07413365	1.82765153	0.04056225	0.96764488	0.99766907	0.014284	0.001013492
CREBRF	627.667319	0.06742636	1.71992585	0.03920306	0.96872849	0.99766907	0.01379793	0.001013492
GPX1	136.83723	0.05561473	1.44661261	0.0384448	0.96933304	0.99766907	0.01352698	0.001013492
RSBN1L	37.0450057	0.08545457	2.22813972	0.03835243	0.96940669	0.99766907	0.01349399	0.001013492
RP11.211G3.2	8.74950986	0.0985849	2.57319353	0.03831228	0.9694387	0.99766907	0.01347965	0.001013492
QKI	49.1427228	0.07493708	2.05446888	0.03647516	0.97090348	0.99766907	0.01282394	0.001013492
NPIPB4	25.0323648	0.04774658	1.32671502	0.03598857	0.97129147	0.99766907	0.01265042	0.001013492
PRPF38B	23.9994105	0.07955979	2.24172279	0.03549047	0.97168865	0.99766907	0.01247287	0.001013492
ELL2	5.28696086	0.08926415	2.53619886	0.03519604	0.97192342	0.99766907	0.01236795	0.001013492
SLC39A9	75.8667181	0.07902006	2.24767524	0.03515635	0.97195507	0.99766907	0.01235381	0.001013492
TOM1L2	160.138124	0.06535973	1.86630082	0.035021	0.97206299	0.99766907	0.01230559	0.001013492
CA13	80.9378652	0.06854318	1.97153103	0.03476647	0.97226595	0.99766907	0.01221492	0.001013492
ITPR1	234.530604	0.06455074	1.87861789	0.03436076	0.97258947	0.99766907	0.01207043	0.001013492
CENPT	282.830753	0.05245879	1.54408856	0.03397395	0.97289792	0.99766907	0.01193272	0.001013492
AKAP13	154.753717	0.06457402	1.94912346	0.03312978	0.9735711	0.99766907	0.01163233	0.001013492
HEXIM2	24.8034101	0.05228082	1.57915134	0.03310691	0.97358933	0.99766907	0.01162419	0.001013492
SRSF3	621.000575	0.05741488	1.74399665	0.03292144	0.97373724	0.99766907	0.01155822	0.001013492
SEC23IP	12.6666943	0.08569828	2.69273906	0.03182569	0.97461106	0.99766907	0.01116866	0.001013492
ENO1	18.0833467	0.07413458	2.33565469	0.03174039	0.97467909	0.99766907	0.01113835	0.001013492
LCAT	4.84521092	0.044827	1.41443521	0.03169251	0.97471727	0.99766907	0.01112134	0.001013492
ABCC3	1744.09523	0.04169462	1.33810635	0.03115942	0.9751424	0.99766907	0.01093196	0.001013492

SRSF4	173.833372	0.06964033	2.24811676	0.03097718	0.97528774	0.99766907	0.01086724	0.001013492
PRPF8	82.4116872	0.07160106	2.38325892	0.03004334	0.97603249	0.99766907	0.01053573	0.001013492
PGM2L1	36.5753583	0.05566122	1.90225776	0.02926061	0.97665674	0.99766907	0.01025805	0.001013492
CDK11B	6.23926634	0.06037665	2.07834164	0.02905039	0.9768244	0.99766907	0.0101835	0.001013492
ITGAX	498.825497	0.06732176	2.37982611	0.02828852	0.97743204	0.99804488	0.00991343	0.000849931
STAT3	310.869066	0.05510189	1.99593679	0.02760703	0.97797557	0.99807098	0.00967199	0.00083857
SNX1	16.8703818	0.05935582	2.37713202	0.02496951	0.98007928	0.99807098	0.00873879	0.00083857
ETV6	164.016133	0.04638176	2.00649535	0.02311581	0.9815579	0.99807098	0.00808408	0.00083857
CAPZA1	167.222269	0.05845405	2.64952365	0.0220621	0.98239842	0.99807098	0.00771234	0.00083857
UBA3	35.7154126	0.0562581	2.56222916	0.0219567	0.98248249	0.99807098	0.00767518	0.00083857
NFX1	119.52624	0.03752037	1.76227019	0.02129093	0.98301358	0.99807098	0.00744048	0.00083857
SLC30A7	72.964389	0.05289983	2.5160015	0.02102536	0.98322543	0.99807098	0.0073469	0.00083857
ITPKB	178.734822	0.04256353	2.0756453	0.02050617	0.98363959	0.99807098	0.007164	0.00083857
KTN1	48.1939198	0.03231574	1.61871128	0.01996387	0.9840722	0.99807098	0.00697304	0.00083857
ITGA2B	3804.7844	0.02285145	1.14596015	0.01994088	0.98409053	0.99807098	0.00696495	0.00083857
NBPF26	3.33220603	0.03765501	1.88926072	0.01993108	0.98409835	0.99807098	0.0069615	0.00083857
NDUFA6	106.524331	0.03548424	1.79852767	0.01972961	0.98425907	0.99807098	0.00689057	0.00083857
RBSN	18.8616481	0.04802045	2.43689218	0.01970561	0.98427821	0.99807098	0.00688213	0.00083857
RAB10	109.291106	0.04023291	2.14241864	0.0187792	0.98501724	0.99807098	0.00655617	0.00083857
HDAC9	30.4752671	0.04337285	2.31829473	0.01870895	0.98507329	0.99807098	0.00653146	0.00083857
CHD1	62.9229296	0.04979053	2.67105944	0.01864074	0.9851277	0.99807098	0.00650747	0.00083857
MT.CO2	667.795698	0.02359433	1.37993789	0.01709811	0.98635835	0.99824838	0.00596528	0.000761384
IFT57	52.4681077	0.04410905	2.64745111	0.01666095	0.9867071	0.99824838	0.00581175	0.000761384
HAUS4	2.88166144	0.03142894	1.88803206	0.0166464	0.98671871	0.99824838	0.00580664	0.000761384
HIPK2	272.977565	0.02035564	1.22710969	0.01658828	0.98676507	0.99824838	0.00578623	0.000761384
MANBA	48.1547951	0.03537161	2.15441457	0.0164182	0.98690076	0.99824838	0.00572652	0.000761384
CASP2	163.042493	0.04207538	2.67393381	0.01573538	0.9874455	0.99824838	0.00548687	0.000761384
STX7	112.373494	0.03659199	2.33038965	0.01570209	0.98747206	0.99824838	0.00547518	0.000761384
NPAT	14.7822208	0.03140705	2.1328062	0.01472569	0.98825102	0.99824838	0.00513273	0.000761384
CHRNA2	156.451051	0.03405642	2.35782086	0.01444402	0.98847574	0.99824838	0.00503399	0.000761384
BBX	84.9161072	0.02657291	2.02443941	0.01312606	0.98952722	0.9982633	0.00457225	0.000754895
RPS17	63.2390471	0.03445851	2.64933709	0.01300646	0.98962264	0.9982633	0.00453038	0.000754895
FADS2	64.5059466	0.02785356	2.34190667	0.01189354	0.99051055	0.9985122	0.00414089	0.000646626
SCAP	6.29577643	0.02355979	2.01580684	0.01168752	0.99067492	0.9985122	0.00406883	0.000646626
NPTN	40.9103633	0.026046	2.26837596	0.01148222	0.99083871	0.9985122	0.00399703	0.000646626
DYSF	9.80947091	0.03210308	2.89677393	0.01108236	0.99115774	0.99853656	0.00385722	0.000636029
DDX17	452.945296	0.01313636	1.21063688	0.01085079	0.99134249	0.99853656	0.00377628	0.000636029
PURA	48.8377053	0.01990505	2.01934367	0.00985719	0.99213523	0.99862935	0.00342913	0.000595676
ARID1A	540.835461	0.01249373	1.31941916	0.00946911	0.99244486	0.99862935	0.00329362	0.000595676
ARL6IP5	3.04691501	0.02564353	2.87303044	0.0089256	0.99287849	0.99862935	0.0031039	0.000595676
TRIP12	141.076218	0.01976532	2.31378457	0.00854242	0.99318422	0.99862935	0.00297019	0.000595676
FTL	118.351719	0.01174525	1.45880618	0.00805127	0.99357608	0.99862935	0.00279887	0.000595676
ARHGAP6	125.21024	0.0135167	1.77444727	0.00761741	0.99392224	0.99862935	0.00264759	0.000595676
FTO	2.74305702	0.00771399	1.2181442	0.00633258	0.99494737	0.99862935	0.00219989	0.000595676
UBN2	3.34837136	0.01200204	2.32580716	0.00516038	0.99588263	0.99862935	0.00179184	0.000595676
MT.ATP8	63.8469338	0.0058456	1.17420755	0.00497834	0.99602788	0.99862935	0.00172851	0.000595676
MSN	70.7055946	0.00594151	1.31234324	0.00452741	0.99638766	0.99862935	0.00157166	0.000595676
SLAIN2	7.10113912	0.01013032	2.27431029	0.00445424	0.99644604	0.99862935	0.00154621	0.000595676
SP1	70.4821401	0.01111593	2.67595018	0.00415401	0.99668559	0.99862935	0.00144182	0.000595676
SETD5	81.4779772	0.00782792	2.0481766	0.0038219	0.99695057	0.99862935	0.00132637	0.000595676
MAML1	104.375317	0.00654129	2.57410908	0.00254118	0.99797243	0.99911785	0.00088146	0.000383284
HERC1	3100.43296	0.00181236	0.85629732	0.00211651	0.99831127	0.99911785	0.00073402	0.000383284
TCEAL8	3.88834884	0.00496572	2.90893058	0.00170706	0.99863796	0.99911785	0.00059193	0.000383284
MTURN	80.7505064	-0.00010358	1.64000162	-6.3157E-05	0.99994961	0.99994961	-2.1886E-05	2.18856E-05
ARMCX3	112.774094	-0.00076458	2.63048329	-0.00029066	0.99976809	0.99994961	-0.00010073	2.18856E-05
BEST1	3.47531046	-0.00370405	1.94397379	-0.0019054	0.99847971	0.99911785	-0.00066076	0.000383284
MTSS1	242.706524	-0.00501891	2.05333496	-0.00244427	0.99804975	0.99911785	-0.00084781	0.000383284
RPL13A	94.198746	-0.00919271	2.24831801	-0.00408871	0.99673769	0.99862935	-0.00141912	0.000595676
TSPYL1	89.3226466	-0.01000184	2.41130179	-0.0041479	0.99669046	0.99862935	-0.0014397	0.000595676
ENKD1	9.22272012	-0.01141146	2.30383288	-0.00495325	0.99604789	0.99862935	-0.00171978	0.000595676
HIST4H4	3.72295897	-0.01187835	2.32999531	-0.00509802	0.99593239	0.99862935	-0.00177014	0.000595676
HPN	3.68046578	-0.01224261	2.39988733	-0.00510133	0.99592975	0.99862935	-0.0017713	0.000595676

STK17B	31.142591	-0.01306715	2.3611965	-0.00553412	0.99558443	0.99862935	-0.0019219	0.000595676
PCP2	52.9562013	-0.01006347	1.28629323	-0.00782362	0.99375772	0.99862935	-0.00271948	0.000595676
TRIM24	372.813404	-0.01628322	1.96910486	-0.00826935	0.99340209	0.99862935	-0.00287493	0.000595676
TRIM58	29.6918364	-0.01400725	1.68683911	-0.00830385	0.99337456	0.99862935	-0.00288696	0.000595676
TC2N	21.3178436	-0.01758757	2.10083146	-0.00837172	0.99332041	0.99862935	-0.00291064	0.000595676
ZNF678	11.2997519	-0.02169773	1.70938993	-0.01269326	0.98987252	0.9982633	-0.00442073	0.000754895
FAM69B	489.448054	-0.02076269	1.63244753	-0.01271875	0.98985218	0.9982633	-0.00442966	0.000754895
X5.Sep	176.873098	-0.01999986	1.4374174	-0.01391375	0.9888988	0.99824838	-0.00484815	0.000761384
DHX9	153.591443	-0.03288839	2.33342479	-0.01409447	0.98875461	0.99824838	-0.00491148	0.000761384
NFKB2	4.62891407	-0.04277831	2.90598594	-0.01472076	0.98825496	0.99824838	-0.005131	0.000761384
SRGAP1	10.0708873	-0.03695642	2.49804344	-0.01479415	0.98819641	0.99824838	-0.00515673	0.000761384
RP3.370M22.8	79.6426737	-0.03782156	2.51885327	-0.01501539	0.9880199	0.99824838	-0.00523431	0.000761384
SLC9A9	69.310286	-0.03163228	1.9216403	-0.01646108	0.98686655	0.99824838	-0.00574157	0.000761384
WDR13	84.1710013	-0.04328498	2.25004891	-0.01923735	0.98465176	0.99807098	-0.00671734	0.00083857
HIST1H1B	51.4819531	-0.04874769	2.38497796	-0.02043947	0.9836928	0.99807098	-0.00714051	0.00083857
GATA1	497.211334	-0.02078738	1.01420476	-0.02049624	0.98364751	0.99807098	-0.0071605	0.00083857
RBM10	104.14398	-0.04412103	2.13180079	-0.0206966	0.98348768	0.99807098	-0.00723108	0.00083857
NUP58	2.88760741	-0.05681487	2.67326245	-0.02125301	0.98304383	0.99807098	-0.00742712	0.00083857
MACF1	1622.91278	-0.02818862	1.29331206	-0.02179568	0.98261094	0.99807098	-0.00761841	0.00083857
TAF1D	3.10891415	-0.06366316	2.91333764	-0.02185231	0.98256577	0.99807098	-0.00763837	0.00083857
PLA2G12A	81.4833608	-0.04886042	2.19920823	-0.02221728	0.98227464	0.99807098	-0.00776707	0.00083857
PPP4R2	6.98700233	-0.05624417	2.39957133	-0.02343926	0.98129989	0.99807098	-0.00819825	0.00083857
PRKCSH	14.6572507	-0.05715768	2.38567685	-0.02395868	0.98088556	0.99807098	-0.00838166	0.00083857
NT5C2	82.2153526	-0.04115516	1.68598606	-0.02441014	0.98052546	0.99807098	-0.00854113	0.00083857
CKAP5	7.58317009	-0.0430834	1.74201491	-0.02473194	0.98026878	0.99807098	-0.00865483	0.00083857
STYX	3.77485647	-0.05814317	2.33389857	-0.02491246	0.98012479	0.99807098	-0.00871863	0.00083857
IKZF5	13.0925205	-0.05000171	1.98487404	-0.02519138	0.97990232	0.99807098	-0.00881722	0.00083857
TTN	51.7477405	-0.04524838	1.79594056	-0.02519481	0.97989958	0.99807098	-0.00881843	0.00083857
RCHY1	195.303066	-0.06570867	2.48768895	-0.02641354	0.9789275	0.99807098	-0.00924947	0.00083857
DEK	42.15376	-0.06781125	2.31728246	-0.02926327	0.97665462	0.99766907	-0.01025899	0.001013492
EWSR1	237.353632	-0.04399465	1.50073639	-0.02931537	0.97661307	0.99766907	-0.01027747	0.001013492
UBXN4	209.755894	-0.06380399	2.16475176	-0.02947404	0.97648652	0.99766907	-0.01033375	0.001013492
PSD3	111.336415	-0.06866082	2.26964679	-0.03025176	0.97586627	0.99766907	-0.01060969	0.001013492
CHD2	385.967045	-0.06269437	2.0590061	-0.03044885	0.97570908	0.99766907	-0.01067965	0.001013492
SLC23A2	64.664284	-0.06855287	2.24660728	-0.03051395	0.97565717	0.99766907	-0.01070276	0.001013492
CCAR2	16.9401985	-0.08911691	2.88118423	-0.03093065	0.97532484	0.99766907	-0.01085071	0.001013492
PTBP2	104.541016	-0.06026053	1.92607536	-0.0312867	0.9750409	0.99766907	-0.01097717	0.001013492
INTS6	330.233877	-0.0629303	1.96774161	-0.03198098	0.97448722	0.99766907	-0.01122385	0.001013492
B3GNT2	5.01981783	-0.08424704	2.59058765	-0.03252044	0.97405702	0.99766907	-0.01141562	0.001013492
KLC1	3.4093265	-0.06697039	1.94846842	-0.03437079	0.97258148	0.99766907	-0.01207401	0.001013492
DCAF7	166.955942	-0.08348601	2.35228573	-0.03549144	0.97168787	0.99766907	-0.01247322	0.001013492
ADRM1	20.7558259	-0.07401042	2.06261482	-0.03588184	0.97137657	0.99766907	-0.01261237	0.001013492
NOLC1	4.75591995	-0.10520845	2.90575654	-0.0362069	0.97111738	0.99766907	-0.01272827	0.001013492
PCBP2	54.3786425	-0.05268228	1.43931875	-0.03660224	0.97080216	0.99766907	-0.01286927	0.001013492
SFSWAP	19.9074109	-0.09886974	2.69597702	-0.03667306	0.97074569	0.99766907	-0.01289453	0.001013492
KMT2C	937.437944	-0.04477497	1.22050496	-0.03668561	0.97073568	0.99766907	-0.01289901	0.001013492
INPP5B	51.5412729	-0.05714617	1.5558144	-0.03673071	0.97069972	0.99766907	-0.0129151	0.001013492
CERS5	23.5439387	-0.08927698	2.31993757	-0.03848249	0.96930299	0.99766907	-0.01354045	0.001013492
SMYD4	34.3062176	-0.0861891	2.22858487	-0.03867436	0.96915001	0.99766907	-0.01360899	0.001013492
MT.ATP6	334.170822	-0.038934	0.99606155	-0.03908795	0.96882027	0.99766907	-0.01375678	0.001013492
DNAJC3	78.6533844	-0.10915578	2.7154341	-0.04019828	0.96793505	0.99766907	-0.01415378	0.001013492
SPATS2	3.82122417	-0.09432227	2.33209831	-0.04044524	0.96773817	0.99766907	-0.01424213	0.001013492
WBP11	8.5732053	-0.11462152	2.82343174	-0.04059652	0.96761756	0.99766907	-0.01429626	0.001013492
SNPH	107.323658	-0.10984806	2.70068628	-0.04067413	0.96755569	0.99766907	-0.01432403	0.001013492
SSH2	117.331104	-0.0867632	2.10889762	-0.0411415	0.96718309	0.99766907	-0.0144913	0.001013492
KEL	131.860263	-0.10049273	2.39938342	-0.04188273	0.96659218	0.99766907	-0.01475672	0.001013492
TMSB4Y	20.8285617	-0.11269246	2.68949294	-0.04190101	0.96657762	0.99766907	-0.01476327	0.001013492
NQO2	14.4448782	-0.07422595	1.72368467	-0.04306237	0.96565181	0.99766907	-0.01517944	0.001013492
ZFAND1	28.5433889	-0.11743032	2.71685765	-0.04322284	0.96552389	0.99766907	-0.01523697	0.001013492
NOP10	9.17427883	-0.11659926	2.63706828	-0.04421549	0.96473264	0.99766907	-0.01559303	0.001013492
MCL1	5.65545695	-0.12614546	2.76901551	-0.04555607	0.96366408	0.99766907	-0.01607433	0.001013492
RNF141	5.63966845	-0.11953114	2.55435856	-0.04679497	0.96267664	0.99766907	-0.01651957	0.001013492

MTMR10	15.4489465	-0.12062435	2.46835869	-0.04886824	0.9610243	0.99766907	-0.01726563	0.001013492
PCED1B	19.9677025	-0.12073692	2.3260861	-0.05190561	0.95860391	0.99762923	-0.01836081	0.001030833
PPBP	158.263104	-0.08763611	1.68586226	-0.05198296	0.95854227	0.99762923	-0.01838873	0.001030833
MAD2L1BP	159.925576	-0.06651641	1.27250009	-0.05227222	0.95831179	0.99762923	-0.01849317	0.001030833
PRKAR2B	126.129019	-0.12007731	2.18319044	-0.05500084	0.95613779	0.99658517	-0.01947952	0.001485578
FAM110B	3.83449915	-0.13118294	2.37227577	-0.05529835	0.95590078	0.99658517	-0.01958719	0.001485578
TESPA1	6.00966882	-0.16114657	2.90252518	-0.05551944	0.95572464	0.99658517	-0.01966722	0.001485578
PIEZO1	87.2812926	-0.09394756	1.66638568	-0.05637804	0.95504065	0.99658517	-0.01997814	0.001485578
HNRNPA2B1	407.500179	-0.10741685	1.90369358	-0.05642549	0.95500285	0.99658517	-0.01999533	0.001485578
NFKBIZ	304.873699	-0.1065312	1.85674066	-0.05737538	0.95424618	0.99658517	-0.02033957	0.001485578
TIMP1	41.9227563	-0.10009461	1.73169084	-0.05780167	0.95390661	0.99650455	-0.02049414	0.001520714
ISCA1	20.8362771	-0.1216969	2.04174049	-0.05960449	0.95247064	0.99575389	-0.0211484	0.001847987
BOLA1	4.6598484	-0.12265418	2.03468573	-0.06028164	0.95193133	0.99543999	-0.02139438	0.001984915
DDX5	965.830167	-0.07697265	1.27632129	-0.06030821	0.95191017	0.99543999	-0.02140403	0.001984915
ZC3H6	5.80818321	-0.13212948	2.13580443	-0.06186404	0.95067111	0.99512179	-0.0219697	0.002123763
SYTL4	138.812499	-0.13592856	2.18962647	-0.06207843	0.95050038	0.99512179	-0.02204771	0.002123763
ABI2	64.1844711	-0.11379764	1.82248143	-0.06244104	0.95021161	0.99512179	-0.02217967	0.002123763
TANK	6.34625155	-0.12596182	2.00741663	-0.06274822	0.949967	0.99512179	-0.02229148	0.002123763
MICAL2	91.2077609	-0.17513202	2.75493176	-0.06357037	0.94931233	0.99492368	-0.02259088	0.002210232
CNOT7	23.3458234	-0.17194599	2.70206978	-0.06363492	0.94926093	0.99492368	-0.02261439	0.002210232
YWHAE	164.327733	-0.13509199	2.00150085	-0.06749535	0.94618737	0.99312644	-0.02402286	0.002995458
VAPA	112.832652	-0.12945403	1.9137809	-0.06764308	0.94606976	0.99312644	-0.02407684	0.002995458
FAM208A	99.0317295	-0.19522262	2.88414477	-0.06768822	0.94603383	0.99312644	-0.02409333	0.002995458
GK	8.44908143	-0.16794715	2.43323259	-0.06902223	0.94497192	0.9928765	-0.0245811	0.003104768
TAF8	3.34374639	-0.1396255	1.98058942	-0.07049694	0.94379813	0.99191707	-0.02512089	0.003524636
UBR2	389.209531	-0.10776307	1.50631497	-0.07154086	0.94296731	0.99142999	-0.02550336	0.003737948
CCNL1	288.508537	-0.18688063	2.61023942	-0.07159521	0.94292406	0.99142999	-0.02552328	0.003737948
SLC9A1	24.0976417	-0.17378904	2.41393908	-0.07199396	0.94260671	0.99142999	-0.02566947	0.003737948
ASPH	21.5846236	-0.16200972	2.17637757	-0.07444008	0.94066022	0.99061941	-0.02656722	0.004093166
TNKS2	9.41214231	-0.18438168	2.47344074	-0.07454461	0.94057705	0.99061941	-0.02660562	0.004093166
HLA.F	124.308814	-0.1027099	1.34312328	-0.07647094	0.93904444	0.99061941	-0.02731386	0.004093166
ANKHD1	112.138421	-0.13799117	1.80286601	-0.07653989	0.93898958	0.99061941	-0.02733923	0.004093166
MOB2	12.9933056	-0.17531026	2.28742786	-0.07664078	0.93890932	0.99061941	-0.02737635	0.004093166
EHD1	234.20196	-0.15148531	1.94644796	-0.07782654	0.93796603	0.99028666	-0.02781289	0.004239073
RALGAPA1	19.9306972	-0.15364063	1.97010593	-0.07798598	0.93783921	0.99028666	-0.02787161	0.004239073
ARRDC4	5.61332723	-0.23327474	2.90370152	-0.08033702	0.93596921	0.98918168	-0.02873844	0.004723937
RAD9A	4.19268449	-0.1907131	2.35324862	-0.08104248	0.93540817	0.98909081	-0.02899884	0.004763831
PDZD8	28.3358364	-0.19086739	2.35362256	-0.08109516	0.93536628	0.98909081	-0.02901829	0.004763831
SOD1	54.1824744	-0.167835	2.02122861	-0.08303613	0.93382281	0.98851893	-0.02973552	0.005015008
UBXN7	8.19032629	-0.1622925	1.93875176	-0.08370979	0.93328717	0.98851893	-0.0299847	0.005015008
NDUFAF6	12.192662	-0.17425214	2.01353542	-0.08654039	0.93103685	0.98722624	-0.03103313	0.00558331
EEA1	25.0716691	-0.22928306	2.61707704	-0.08761036	0.93018637	0.98677812	-0.03143003	0.005780489
CAMTA2	4.00260813	-0.24238541	2.74171695	-0.08840643	0.92955365	0.98640709	-0.03172554	0.005943817
MED25	6.70858977	-0.19973198	2.25671963	-0.08850544	0.92947496	0.98640709	-0.03176231	0.005943817
LCP2	144.616899	-0.12982656	1.44721775	-0.08970769	0.92851951	0.98586768	-0.03220897	0.00618137
ASH1L	28.5415003	-0.17711642	1.94724734	-0.09095733	0.92752649	0.9855009	-0.03267368	0.006342973
TRAK1	6.93847743	-0.18546327	2.02085414	-0.09177469	0.92687705	0.9855009	-0.03297787	0.006342973
ASAP1	228.873766	-0.11905929	1.27944937	-0.0930551	0.92585979	0.98499748	-0.03345478	0.006564879
PBRM1	100.182034	-0.19259926	2.05287146	-0.09381944	0.92525259	0.98485475	-0.03373969	0.006627818
UBXN11	73.2090074	-0.20280203	2.15666444	-0.09403504	0.92508132	0.98485475	-0.03382009	0.006627818
BRCA2	5.19098549	-0.21256	2.23676404	-0.09503014	0.92429089	0.98485475	-0.03419133	0.006627818
EGLN3	8.63902644	-0.2138218	2.21875366	-0.09637023	0.92322654	0.98485475	-0.03469172	0.006627818
GTF3C5	157.074037	-0.18489466	1.90333904	-0.09714226	0.92261342	0.98485475	-0.03498023	0.006627818
IL27RA	17.7107181	-0.21270455	2.1835002	-0.09741449	0.92239724	0.98485475	-0.035082	0.006627818
FAM65B	157.50766	-0.16561564	1.69215956	-0.09787236	0.92203365	0.98485475	-0.03525323	0.006627818
GDI1	27.4032692	-0.19324393	1.97147749	-0.09801985	0.92191653	0.98485475	-0.0353084	0.006627818
TRIM56	52.7185458	-0.15896248	1.62152089	-0.09803295	0.92190613	0.98485475	-0.0353133	0.006627818
ZNF778	358.855761	-0.15803028	1.60752478	-0.09830659	0.92168884	0.98485475	-0.03541567	0.006627818
TMEM63A	1407.68653	-0.14154068	1.43040749	-0.0989513	0.92117694	0.98485475	-0.03565694	0.006627818
YY1	83.3669804	-0.16797967	1.69488986	-0.09910949	0.92105134	0.98485475	-0.03571616	0.006627818
AKIRIN2	29.978686	-0.21210302	2.12172135	-0.09996743	0.92037019	0.98485475	-0.03603746	0.006627818
ZNF326	6.67778617	-0.23036634	2.29005693	-0.10059415	0.91987264	0.98485475	-0.0362723	0.006627818

MYL12A	306.486201	-0.1573827	1.55697233	-0.10108253	0.91948494	0.98485475	-0.03645538	0.006627818
KLF13	5.52505873	-0.21120714	2.07133006	-0.10196691	0.91878294	0.98485475	-0.03678708	0.006627818
SMG1	87.8446031	-0.17545481	1.69482447	-0.10352388	0.9175472	0.98485475	-0.03737159	0.006627818
ZNF521	72.4633035	-0.24581343	2.3438685	-0.10487509	0.91647492	0.98482621	-0.03787941	0.0066404
ZNF217	11.6810384	-0.27994774	2.66617599	-0.10499972	0.91637603	0.98482621	-0.03792628	0.0066404
HIST1H2BD	143.106226	-0.22346961	2.12529029	-0.1051478	0.91625853	0.98482621	-0.03798197	0.0066404
PTPRJ	281.126719	-0.16006813	1.51920092	-0.10536337	0.91608748	0.98482621	-0.03806305	0.0066404
SWI5	11.6410291	-0.22649623	2.13868881	-0.10590425	0.91565832	0.98482621	-0.03826655	0.0066404
IP6K1	114.280827	-0.24433109	2.29848898	-0.10630074	0.91534374	0.98482621	-0.03841578	0.0066404
TAOK2	41.2803548	-0.21504198	1.99980675	-0.10753138	0.91436743	0.98482621	-0.03887925	0.0066404
KIFC3	1091.18234	-0.13857414	1.28856654	-0.10754131	0.91435955	0.98482621	-0.03888299	0.0066404
SPNS1	79.2448412	-0.17239618	1.59988931	-0.10775506	0.91418999	0.98482621	-0.03896354	0.0066404
HIST1H3H	774.525282	-0.13168226	1.21829569	-0.10808728	0.91392646	0.98482621	-0.03908875	0.0066404
GIGYF1	38.2276268	-0.21233938	1.9613137	-0.10826385	0.91378639	0.98482621	-0.03915531	0.0066404
TET2	376.898527	-0.14733681	1.35499239	-0.10873626	0.91341168	0.98482621	-0.03933344	0.0066404
ATP5S	59.5310118	-0.20301007	1.83788245	-0.11045868	0.91204562	0.98482621	-0.03998344	0.0066404
STK11IP	4.17355147	-0.24881808	2.21956019	-0.11210243	0.91074219	0.98476513	-0.04060454	0.00666734
GADD45A	133.004046	-0.28733134	2.53650317	-0.11327853	0.90980974	0.98426806	-0.04104942	0.006886608
EIF3M	3.22923084	-0.31206645	2.7015084	-0.11551563	0.90803643	0.98337157	-0.04189673	0.007282351
HIST1H2AB	34.6387857	-0.21201933	1.83039995	-0.11583224	0.9077855	0.98335557	-0.04201676	0.007289419
ZNF438	29.6715015	-0.25975545	2.22730693	-0.1166231	0.90715873	0.98335557	-0.04231672	0.007289419
PRKD2	43.3973429	-0.2590157	2.20253638	-0.11759883	0.90638553	0.98311783	-0.04268704	0.007394427
GOSR1	4.79715674	-0.23302555	1.97743926	-0.11784208	0.90619279	0.98311783	-0.0427794	0.007394427
VPS50	10.0530159	-0.25545088	2.15655996	-0.11845295	0.90570878	0.98299593	-0.04301142	0.007448282
RBM38	441.338707	-0.13436828	1.11148638	-0.12089062	0.90377767	0.98213474	-0.04393839	0.007828929
TMEM159	2.78948356	-0.35386335	2.91644772	-0.12133369	0.90342674	0.98213474	-0.04410706	0.007828929
ABHD4	239.673609	-0.24710301	2.02473116	-0.12204238	0.90286545	0.98213474	-0.04437696	0.007828929
HIST1H4K	15.0682703	-0.27193474	2.20989013	-0.12305351	0.90206472	0.98201242	-0.0447623	0.00788302
NGLY1	3.67566573	-0.28544589	2.31283744	-0.12341805	0.90177606	0.9819549	-0.0449013	0.00790846
BICD2	45.8594621	-0.26918656	2.14884349	-0.12527044	0.90030946	0.9808709	-0.04560819	0.008388151
VAPB	8.95380126	-0.24082136	1.87948842	-0.12813133	0.89804504	0.9794289	-0.04670188	0.009027085
LRRFIP2	18.5438651	-0.28996622	2.24323457	-0.12926255	0.8971499	0.97922207	-0.04713498	0.009118806
MTHFR	46.2622923	-0.25027801	1.93029559	-0.12965787	0.89683712	0.97922207	-0.04728643	0.009118806
TRIP10	73.3699574	-0.20402812	1.56948999	-0.12999644	0.89656924	0.97922207	-0.04741616	0.009118806
MICAL3	73.5013754	-0.22854731	1.75539375	-0.13019718	0.89641043	0.97922207	-0.0474931	0.009118806
EPHA6	3.43645706	-0.30362163	2.31079619	-0.13139265	0.89546471	0.97922207	-0.04795153	0.009118806
FBXO22	77.2424285	-0.27800559	2.08177059	-0.13354286	0.89376407	0.97860468	-0.04877711	0.009392711
MEPCE	6.63795286	-0.24199213	1.80845626	-0.13381144	0.89355168	0.97860468	-0.04888032	0.009392711
PRKAR1A	102.292778	-0.26372289	1.95845894	-0.13465837	0.89288199	0.97815327	-0.04920594	0.009593087
MLH3	36.995957	-0.29487261	2.16219005	-0.13637683	0.89152339	0.97779196	-0.04986726	0.00975354
MBD6	57.1754551	-0.21772703	1.58241405	-0.13759169	0.89056312	0.97767067	-0.05033529	0.009807415
CCL5	85.6977881	-0.2273169	1.63320809	-0.13918429	0.88930453	0.97726004	-0.0509495	0.009989859
KCNA3	160.493098	-0.25862031	1.84502564	-0.14017166	0.88852436	0.97697793	-0.05133066	0.010115245
XAB2	6.31498519	-0.30290383	2.14843133	-0.14098837	0.88787913	0.97697793	-0.05164615	0.010115245
FXYD5	130.675331	-0.19595979	1.38467026	-0.14152091	0.88745844	0.97697793	-0.05185197	0.010115245
MPRIP	25.0155326	-0.31549257	2.19551093	-0.14369893	0.88573822	0.97697793	-0.05269462	0.010115245
SF3A2	3.46649898	-0.28636726	1.98584124	-0.14420451	0.88533898	0.97697793	-0.05289041	0.010115245
PPP1R10	32.5943787	-0.26840419	1.85900492	-0.14438057	0.88519996	0.97697793	-0.05295862	0.010115245
CRTC2	27.9894816	-0.36752513	2.54465221	-0.1444304	0.88516061	0.97697793	-0.05297792	0.010115245
SIAE	121.341652	-0.31314584	2.16713697	-0.14449748	0.88510764	0.97697793	-0.05300391	0.010115245
ATXN2L	62.8163084	-0.24623031	1.6970408	-0.14509393	0.88463671	0.97697793	-0.05323504	0.010115245
NAA38	13.951754	-0.38940783	2.65156527	-0.14685961	0.88324283	0.97697793	-0.05391988	0.010115245
VAMP2	256.198666	-0.35169673	2.34489993	-0.14998368	0.88077749	0.97567371	-0.05513379	0.010695399
ACTR3B	27.2185617	-0.35608058	2.36911038	-0.15030139	0.88052684	0.9756556	-0.0552574	0.010703458
HERPUD2	9.88127663	-0.42086838	2.79980036	-0.15032085	0.88051148	0.9756556	-0.05526498	0.010703458
BRAF	30.8579311	-0.34545023	2.26271809	-0.15267047	0.87865815	0.9750799	-0.05618006	0.010959797
LAMTOR1	21.079728	-0.39095302	2.53536386	-0.15419997	0.87745207	0.9750799	-0.0567766	0.010959797
SRRM2	819.241927	-0.18689169	1.20912462	-0.15456776	0.87716208	0.9750799	-0.05692015	0.010959797
JARID2	90.9965309	-0.22124547	1.42735402	-0.15500392	0.87681823	0.9750799	-0.05709043	0.010959797
LPP	22.8742234	-0.37349752	2.40752352	-0.15513764	0.87671281	0.9750799	-0.05714265	0.010959797
ZNF292	64.0508698	-0.39451159	2.53658553	-0.1555286	0.87640461	0.9750799	-0.05729535	0.010959797
ACOX1	24.7674728	-0.38664071	2.47590736	-0.15616122	0.87590594	0.9750799	-0.05754253	0.010959797

ZFC3H1	129.811913	-0.26832351	1.71357278	-0.15658717	0.87557021	0.9750799	-0.05770902	0.010959797
SESN1	8.76860136	-0.39552334	2.5210204	-0.15689018	0.8753314	0.9750799	-0.05782749	0.010959797
GNAI2	14.9891397	-0.35070678	2.23244946	-0.15709506	0.87516993	0.9750799	-0.05790761	0.010959797
MTRNR2L8	3.79256647	-0.18917617	1.1986298	-0.15782702	0.87459311	0.9750799	-0.05819395	0.010959797
SLC37A1	106.058524	-0.37804698	2.38768841	-0.15833179	0.87419536	0.9750799	-0.0583915	0.010959797
LYPLAL1	6.34719335	-0.40371356	2.54640945	-0.15854228	0.87402951	0.9750799	-0.05847391	0.010959797
SMCHD1	6.7246772	-0.38806275	2.40167266	-0.1615802	0.87163645	0.9738238	-0.05966462	0.011519615
RSRP1	215.931099	-0.32604171	2.0078213	-0.16238582	0.87100203	0.97374455	-0.05998083	0.01155496
BFAR	8.15708359	-0.18092003	1.11387029	-0.16242468	0.87097144	0.97374455	-0.05999609	0.01155496
POLR2A	195.941508	-0.2871361	1.75066011	-0.1640159	0.86971862	0.97326212	-0.06062123	0.011770179
LOXHD1	163.247839	-0.30733076	1.86278221	-0.16498481	0.86895594	0.97267004	-0.06100224	0.012034462
PLEK	2.81939865	-0.43832966	2.65550471	-0.16506454	0.86889318	0.97267004	-0.06103361	0.012034462
AQP10	35.8546822	-0.33022974	1.99932148	-0.16517091	0.86880946	0.97267004	-0.06107546	0.012034462
CACYBP	5.03192313	-0.48154565	2.90665173	-0.16567023	0.86841648	0.97267004	-0.06127195	0.012034462
LAT	1399.65083	-0.18194947	1.09760349	-0.16576976	0.86833814	0.97267004	-0.06131112	0.012034462
IFT81	120.453462	-0.40723154	2.44627244	-0.16647023	0.86778691	0.97267004	-0.06158691	0.012034462
MAP4K5	314.544573	-0.20772478	1.23077833	-0.16877513	0.86597352	0.97213278	-0.06249539	0.012274413
NUTF2	152.947023	-0.29683866	1.75312006	-0.16932021	0.86554478	0.97213278	-0.06271046	0.012274413
CEP350	215.049059	-0.31722293	1.87048227	-0.16959419	0.86532929	0.97213278	-0.0628186	0.012274413
SYNGAP1	2.75084606	-0.26345634	1.54427685	-0.17060175	0.86453692	0.97203652	-0.06321646	0.012317416
TOR1AIP2	4.33827587	-0.28123883	1.645563	-0.17090736	0.86429661	0.97203652	-0.06333719	0.012317416
SAFB2	10.3297079	-0.31252434	1.81814912	-0.17189148	0.86352285	0.97203652	-0.06372617	0.012317416
PYCR2	53.5843753	-0.32783952	1.90449466	-0.1721399	0.86332755	0.97203652	-0.0638244	0.012317416
RAC1	21.2055641	-0.36367638	2.06944766	-0.17573596	0.86050138	0.97125306	-0.06524843	0.012667598
TMPRSS12	3.23580049	-0.46945842	2.67122385	-0.17574657	0.86049305	0.97125306	-0.06525263	0.012667598
CFL1	52.0434793	-0.20743459	1.16651294	-0.17782451	0.8588608	0.97125306	-0.06607722	0.012667598
ATP6V0A2	22.263792	-0.31149317	1.74861501	-0.17813708	0.85861532	0.97125306	-0.06620136	0.012667598
PLXNB2	128.557872	-0.32755985	1.82892573	-0.17909959	0.8578595	0.97125306	-0.06658383	0.012667598
MIDN	15.46236	-0.324342	1.78169864	-0.18204089	0.85555064	0.97086669	-0.06775428	0.012840401
PTP4A2	79.6494001	-0.29528893	1.60491328	-0.18399058	0.85402084	0.97003349	-0.06853153	0.01321327
DRAP1	4.42299887	-0.46880249	2.54480658	-0.1842193	0.85384142	0.97003349	-0.06862278	0.01321327
ZMYM4	7.88119068	-0.36935292	2.00398185	-0.18430951	0.85377065	0.97003349	-0.06865878	0.01321327
FRMD3	34.2457502	-0.42388392	2.29851264	-0.18441661	0.85368664	0.97003349	-0.06870152	0.01321327
STT3B	64.4674751	-0.36276294	1.95797101	-0.18527493	0.8530134	0.96994752	-0.06904414	0.013251764
PECAM1	462.298809	-0.30166436	1.62555256	-0.1855765	0.85277688	0.96994752	-0.06916458	0.013251764
TDP2	24.8285818	-0.43308352	2.29854097	-0.18841671	0.85055	0.96895794	-0.07030015	0.013695074
SEMA4D	150.4261	-0.39177084	2.0588983	-0.19028178	0.84908833	0.96839326	-0.07104713	0.013948241
RNPEPL1	56.8162347	-0.51690093	2.71455378	-0.19041838	0.8489813	0.96839326	-0.07110188	0.013948241
HELZ	140.076717	-0.308257	1.60265832	-0.19234106	0.84747506	0.96761342	-0.07187307	0.014298117
HFE	54.1180264	-0.45085487	2.32134532	-0.19422137	0.84600255	0.96699668	-0.07262833	0.014575016
RUSC2	189.26437	-0.39845078	2.04824306	-0.19453296	0.84575859	0.96699668	-0.07275358	0.014575016
RPL22L1	41.1438125	-0.56502265	2.8909362	-0.19544625	0.84504361	0.96695839	-0.07312088	0.014592213
UTRN	86.2578508	-0.30887084	1.55892963	-0.19813007	0.8429433	0.96561648	-0.07420164	0.015195333
HSPA2	59.2817059	-0.4301401	2.16807565	-0.19839718	0.84273432	0.96561648	-0.07430932	0.015195333
NUDCD2	73.3882216	-0.41574963	2.09515219	-0.1984341	0.84270544	0.96561648	-0.0743242	0.015195333
YWHAZ	286.724172	-0.28704996	1.43976807	-0.19937236	0.84197148	0.96561648	-0.07470262	0.015195333
ITGA5	144.006624	-0.54820297	2.74957131	-0.19937761	0.84196737	0.96561648	-0.07470474	0.015195333
PDXDC1	14.67021	-0.57984683	2.89508587	-0.20028657	0.84125646	0.96561648	-0.07507159	0.015195333
CTSD	8.92575019	-0.4402034	2.19212161	-0.20081158	0.84084591	0.96561648	-0.07528358	0.015195333
SMG7	54.6042731	-0.48369535	2.40482344	-0.2011355	0.84059263	0.96561648	-0.07541442	0.015195333
SRRT	8.31264607	-0.4415329	2.17381088	-0.20311468	0.8390454	0.96491364	-0.07621454	0.015511554
H3F3A	142.610271	-0.29641723	1.45898603	-0.2031666	0.83900482	0.96491364	-0.07623554	0.015511554
SAT1	263.462303	-0.23435044	1.14317966	-0.20499878	0.83757307	0.9642395	-0.07697729	0.01581508
CHD7	51.0843145	-0.48304097	2.35028696	-0.20552425	0.83716255	0.9642395	-0.07719021	0.01581508
UPF1	66.0791184	-0.37951725	1.82828581	-0.20758092	0.8355562	0.96378283	-0.07802433	0.016020814
ANKRD11	121.465119	-0.33179193	1.59671711	-0.20779632	0.83538801	0.96378283	-0.07811176	0.016020814
HNRNPC	158.164519	-0.4760911	2.28652216	-0.20821626	0.83506011	0.96378283	-0.07828226	0.016020814
CLK1	634.214859	-0.29935771	1.43710329	-0.20830633	0.83498979	0.96378283	-0.07831883	0.016020814
FLOT2	31.0456335	-0.53346792	2.56052448	-0.20834322	0.83496098	0.96378283	-0.07833382	0.016020814
BZW1	102.898067	-0.48167443	2.29481707	-0.20989665	0.83374833	0.96378283	-0.07896502	0.016020814
SPRY1	13.0894959	-0.48066829	2.27020702	-0.21172884	0.83231859	0.96271517	-0.07971041	0.016502186
SPPL3	95.1407052	-0.47414592	2.23501382	-0.21214451	0.83199429	0.96260746	-0.07987965	0.016550779

SETD2	73.5567993	-0.50889657	2.38884286	-0.21303058	0.83130311	0.96234411	-0.08024059	0.016669609
ZNF451	322.255002	-0.35172308	1.64735937	-0.2135072	0.83093138	0.96234411	-0.08043484	0.016669609
TMSB4X	335.031351	-0.32001721	1.49542382	-0.21399767	0.83054888	0.96234411	-0.0806348	0.016669609
UGT2B7	7.80683068	-0.59149975	2.76384619	-0.21401326	0.83053671	0.96234411	-0.08064117	0.016669609
KLF9	59.4039019	-0.36401169	1.70028793	-0.21408826	0.83047823	0.96234411	-0.08067175	0.016669609
ACYP2	5.307477	-0.41983301	1.95368349	-0.21489306	0.8298507	0.96234411	-0.08100004	0.016669609
CCDC186	92.5426965	-0.53919552	2.49846348	-0.21581085	0.82913519	0.96234411	-0.08137465	0.016669609
ARHGAP35	18.4201157	-0.54576676	2.52107911	-0.21648141	0.82861252	0.96234411	-0.08164851	0.016669609
HIST1H2AC	490.655864	-0.25361747	1.16508426	-0.21768165	0.82767716	0.96188884	-0.08213903	0.016875115
HIST1H2BG	2303.5206	-0.28866199	1.3154259	-0.21944375	0.82630439	0.96125495	-0.08285994	0.017161411
KIF20B	5.86879697	-0.51965385	2.36745229	-0.21949918	0.82626121	0.96125495	-0.08288263	0.017161411
TTC17	13.9719787	-0.62241601	2.83038333	-0.2199052	0.82594499	0.96125495	-0.08304888	0.017161411
EFCAB13	36.8027953	-0.3708487	1.6765787	-0.22119373	0.82494159	0.96112389	-0.0835768	0.017220629
ZBTB20	18.1543939	-0.50628385	2.27772666	-0.22227595	0.82409907	0.96094891	-0.08402057	0.017299703
FZR1	73.5343275	-0.50395219	2.26568351	-0.22242833	0.82398046	0.96094891	-0.08408309	0.017299703
EYS	4.80213964	-0.45681249	2.04868547	-0.22297834	0.82355236	0.96084955	-0.08430878	0.017344609
SNAP23	42.4384592	-0.37744203	1.69044144	-0.22328016	0.82331747	0.96084955	-0.08443267	0.017344609
CLN5	106.453903	-0.44487875	1.99139933	-0.22340007	0.82322415	0.96084955	-0.0844819	0.017344609
PLXDC2	44.4229601	-0.5568821	2.48887358	-0.22374865	0.82295289	0.96084955	-0.08462503	0.017344609
DOCK4	5.78285973	-0.56806503	2.53692965	-0.22391832	0.82282086	0.96084955	-0.08469471	0.017344609
COG1	4.96026374	-0.49599287	2.20720981	-0.22471487	0.82220109	0.96084955	-0.08502195	0.017344609
RAB9A	7.51050076	-0.62806092	2.78202138	-0.22575704	0.82139039	0.96084955	-0.08545038	0.017344609
ZFAND2A	36.8511337	-0.62999212	2.78608522	-0.22612091	0.82110738	0.96084955	-0.08560004	0.017344609
ZMYM2	20.3191897	-0.49157536	2.17285153	-0.22623514	0.82101854	0.96084955	-0.08564703	0.017344609
HIST1H3D	92.2539708	-0.4219366	1.86492797	-0.2262482	0.82100838	0.96084955	-0.08565241	0.017344609
RBM17	216.526538	-0.34221021	1.50123456	-0.22795252	0.81968315	0.96084955	-0.08635399	0.017344609
NABP1	88.04547	-0.36961234	1.61490854	-0.22887509	0.818966	0.96084955	-0.08673413	0.017344609
BTAF1	42.3584866	-0.47361435	2.06274594	-0.22960382	0.81839964	0.9607601	-0.08703457	0.017385039
NFYC	5.66450387	-0.50572897	2.20160895	-0.22970881	0.81831805	0.9607601	-0.08707787	0.017385039
KMT2A	896.21575	-0.21730912	0.94258995	-0.2305447	0.81766853	0.96071438	-0.08742272	0.017405708
INPPL1	115.514339	-0.43741784	1.89114951	-0.23129733	0.81708382	0.96056946	-0.08773339	0.017471226
CTNS	81.65709	-0.43980859	1.89562265	-0.23201273	0.81652813	0.96028244	-0.08802885	0.017601014
SLC8B1	5.57522676	-0.60492413	2.60056781	-0.23261233	0.81606245	0.96018199	-0.0882766	0.017646445
OVOS2	5.50738632	-0.59416618	2.55147464	-0.23287168	0.81586105	0.96018199	-0.0883838	0.017646445
RANBP9	142.238056	-0.56910068	2.44331527	-0.23292151	0.81582236	0.96018199	-0.0884044	0.017646445
PCGF5	50.9078646	-0.45334487	1.94359009	-0.23325128	0.81556629	0.96018199	-0.08854073	0.017646445
LAMA5	140.390352	-0.4422939	1.87235213	-0.23622367	0.81325911	0.95905153	-0.08977106	0.018158059
WTAP	200.952911	-0.45389503	1.91960807	-0.23645193	0.813082	0.95905153	-0.08986565	0.018158059
SNAP29	15.3842427	-0.56837104	2.40126071	-0.23669693	0.81289192	0.95905153	-0.0899672	0.018158059
MAP3K5	159.447927	-0.36992992	1.55876938	-0.23732178	0.81240717	0.95905153	-0.09022625	0.018158059
PHF12	83.5730856	-0.50650813	2.12636523	-0.23820373	0.81172308	0.95905153	-0.0905921	0.018158059
PDS5A	4.39880441	-0.64677024	2.71438365	-0.23827518	0.81166767	0.95905153	-0.09062175	0.018158059
TMEM50B	10.179446	-0.62922835	2.63648365	-0.23866196	0.81136772	0.95905153	-0.09078228	0.018158059
ARHGAP21	247.562694	-0.40410507	1.68439342	-0.23991133	0.810399	0.95905153	-0.09130111	0.018158059
BRD2	126.592639	-0.29091287	1.21253166	-0.23992188	0.81039082	0.95905153	-0.09130549	0.018158059
ATM	47.9940238	-0.36701225	1.52229707	-0.24109109	0.80948452	0.95905153	-0.09179145	0.018158059
JAK1	315.850916	-0.41731361	1.72858788	-0.2414188	0.80923054	0.95905153	-0.09192773	0.018158059
RUBCN	77.6854698	-0.53978499	2.21692072	-0.24348412	0.80763039	0.95811194	-0.09278735	0.018583746
TBC1D5	3.16417225	-0.42111468	1.72250323	-0.24447831	0.8068604	0.9575803	-0.0932016	0.018824795
KIAA1586	109.592428	-0.52342714	2.13487706	-0.24517905	0.80631779	0.9575803	-0.09349376	0.018824795
NPIPB3	21.1798587	-0.29304946	1.1949103	-0.24524808	0.80626435	0.9575803	-0.09352254	0.018824795
HLA.C	271.956939	-0.20473219	0.8335552	-0.24561323	0.80598165	0.9575803	-0.09367485	0.018824795
RAB5B	5.0114973	-0.5671101	2.30599488	-0.24592861	0.8057375	0.9575803	-0.09380642	0.018824795
ZFP36L1	57.6920654	-0.45742035	1.84950202	-0.24732081	0.80465995	0.95681946	-0.09438761	0.01917
CDIP1	13.1637549	-0.48371619	1.9544434	-0.24749563	0.80452468	0.95681946	-0.09446063	0.01917
YPEL3	295.339599	-0.27835621	1.12222556	-0.24803945	0.80410388	0.95681946	-0.09468784	0.01917
SON	379.994529	-0.38337164	1.54466682	-0.24819051	0.80398701	0.95681946	-0.09475097	0.01917
CTDSP1	70.5243122	-0.51236466	2.05456998	-0.24937805	0.80306836	0.95677892	-0.09524748	0.019188401
NEURL1B	21.5108429	-0.72232978	2.89325647	-0.24965978	0.80285046	0.95677892	-0.09536534	0.019188401
NMRK1	61.054236	-0.65633674	2.62507493	-0.25002591	0.80256732	0.95677892	-0.09551853	0.019188401
HNRNPH1	1552.99391	-0.39692796	1.58477327	-0.25046356	0.80222888	0.95677892	-0.09570171	0.019188401
ZSWIM8	7.38682585	-0.65906041	2.62694928	-0.25088433	0.80190354	0.95677892	-0.09587787	0.019188401

SIK2	3.10662109	-0.73341092	2.91636004	-0.25148161	0.80144178	0.95677892	-0.09612802	0.019188401
GNAS	771.472164	-0.21406169	0.84977967	-0.25190257	0.80111637	0.95677892	-0.09630439	0.019188401
CDK16	14.954769	-0.62631462	2.48325555	-0.25221513	0.80087478	0.95677892	-0.09643538	0.019188401
TRA2B	132.287484	-0.34374989	1.34549177	-0.25548272	0.79835029	0.955542	-0.09780651	0.01975022
OFD1	79.4660423	-0.43489296	1.70061018	-0.2557276	0.79816118	0.955542	-0.0979094	0.01975022
NAA60	49.6121158	-0.4523054	1.76834538	-0.25577888	0.79812158	0.955542	-0.09793094	0.01975022
PRR14	31.3391306	-0.42912493	1.6553091	-0.25924157	0.79544886	0.95430668	-0.09938774	0.020312037
TOPORS	105.528286	-0.62753733	2.41747352	-0.25958395	0.79518472	0.95430668	-0.09953197	0.020312037
ACTN1	199.651935	-0.39023881	1.49915673	-0.26030554	0.7946281	0.95410363	-0.09983608	0.020404451
HBG2	3.86336659	-0.75799546	2.89957218	-0.26141631	0.79377148	0.95362506	-0.10030451	0.020622347
BIN2	2156.86555	-0.31151523	1.1905527	-0.26165598	0.79358668	0.95362506	-0.10040563	0.020622347
KCTD10	75.8166782	-0.49670732	1.89694842	-0.26184545	0.7934406	0.95362506	-0.10048558	0.020622347
RALY	37.8688642	-0.48705212	1.84837919	-0.26350227	0.79216347	0.95290343	-0.10118519	0.020951111
ZNF429	16.0554858	-0.54463777	2.06415386	-0.26385522	0.79189148	0.95290343	-0.10133433	0.020951111
CNOT8	15.4573281	-0.7041423	2.65705557	-0.26500849	0.79100292	0.95277297	-0.10182191	0.021010573
ITGA2	2.82229931	-0.56220314	2.10657203	-0.26688057	0.78956112	0.95173872	-0.10261425	0.021482262
ITGB5	327.756774	-0.47396145	1.77556562	-0.26693548	0.78951884	0.95173872	-0.1026375	0.021482262
UBE2Q1	12.8789992	-0.71789218	2.67960165	-0.26791004	0.78876857	0.9514578	-0.1030504	0.021610471
UBE2O	17.2648484	-0.65216764	2.41615126	-0.26992004	0.78722177	0.95101119	-0.1039029	0.021814374
HIST1H2BE	8.42810521	-0.7128495	2.6361978	-0.2704082	0.78684623	0.95101119	-0.10411013	0.021814374
WIPI2	12.5817208	-0.68104594	2.51694949	-0.27058387	0.7867111	0.95101119	-0.10418472	0.021814374
STAG2	139.802511	-0.50878125	1.87317016	-0.27161507	0.78591801	0.95077356	-0.10462276	0.021922905
PHF20L1	104.458961	-0.4684445	1.71720764	-0.27279432	0.78501132	0.95050513	-0.10512408	0.022045534
BAG6	3.11259854	-0.56993247	2.0833139	-0.27357014	0.78441498	0.95050513	-0.10545412	0.022045534
PPFIA1	32.7643395	-0.63519003	2.30865078	-0.27513474	0.78321272	0.94970815	-0.10612027	0.022409836
MT.CYB	499.459484	-0.43877058	1.59412124	-0.27524292	0.78312962	0.94970815	-0.10616635	0.022409836
HSPA4	39.786829	-0.66133185	2.39319202	-0.27633882	0.78228785	0.94970815	-0.10663342	0.022409836
ADIPOR2	29.6364274	-0.63378246	2.28748643	-0.27706501	0.78173019	0.94956957	-0.10694312	0.02247321
VAV3	27.6574827	-0.61484885	2.21652449	-0.27739321	0.78147819	0.94954047	-0.10708314	0.02248652
SFXN3	42.7055738	-0.7691141	2.76633172	-0.27802671	0.78099186	0.94950368	-0.10735349	0.022503349
PRKAA1	39.0873053	-0.80818344	2.89126577	-0.27952582	0.77984132	0.94893608	-0.10799376	0.022763039
CHD3	17.576998	-0.65600187	2.34505358	-0.27973854	0.7796781	0.94893608	-0.10808466	0.022763039
MADD	822.186725	-0.3684039	1.3138523	-0.28039978	0.7791708	0.94893608	-0.10836733	0.022763039
PPP1R15A	1221.71784	-0.31031142	1.09214786	-0.2841295	0.77631114	0.94753175	-0.10996418	0.023406231
ZNF664	22.4227011	-0.57771814	2.03072996	-0.28448792	0.77603649	0.94753175	-0.11011786	0.023406231
HBP1	3.26887422	-0.59769568	2.07908311	-0.28748042	0.77374449	0.94677404	-0.11140243	0.023753657
CUL3	37.8889437	-0.5113301	1.77639254	-0.28784747	0.7734635	0.94670841	-0.11156018	0.023783766
GSAP	81.6268697	-0.63732512	2.2112484	-0.2882196	0.77317865	0.94663802	-0.11172015	0.023816059
LARP4B	52.8625079	-0.63605168	2.20139405	-0.28893131	0.77263394	0.94624933	-0.11202622	0.023994414
SCARF1	15.7456538	-0.50254406	1.73589627	-0.28950121	0.77219786	0.94599349	-0.11227141	0.024111851
COPB1	7.42318901	-0.74903907	2.57613413	-0.2907609	0.77123419	0.9453725	-0.11281372	0.024397033
KIAA0922	806.986629	-0.41507187	1.42422522	-0.29143696	0.77071715	0.94529196	-0.11310498	0.024434034
VPS26B	4.24350164	-0.74913355	2.56846259	-0.29166613	0.77054191	0.94529196	-0.11320374	0.024434034
SLC44A2	341.395379	-0.38627824	1.32370822	-0.29181525	0.77042789	0.94529196	-0.11326801	0.024434034
CDC14B	111.25848	-0.63236242	2.16158076	-0.29254628	0.76986898	0.94529196	-0.11358318	0.024434034
TMCC2	12.2335868	-0.63475546	2.16790151	-0.29279719	0.76967718	0.94529196	-0.11369139	0.024434034
SSFA2	300.454564	-0.51711056	1.76500395	-0.29297983	0.76953757	0.94529196	-0.11377017	0.024434034
DHRS11	15.3909516	-0.66347787	2.26343185	-0.29312915	0.76942343	0.94529196	-0.11383459	0.024434034
PTPN12	187.905665	-0.57118125	1.94778069	-0.29324721	0.7693332	0.94529196	-0.11388552	0.024434034
PER1	152.908207	-0.51263169	1.73273007	-0.29585202	0.7673431	0.94477133	-0.11501041	0.024673293
CMTM6	348.556494	-0.58308483	1.97026777	-0.29594192	0.76727443	0.94477133	-0.11504927	0.024673293
HIST1H2BB	99.4573793	-0.52147176	1.76144497	-0.29604772	0.76719364	0.94477133	-0.115095	0.024673293
MST1R	2.95998792	-0.34831227	1.17247225	-0.29707506	0.7664092	0.94473887	-0.11553929	0.024688214
GRTP1	24.983598	-0.72375917	2.42674021	-0.29824337	0.76551742	0.94419862	-0.11604492	0.024936637
PDIA5	6.17927907	-0.77001289	2.57992094	-0.29846376	0.76534924	0.94419862	-0.11614035	0.024936637
ESYT1	3.24719722	-0.69030565	2.30773603	-0.29912678	0.76484331	0.94419862	-0.11642753	0.024936637
KDM3A	243.597586	-0.50918331	1.7021687	-0.29913798	0.76483477	0.94419862	-0.11643238	0.024936637
RNF19A	291.650777	-0.71678451	2.39169767	-0.29969695	0.76440832	0.94419862	-0.11667459	0.024936637
MED13L	123.529409	-0.52553995	1.75282988	-0.29982371	0.76431163	0.94419862	-0.11672953	0.024936637
TRMT1	21.5692279	-0.50885673	1.69251257	-0.30065167	0.76368013	0.94419862	-0.11708851	0.024936637
IRAK2	48.5165145	-0.60264867	2.00161373	-0.3010814	0.76335242	0.94419862	-0.11727491	0.024936637
CD82	37.7437649	-0.59412303	1.96996761	-0.30159025	0.76296444	0.94419862	-0.1174957	0.024936637

MAU2	98.5802314	-0.64251567	2.12603287	-0.30221342	0.76248937	0.94419862	-0.11776621	0.024936637
CLK4	88.4288025	-0.70565176	2.33395361	-0.30234181	0.76239151	0.94419862	-0.11782195	0.024936637
ZNF24	282.416975	-0.61250479	2.01614484	-0.30379999	0.76128027	0.94400567	-0.11845543	0.025025396
ARHGEF26	11.7893172	-0.6982433	2.29535463	-0.30419844	0.76097671	0.94400307	-0.11862863	0.025026594
KHDRBS1	4.7218147	-0.73959094	2.40017495	-0.30814043	0.75797548	0.94074865	-0.12034484	0.026526396
CLMN	9.35208143	-0.599688	1.94396448	-0.30848712	0.7577117	0.94074865	-0.12049601	0.026526396
ABLIM1	134.330599	-0.56546411	1.83260033	-0.30855833	0.75765752	0.94074865	-0.12052706	0.026526396
TSPAN33	153.940614	-0.73500898	2.3677096	-0.31043038	0.7562337	0.94026788	-0.12134397	0.026748401
ZNF850	37.6669366	-0.67933347	2.18808813	-0.31046897	0.75620435	0.94026788	-0.12136083	0.026748401
SSBP2	142.714187	-0.58022097	1.86040248	-0.31187927	0.75513228	0.94026788	-0.12197697	0.026748401
PAIP2	81.3299951	-0.62540568	2.00502336	-0.3119194	0.75510178	0.94026788	-0.12199451	0.026748401
LRRC71	14.7706319	-0.78488434	2.48191826	-0.31624101	0.75181958	0.93786002	-0.12388637	0.027861979
PDCD4	60.850588	-0.48942088	1.54727156	-0.31631221	0.75176555	0.93786002	-0.12391758	0.027861979
MMD	55.7256834	-0.48771071	1.5413932	-0.31640902	0.75169207	0.93786002	-0.12396003	0.027861979
TRPC4AP	191.779341	-0.75453595	2.37745258	-0.31737161	0.75096165	0.93786002	-0.12438224	0.027861979
NOTCH1	186.149208	-0.53433633	1.68299086	-0.31749212	0.75087022	0.93786002	-0.12443512	0.027861979
CREBBP	26.2269582	-0.50177615	1.56572419	-0.32047544	0.74860795	0.93637834	-0.12574557	0.028548641
MED23	4.4383212	-0.93309186	2.91121872	-0.32051589	0.74857729	0.93637834	-0.12576335	0.028548641
ODC1	327.422198	-0.65448587	2.041152	-0.32064534	0.74847918	0.93637834	-0.12582028	0.028548641
VIL1	45.3662337	-0.78721482	2.44434755	-0.32205519	0.74741088	0.93628728	-0.12644058	0.028590876
MYH9	778.896377	-0.34008469	1.05373978	-0.32274068	0.74689164	0.93628728	-0.1267424	0.028590876
PAPOLA	97.2649745	-0.69883141	2.15862636	-0.32373894	0.74613569	0.93628728	-0.12718219	0.028590876
PHTF2	140.338963	-0.62766649	1.93157524	-0.32495058	0.74521848	0.93551093	-0.12771639	0.028951136
CHERP	41.8236913	-0.58729475	1.80662313	-0.32507873	0.74512149	0.93551093	-0.12777291	0.028951136
STAM	72.1557575	-0.7601494	2.32970751	-0.32628534	0.74420848	0.93480667	-0.12830538	0.029278197
SVIL	113.654735	-0.57554587	1.75708406	-0.32755739	0.74324634	0.93387984	-0.12886722	0.029709002
TUBA1B	5.78811831	-0.95454441	2.90697659	-0.32836329	0.74263699	0.93367767	-0.12922342	0.029803029
ZMYND8	669.206147	-0.43074051	1.30792605	-0.32933094	0.74190556	0.93303979	-0.12965137	0.030099833
USO1	17.2612961	-0.8349066	2.53473431	-0.32938624	0.74186377	0.93303979	-0.12967584	0.030099833
POU6F2	3.2505336	-0.71305822	2.16388659	-0.32952661	0.74175768	0.93303979	-0.12973795	0.030099833
NCAPG2	6.93184851	-0.83833896	2.54283332	-0.32968695	0.74163652	0.93303979	-0.1298089	0.030099833
BCL6	302.995587	-0.9327778	2.807131	-0.33228866	0.7396713	0.9319187	-0.13096123	0.030621975
SLC18A2	41.8836672	-0.7473296	2.23809678	-0.33391299	0.73844521	0.93093729	-0.13168172	0.031079573
MED12L	59.5936824	-0.54736121	1.63813413	-0.33413699	0.73827619	0.93093729	-0.13178114	0.031079573
SLMAP	34.0430109	-0.83127995	2.47747914	-0.33553459	0.73722185	0.9306315	-0.1324018	0.031222252
PLCH1	183.16317	-0.86731347	2.58202087	-0.3359049	0.73694258	0.9306315	-0.13256635	0.031222252
ATP2C1	123.388489	-0.59378776	1.76641063	-0.336155	0.73675399	0.9306315	-0.13267751	0.031222252
TTC32	5.86628184	-0.97822579	2.90689841	-0.33651874	0.73647972	0.9306315	-0.13283921	0.031222252
CCNG1	601.433239	-0.74872155	2.22472153	-0.33654619	0.73645903	0.9306315	-0.13285141	0.031222252
CKLF	4.50736299	-0.98442652	2.91125397	-0.33814519	0.73525378	0.9306315	-0.13356273	0.031222252
MTG1	33.8290705	-0.82084827	2.4201723	-0.33916935	0.73448216	0.93044834	-0.13401875	0.031307734
ARID5B	27.1840385	-0.8735769	2.57325692	-0.33948297	0.73424592	0.93044834	-0.13415846	0.031307734
POLR2J	3.73555366	-0.99277086	2.91448589	-0.34063327	0.73337968	0.93044834	-0.13467113	0.031307734
MBD5	10.0497974	-0.64287676	1.87210946	-0.34339699	0.73129982	0.92981144	-0.13590453	0.031605116
HIST1H2AD	67.9702403	-0.68841924	1.99578703	-0.34493622	0.73014232	0.928907	-0.13659248	0.032027765
UXS1	18.6539668	-0.81319113	2.33756299	-0.34787988	0.7279304	0.92727323	-0.13791014	0.032792278
TJP2	376.894661	-0.74087841	2.12358582	-0.34888084	0.72717877	0.92727323	-0.13835881	0.032792278
DNAH11	10.0231144	-0.86546974	2.46288753	-0.35140449	0.72528491	0.92688217	-0.13949136	0.032975471
GMIP	28.6511155	-0.81453719	2.31378844	-0.35203616	0.72481115	0.92665447	-0.13977514	0.033082175
ITM2B	113.020628	-0.47287207	1.34175525	-0.35242797	0.72451733	0.92656332	-0.13995122	0.033124896
KPNA1	23.200489	-0.60103103	1.70270664	-0.35298566	0.72409919	0.92637625	-0.14020194	0.03321259
TG	17.7721633	-0.88326397	2.49941825	-0.35338782	0.72379772	0.92637625	-0.14038279	0.03321259
ARFGEF2	41.881782	-0.88946855	2.49952207	-0.35585545	0.72194882	0.92583766	-0.14149359	0.033465155
SETX	145.82883	-0.48549191	1.36149181	-0.3565882	0.72140012	0.925419	-0.14182379	0.033661587
PNKD	162.749854	-0.6644773	1.85478982	-0.35824938	0.7201567	0.92496375	-0.142573	0.033875285
MTMR14	136.669059	-0.90211189	2.51333887	-0.35892967	0.7196477	0.92488057	-0.14288006	0.033914345
FAM65C	5201.71061	-0.36250103	1.00850316	-0.35944462	0.7192625	0.92484127	-0.14311258	0.033932798
SIRT7	3.45334217	-0.97323661	2.70624417	-0.35962631	0.7191266	0.92484127	-0.14319464	0.033932798
ABC13.47488	67.903144	-0.78040835	2.16646875	-0.36022137	0.71868159	0.92478064	-0.14346348	0.033961271
X1.Mar.1	23.3135962	-1.04208687	2.88866194	-0.36075072	0.71828581	0.92471229	-0.14370272	0.033993369
PRCC	11.4424038	-0.83169757	2.29818589	-0.36189308	0.71743193	0.92431515	-0.1442193	0.034179929
NUP50	26.4915279	-0.65541812	1.80840665	-0.3624285	0.71703184	0.92408561	-0.14446156	0.034287795

TIA1	8.36042004	-0.92668898	2.5531343	-0.36296131	0.71663378	0.92394204	-0.14470272	0.034355271
RABGEF1	5.03424265	-0.8015445	2.20642001	-0.3632783	0.716397	0.92394204	-0.14484624	0.034355271
PTGIR	525.175811	-0.49801772	1.37081934	-0.36329931	0.71638131	0.92394204	-0.14485575	0.034355271
NFE2	1460.3184	-0.39111215	1.07545877	-0.36367005	0.71610441	0.92394204	-0.14502365	0.034355271
CCS	190.430107	-0.61834243	1.6899522	-0.36589344	0.71444459	0.92312855	-0.14603145	0.034737817
PHF21A	4.56992147	-0.87355613	2.3865905	-0.36602682	0.71434506	0.92312855	-0.14609195	0.034737817
TRRAP	21.383152	-0.65421196	1.77960175	-0.36761706	0.71315879	0.92280709	-0.14681376	0.034889078
AKAP7	2.74186538	-1.0813109	2.92203021	-0.37005466	0.71134176	0.9218883	-0.14792169	0.035321698
CEBPZ	15.4640379	-0.86756216	2.34169119	-0.3704853	0.71102093	0.92186522	-0.14811762	0.035332569
ZSWIM6	66.1901925	-0.79272006	2.13782108	-0.37080749	0.71078093	0.92186522	-0.14826424	0.035332569
IRS2	57.4871552	-0.6402729	1.71741978	-0.37281095	0.70928915	0.92066086	-0.14917668	0.035900321
ZFP36	403.3456	-0.42335295	1.13459086	-0.3731327	0.70904968	0.92066086	-0.14932333	0.035900321
HSPB11	2.71527119	-0.98987371	2.64876796	-0.373711	0.70861934	0.92066086	-0.149587	0.035900321
THOC2	470.687842	-0.58339709	1.55451416	-0.37529223	0.70744314	0.92066086	-0.15030846	0.035900321
RELA	4.63909932	-0.58881331	1.56754526	-0.37562763	0.70719375	0.92066086	-0.15046159	0.035900321
NFYB	28.5155843	-0.90272735	2.40120589	-0.3759475	0.70695593	0.92066086	-0.15060766	0.035900321
RRP8	12.672369	-0.8461436	2.2499153	-0.37607798	0.70685892	0.92066086	-0.15066725	0.035900321
LONP2	49.0410811	-0.78691624	2.08598713	-0.37723926	0.70599581	0.92066086	-0.15119788	0.035900321
APP	7.36242964	-0.91081207	2.41063851	-0.37783022	0.70555672	0.92066086	-0.15146807	0.035900321
CAMTA1	51.703956	-0.83480829	2.18906802	-0.38135329	0.70294112	0.92066086	-0.15308105	0.035900321
RHD	42.7310679	-0.87622818	2.28853743	-0.38287693	0.70181102	0.92013258	-0.15377982	0.03614959
ZCCHC17	5.96455186	-0.98551287	2.56759228	-0.38382764	0.7011062	0.92012882	-0.15421619	0.036151365
KIAA1683	257.928194	-1.1050063	2.86520719	-0.38566366	0.69974578	0.91945138	-0.15505971	0.036471231
FAM212B	60.5158504	-0.69244876	1.79012468	-0.38681594	0.69889248	0.91920034	-0.15558964	0.036589825
RSBN1	60.9892312	-1.11884205	2.89054204	-0.38706998	0.6987044	0.91920034	-0.15570652	0.036589825
SQSTM1	149.945546	-0.47721135	1.22721042	-0.38885862	0.69738073	0.918082	-0.15653006	0.037118527
MYOM1	163.032355	-0.67885431	1.74472378	-0.38908985	0.69720968	0.918082	-0.15663659	0.037118527
BID	4.93420721	-1.13678302	2.91079406	-0.39054052	0.6961369	0.918082	-0.15730534	0.037118527
AFF4	109.366409	-0.82271406	2.10323059	-0.39116684	0.69567392	0.918082	-0.15759428	0.037118527
ABI1	75.1939252	-0.87289542	2.2255456	-0.39221637	0.69489835	0.91771542	-0.15807872	0.037291969
FKBP1B	29.2575188	-0.9191253	2.34126507	-0.39257635	0.69463241	0.91771542	-0.15824496	0.037291969
HIST1H2BC	143.806879	-0.70902221	1.80364521	-0.39310514	0.69424183	0.91771542	-0.15848922	0.037291969
DENND5B	10.8264986	-0.58120332	1.46190088	-0.39756685	0.6909495	0.91656022	-0.16055369	0.037838996
TNNC2	11.9165668	-1.11011818	2.78565945	-0.39851181	0.69025296	0.91593796	-0.16099172	0.038133943
WASL	163.308721	-0.68667337	1.71381704	-0.40066901	0.68866383	0.91503075	-0.16199273	0.038564309
ERCC6	21.1578709	-1.00958791	2.51918385	-0.40075992	0.68859689	0.91503075	-0.16203494	0.038564309
ZNF746	42.2244877	-0.95311074	2.37608856	-0.40112593	0.68832741	0.91503075	-0.16220494	0.038564309
GADD45B	3.25851336	-0.9567908	2.3843742	-0.40127544	0.68821734	0.91503075	-0.16227439	0.038564309
TNK2	78.9828321	-0.58153337	1.44166728	-0.40337558	0.68667195	0.91497664	-0.16325069	0.038589993
SLFN14	367.494669	-0.66312782	1.64389093	-0.40338919	0.68666194	0.91497664	-0.16325702	0.038589993
ATP2A3	734.080148	-0.47812565	1.1808341	-0.40490502	0.68554733	0.91494201	-0.16396256	0.03860643
HIST1H2AI	12.9137966	-0.87736491	2.16545422	-0.40516438	0.68535669	0.91494201	-0.16408334	0.03860643
TMED5	3.34582611	-1.0956378	2.69699928	-0.40624327	0.68456386	0.91482038	-0.16458603	0.038664168
SPTSSA	19.7626564	-0.94209967	2.31837861	-0.40636144	0.68447705	0.91482038	-0.16464111	0.038664168
SMIM5	71.2039699	-0.80075366	1.96361259	-0.40779615	0.68342334	0.91475714	-0.1653102	0.038694192
UQCRH	75.9036538	-0.8828679	2.16361796	-0.40805166	0.68323575	0.91475714	-0.16542942	0.038694192
USP20	224.45622	-0.75772678	1.85682124	-0.4080774	0.68321685	0.91475714	-0.16544143	0.038694192
RAD21	12.159148	-0.85814835	2.10219564	-0.40821526	0.68311564	0.91475714	-0.16550577	0.038694192
LTBP1	712.630367	-0.4720246	1.14778458	-0.41124842	0.68089038	0.91341094	-0.1669228	0.03933379
TBC1D3L	25.1744711	-0.91025564	2.21251075	-0.41141298	0.68076974	0.91341094	-0.16699976	0.03933379
SNRNP70	17.8140159	-0.930406	2.25839586	-0.41197649	0.68035666	0.9132834	-0.16726336	0.039394434
SPATA22	11.7798243	-0.3646379	0.8796695	-0.41451692	0.67849558	0.91196113	-0.16845298	0.040023672
DBN1	86.6991258	-0.87775167	2.11305011	-0.41539558	0.67785234	0.91168513	-0.1688649	0.040155129
VANGL1	11.4977164	-1.09883058	2.64443843	-0.41552512	0.67775753	0.91168513	-0.16892565	0.040155129
LSMEM1	18.3125879	-1.10832404	2.66059647	-0.41656976	0.67699313	0.91141267	-0.16941574	0.040284938
NUFIP2	17.3419802	-0.85052974	2.0286372	-0.41926163	0.67502494	0.9102344	-0.17068018	0.040846755
ZNF7	32.3900022	-1.2217456	2.89237358	-0.42240242	0.67273131	0.90890759	-0.17215836	0.041480271
SEC16A	98.4810104	-0.89525225	2.119358	-0.42241672	0.67272088	0.90890759	-0.17216509	0.041480271
GPR55	14.8894165	-1.05497564	2.49698088	-0.42250049	0.67265974	0.90890759	-0.17220456	0.041480271
ANKRD55	77.1060797	-0.54149486	1.27699035	-0.4240399	0.67153671	0.90817761	-0.17293024	0.041829208
SENP5	153.244884	-0.72507889	1.70891227	-0.42429263	0.67135241	0.90817761	-0.17304945	0.041829208
FYB	188.836298	-0.77608949	1.82896001	-0.42433377	0.67132242	0.90817761	-0.17306885	0.041829208

DAG1	16.7035568	-1.2013896	2.82598492	-0.42512244	0.67074742	0.90817761	-0.17344099	0.041829208
DNM1	40.1664335	-1.0109201	2.3727069	-0.42606194	0.67006271	0.90817761	-0.17388455	0.041829208
MAGED2	235.04929	-0.78131449	1.82932759	-0.42710474	0.66930304	0.90781038	-0.1743772	0.042004858
TADA2B	9.70646598	-1.08897234	2.54887655	-0.4272362	0.6692073	0.90781038	-0.17443933	0.042004858
ULK4	5.2585229	-0.94137604	2.20323625	-0.42726968	0.66918292	0.90781038	-0.17445516	0.042004858
HIST1H2BK	111.657313	-0.82097341	1.89828795	-0.43248097	0.66539188	0.90575083	-0.1769225	0.042991258
KAT6A	209.192224	-0.47805207	1.10359081	-0.43317873	0.66488493	0.90535672	-0.17725351	0.043180272
SGMS1	100.904029	-1.02337031	2.36228946	-0.43321123	0.66486132	0.90535672	-0.17726893	0.043180272
STON2	507.738298	-0.60112377	1.38731334	-0.43330065	0.66479637	0.90535672	-0.17731136	0.043180272
TRIM28	27.3160434	-0.97711101	2.2543959	-0.43342476	0.66470621	0.90535672	-0.17737026	0.043180272
DNAJC6	58.8213982	-0.89968172	2.07507988	-0.43356486	0.66460446	0.90535672	-0.17743675	0.043180272
HEXIM1	184.240894	-0.58227006	1.34178638	-0.43395138	0.66432375	0.90535672	-0.17762022	0.043180272
HIST2H2BE	616.011506	-0.80933347	1.86211258	-0.43463187	0.66382966	0.90535672	-0.17794335	0.043180272
AP2B1	103.082483	-0.86252857	1.97562506	-0.43658515	0.66241223	0.90535672	-0.17887166	0.043180272
DSCR3	5.31768261	-1.03796083	2.37711373	-0.43664753	0.66236699	0.90535672	-0.17890132	0.043180272
HP1BP3	50.3785332	-0.85358408	1.95068489	-0.43758174	0.66168951	0.90535672	-0.17934575	0.043180272
GON4L	23.1752662	-1.05039116	2.39346965	-0.4388571	0.66076508	0.90535672	-0.17995292	0.043180272
INAFM2	33.528845	-1.06500647	2.42038257	-0.44001576	0.6599257	0.90535672	-0.18050496	0.043180272
CALR	15.5008714	-1.05995602	2.4058322	-0.4405777	0.65951875	0.90535672	-0.18077285	0.043180272
RASA3	554.733005	-0.56812562	1.28642522	-0.44163129	0.65875604	0.90484146	-0.18127539	0.043427509
FAM160B1	16.0306783	-1.10680031	2.50303151	-0.44218393	0.65835611	0.90475078	-0.18153913	0.043471034
IRF2	11.7663713	-0.7349917	1.6588499	-0.44307306	0.65771289	0.90416523	-0.18196365	0.043752199
RTN4	52.4995377	-0.95900391	2.16181076	-0.4436114	0.65732356	0.90392844	-0.1822208	0.043865951
B2M	3765.80348	-0.62816454	1.41469161	-0.44402931	0.65702139	0.90381139	-0.18242049	0.043922189
DHCR7	13.9574002	-0.90161314	1.99481836	-0.45197756	0.65128515	0.89978479	-0.18622883	0.045861354
BRWD1	72.5444943	-0.87163696	1.91851443	-0.45432911	0.64959196	0.89801949	-0.18735936	0.046714239
PRPF4B	38.2383839	-0.86888249	1.91149606	-0.45455625	0.64942851	0.89801949	-0.18746865	0.046714239
RCN2	2.86695026	-0.54044549	1.18547468	-0.45588953	0.64846942	0.89801949	-0.1881105	0.046714239
CNTRL	4.33718708	-1.09329315	2.39083809	-0.45728448	0.64746658	0.89748697	-0.18878264	0.046971849
USP47	228.536147	-0.95965642	2.09614628	-0.45781939	0.6470822	0.89748697	-0.18904054	0.046971849
SBNO1	348.425646	-0.82403783	1.79969104	-0.45787738	0.64704054	0.89748697	-0.18906851	0.046971849
PLEKHO1	266.343965	-0.57614675	1.25782482	-0.45805007	0.64691647	0.89748697	-0.18915179	0.046971849
VIM	47.1282649	-1.05836032	2.30003495	-0.46014967	0.6454088	0.89660412	-0.19016512	0.047399272
RLF	4.3528806	-1.34181595	2.91420722	-0.46043944	0.64520083	0.89660412	-0.19030508	0.047399272
ING4	84.3597379	-1.1755355	2.55217167	-0.46060205	0.64508414	0.89660412	-0.19038363	0.047399272
PTPN1	116.955213	-0.70725697	1.53316162	-0.4613062	0.64457893	0.8964685	-0.19072389	0.047464967
TNFAIP3	353.102838	-1.29522911	2.80249871	-0.46216939	0.64395985	0.89620616	-0.19114121	0.047592075
SERPINB1	70.2314232	-1.0342651	2.2284917	-0.46410992	0.642569	0.89510039	-0.19208023	0.048128254
PPP2R2A	3.5179303	-0.96103158	2.06477799	-0.46544063	0.64161594	0.89443883	-0.19272485	0.048449357
LCORL	3.34563039	-1.09871547	2.35070649	-0.46739798	0.64021517	0.89368285	-0.19367404	0.048816574
LGALS12	301.962576	-0.83185141	1.77953754	-0.4674537	0.64017531	0.89368285	-0.19370108	0.048816574
LYST	225.696013	-0.64378625	1.37705987	-0.46750781	0.6401366	0.89368285	-0.19372734	0.048816574
PSME4	195.050708	-0.70425864	1.50339241	-0.46844632	0.63946545	0.89368285	-0.19418292	0.048816574
RC3H2	122.954612	-1.01310796	2.15663277	-0.46976378	0.63852379	0.89368285	-0.19482291	0.048816574
MAN2A2	312.933777	-0.74827195	1.58783572	-0.47125275	0.63746025	0.89343133	-0.19554689	0.04893882
SH3TC2	14.4211861	-1.04597627	2.21814844	-0.47155378	0.63724533	0.89343082	-0.19569334	0.04893907
CEP95	7.17183347	-1.3260141	2.79417513	-0.4745637	0.63509798	0.89192175	-0.19715927	0.049673243
GNAZ	8.48886775	-1.15794711	2.42998829	-0.47652374	0.6337013	0.89109937	-0.1981154	0.050073864
CIC	14.1432923	-1.11738565	2.34262873	-0.4769794	0.63337679	0.89100708	-0.19833786	0.050118846
PPTC7	12.1078951	-1.38858818	2.89928256	-0.478942	0.63197989	0.89024502	-0.19929674	0.050490449
ANP32B	3.46782993	-0.90637761	1.88681144	-0.4803753	0.63096055	0.88945902	-0.19999779	0.050874055
ERICH1	212.687602	-0.61336931	1.27505514	-0.48105317	0.63047871	0.88945902	-0.20032957	0.050874055
EZH2	34.7007816	-0.94228475	1.95501433	-0.48198355	0.62981763	0.88945902	-0.20078519	0.050874055
ACRBP	859.830012	-0.56328945	1.16703246	-0.48266819	0.62933135	0.88945902	-0.20112063	0.050874055
CD164	69.1752294	-1.23672566	2.559063	-0.48327285	0.62890202	0.88945902	-0.20141701	0.050874055
ATG12	50.5073695	-0.84419052	1.74371166	-0.48413424	0.6282906	0.88895755	-0.20183944	0.051118978
ARFGEF1	30.2633567	-0.70441952	1.45454101	-0.4842899	0.62818014	0.88895755	-0.2019158	0.051118978
MCU	8.55637881	-1.1840892	2.44472473	-0.48434459	0.62814133	0.88895755	-0.20194263	0.051118978
PKD1	3.48340343	-0.8528968	1.76021565	-0.48454108	0.62800191	0.88895755	-0.20203903	0.051118978
ZYX	75.5827595	-0.95110513	1.96005379	-0.4852444	0.62750299	0.88895755	-0.2023842	0.051118978
DMXL1	29.7062033	-1.1531472	2.37552138	-0.4854291	0.627372	0.88895755	-0.20247487	0.051118978
PRR29	141.988808	-0.68009599	1.40088022	-0.48547761	0.62733759	0.88895755	-0.20249869	0.051118978

SH3GLB2	19.8589847	-1.21243584	2.49555687	-0.48583779	0.62708218	0.88895755	-0.20267554	0.051118978
PTBP1	99.7435465	-0.84634143	1.74069733	-0.48620827	0.62681951	0.88895755	-0.20285749	0.051118978
AAK1	4.4864614	-0.87660178	1.79789083	-0.48757231	0.62585281	0.88895755	-0.20352779	0.051118978
TAB2	43.915274	-1.00405036	2.05905363	-0.4876271	0.62581399	0.88895755	-0.20355473	0.051118978
XPNPEP1	337.388908	-0.66294449	1.35777409	-0.48825832	0.62536688	0.88895755	-0.20386512	0.051118978
WDR45B	35.8796273	-1.3585011	2.77516553	-0.48952075	0.62447307	0.88895755	-0.20448629	0.051118978
CRAMP1	27.7397928	-1.12006649	2.28784637	-0.48957242	0.62443649	0.88895755	-0.20451172	0.051118978
SEC63	11.9483292	-1.14912385	2.33616567	-0.49188457	0.62280095	0.88794166	-0.20565074	0.051615567
ESAM	280.680273	-0.68536849	1.3918252	-0.49242426	0.62241945	0.88789127	-0.20591684	0.051640216
PPP4R3B	9.37980238	-1.11196812	2.25734357	-0.4926003	0.62229504	0.88789127	-0.20600366	0.051640216
PATL1	3.33153372	-1.29519977	2.62246482	-0.49388642	0.62138639	0.88732954	-0.20663826	0.051915059
SNX17	20.8839485	-1.42435673	2.8802589	-0.49452385	0.62093627	0.88729534	-0.20695297	0.051931801
PIKFYVE	6.8314926	-0.95776402	1.93596539	-0.49472166	0.62079661	0.88729534	-0.20705066	0.051931801
TUBB	3.10984222	-1.17420667	2.36783814	-0.4958982	0.61996624	0.88662815	-0.20763196	0.052258485
TMEM39B	60.6398183	-1.30207412	2.62489652	-0.49604779	0.61986069	0.88662815	-0.2077059	0.052258485
CLTA	13.5523171	-1.14260823	2.30032721	-0.49671552	0.61938968	0.8866066	-0.20803604	0.052269038
MAN2C1	7.06961442	-1.44777233	2.90712389	-0.49800847	0.61847807	0.88560615	-0.20867569	0.052759375
PTGDR	385.808112	-1.12193975	2.25096648	-0.49842579	0.61818396	0.88548951	-0.20888227	0.052816577
PJA2	254.107182	-1.25561385	2.51816383	-0.49862278	0.61804515	0.88548951	-0.2089798	0.052816577
FUT11	18.3915398	-1.43824569	2.88297758	-0.49887509	0.61786739	0.88548951	-0.20910473	0.052816577
CNOT10	2.83263346	-1.05217121	2.10292744	-0.50033643	0.61683821	0.88501124	-0.20982873	0.053051215
HLA.G	5.16141818	-0.56012466	1.11811277	-0.50095543	0.61640249	0.88476387	-0.21013561	0.053172622
CDKN2D	26.4706382	-1.05546851	2.10678748	-0.5009848	0.61638182	0.88476387	-0.21015018	0.053172622
MAFG	63.203241	-1.13208509	2.25021574	-0.50310069	0.61489348	0.88385668	-0.21120011	0.053618152
DDX24	162.301202	-1.39930688	2.77773252	-0.50375868	0.61443096	0.88376184	-0.21152691	0.053664753
HNRNPLL	150.754905	-0.67369979	1.33421683	-0.50494026	0.6136008	0.88347601	-0.21211409	0.053805238
NID2	5.67603434	-1.4704828	2.91068921	-0.5052009	0.61341774	0.88347601	-0.21224367	0.053805238
EGFL7	2194.63719	-0.57393692	1.13543434	-0.50547787	0.61322324	0.88347601	-0.21238139	0.053805238
SMARCA5	20.5692921	-1.21807417	2.40927681	-0.50557668	0.61315386	0.88347601	-0.21243054	0.053805238
FIP1L1	10.3719377	-1.16286436	2.29702381	-0.50624828	0.61268236	0.88347601	-0.21276462	0.053805238
EP300	349.41462	-0.52138425	1.02815149	-0.50710839	0.61207877	0.88347601	-0.21319269	0.053805238
SPG20	77.3771732	-1.44911895	2.85508543	-0.50755713	0.61176396	0.88347601	-0.21341611	0.053805238
NPIPB11	5.76850651	-0.83318258	1.64008777	-0.50801097	0.61144565	0.88347601	-0.21364214	0.053805238
ZSCAN29	179.963735	-1.0746342	2.11304802	-0.50857065	0.61105321	0.88347601	-0.21392097	0.053805238
DOK2	32.9658525	-1.05051522	2.04873634	-0.51276252	0.60811745	0.88237111	-0.21601254	0.054348718
PFKP	41.8452341	-1.1456236	2.22024173	-0.51599048	0.60586106	0.88179148	-0.21762696	0.0546341
RASGRP2	377.036494	-0.66071594	1.27950258	-0.516385	0.60558554	0.88179148	-0.2178245	0.0546341
CMPK1	3.72547242	-1.41158934	2.70120537	-0.52257757	0.60126825	0.87848456	-0.22093173	0.056265866
DENND4C	220.313227	-0.79494063	1.52001614	-0.5229817	0.60098699	0.87838182	-0.22113493	0.05631666
ARHGEF3	7.9273542	-1.34874681	2.57781809	-0.52321256	0.60082634	0.87838182	-0.22125104	0.05631666
AGO3	8.40777258	-0.8324053	1.58980585	-0.52358928	0.60056424	0.87838182	-0.22144053	0.05631666
REST	11.7602636	-1.41689289	2.69123072	-0.52648511	0.5985512	0.87728518	-0.2228987	0.056859209
LRRFIP1	81.111125	-1.07641151	2.04248012	-0.52701199	0.59818526	0.87728518	-0.22316429	0.056859209
DYRK1A	126.181656	-0.76997364	1.45692538	-0.52849216	0.59715779	0.87709525	-0.2239109	0.056953241
TUBA4A	417.279693	-0.71300178	1.34881688	-0.52861274	0.59707412	0.87709525	-0.22397175	0.056953241
VWF	140.483356	-1.07732448	2.02911565	-0.53093301	0.5954652	0.8755357	-0.22514361	0.057726142
GOLGA2	210.193838	-0.90684112	1.70336199	-0.53238309	0.59446069	0.87539959	-0.22587686	0.057793661
SCN1B	9.32401889	-1.12465803	2.1079346	-0.53353554	0.59366292	0.87535849	-0.22646008	0.057814051
SLC38A2	122.1099	-0.72029952	1.34529066	-0.53542297	0.59235741	0.87412711	-0.22741617	0.058425411
DENND4A	270.486675	-0.9684206	1.80284724	-0.53716176	0.59115588	0.87412711	-0.22829798	0.058425411
TBC1D20	40.8584346	-1.07570257	2.00216768	-0.53726897	0.59108183	0.87412711	-0.22835239	0.058425411
TUBB4B	32.2171933	-1.14567608	2.13222805	-0.53731405	0.5910507	0.87412711	-0.22837526	0.058425411
CSNK1G1	4.65857374	-1.48984023	2.75436476	-0.54090157	0.58857543	0.8724913	-0.23019787	0.059238893
WRB	3.31625001	-1.19241299	2.20115634	-0.54172117	0.5880106	0.87196444	-0.23061484	0.059501226
ZNF585B	4.90843328	-1.28624592	2.37301356	-0.54203058	0.58779744	0.87196444	-0.23077231	0.059501226
GIPC3	28.0405802	-1.24199633	2.28913379	-0.5425617	0.58743161	0.87196444	-0.23104268	0.059501226
RBM23	40.891948	-1.36522442	2.51376978	-0.54309843	0.58706203	0.87196444	-0.23131601	0.059501226
MAP4K4	9.76445959	-1.05049455	1.93347387	-0.54331975	0.58690967	0.87196444	-0.23142874	0.059501226
GNG8	16.1961005	-0.63294324	1.15687864	-0.54711291	0.58430117	0.8701005	-0.23336325	0.060430581
ZNF160	3.11083194	-1.0664893	1.94118845	-0.54940019	0.58273084	0.86890053	-0.23453199	0.061029936
HDAC5	675.664924	-0.61939254	1.12384388	-0.55113753	0.5815394	0.86762094	-0.23542086	0.061669973
PPIL4	3.33883058	-1.61202415	2.92263767	-0.55156483	0.58124654	0.86749483	-0.23563962	0.061733103

MAN2A1	8.11648206	-1.2080385	2.1800251	-0.55413972	0.57948323	0.86628875	-0.23695913	0.062337328
ATG2B	52.5089815	-1.09216733	1.97077632	-0.55418127	0.57945479	0.86628875	-0.23698044	0.062337328
JMJD1C	63.3778092	-1.09622022	1.97580146	-0.55482306	0.57901569	0.86628875	-0.23730967	0.062337328
GBF1	298.588686	-0.84830041	1.52836305	-0.55503855	0.5788683	0.86628875	-0.23742024	0.062337328
EML3	11.4736826	-1.15040486	2.07263828	-0.55504372	0.57886476	0.86628875	-0.23742289	0.062337328
PLEKHA3	13.7346081	-1.44891441	2.60909483	-0.55533221	0.57866745	0.86628875	-0.23757095	0.062337328
TELO2	62.9442964	-1.30695215	2.35048599	-0.55603486	0.57818702	0.86628875	-0.23793166	0.062337328
AP3M1	4.1433126	-1.41520877	2.54412582	-0.55626525	0.57802954	0.86628875	-0.23804996	0.062337328
KCNK6	274.871621	-0.80919242	1.4543828	-0.55638201	0.57794973	0.86628875	-0.23810993	0.062337328
TMEM135	4.4354253	-1.41714129	2.53928981	-0.55808569	0.57678588	0.86628875	-0.23898538	0.062337328
MINK1	349.817339	-0.69606841	1.24672177	-0.55831897	0.5766266	0.86628875	-0.23910533	0.062337328
SYNRG	5.58559403	-1.40277613	2.50726423	-0.55948476	0.57583093	0.86592993	-0.23970501	0.062517248
PDLIM5	17.6228309	-1.25086098	2.23273624	-0.56023679	0.57531794	0.86547106	-0.24009209	0.06274745
HIST1H2BN	222.023623	-0.70672935	1.25940432	-0.56116161	0.57468737	0.86529969	-0.24056835	0.06283345
CAPN2	142.19874	-1.12764242	2.0092287	-0.56123149	0.57463974	0.86529969	-0.24060435	0.06283345
WDR26	165.834223	-0.81645572	1.45152793	-0.5624802	0.57378889	0.86473288	-0.24124786	0.063118027
MYO9A	4.22540259	-1.24398053	2.20918805	-0.563094	0.57337088	0.86441576	-0.24156437	0.063277322
CLN8	38.571653	-1.22986203	2.18378946	-0.56317793	0.57331373	0.86441576	-0.24160766	0.063277322
VPS9D1	38.4171471	-1.14681413	2.03094994	-0.56466883	0.57229904	0.86393474	-0.24237698	0.063519062
SLC25A45	66.9412365	-1.13264694	2.00533531	-0.56481673	0.57219842	0.86393474	-0.24245334	0.063519062
SLC26A4	4.76815423	-1.42637401	2.51843649	-0.56637283	0.57114036	0.86324082	-0.24325715	0.063868032
TAP1	149.189342	-1.1099896	1.95067169	-0.56902943	0.56933617	0.86082637	-0.24463122	0.06508444
RSPH9	18.7493742	-1.42015105	2.49459209	-0.56929189	0.56915807	0.86082637	-0.2447671	0.06508444
PTDSS2	109.883921	-1.25999875	2.20830614	-0.5705725	0.56828947	0.85986822	-0.24543039	0.065568103
PSTPIP2	430.505707	-0.86823089	1.51661917	-0.57247786	0.56699828	0.85853849	-0.24641826	0.066240228
EFCAB6	55.4697141	-1.28620463	2.2456694	-0.57274888	0.56681474	0.85853849	-0.24655887	0.066240228
LENG8	105.787045	-0.82719955	1.44405341	-0.57283169	0.56675866	0.85853849	-0.24660183	0.066240228
WNT11	13.3227421	-1.18395804	2.06482484	-0.57339394	0.56637799	0.85853849	-0.24689363	0.066240228
VPS37B	46.724968	-0.91065959	1.58786017	-0.57351372	0.56629691	0.85853849	-0.24695581	0.066240228
FAM46C	323.956311	-0.86799098	1.51287911	-0.57373452	0.56614746	0.85853849	-0.24707044	0.066240228
LMNA	105.361609	-0.90944214	1.58134531	-0.57510661	0.5652192	0.85802871	-0.2477831	0.066498182
DGKD	4276.16594	-0.68832121	1.19324359	-0.57684887	0.56404155	0.85658885	-0.2486889	0.067227583
RAB30	216.522858	-1.2766851	2.19505167	-0.58161961	0.56082293	0.85290967	-0.25117424	0.069096961
RHOC	61.7507517	-1.19620645	2.05507057	-0.58207561	0.56051576	0.85275397	-0.25141218	0.069176252
MDM2	43.0143211	-1.01408493	1.73787921	-0.58351865	0.55954421	0.85189838	-0.2521656	0.069612207
ZNF891	21.7780678	-1.57120784	2.69258441	-0.58353151	0.55953555	0.85189838	-0.25217231	0.069612207
GLG1	22.0271032	-0.93637783	1.59868055	-0.58571916	0.55806426	0.85189838	-0.25331579	0.069612207
MON2	16.40446	-1.300022	2.21393581	-0.5871995	0.55706973	0.85155593	-0.25409044	0.069786822
RBM7	6.82364649	-1.39855171	2.37573375	-0.58868201	0.55607461	0.85128481	-0.25486693	0.069925116
C16orf54	14.9317541	-1.55969807	2.64933498	-0.58871305	0.55605378	0.85128481	-0.2548832	0.069925116
PPP1R15B	88.2500232	-1.56064201	2.64645844	-0.58970962	0.55538534	0.85085525	-0.25540559	0.070144318
STX16	16.6408016	-1.33419961	2.25274549	-0.59225492	0.5536799	0.84917977	-0.25674125	0.071000359
RNPS1	60.5017212	-1.24206652	2.09577411	-0.59265286	0.5534135	0.84908394	-0.25695025	0.071049376
SKAP2	4.07752314	-1.32736656	2.23811549	-0.59307331	0.55313209	0.84898418	-0.25717114	0.071100401
SORT1	3.46161004	-1.56392161	2.63283238	-0.59400728	0.55250724	0.84898418	-0.25766203	0.071100401
PARD3	207.708096	-1.03369063	1.73982627	-0.5941344	0.55242223	0.84898418	-0.25772886	0.071100401
LHFPL4	49.8897353	-1.672921	2.81212224	-0.59489626	0.55191282	0.84898418	-0.25812952	0.071100401
CNOT4	50.7842322	-1.18747877	1.99047666	-0.5965801	0.55078776	0.84849435	-0.25901572	0.071351045
RNF138	20.7328008	-1.37814691	2.30715137	-0.59733701	0.5502824	0.84802957	-0.25941438	0.071589004
PRRC2C	222.093119	-0.83151535	1.39016338	-0.59814218	0.54974507	0.84751517	-0.25983866	0.07185252
CBFA2T3	431.579365	-0.73796469	1.22921757	-0.60035319	0.54827088	0.84581792	-0.26100482	0.072723117
SFPQ	67.8725259	-1.04642489	1.73674321	-0.60252136	0.54682715	0.84426705	-0.26214993	0.073520159
MTFR1L	106.48602	-1.47014455	2.43838216	-0.60291802	0.54656323	0.84426705	-0.26235959	0.073520159
PPM1A	144.36928	-0.92178631	1.52627666	-0.60394444	0.54588058	0.84374415	-0.26290236	0.073789227
CTNNAL1	83.6425188	-0.9312417	1.5409827	-0.60431678	0.54563305	0.84367472	-0.26309933	0.073824963
TMEM55B	8.90497747	-1.67467792	2.76385867	-0.60592024	0.54456772	0.84335319	-0.26394811	0.073990507
HIST1H2BH	139.458022	-1.13144755	1.8619776	-0.60765905	0.54341362	0.84306048	-0.26486948	0.074141268
RBM39	439.381735	-0.88591527	1.44913219	-0.61134193	0.54097324	0.84052708	-0.26682422	0.075448292
MTCH1	61.8323898	-1.71014492	2.79490687	-0.61187903	0.54061779	0.84028835	-0.26710966	0.075571657
CASP3	12.9668221	-1.79650253	2.90071374	-0.6193312	0.53569819	0.83607469	-0.27107982	0.077754922
NUP153	3.43605403	-1.53461396	2.47233452	-0.62071453	0.53478747	0.83496626	-0.27181878	0.078331073
DOCK5	24.4141735	-1.39293233	2.23726323	-0.62260547	0.53354381	0.83396263	-0.27282991	0.078853411

SEL1L	36.7589923	-1.61279856	2.58941922	-0.62284181	0.53338848	0.83396263	-0.27295637	0.078853411
USF2	16.2545219	-1.37874738	2.21318617	-0.62296945	0.53330459	0.83396263	-0.27302467	0.078853411
TRMT1L	3.73316063	-1.55810563	2.49414378	-0.62470562	0.53216428	0.83305717	-0.27395428	0.079325192
HIPK3	43.2680528	-1.75873665	2.81088521	-0.62568782	0.53151972	0.83236108	-0.27448062	0.079688233
WASHC1	7.2795596	-0.9867082	1.57662587	-0.62583534	0.53142295	0.83236108	-0.2745597	0.079688233
BLZF1	14.3659973	-1.35307371	2.15845592	-0.62687113	0.53074371	0.83208464	-0.27511514	0.079832494
BUB1	6.00658929	-1.71350529	2.73216666	-0.62715987	0.53055445	0.83208464	-0.27527004	0.079832494
SLC35E2B	7.08345222	-1.37545859	2.19130807	-0.62768837	0.5302081	0.83208464	-0.27555364	0.079832494
ABHD16A	1083.61424	-0.80157677	1.27263138	-0.62985777	0.52878764	0.830895	-0.2767187	0.080453856
PTK2	396.483493	-0.83646572	1.32670365	-0.63048423	0.52837782	0.83067637	-0.27705542	0.080568142
TUBB1	213.175326	-0.81002026	1.28454164	-0.6305909	0.52830805	0.83067637	-0.27711277	0.080568142
SERPINB9	195.976843	-1.28683246	2.03899932	-0.63110981	0.52796873	0.83067637	-0.2773918	0.080568142
SETDB1	10.1155232	-1.76063227	2.78770277	-0.63157101	0.52766723	0.83067637	-0.27763987	0.080568142
SLC35E1	2.86262361	-1.34173277	2.12411815	-0.63166579	0.52760529	0.83067637	-0.27769086	0.080568142
RCSD1	15.2823169	-1.36708902	2.16093306	-0.6326383	0.52696987	0.83067637	-0.27821422	0.080568142
EHD3	307.778742	-1.03636777	1.63468195	-0.63398741	0.52608904	0.830415	-0.27894074	0.080704815
UBQLN1	20.312492	-1.41406994	2.22980289	-0.63416814	0.5259711	0.830415	-0.27903812	0.080704815
CCDC92	116.873284	-1.26527265	1.98790097	-0.63648676	0.52445921	0.8300449	-0.28028829	0.080898414
XRCC5	46.9630833	-1.77672995	2.79108669	-0.63657283	0.52440313	0.8300449	-0.28033473	0.080898414
STRN4	474.836461	-1.0502521	1.64682785	-0.63774249	0.52364132	0.82969652	-0.28096609	0.081080732
ZNF844	3.89393644	-1.32116776	2.07136649	-0.63782424	0.5235881	0.82969652	-0.28101024	0.081080732
TSC22D2	174.780968	-1.23435668	1.93351501	-0.63840036	0.5232131	0.8296494	-0.28132139	0.081105396
CTNNB1	658.076749	-0.91633058	1.43512301	-0.63850316	0.5231462	0.8296494	-0.28137693	0.081105396
LRRK1	3.68416791	-1.82939794	2.86115774	-0.6393908	0.52256873	0.82957537	-0.28185658	0.08114415
KSR1	12.4131716	-1.78971701	2.79847977	-0.63953187	0.52247699	0.82957537	-0.28193283	0.08114415
LY6G6E	10.6820754	-1.2612166	1.96694653	-0.64120533	0.52138929	0.82865713	-0.2828379	0.08162513
KIAA0513	604.285877	-0.90397611	1.40910524	-0.64152491	0.5211817	0.82865713	-0.28301084	0.08162513
TAGLN2	202.020016	-0.8702776	1.35644321	-0.64158793	0.52114077	0.82865713	-0.28304495	0.08162513
ARID1B	35.0174313	-1.00449792	1.56443067	-0.64208529	0.52081781	0.82865713	-0.28331418	0.08162513
CYB5R3	73.9870999	-1.40708916	2.18974148	-0.64258232	0.52049516	0.82865713	-0.28358331	0.08162513
CNOT1	73.9427336	-1.51412149	2.34861852	-0.644686	0.51913069	0.82840419	-0.2847233	0.081757715
DNAH8	5.50994869	-1.53767784	2.37788286	-0.64665837	0.51785307	0.82681756	-0.28579344	0.082590307
GNAQ	130.24172	-1.21064519	1.87151838	-0.6468786	0.51771052	0.82681756	-0.28591301	0.082590307
SPOPL	12.3451212	-1.71027897	2.64121703	-0.64753443	0.51728612	0.8265462	-0.28626918	0.082732867
FGD3	206.762112	-1.3517375	2.08482152	-0.64837085	0.51674512	0.8261362	-0.28672362	0.082948346
FBXO18	143.709208	-0.95420062	1.47163905	-0.64839311	0.51673073	0.8261362	-0.28673571	0.082948346
PRR14L	9.0957013	-1.48366592	2.28377301	-0.6496556	0.51591471	0.82562216	-0.28742209	0.08321866
KRAS	69.9160645	-1.61118271	2.47825165	-0.65012877	0.51560904	0.82545023	-0.28767947	0.083309105
VCL	765.900459	-0.74163888	1.13719102	-0.65216737	0.51429321	0.82397727	-0.28878921	0.084084771
RFX3	3.20122963	-1.20344914	1.84045258	-0.65388761	0.51318422	0.82283369	-0.28972671	0.084687937
FERMT3	1015.26033	-0.92550201	1.41031416	-0.65623819	0.51167088	0.82103952	-0.2910093	0.085635939
TPM4	75.8184109	-1.03374861	1.57197553	-0.65761113	0.51078804	0.81993886	-0.29175928	0.086218532
RAB14	3.35817842	-1.26504697	1.92200038	-0.65819288	0.5104142	0.81965473	-0.29207725	0.086369051
PAPD5	5.46405891	-1.39313201	2.1164101	-0.65825239	0.51037596	0.81965473	-0.29210979	0.086369051
ZNF185	67.8092425	-1.23898661	1.87794492	-0.65975663	0.50941002	0.8189897	-0.29293252	0.08672156
VDAC2	2.71791767	-1.93499527	2.93287857	-0.65975976	0.50940801	0.8189897	-0.29293423	0.08672156
SLC15A2	3.27963106	-1.93289293	2.92739008	-0.66027857	0.50907508	0.8189897	-0.29321816	0.08672156
ARID4A	15.9948232	-1.37570292	2.08321449	-0.66037507	0.50901316	0.8189897	-0.29327099	0.08672156
OTUD5	65.9789151	-1.15458189	1.74747799	-0.66071327	0.50879621	0.8189897	-0.29345613	0.08672156
NUF2	2.99616834	-1.73616451	2.6174821	-0.66329566	0.50714122	0.81766998	-0.29487109	0.087421945
LRRK2	3.27496685	-1.50819618	2.27298243	-0.66353183	0.50699	0.81766998	-0.2950006	0.087421945
PBX4	49.383233	-1.5484319	2.33008104	-0.66453993	0.5063448	0.81721695	-0.29555365	0.087662632
ZMYM6	5.73456808	-0.86265078	1.29636714	-0.66543709	0.50577097	0.81660733	-0.2960461	0.087986724
SF3B1	474.335861	-1.04037487	1.55317013	-0.66983961	0.50296004	0.81427901	-0.29846652	0.08922676
CAPN1	722.400609	-0.80627513	1.2022345	-0.67064715	0.50244534	0.81376212	-0.29891118	0.089502532
SIK3	171.15613	-1.35482837	2.0037541	-0.67614503	0.49894857	0.81125414	-0.30194422	0.090843076
FILIP1L	5.38192881	-1.71164341	2.52329844	-0.6783357	0.49755887	0.80994337	-0.30315553	0.091545347
SH3BGRL2	26.0284967	-1.70564095	2.51356318	-0.67857493	0.49740723	0.80994337	-0.3032879	0.091545347
NAPG	3.54524645	-1.71753102	2.52700146	-0.67967156	0.49671245	0.80983189	-0.30389495	0.091605126
PHKB	636.595973	-0.73749358	1.08344992	-0.68069005	0.49606764	0.80973173	-0.30445911	0.091658843
HHEX	290.001886	-1.53710934	2.25566202	-0.68144488	0.49559003	0.80961116	-0.30487744	0.091723516
CHD4	290.786157	-1.24711101	1.82352651	-0.68390067	0.49403789	0.80768503	-0.30623974	0.092757967

CMTM5	43.5630039	-1.04262344	1.5202732	-0.6858132	0.49283091	0.80634495	-0.30730206	0.093479129
RAP1B	49.3718576	-1.13905126	1.65617035	-0.68776214	0.49160258	0.80551302	-0.30838584	0.093927437
MAVS	177.405286	-1.19914047	1.74239623	-0.68821342	0.49131839	0.80545268	-0.30863698	0.093959971
CAP1	51.7698614	-1.19906259	1.74203275	-0.68831231	0.49125613	0.80545268	-0.30869202	0.093959971
AP1B1	222.974884	-1.40087837	2.0305019	-0.68991729	0.4902462	0.80496261	-0.30958576	0.094224291
SLC50A1	52.2141841	-1.46255847	2.11965381	-0.68999875	0.49019498	0.80496261	-0.30963114	0.094224291
BANP	5.62281708	-1.36056031	1.96153787	-0.69361919	0.48792105	0.80241045	-0.31165044	0.095603422
MT.ND2	406.967893	-0.72970277	1.0494655	-0.69530896	0.4868617	0.80130123	-0.31259439	0.096204191
DNAH14	3.04040449	-1.1491892	1.65034142	-0.69633422	0.48621955	0.80056078	-0.31316758	0.096605692
VDAC3	28.7799068	-1.65296732	2.37148872	-0.69701674	0.48579232	0.80056078	-0.31354935	0.096605692
C9orf16	38.853096	-1.58968792	2.27745524	-0.69801061	0.48517056	0.80056078	-0.31410556	0.096605692
HMGB1	145.733788	-0.93966039	1.34585935	-0.69818617	0.48506077	0.80056078	-0.31420385	0.096605692
WRNIP1	97.6131426	-1.45686035	2.08628058	-0.69830509	0.48498642	0.80056078	-0.31427042	0.096605692
PF4V1	12.1977565	-2.04243207	2.90311952	-0.70353014	0.48172543	0.79884696	-0.31720042	0.097536416
LGALS8	174.65237	-1.26675249	1.80037059	-0.70360652	0.48167785	0.79884696	-0.31724333	0.097536416
KAT7	32.442831	-1.57198831	2.22766269	-0.70566712	0.48039517	0.79727601	-0.31840136	0.098391304
MFAP3L	145.790743	-1.58124959	2.2309739	-0.70877099	0.4784666	0.79490853	-0.32014837	0.099682842
TRAPPC3	78.9611594	-1.79970101	2.53903481	-0.70881305	0.4784405	0.79490853	-0.32017207	0.099682842
CFLAR	301.756887	-0.94051272	1.32486107	-0.70989535	0.47776903	0.79482924	-0.320782	0.099726165
MARK3	41.3109358	-1.31990732	1.85214308	-0.71263788	0.47606986	0.79294196	-0.32232931	0.100758598
SAP18	31.249319	-1.7023462	2.38441625	-0.71394674	0.47526011	0.79264224	-0.32306863	0.100922785
INF2	983.467445	-1.14252376	1.59893809	-0.71455159	0.47488616	0.79255551	-0.32341048	0.100970312
CD99	27.7769654	-1.23867869	1.7277709	-0.716923	0.47342161	0.79087915	-0.32475192	0.101889873
ANKRD49	12.7207562	-1.67957468	2.34252451	-0.71699343	0.47337815	0.79087915	-0.32479179	0.101889873
PIAS4	60.6206002	-1.59395314	2.22056926	-0.71781285	0.47287269	0.79087915	-0.32525577	0.101889873
PXK	3.22754711	-1.89485958	2.63723461	-0.71850247	0.47244753	0.79087915	-0.32564642	0.101889873
DCTN2	457.919129	-1.28009145	1.78087892	-0.71879757	0.47226565	0.79087915	-0.32581364	0.101889873
GNL3	6.35405972	-2.10553842	2.91383179	-0.72260122	0.46992492	0.79087915	-0.32797153	0.101889873
TP53BP1	39.5042691	-1.73578072	2.39881669	-0.72359873	0.46931212	0.79054032	-0.32853823	0.102075976
FRMD8	57.0572815	-1.37545098	1.89102677	-0.7273567	0.46700748	0.78825259	-0.33067617	0.103334596
C1orf116	33.683765	-1.72049642	2.36499796	-0.72748326	0.46692997	0.78825259	-0.33074825	0.103334596
PGD	114.528176	-1.85845388	2.554545	-0.72750877	0.46691435	0.78825259	-0.33076278	0.103334596
CDK2	4.1971472	-1.84657368	2.53782619	-0.72762023	0.46684609	0.78825259	-0.33082627	0.103334596
BCAS3	3.57685689	-1.36936927	1.87548306	-0.73014217	0.46530329	0.78697112	-0.33226388	0.104041208
PDE2A	323.189332	-1.23896783	1.69341323	-0.7316394	0.4643887	0.78606552	-0.33311836	0.104541251
RBMS3	51.817566	-1.57311986	2.14620377	-0.73297786	0.46357193	0.78595827	-0.33388287	0.104600511
RBM5	722.905493	-0.94541792	1.28972447	-0.73303867	0.46353484	0.78595827	-0.33391762	0.104600511
KIF2A	261.466413	-0.94583929	1.28666713	-0.735108	0.46227372	0.7850358	-0.33510079	0.105110536
LDB1	18.5814592	-1.72258853	2.34310588	-0.73517315	0.46223405	0.7850358	-0.33513807	0.105110536
ARRDC3	250.158761	-1.83822748	2.49830381	-0.73579021	0.46185838	0.78497073	-0.33549117	0.105146536
MEF2C	9.00223714	-1.5882465	2.15803534	-0.73596872	0.46174973	0.78497073	-0.33559335	0.105146536
KPNB1	10.8754928	-1.29056194	1.75200711	-0.73661912	0.461354	0.78475411	-0.33596571	0.1052664
RAB2A	6.75066852	-2.14611462	2.91311971	-0.73670664	0.46130077	0.78475411	-0.33601582	0.1052664
MCFD2	3.26887007	-2.15973024	2.93075013	-0.73692063	0.46117062	0.78475411	-0.33613837	0.1052664
KDM3B	164.735278	-1.75848846	2.38476973	-0.73738292	0.46088952	0.78475411	-0.33640317	0.1052664
ZNF44	22.015761	-1.29314902	1.7528299	-0.73774929	0.46066681	0.78475411	-0.33661308	0.1052664
TRIM13	240.201196	-1.12638015	1.52421036	-0.73899258	0.4599115	0.78474898	-0.33732573	0.105269238
PSPC1	3.9498072	-1.59943683	2.15678194	-0.74158486	0.4583389	0.783466	-0.33881328	0.105979845
ARID3B	70.8063034	-1.43188263	1.92832265	-0.74255345	0.45775208	0.78310586	-0.33936967	0.106179525
NASP	113.561274	-1.31149834	1.76619262	-0.7425568	0.45775005	0.78310586	-0.3393716	0.106179525
NUP54	7.60697906	-1.75080508	2.35352876	-0.74390639	0.45693311	0.78299155	-0.34014737	0.106242924
NFE2L2	33.9018501	-1.59611161	2.1361754	-0.74718191	0.45495376	0.78155276	-0.34203274	0.107041701
ZNF555	11.9709925	-1.58897333	2.1189884	-0.74987354	0.45333087	0.78002882	-0.34358471	0.10788935
LY6G6F	158.667649	-1.39116252	1.85506037	-0.74992844	0.45329781	0.78002882	-0.34361638	0.10788935
RECQL5	4.54685973	-1.889615	2.51952914	-0.74998736	0.45326232	0.78002882	-0.34365038	0.10788935
TPM3	51.1202713	-1.27739505	1.70273289	-0.75020284	0.45313255	0.78002882	-0.34377474	0.10788935
SLC35D1	7.59858886	-1.79773109	2.39578261	-0.75037321	0.45302996	0.78002882	-0.34387307	0.10788935
HIVEP1	21.684092	-1.82422323	2.42243405	-0.75305383	0.45141757	0.77898995	-0.34542154	0.108468144
C16orf72	65.3224365	-1.78641426	2.3670726	-0.75469348	0.45043292	0.77793557	-0.34636988	0.109056369
UHRF1BP1	312.906566	-1.44130232	1.9034259	-0.75721483	0.44892117	0.77681423	-0.34782992	0.109682828
EIF4H	78.5570386	-1.78895164	2.36000566	-0.75802854	0.4484339	0.77681423	-0.34830156	0.109682828
RUFY1	1429.6933	-0.93403129	1.23073217	-0.75892328	0.44789845	0.77681423	-0.34882044	0.109682828

PLEKHO2	33.4354934	-1.79846286	2.36782062	-0.75954354	0.44752748	0.77678384	-0.34918029	0.10969982
ZNF277	3.40552634	-2.22670702	2.93049809	-0.7598391	0.44735077	0.77678384	-0.34935181	0.10969982
RGS18	247.365229	-1.2093275	1.59074809	-0.76022565	0.44711972	0.77678384	-0.34957618	0.10969982
BBC3	50.0638584	-0.90573348	1.19079111	-0.76061492	0.44688711	0.77678384	-0.34980218	0.10969982
HTT	760.916857	-1.02162355	1.34176708	-0.76140156	0.44641725	0.77678384	-0.35025903	0.10969982
CLASRP	5.83794169	-1.69130966	2.21302292	-0.76425311	0.44471642	0.77481137	-0.35191684	0.110804015
CALM1	169.782582	-1.31947198	1.71974253	-0.76724973	0.44293305	0.772998	-0.35366191	0.111821629
RNF34	11.2485803	-1.88698767	2.45788917	-0.76772692	0.44264944	0.77282695	-0.35394008	0.111917741
EGFL8	23.5971111	-2.21857276	2.88808974	-0.76818	0.44238026	0.77268095	-0.35420426	0.111999794
GNG11	64.2308168	-1.30654911	1.70066281	-0.76825877	0.44233347	0.77268095	-0.3542502	0.111999794
CBLB	4.28951457	-1.64250906	2.13783888	-0.76830348	0.44230691	0.77268095	-0.35427628	0.111999794
NDUFA5	11.053302	-1.39413425	1.81401663	-0.76853444	0.44216974	0.77268095	-0.35441098	0.111999794
TBXAS1	235.245899	-1.15230878	1.49747963	-0.7694988	0.44159726	0.77268095	-0.35497363	0.111999794
YIF1B	229.690293	-1.1479943	1.49078535	-0.77006009	0.44126425	0.77268095	-0.35530126	0.111999794
UBA1	77.7303405	-1.31377014	1.69939118	-0.77308283	0.43947336	0.77140115	-0.35706744	0.11271972
KCTD20	29.8170844	-1.56850144	2.02472482	-0.77467388	0.43853239	0.77048475	-0.35799833	0.113235951
RSF1	54.6461979	-1.69807903	2.17474425	-0.7808178	0.43490966	0.76541159	-0.36160094	0.116104963
JUND	13.7027707	-1.3558459	1.73595034	-0.78103957	0.43477922	0.76541159	-0.36173122	0.116104963
EPS15	39.4194807	-1.43910536	1.84009262	-0.78208311	0.43416573	0.76513083	-0.36234446	0.1162643
MT.ND5	259.812323	-1.09570342	1.40026182	-0.78249896	0.43392139	0.76513083	-0.36258894	0.1162643
TAOK3	184.900286	-1.36719231	1.7420794	-0.78480482	0.43256802	0.76437796	-0.3639456	0.116691844
MBTD1	27.787579	-1.32644357	1.68994359	-0.78490405	0.43250983	0.76437796	-0.36400402	0.116691844
DISP2	3.55569491	-1.56367159	1.97999897	-0.78973353	0.4296834	0.7633113	-0.36685142	0.117298307
U2SURP	4.53986576	-1.51788623	1.92067539	-0.79028775	0.42935974	0.76306187	-0.36717868	0.117440249
WDR1	262.110333	-0.99038634	1.25314305	-0.79032186	0.42933983	0.76306187	-0.36719883	0.117440249
KANSL3	44.5421405	-1.831584	2.3163407	-0.79072306	0.42910562	0.76306187	-0.3674358	0.117440249
MEF2A	46.6681616	-1.64411164	2.07829981	-0.79108492	0.42889444	0.76306187	-0.36764959	0.117440249
VEZT	10.5498382	-1.76788485	2.22940249	-0.79298595	0.42778601	0.76286978	-0.36877342	0.117549587
CBX6	5.00659901	-0.86632156	1.08984778	-0.79490144	0.42667084	0.76185993	-0.36990704	0.118124865
HLA.E	596.592046	-0.94018104	1.18276117	-0.79490354	0.42666962	0.76185993	-0.36990828	0.118124865
ORAI2	54.6220993	-1.18012487	1.48135563	-0.79665197	0.42565319	0.76134874	-0.37094411	0.118416365
OSGEP	16.7857589	-2.11827035	2.65787702	-0.79697831	0.42546363	0.76134874	-0.37113756	0.118416365
AKAP17A	17.3229077	-2.03615061	2.54004465	-0.80162001	0.4227728	0.75815071	-0.37389296	0.120244456
TXNIP	145.693828	-1.43813063	1.79231408	-0.80238762	0.42232877	0.75815071	-0.37434933	0.120244456
PPP2R2D	92.7467584	-1.0799007	1.33676689	-0.80784519	0.4191797	0.75447922	-0.37759975	0.12235272
SNW1	18.8954776	-2.29692338	2.84292131	-0.80794476	0.41912238	0.75447922	-0.37765915	0.12235272
GRK4	3.66715731	-1.56001793	1.92775865	-0.80923923	0.41837755	0.75447922	-0.37843163	0.12235272
PDLIM7	832.57413	-0.83065993	1.02265924	-0.81225485	0.41664541	0.7523467	-0.3802334	0.123581979
ZNF274	4.96343626	-2.34289501	2.88209726	-0.81291324	0.41626781	0.75205759	-0.38062718	0.123748902
VPS37A	16.1948411	-1.8575163	2.28063391	-0.81447368	0.41537364	0.75135354	-0.38156107	0.124155664
FOXO1	3.91733502	-2.20537279	2.70364439	-0.81570372	0.41466961	0.75105883	-0.38229779	0.124326043
TAL1	611.788844	-1.01660618	1.24025606	-0.81967443	0.41240173	0.74923246	-0.38467952	0.125383415
TCF20	3.04708462	-1.90054672	2.31429122	-0.82122194	0.41151986	0.74828328	-0.3856092	0.12593396
CYLD	172.722739	-1.93524398	2.3539494	-0.82212641	0.41100495	0.74800027	-0.38615294	0.126098243
TNKS	27.4815912	-2.07076096	2.51531566	-0.82326087	0.41035966	0.74747928	-0.38683534	0.126400844
ATP8A1	90.2415527	-1.71650345	2.06924844	-0.8295299	0.40680462	0.74262799	-0.39061412	0.129228684
C6orf136	32.0448123	-2.4131384	2.89545485	-0.83342291	0.40460626	0.74087059	-0.3929674	0.130257647
GTPBP2	1643.17123	-1.0926019	1.31078285	-0.83354913	0.4045351	0.74087059	-0.39304379	0.130257647
USP42	304.018554	-1.3211723	1.58432057	-0.83390466	0.40433471	0.74087059	-0.39325897	0.130257647
GP6	159.119628	-0.98232938	1.17625243	-0.83513484	0.40364179	0.74075118	-0.39400388	0.13032765
MED15	60.4620886	-1.57701117	1.88172182	-0.83806817	0.40199242	0.73846165	-0.39578214	0.131672056
RREB1	114.434284	-1.67491089	1.99424218	-0.83987336	0.40097939	0.73749037	-0.39687795	0.132243647
UBR5	155.510708	-1.43422796	1.70330071	-0.84202863	0.39977193	0.73624516	-0.3981877	0.132977546
MATR3	5.92891943	-1.93677874	2.29557797	-0.84369983	0.39883717	0.73536075	-0.39920438	0.133499554
GGA3	51.0865198	-2.327571	2.7581499	-0.84388851	0.39873171	0.73536075	-0.39931922	0.133499554
TSC22D1	204.122312	-1.25437604	1.48494289	-0.84473016	0.39826152	0.73536075	-0.39983166	0.133499554
ABHD11	67.2964407	-1.6584775	1.95965478	-0.84631105	0.39737924	0.73484017	-0.40079482	0.133807108
ZNF507	4.30596489	-1.94416101	2.2966212	-0.84653099	0.3972566	0.73484017	-0.40092888	0.133807108
FURIN	206.154774	-1.39427405	1.63723122	-0.85160486	0.39443344	0.73256952	-0.40402627	0.135151156
SFI1	556.968217	-1.18646353	1.39239334	-0.85210371	0.39415653	0.73238189	-0.40433127	0.135262405
ZFR	21.8734401	-2.1300348	2.4965078	-0.85320575	0.39354522	0.73157245	-0.40500536	0.135742659
PDE4D	84.7838154	-1.59357627	1.86127446	-0.85617479	0.3919011	0.7291672	-0.40682351	0.137172875

SERTAD3	16.8961506	-2.4137396	2.81664894	-0.85695436	0.39147011	0.72872268	-0.4073014	0.137437715
SPTB	277.566605	-1.23372793	1.4387689	-0.8574886	0.39117491	0.72872268	-0.40762901	0.137437715
FSIP2	4.05177988	-2.02821148	2.35863094	-0.85991049	0.38983839	0.7278957	-0.4091154	0.137930848
R3HDM4	152.520563	-1.05619428	1.22369005	-0.86312239	0.38807018	0.72560585	-0.41108973	0.139299225
ABCC1	170.388902	-1.3439487	1.5473535	-0.86854665	0.38509514	0.72155034	-0.41443196	0.141733363
TNIP1	12.691867	-1.65692839	1.90465682	-0.8699354	0.38433571	0.72056456	-0.41528926	0.1423271
SH3D19	3.25278123	-2.1478228	2.45335481	-0.87546359	0.38132172	0.71685039	-0.41870846	0.144571472
PSMA1	4.52453287	-2.56432676	2.92822778	-0.87572653	0.38117872	0.71685039	-0.41887135	0.144571472
ABCA1	25.1441486	-1.90137773	2.16419097	-0.87856283	0.37963836	0.71479391	-0.42062991	0.145819156
PGRMC1	192.727396	-2.10521076	2.38868811	-0.88132509	0.37814189	0.71300883	-0.42234521	0.146905089
MKRN1	152.572447	-1.4437021	1.63414551	-0.88345994	0.37698782	0.71129822	-0.42367269	0.147948281
CAMKK1	5.70685889	-2.2954233	2.59366663	-0.88501093	0.37615074	0.71098124	-0.42463808	0.148141857
PEAR1	1486.90158	-1.06462404	1.20266975	-0.88521728	0.37603946	0.71098124	-0.42476658	0.148141857
TNFAIP8L1	64.6887788	-1.35708775	1.53165156	-0.88602904	0.37560188	0.71091192	-0.42527224	0.148184201
CDK11A	19.893622	-2.20783947	2.48651592	-0.88792493	0.37458113	0.71027133	-0.4264541	0.148575714
WBP4	2.9990157	-1.88637003	2.11918961	-0.89013745	0.37339209	0.7086621	-0.42783489	0.14956079
CPSF2	5.44211974	-1.70858186	1.91143471	-0.89387404	0.37138929	0.70582611	-0.43017062	0.15130228
BRD9	148.400451	-1.71704907	1.91809467	-0.89518473	0.37068834	0.70481565	-0.43099107	0.151924463
TSPAN32	253.228271	-1.23381704	1.3746264	-0.89756536	0.36941731	0.70271982	-0.43248275	0.153217794
MKNK1	80.3500656	-1.69472017	1.88796007	-0.89764619	0.3693742	0.70271982	-0.43253344	0.153217794
APPL1	33.0292364	-2.48025764	2.75237611	-0.90113325	0.36751747	0.70070639	-0.43472201	0.15446392
SCAMP2	80.874386	-2.13958129	2.35654358	-0.90793198	0.36391416	0.69706246	-0.43900105	0.156728303
PTPN18	126.356321	-1.56666984	1.72401245	-0.90873465	0.36349021	0.69691366	-0.43950728	0.156821025
CAV2	2.80584118	-2.07888799	2.28012365	-0.91174353	0.36190374	0.69573739	-0.44140693	0.157554658
RNF170	5.67697081	-2.62811112	2.88237952	-0.91178525	0.36188178	0.69573739	-0.44143328	0.157554658
FOS	223.799357	-1.94983743	2.13209237	-0.91451827	0.36044458	0.69437058	-0.4431615	0.158408691
ANKRD28	62.2859367	-1.65168015	1.80069208	-0.91724741	0.35901301	0.6938303	-0.44488981	0.158746738
RP11.382A20.	7.57061657	-2.22260203	2.41090836	-0.92189403	0.35658385	0.69116528	-0.44783833	0.160418087
KLHL7	4.37610312	-1.31795703	1.42838714	-0.92268895	0.35616932	0.69116528	-0.44834349	0.160418087
ALOX12	207.08887	-1.50731013	1.62344562	-0.92846358	0.35316715	0.68847753	-0.4520197	0.162110226
ENY2	34.2292989	-2.13720982	2.29969031	-0.92934679	0.35270939	0.68820484	-0.45258298	0.162282276
HCFC2	9.27313414	-2.56414866	2.75424768	-0.93097969	0.35186407	0.68820484	-0.45362508	0.162282276
HIST1H1C	465.984521	-1.52645992	1.63373287	-0.93433875	0.35012918	0.68641145	-0.45577169	0.163415483
GRPEL2	44.8686176	-2.6816681	2.86974137	-0.93446334	0.35006494	0.68641145	-0.45585138	0.163415483
ST3GAL5	12.2803044	-2.2157431	2.36073274	-0.93858278	0.347945	0.6847084	-0.4584894	0.164494347
GAS2L1	51.1475644	-1.50488921	1.60224826	-0.93923597	0.3476096	0.6847084	-0.45890823	0.164494347
UBE2J1	18.7502995	-2.09304047	2.22695736	-0.93986554	0.34728654	0.68470698	-0.45931205	0.164495245
UTY	109.232938	-1.94452641	2.06475145	-0.94177263	0.34630906	0.68337558	-0.46053615	0.165340544
DENND5A	4.33332793	-2.71516816	2.8762917	-0.9439822	0.34517875	0.68196324	-0.46195595	0.166239035
ECE1	208.589746	-1.4193661	1.49698215	-0.94815165	0.34305226	0.6795764	-0.46463972	0.16776171
USP38	6.77375738	-2.27980288	2.39848777	-0.95051679	0.34184973	0.67846037	-0.46616476	0.168475513
LARP4	55.8524257	-2.1213493	2.23176643	-0.95052478	0.34184567	0.67846037	-0.46616992	0.168475513
MYO9B	195.119637	-1.66487973	1.74877122	-0.95202832	0.34108262	0.67846037	-0.46714041	0.168475513
YWHAH	86.4290198	-2.07321919	2.15956464	-0.9600172	0.33704656	0.67368063	-0.4723101	0.171545938
AP1S2	4.80601817	-2.26352246	2.35737756	-0.96018665	0.33696128	0.67368063	-0.47241999	0.171545938
FAM127B	3.17899997	-2.8388201	2.94347039	-0.96444663	0.33482205	0.67062562	-0.47518595	0.173519858
G3BP2	10.405229	-1.8747005	1.9372852	-0.96769464	0.3331969	0.66851669	-0.47729905	0.174887745
PDGFRA	4.23806701	-2.05806897	2.11889205	-0.97129486	0.33140147	0.66644576	-0.47964557	0.176235189
PYGB	3.63052625	-2.25952616	2.32610439	-0.9713778	0.33136018	0.66644576	-0.47969968	0.176235189
RAB37	9.3538793	-1.71801295	1.76056928	-0.97582808	0.32914967	0.6643622	-0.48260658	0.177595088
PYGL	54.0976065	-2.21559634	2.26948223	-0.97625631	0.32893747	0.66425587	-0.48288666	0.177664599
GOLGA1	20.5270703	-2.43759015	2.49419035	-0.97730719	0.3284171	0.66384893	-0.48357424	0.177930742
TTLL7	122.515062	-1.89458115	1.93382542	-0.9797064	0.32723106	0.66241621	-0.48514548	0.178869047
MFSD11	49.6231112	-2.23318742	2.27720841	-0.98066888	0.32675606	0.66177637	-0.48577636	0.179288745
CLTC	3.04846614	-2.23967099	2.28327735	-0.98090186	0.32664114	0.66177637	-0.48592911	0.179288745
ST3GAL6	28.9660712	-2.16106567	2.196781	-0.98374197	0.3252424	0.66063871	-0.48779284	0.180035985
ABCB6	13.1789033	-2.0415016	2.07172426	-0.98541183	0.32442182	0.6594522	-0.48888994	0.180816678
TMEM40	1061.58549	-1.26215556	1.27537899	-0.98963176	0.32235414	0.65701549	-0.49166675	0.182424393
SERPINB6	9.48136023	-2.42754775	2.44738088	-0.99189618	0.32124817	0.65540293	-0.49315933	0.183491619
ERI1	4.06435543	-1.22709504	1.23280539	-0.995368	0.31955731	0.65484087	-0.49545124	0.18386422
IRX3	6.81717592	-2.34044951	2.34951052	-0.99614345	0.31918045	0.65439064	-0.49596372	0.184162919
SLC22A23	40.9757176	-2.03765621	2.04322469	-0.99727466	0.31863121	0.65358639	-0.49671169	0.184697003

MKL1	5.67047279	-2.3212923	2.32696641	-0.99756158	0.318492	0.65358639	-0.49690147	0.184697003
PPP2R5A	4.93463846	-2.48543738	2.4897361	-0.99827342	0.31814679	0.65323631	-0.49737245	0.184929683
C11orf21	184.976095	-2.32360441	2.3229396	-1.0002862	0.31717203	0.65187775	-0.49870512	0.185833845
CMIP	874.583789	-1.22235754	1.22177828	-1.00047411	0.31708112	0.65187775	-0.49882962	0.185833845
TECPR2	316.594112	-1.39078047	1.38936442	-1.00101921	0.31681752	0.65179257	-0.49919081	0.185890594
FKBP15	15.6422215	-2.36208938	2.35178823	-1.00438014	0.31519542	0.65038341	-0.5014201	0.186830543
DDX41	39.7663608	-2.93070576	2.89603952	-1.01197023	0.31155229	0.64446286	-0.50646905	0.190802107
MAP1A	47.27666	-2.28670425	2.2580133	-1.01270628	0.31120048	0.64409807	-0.50695975	0.191048003
IP6K2	89.9748026	-2.3659225	2.32927042	-1.01573543	0.30975539	0.64202163	-0.50898113	0.192450343
CXCL3	80.7996151	-1.73557029	1.70806672	-1.01610216	0.30958074	0.64202163	-0.50922607	0.192450343
SSX2IP	105.436483	-2.55977288	2.51127387	-1.01931251	0.30805462	0.64035477	-0.51137227	0.193579352
CD84	153.741659	-2.20447007	2.15357797	-1.02363142	0.3060094	0.63679317	-0.51426523	0.196001601
HLA.A	237.174604	-1.23518298	1.20394542	-1.02594599	0.30491704	0.63579097	-0.5158183	0.196685647
PDGFA	80.2521906	-1.25232255	1.2163945	-1.02953651	0.30322763	0.63416689	-0.51823123	0.197796439
NECAB3	97.4063552	-2.44368376	2.35734475	-1.03662553	0.29991041	0.63032898	-0.52300846	0.200432729
ARPC1B	126.466156	-1.98032783	1.90614442	-1.03891804	0.29884285	0.62847558	-0.52455712	0.201711594
FAM177A1	9.1443419	-2.68982477	2.58212758	-1.0417087	0.29754675	0.62733402	-0.52644478	0.202501163
ACVR1B	63.0910672	-2.11917369	2.03040431	-1.04372005	0.29661493	0.62671293	-0.52780699	0.202931344
SLC43A2	3.87514143	-1.30640677	1.25030143	-1.04487345	0.29608146	0.62614688	-0.52858878	0.203323779
CHMP6	177.128694	-2.50850402	2.39946313	-1.04544387	0.29581787	0.62590732	-0.5289756	0.203489969
TTYH3	47.1260458	-2.24852432	2.14740535	-1.0470889	0.29505858	0.62510342	-0.53009176	0.204048128
EVL	19.564797	-2.58519631	2.45521631	-1.05294034	0.29236833	0.62113354	-0.53406967	0.206815016
CDC42SE2	147.954389	-2.22047394	2.10735205	-1.05367963	0.29202962	0.62113354	-0.5345731	0.206815016
CNPY3	203.983815	-2.02864461	1.91551424	-1.05906005	0.28957243	0.61771496	-0.53824279	0.209211879
PRRT3	49.0931073	-2.07668882	1.95652133	-1.06141895	0.28849955	0.61637358	-0.53985486	0.210155983
VPS13D	5.47775852	-2.36090869	2.21830473	-1.06428511	0.28719956	0.61391118	-0.54181623	0.21189446
CARD19	163.477553	-1.88594228	1.76669042	-1.06750015	0.28574605	0.61174629	-0.54401977	0.213428653
TBCK	10.8279352	-1.94097108	1.81590389	-1.06887324	0.28512679	0.61104887	-0.54496197	0.213924054
LPAR5	5.87345671	-1.90869935	1.78108583	-1.07164929	0.28387759	0.61071548	-0.54686889	0.214161073
FBRS	18.960166	-1.95277646	1.81934721	-1.07333908	0.28311901	0.61020059	-0.54803097	0.214527374
AHCTF1	15.8909278	-2.48264195	2.31227167	-1.07368091	0.28296572	0.61018605	-0.54826617	0.214537726
STRIP2	207.206218	-1.94226777	1.79839503	-1.08000063	0.2801419	0.60755774	-0.55262193	0.216412439
PTTG1IP	319.023188	-1.57440704	1.45420978	-1.08265469	0.27896172	0.60594502	-0.55445539	0.217566778
CUBN	5.40540397	-2.17906994	2.00344544	-1.08766124	0.27674469	0.60238624	-0.55792071	0.220124957
ADD3	281.816202	-1.5451144	1.4159769	-1.09120029	0.27518476	0.60056045	-0.56037562	0.221443272
ZMIZ1	35.0196699	-2.03877424	1.86810892	-1.09135726	0.27511571	0.60056045	-0.56048461	0.221443272
TOMM70	5.91493092	-2.69001238	2.46351633	-1.09194014	0.27485941	0.60056045	-0.56088939	0.221443272
EXOC3	6.40948786	-2.21551889	2.0211421	-1.09617176	0.27300362	0.59865902	-0.5638316	0.222820472
PML	20.7319217	-2.32056524	2.11678634	-1.09626805	0.27296149	0.59865902	-0.56389863	0.222820472
PFDN2	7.1321643	-3.22256654	2.92597118	-1.10136647	0.27073719	0.59585078	-0.56745208	0.224862488
SLA2	30.8683982	-2.18799701	1.97680326	-1.106836	0.26836484	0.59329083	-0.57127438	0.226732366
FAM214A	67.8882191	-2.17430505	1.96084106	-1.10886348	0.26748908	0.593199	-0.57269394	0.226799592
OPHN1	31.4802465	-2.7961804	2.52155977	-1.10890903	0.26746943	0.593199	-0.57272585	0.226799592
DNTTIP1	40.9756826	-2.69551534	2.41789257	-1.11482014	0.2649275	0.59023976	-0.57687296	0.228971536
KCNAB2	8.41562519	-2.74905807	2.4657629	-1.11489149	0.26489692	0.59023976	-0.57692308	0.228971536
F11R	11.1549921	-2.15672128	1.93412428	-1.11508929	0.26481216	0.59023976	-0.57706208	0.228971536
CHST12	78.5484557	-2.24712423	2.01006753	-1.11793469	0.26359489	0.58916216	-0.57906302	0.229765154
GP9	577.914609	-1.42827948	1.27498324	-1.12023393	0.26261409	0.5880053	-0.58068198	0.23061876
ENKUR	19.8263797	-2.45030599	2.18478027	-1.12153429	0.26206051	0.58762517	-0.58159843	0.230899609
TOP2B	40.1719131	-2.10867487	1.87848423	-1.12254063	0.26163264	0.58698185	-0.58230807	0.231375325
ADAM32	5.41287215	-2.24507338	1.99928174	-1.12293997	0.26146299	0.58691746	-0.58258978	0.231422972
ZRANB1	4.90505265	-3.2458808	2.88854145	-1.12370927	0.26113638	0.58691746	-0.58313261	0.231422972
C1orf112	50.5611348	-2.59183463	2.30612924	-1.12388958	0.26105987	0.58691746	-0.58325988	0.231422972
BAZ2B	25.7501607	-2.49386806	2.21696186	-1.12490346	0.26062995	0.58691746	-0.58397568	0.231422972
LDLR	62.5246789	-2.44973787	2.17080961	-1.12849043	0.25911286	0.58510876	-0.58651104	0.2327634
ATXN1	4.45920765	-2.85000886	2.52445112	-1.12896179	0.25891395	0.58510876	-0.58684454	0.2327634
SLK	10.9218312	-2.45365256	2.14446292	-1.14418045	0.25254881	0.57475659	-0.59765468	0.240516037
BTN3A2	15.8390395	-2.1423802	1.87045931	-1.14537653	0.25205321	0.57465082	-0.59850776	0.24059597
GRB14	3.10213362	-3.39530474	2.95493855	-1.14902719	0.25054477	0.5738905	-0.60111466	0.241170963
NBPF11	8.00598071	-2.69826645	2.34539235	-1.15045419	0.24995685	0.57345473	-0.60213495	0.241500862
RANBP3	31.1779346	-2.54699884	2.2113942	-1.15176156	0.24941907	0.57307347	-0.60307035	0.241789695
MCF2L	6.943948	-2.0372697	1.76540151	-1.15399794	0.24850101	0.57200565	-0.60467183	0.24259968

ADSL	28.6498288	-2.89261778	2.50607169	-1.15424382	0.24840022	0.57200565	-0.60484802	0.24259968
TRAFD1	14.2877311	-2.46182452	2.13227966	-1.15455049	0.24827455	0.57200565	-0.60506779	0.24259968
HNRNPUL2	4.7261068	-3.35978046	2.90484647	-1.15661206	0.24743089	0.57175485	-0.60654608	0.242790144
SDE2	17.2187716	-3.05206342	2.63522249	-1.15818054	0.24679037	0.57059139	-0.6076718	0.243674788
ST6GAL1	189.405123	-2.03988337	1.7540379	-1.16296425	0.24484401	0.56735139	-0.61111052	0.246147876
GMFG	69.4858661	-1.90474726	1.63558787	-1.16456431	0.2441954	0.56616349	-0.61226253	0.247058137
PTMA	121.064053	-1.35559831	1.16034492	-1.16827185	0.24269713	0.56363125	-0.61493536	0.249004933
C12orf76	7.27325893	-2.80189817	2.38961838	-1.17252955	0.24098451	0.56059191	-0.61801087	0.251353176
EDC4	14.15399	-2.61846531	2.23001537	-1.1741916	0.24031829	0.55935459	-0.61921318	0.252312796
POLD4	37.5342053	-2.7087103	2.30340597	-1.17595871	0.23961138	0.55840241	-0.62049256	0.253052718
CLEC1B	192.839008	-2.51909868	2.13349916	-1.18073572	0.23770773	0.5557636	-0.6239567	0.255109902
RNF122	5.5108632	-2.72701651	2.30339964	-1.18390941	0.23644892	0.55457443	-0.62626266	0.256040155
KLF6	284.39344	-2.05330618	1.73308227	-1.18477133	0.23610787	0.55420134	-0.62688953	0.256332426
MROH1	16.844593	-3.12321692	2.62106659	-1.19158244	0.23342501	0.54976344	-0.63185261	0.259824148
MLLT11	7.2523825	-2.8286128	2.36724718	-1.19489542	0.23212789	0.54732761	-0.63427267	0.261752646
CRIM1	3.45435482	-2.75394623	2.29571616	-1.19960223	0.23029386	0.54423588	-0.63771764	0.264212826
ABTB1	60.3866136	-2.42747224	2.0133867	-1.20566617	0.22794623	0.54083653	-0.64216758	0.266933981
MAT2B	42.2668562	-2.97363038	2.45246838	-1.21250508	0.2253191	0.53582452	-0.64720199	0.270977419
MYLIP	46.0808725	-3.10240716	2.55260031	-1.21539089	0.22421704	0.53350843	-0.64933138	0.27285871
RALB	5.22588065	-3.31160164	2.7240523	-1.21568945	0.22410325	0.53350843	-0.64955185	0.27285871
MIEF1	3.65798492	-3.59932907	2.95278163	-1.21896216	0.22285855	0.53149085	-0.65197069	0.274504209
SRSF11	70.5926809	-2.80480503	2.29959828	-1.21969348	0.22258109	0.53113334	-0.65251173	0.274796438
ZC3H10	4.31943368	-3.07868776	2.52196102	-1.22075153	0.22218011	0.53078485	-0.65329482	0.275081484
FNBP4	49.9200655	-3.33398327	2.72810848	-1.22208603	0.22167509	0.52988237	-0.6542831	0.275820528
SYNE2	2.84028099	-2.27365639	1.85857594	-1.22333252	0.22120413	0.52906031	-0.65520677	0.276494819
TLK1	189.079232	-1.77994206	1.45392757	-1.22423022	0.22086539	0.52855374	-0.65587233	0.276910854
H3F3C	3.72400372	-2.65322671	2.15911878	-1.22884704	0.21912916	0.52500221	-0.65929982	0.279838872
NISCH	16.0063015	-2.56672882	2.08610958	-1.23039022	0.21855102	0.5239185	-0.66044717	0.280736268
LAPTM4B	37.9954087	-2.68543858	2.16749266	-1.23896086	0.21535999	0.51925825	-0.66683497	0.284616591
TSC22D3	20.4620488	-3.06259941	2.47145316	-1.23918975	0.21527524	0.51925825	-0.66700592	0.284616591
ANKRD54	4.35534232	-3.35792775	2.70935538	-1.23938254	0.21520387	0.51925825	-0.66714993	0.284616591
TGIF2	3.97352728	-3.63495724	2.92813412	-1.24139028	0.21446161	0.51919659	-0.66865045	0.284668171
MTHFD2L	31.0441728	-3.10985739	2.50493815	-1.24149069	0.21442454	0.51919659	-0.66872552	0.284668171
RNF103	16.3832396	-2.85868196	2.30253969	-1.24153428	0.21440844	0.51919659	-0.66875812	0.284668171
H3F3B	4279.24064	-1.76566532	1.42070857	-1.24280613	0.21393928	0.51919659	-0.66970947	0.284668171
MT.ND1	827.587733	-1.34045706	1.0754906	-1.246368	0.21262933	0.51716619	-0.67237684	0.286369879
ATP13A1	9.9586848	-3.5334135	2.82754334	-1.24964079	0.2114308	0.51552024	-0.67483176	0.287754279
RAB8A	9.65537393	-2.76715936	2.21058849	-1.25177498	0.21065187	0.51476196	-0.67643468	0.288393553
RBCK1	2.72512467	-3.71671984	2.9592673	-1.25595948	0.20913066	0.51285045	-0.67958229	0.290009257
TARDBP	298.848125	-1.9179288	1.52488278	-1.25775491	0.20848041	0.51213621	-0.68093475	0.290614518
CSTF3	4.27690955	-3.3349177	2.64717281	-1.25980355	0.20774024	0.51094528	-0.68247937	0.291625609
RBBP6	260.571922	-1.82715007	1.4490928	-1.26089238	0.20734762	0.51048562	-0.68330094	0.292016484
TLE4	23.049885	-2.77020535	2.19399982	-1.26262788	0.20672294	0.50994925	-0.68461132	0.292473042
SART3	39.3217217	-2.84775666	2.25249654	-1.26426683	0.20613427	0.5091003	-0.6858498	0.293196649
SELP	217.626526	-2.02509649	1.59069342	-1.27309038	0.20298597	0.50306347	-0.69253398	0.298377216
GTDC1	48.716801	-2.41378894	1.88451573	-1.2808537	0.20024506	0.49899487	-0.69843819	0.301903922
RGS9	129.876366	-2.55439258	1.99265807	-1.28190211	0.19987699	0.49850433	-0.69923721	0.302331069
CSMD2	4.81634602	-3.62056634	2.82328655	-1.28239421	0.19970439	0.49850433	-0.69961238	0.302331069
MEF2D	70.0306995	-2.1576534	1.66754144	-1.29391291	0.1956955	0.49059367	-0.70841915	0.309278062
DNAH7	6.80046091	-2.12597107	1.64193981	-1.29479233	0.19539188	0.49042301	-0.7090935	0.30942916
ZNF92	10.3199948	-3.62000536	2.78951588	-1.29771814	0.19438421	0.48907302	-0.71133902	0.310626298
ZNF250	2.92384694	-3.04560166	2.3355045	-1.30404444	0.19221843	0.48618361	-0.71620497	0.313199686
UBR1	6.91308321	-2.32763576	1.7816542	-1.30644643	0.19140079	0.48449415	-0.71805628	0.314711464
ARHGDIB	57.2295159	-2.95401763	2.24975656	-1.31303879	0.18916988	0.48030694	-0.72314802	0.318481143
NRGN	30.4700332	-1.75405021	1.3344334	-1.31445317	0.18869375	0.47939034	-0.72424249	0.319310726
HIST1H4B	16.6101503	-2.69035651	2.04611237	-1.31486255	0.1885561	0.4793331	-0.72455941	0.319362584
FAM193B	174.069342	-2.39030646	1.81027164	-1.32041314	0.18669712	0.47606051	-0.72886238	0.322337846
AMBRA1	3.15028725	-3.48147758	2.62503369	-1.32626015	0.18475354	0.47300437	-0.73340723	0.325134845
SFXN5	12.8321024	-3.42921824	2.58348398	-1.32736191	0.18438899	0.4724897	-0.73426501	0.325607655
DYNC1H1	60.8444136	-2.55161988	1.90253631	-1.3411675	0.17986607	0.46432879	-0.74505074	0.33317439
COPS8	20.1059777	-3.33979568	2.48981265	-1.34138433	0.1797957	0.46432879	-0.7452207	0.33317439
SELENOH	9.74071401	-3.89129974	2.89437645	-1.34443456	0.1788079	0.46274463	-0.74761329	0.334658612

PTCRA	288.252976	-1.92978621	1.43516196	-1.34464699	0.17873926	0.46274463	-0.74778004	0.334658612
F2RL3	217.121456	-2.38599104	1.77404169	-1.34494643	0.17864254	0.46274463	-0.74801513	0.334658612
SMTN	372.252077	-2.37030707	1.76198113	-1.34525111	0.17854415	0.46274463	-0.74825436	0.334658612
FLI1	192.325076	-2.08592778	1.54661992	-1.34870097	0.17743303	0.46033094	-0.75096552	0.336929836
BIRC2	8.03014089	-3.63970062	2.69546677	-1.35030439	0.17691836	0.45928184	-0.75222709	0.337920729
IK	3.86860727	-4.02300089	2.95862946	-1.35975151	0.17390857	0.45316351	-0.75967902	0.343745069
MAP9	8.5219033	-3.48858987	2.55756123	-1.36402986	0.17255817	0.44992625	-0.76306447	0.34685867
TFG	4.04025926	-3.87869547	2.83293016	-1.36914617	0.17095359	0.44770487	-0.76712178	0.349008182
ELL	14.8781644	-3.62473031	2.64581301	-1.36998733	0.17069086	0.44729813	-0.76778974	0.349402919
DNAJC16	3.52716792	-2.99469345	2.17608297	-1.37618532	0.16876426	0.44314141	-0.77271951	0.353457667
PITRM1	22.8117819	-3.88826163	2.82165111	-1.37800935	0.1682004	0.4426666	-0.77417297	0.35392324
CD151	6.8836505	-3.94600189	2.86340428	-1.3780806	0.1681784	0.4426666	-0.77422977	0.35392324
TACC3	513.977504	-1.78931639	1.29702991	-1.37954905	0.16772553	0.44231103	-0.77540082	0.354272228
RGS3	165.875956	-2.72215864	1.97317043	-1.37958617	0.1677141	0.44231103	-0.77543043	0.354272228
MGAT4B	16.5611291	-3.06753016	2.22324334	-1.37975457	0.16766223	0.44231103	-0.77556477	0.354272228
EGF	291.787761	-2.25647979	1.6293566	-1.38489008	0.16608608	0.4398008	-0.77966676	0.356743984
WDR36	17.2824525	-2.51999645	1.81638025	-1.38737274	0.16532813	0.43888319	-0.78165324	0.357651051
SASS6	24.2617926	-3.21852102	2.31536106	-1.39007305	0.16450669	0.43714478	-0.78381642	0.359374699
DAAM1	240.345892	-1.87873567	1.35135212	-1.3902636	0.16444885	0.43714478	-0.78396917	0.359374699
PTGER2	12.7257661	-3.13104984	2.24871239	-1.39237452	0.16380902	0.43640483	-0.7856622	0.360110456
TXNDC16	153.825741	-3.3614286	2.4127546	-1.39319125	0.16356196	0.43602562	-0.78631768	0.360487991
XPC	10.4657895	-3.83788798	2.74939135	-1.39590458	0.16274323	0.43495687	-0.78849707	0.361553801
MXD1	449.50041	-2.50951076	1.7967336	-1.3967072	0.16250164	0.43486942	-0.78914226	0.361641129
CNNM4	18.3993066	-3.9292798	2.81198138	-1.39733493	0.16231288	0.43464361	-0.78964702	0.361866697
TMEM64	124.96036	-2.69779984	1.93055094	-1.39742484	0.16228585	0.43464361	-0.78971933	0.361866697
DHX30	2.8083056	-4.14535803	2.96087118	-1.40004674	0.16149932	0.43358039	-0.79182929	0.362930369
IER2	182.543636	-2.53510074	1.80817397	-1.40202258	0.1609085	0.43311119	-0.793421	0.363400593
ACKR2	4.63474841	-3.42946627	2.44557276	-1.40231619	0.16082085	0.43311119	-0.79365765	0.363400593
RPN1	65.3945576	-3.18622	2.27160506	-1.40262938	0.16072739	0.43311119	-0.79391012	0.363400593
TMEM91	90.6972785	-2.41953304	1.72191038	-1.40514458	0.15997829	0.4325124	-0.79593895	0.364001437
REPS1	6.55732424	-3.39867421	2.39857029	-1.41695835	0.1564951	0.42591217	-0.80549924	0.370679955
ACTR10	3.57497975	-3.73840085	2.62045393	-1.42662339	0.15368848	0.42074874	-0.81335869	0.375977178
ZNF776	13.6283952	-4.18847187	2.92469605	-1.43210501	0.15211377	0.41891649	-0.81783147	0.37787254
RABL2A	3.83908542	-4.24014791	2.95825253	-1.43332858	0.15176396	0.41842492	-0.81883136	0.378382455
BANK1	3.79473972	-3.46505189	2.41741409	-1.43337127	0.15175177	0.41842492	-0.81886625	0.378382455
CTSA	593.329543	-2.16626922	1.51022094	-1.43440549	0.15145658	0.418213	-0.81971185	0.378602473
NAP1L1	456.288925	-1.35774599	0.94389428	-1.43845133	0.15030604	0.41586529	-0.82302358	0.381047322
DYRK3	4.53769218	-4.04427814	2.80771808	-1.44041461	0.14975013	0.41515283	-0.82463278	0.381791995
SCIMP	3.15923788	-3.75702138	2.60172225	-1.44405168	0.14872444	0.41285904	-0.82761766	0.384198198
DOK3	106.950237	-3.58578317	2.47853274	-1.44673625	0.14797081	0.41159014	-0.82982396	0.385535037
BACH2	3.62334543	-3.54319073	2.44035762	-1.45191455	0.14652537	0.40838797	-0.83408719	0.388927062
PKM	43.7090285	-2.48406067	1.71006209	-1.45261431	0.14633087	0.40838797	-0.83466405	0.388927062
FAM122C	55.0054186	-4.23316462	2.90067095	-1.45937429	0.14446212	0.40480502	-0.84024602	0.392754112
PCLO	3.5120403	-3.29705491	2.25409701	-1.46269433	0.14355104	0.40330025	-0.84299366	0.39437151
ANAPC15	6.81408217	-3.89301698	2.65481414	-1.46639907	0.1425396	0.40130825	-0.84606446	0.396521917
CNTN5	3.21377	-4.34518165	2.96046743	-1.467735	0.14217622	0.40082721	-0.84717303	0.397042809
YPEL2	4.96741747	-3.31829597	2.25878647	-1.46906138	0.14181614	0.40008293	-0.84827434	0.397849973
SERPINE2	58.8972957	-2.90198537	1.97195226	-1.47163064	0.14112065	0.39839076	-0.85040944	0.399690742
PPIF	3.65094864	-4.1403713	2.81263274	-1.47206254	0.14100399	0.39833149	-0.8507686	0.399755363
WIPI1	40.1763371	-2.93208194	1.99096582	-1.47269325	0.14083377	0.39819445	-0.85129321	0.399904796
RILP	69.4524692	-2.69261701	1.82084165	-1.47877604	0.13920018	0.39449456	-0.8563602	0.403958977
MUC3A	8.35404549	-2.79654223	1.89029833	-1.47941845	0.13902851	0.39447013	-0.85689613	0.403985876
RGS10	134.583298	-2.72559717	1.84088734	-1.4805888	0.13871618	0.39427589	-0.85787287	0.404199777
ITGB3	356.397066	-1.85161233	1.24899907	-1.48247695	0.13821343	0.39365303	-0.85944975	0.404886398
C15orf39	8.69328716	-2.7303393	1.84029979	-1.48363832	0.1379049	0.39331232	-0.86042031	0.405262445
TRNAU1AP	3.25149205	-3.93131936	2.6420138	-1.48800107	0.13675061	0.39037173	-0.86407073	0.408521645
TEC	64.2867048	-3.79874549	2.54777411	-1.49100561	0.13596002	0.38967671	-0.86658877	0.409295547
SNX13	42.9928349	-2.98236117	1.99990515	-1.49125131	0.13589553	0.38967671	-0.86679484	0.409295547
FXR1	5.37374788	-3.21598434	2.15559481	-1.49192433	0.13571898	0.38967671	-0.8673594	0.409295547
SMAD5	29.6240187	-2.64802817	1.77284579	-1.49365962	0.13526461	0.38924798	-0.86881583	0.409773636
GPD2	2.95308674	-3.5952829	2.40386439	-1.49562634	0.13475106	0.38803831	-0.87046783	0.411125398
ZNF84	3.44099859	-4.42964833	2.96031285	-1.49634466	0.13456386	0.38776742	-0.87107156	0.411428682

GTPBP4	2.73583703	-4.44684007	2.9621829	-1.50120375	0.13330287	0.38573534	-0.8751605	0.413710569
MLLT1	5.28175598	-4.43610188	2.95494989	-1.50124437	0.13329237	0.38573534	-0.87519472	0.413710569
PFKM	32.8968478	-3.24683652	2.16220426	-1.50163265	0.13319201	0.38573534	-0.87552184	0.413710569
ERCC6L2	2.74137781	-4.44979619	2.96217644	-1.50220498	0.13304418	0.38573534	-0.87600412	0.413710569
USP40	6.30329977	-3.94570594	2.6250445	-1.50310059	0.13281311	0.38538939	-0.87675906	0.414100244
IMPA1	2.73559992	-4.44743527	2.95742931	-1.50381795	0.13262825	0.38517967	-0.87736396	0.414336638
FRMD4B	419.158667	-2.62062498	1.74172807	-1.50461201	0.13242386	0.38517967	-0.87803377	0.414336638
KLF7	5.97688934	-3.99696678	2.65358145	-1.50625366	0.13200207	0.38464424	-0.87941926	0.41494077
TANC1	2.764114	-4.46222652	2.96214942	-1.50641507	0.13196066	0.38464424	-0.87955554	0.41494077
LDLRAP1	196.61907	-2.99778056	1.98592139	-1.50951622	0.13116691	0.38382222	-0.8821757	0.415869882
ZNF281	19.3514151	-4.4075713	2.91922959	-1.50984058	0.13108411	0.38382222	-0.88244996	0.415869882
APC	2.92993535	-4.20039577	2.77781449	-1.51212249	0.1305027	0.382772	-0.88438049	0.417059841
CCND3	319.621597	-2.70656056	1.78894314	-1.51293828	0.13029534	0.382772	-0.88507113	0.417059841
ATP6AP1	3.69265634	-4.18961995	2.76643372	-1.51444798	0.12991226	0.38249412	-0.88634987	0.417375233
GLIPR1	3.55256887	-4.48759177	2.96021192	-1.51596976	0.129527	0.38223427	-0.8876397	0.417670375
APLP2	2.84525951	-4.50340138	2.96206156	-1.52036049	0.1284204	0.37978873	-0.89136599	0.420457923
RNMT	8.3771522	-3.89665089	2.55996681	-1.52214899	0.12797175	0.37900027	-0.89288588	0.421360481
NAB1	13.2746912	-3.87204519	2.54131011	-1.52364136	0.12759833	0.37816329	-0.89415502	0.422320628
SYNE3	3.04453182	-4.26017485	2.79089583	-1.52645427	0.12689677	0.37688884	-0.89654943	0.423786721
CRNKL1	17.5097065	-3.212761	2.10207395	-1.52837677	0.12641902	0.37600628	-0.89818759	0.424804901
FRK	5.10891636	-3.14259496	2.05268779	-1.53096588	0.12577783	0.37480141	-0.90039591	0.426198781
TMCC1	2.93052696	-4.54389066	2.96197757	-1.53407328	0.12501162	0.37342066	-0.9030496	0.427801653
MT.ND3	122.749135	-2.43500555	1.5859629	-1.53534837	0.12469828	0.37275207	-0.90413954	0.428579935
AFAP1L2	26.9332349	-3.37357666	2.1961764	-1.5361137	0.1245105	0.37245812	-0.90479403	0.428922549
POLK	3.24225864	-4.34800257	2.82776616	-1.53761037	0.12414391	0.3721636	-0.90607458	0.429266105
RFTN2	8.37346563	-3.59904446	2.33811623	-1.53929236	0.12373294	0.37119881	-0.90751468	0.430393423
LEMD2	30.6023328	-3.49698574	2.27057596	-1.54013158	0.12352828	0.37085204	-0.90823359	0.430799332
POLL	3.29321557	-3.47113343	2.2536074	-1.5402565	0.12349784	0.37085204	-0.90834063	0.430799332
CCDC157	3.239586	-4.00525521	2.59942654	-1.54082262	0.12335996	0.37085204	-0.90882577	0.430799332
MAP4	6.6262052	-3.85669108	2.49339749	-1.54676144	0.12192078	0.36814948	-0.91392226	0.433975807
LASP1	25.4186093	-4.11337213	2.65741568	-1.54788435	0.12165014	0.3676916	-0.91488737	0.434516297
PYGO2	3.01735544	-4.58802295	2.96188867	-1.54901938	0.12137706	0.36757388	-0.91586338	0.434655359
RDH11	253.54785	-3.13517039	2.02343365	-1.54943078	0.1212782	0.3675437	-0.91621726	0.43469102
NOTCH2	4.62227454	-4.17029817	2.6902401	-1.55015835	0.12110351	0.3675437	-0.91684326	0.43469102
SAP30	2.97977442	-4.56816037	2.94613865	-1.55055851	0.12100752	0.3675437	-0.91718764	0.43469102
AMFR	8.20332999	-2.66120181	1.71551922	-1.55125153	0.12084142	0.3675437	-0.91778419	0.43469102
MAX	801.929602	-2.14674425	1.38006786	-1.55553528	0.11981864	0.36604902	-0.92147562	0.436460756
COASY	3.05952413	-4.60792453	2.96184946	-1.5557592	0.11976536	0.36604902	-0.92166876	0.436460756
KLHL9	3.0736938	-4.61361224	2.96183835	-1.55768536	0.11930785	0.36529257	-0.92333097	0.437359158
ZHX3	3.10934799	-4.63130358	2.96180408	-1.56367655	0.11789355	0.36127454	-0.92850996	0.442162648
RAB11A	96.0397335	-2.43107474	1.55304861	-1.5653565	0.11749934	0.36055067	-0.92996457	0.443033694
ZC2HC1A	15.0433126	-4.31992524	2.75855735	-1.56600886	0.11734654	0.36034735	-0.9305297	0.443278665
TMEM70	3.12774649	-4.63980684	2.96178776	-1.56655616	0.11721847	0.36021972	-0.93100394	0.443432516
SSR3	3.13442654	-4.64189844	2.96178376	-1.56726447	0.11705289	0.35997653	-0.93161787	0.443725814
ACSS2	148.342899	-4.23592498	2.69722218	-1.57047685	0.11630422	0.35820323	-0.93440454	0.445870508
HSD17B3	3.1539043	-4.65015931	2.95056344	-1.57602418	0.11502023	0.35556366	-0.93922576	0.449082637
CASC4	12.3337721	-4.38356389	2.77946899	-1.57712279	0.11476727	0.35533022	-0.94018193	0.449367857
PCNX3	28.7690172	-2.73902468	1.73672171	-1.57712353	0.1147671	0.35533022	-0.94018258	0.449367857
ACVR1C	3.21146624	-4.67575559	2.9617198	-1.5787299	0.11439802	0.3549578	-0.94158148	0.449823271
ATP13A2	15.4566082	-3.56314671	2.25671959	-1.57890538	0.11435776	0.3549578	-0.94173435	0.449823271
SLC11A1	3.21693725	-4.67836031	2.96171494	-1.57961195	0.11419576	0.35485907	-0.94235002	0.449944094
KBTBD12	3.50330936	-4.46035254	2.81614176	-1.58385228	0.11322734	0.35290318	-0.9460487	0.452344433
TMEM55A	13.3761006	-3.81770321	2.40943566	-1.58448025	0.11308447	0.35272191	-0.94659702	0.452567568
SLC38A10	4.1741989	-3.33154541	2.1023514	-1.58467582	0.11304001	0.35272191	-0.94676781	0.452567568
ACADM	3.26380225	-4.69944594	2.96167593	-1.58675225	0.11256877	0.35261401	-0.94858209	0.452700442
RSRC1	6.63786756	-3.93562445	2.47360884	-1.59104559	0.11159932	0.35018806	-0.95233847	0.455698671
TMEM8A	3.84813539	-4.25429159	2.6692289	-1.59382793	0.11097458	0.34980935	-0.95477649	0.45616859
CPEB2	38.3968657	-3.39284639	2.12771194	-1.59459855	0.11080204	0.34979506	-0.95545226	0.456186331
TPI1	3.95299375	-4.72559298	2.96022282	-1.59636395	0.11040756	0.34907903	-0.95700118	0.457076238
PPP6C	8.45327745	-3.69401305	2.31270652	-1.5972684	0.11020589	0.34897135	-0.95779519	0.457210223
SHKBP1	4.63184404	-4.52169287	2.8302231	-1.59764538	0.11012192	0.34897083	-0.95812622	0.457210869
MRPL13	3.29371864	-4.7135585	2.95012774	-1.59774725	0.11009924	0.34897083	-0.95821569	0.457210869

SVIP	12.8132338	-3.25714233	2.03798981	-1.59821326	0.10999552	0.34897083	-0.95862498	0.457210869
USP39	3.35172234	-4.73842361	2.9616053	-1.59995108	0.10960944	0.34867356	-0.96015206	0.457580983
IGF2BP3	2.86381112	-4.51074207	2.78892413	-1.61737711	0.1057969	0.33861515	-0.97552705	0.470293621
RIT1	61.5402099	-3.85174071	2.38085962	-1.61779413	0.10570697	0.33858757	-0.97589636	0.470328996
PIAS2	7.50104642	-3.00220116	1.85266641	-1.62047584	0.1051301	0.33734932	-0.97827292	0.471920159
MFNG	3.50089838	-4.80131615	2.96149528	-1.62124727	0.10496462	0.33724743	-0.97895707	0.472051351
PF4	267.804584	-2.5029093	1.54334932	-1.62173868	0.10485931	0.33724743	-0.979393	0.472051351
TRIO	71.8972111	-3.43893953	2.12047018	-1.6217816	0.10485012	0.33724743	-0.97943107	0.472051351
ZNF106	7.28915962	-3.8960061	2.40092626	-1.6227096	0.1046515	0.33724743	-0.98025455	0.472051351
ZBTB8OS	7.90238163	-4.03456258	2.48201205	-1.62552095	0.1040516	0.33612946	-0.98275122	0.473493417
TMEM160	3.59517407	-4.48578246	2.75953374	-1.62555811	0.10404369	0.33612946	-0.98278424	0.473493417
PLCB3	16.8105966	-2.55967973	1.5742862	-1.62593036	0.10396447	0.33612946	-0.98311505	0.473493417
TM9SF1	3.56669574	-4.82819994	2.9614497	-1.63035015	0.10302751	0.33437769	-0.98704678	0.475762711
CASP4	3.59828003	-4.83942187	2.96143092	-1.63414984	0.10222739	0.33255848	-0.99043272	0.47813197
EVI2B	3.57943311	-4.83376316	2.95661528	-1.63489758	0.10207052	0.33255848	-0.99109967	0.47813197
AHSA2	6.61311228	-4.02740782	2.44988829	-1.64391489	0.10019378	0.32825092	-0.99915922	0.483794047
SLC40A1	76.3423514	-3.20534383	1.94781531	-1.64560974	0.09984413	0.3276686	-1.00067745	0.48456518
UBE2G1	12.3257783	-3.97437085	2.41422034	-1.64623369	0.09971566	0.3276686	-1.00123665	0.48456518
APOBEC3F	3.70271724	-4.8827522	2.96135976	-1.64882101	0.09918431	0.32648495	-1.00355703	0.486136835
PARN	6.88044686	-4.16960858	2.52218069	-1.653176	0.09829505	0.32430811	-1.00746835	0.489042189
AMIGO2	7.13367434	-3.60403439	2.17945898	-1.65363718	0.09820125	0.3242744	-1.00788297	0.489087333
ERCC3	3.13139899	-4.64180003	2.80343837	-1.65575248	0.09777196	0.32311303	-1.0097857	0.490645525
HPS3	3.79117932	-4.91537999	2.96130757	-1.65986811	0.09694099	0.32087621	-1.01349256	0.493662477
DDX3Y	3.78817993	-4.91541384	2.96130752	-1.65987957	0.09693868	0.32087621	-1.01350288	0.493662477
TRPC6	3.79887375	-4.91904428	2.96130178	-1.66110874	0.09669161	0.32081583	-1.01461121	0.493744217
REPS2	18.3435045	-3.44432398	2.06833869	-1.6652611	0.09586067	0.31831248	-1.01835952	0.497146333
SLC39A8	3.85316486	-4.93888125	2.9612707	-1.66782498	0.09535048	0.3173776	-1.02067714	0.498423725
PTGS1	207.950824	-2.03727665	1.21960546	-1.67043909	0.09483252	0.31679855	-1.02304271	0.499216816
PARP8	3.03379366	-4.25309389	2.53878459	-1.67524803	0.09388558	0.31426008	-1.02740112	0.50271078
STRN	3.91481296	-4.96183459	2.96001206	-1.67628864	0.09368167	0.31421226	-1.02834538	0.502776875
BCL10	3.02889077	-4.59165821	2.73777877	-1.67714728	0.09351369	0.31402499	-1.02912482	0.503035788
UBOX5	3.93265047	-4.96901668	2.9612243	-1.6780278	0.09334167	0.31395672	-1.02992442	0.50313021
LSM1	3.98161051	-4.98680921	2.96119735	-1.68405163	0.09217169	0.31102344	-1.03540244	0.507206879
ARNT	19.1101947	-3.70623954	2.19805644	-1.68614394	0.09176808	0.31016419	-1.03730836	0.50840835
CLASP1	29.9015071	-4.22546128	2.50150558	-1.68916724	0.09118739	0.30870266	-1.04006524	0.510459628
MYEF2	3.91898398	-4.51776857	2.67006934	-1.69200421	0.09064517	0.30748458	-1.04265533	0.512176656
PELI1	5.04129392	-4.17705564	2.46813336	-1.69239463	0.09057076	0.30748458	-1.04301201	0.512176656
PARP9	4.05936888	-5.01350865	2.96115753	-1.69309083	0.09043818	0.30748458	-1.04364819	0.512176656
CEP68	27.6032265	-4.08821809	2.41059078	-1.69594032	0.08989718	0.30650003	-1.04625392	0.513569473
NUDC	3.79237106	-4.14194216	2.44029512	-1.69731199	0.08963769	0.30614379	-1.04750933	0.514074547
ZCCHC7	4.11118914	-5.03158518	2.96113099	-1.69921061	0.08927951	0.30547237	-1.04924821	0.515028066
RGS12	5.15008992	-4.92501383	2.89546915	-1.70093811	0.08895461	0.30473567	-1.05083155	0.516076701
DNASE1	5.15396971	-4.24458852	2.49527376	-1.70105124	0.08893337	0.30473567	-1.05093527	0.516076701
GAB1	56.692073	-4.6563564	2.73192531	-1.70442302	0.08830209	0.30387594	-1.05402904	0.517303688
DCN	4.16217367	-5.05159733	2.96110198	-1.7059856	0.08801076	0.30337484	-1.05546422	0.518020435
AGPAT5	3.21542993	-4.32219493	2.53323692	-1.70619452	0.08797187	0.30337484	-1.05565618	0.518020435
SLFN13	15.1361946	-4.14979324	2.42644116	-1.71023856	0.08722177	0.30115376	-1.05937512	0.521211704
MIGA2	6.48025177	-3.97494439	2.3223473	-1.71160635	0.08696924	0.30103067	-1.06063434	0.521389248
FAM206A	4.24007783	-5.07704306	2.96106568	-1.71459995	0.0864186	0.29962285	-1.06339278	0.523425066
AP1M1	4.03080813	-3.26751622	1.90226446	-1.71769819	0.08585168	0.29790533	-1.06625121	0.525921732
NECAB1	4.27660988	-5.08933608	2.96104838	-1.71876154	0.0856578	0.29772878	-1.06723308	0.526179179
PSMC2	10.1462583	-5.07810014	2.95028286	-1.72122484	0.08521003	0.29691596	-1.06950927	0.527366454
RAB3B	3.37922486	-4.49595495	2.60661926	-1.72482227	0.08455951	0.29527042	-1.07283756	0.529780052
DHX15	72.046514	-3.34766047	1.93826911	-1.72713915	0.08414268	0.29492433	-1.07498368	0.530289402
WDR20	8.05073329	-3.87739922	2.24459294	-1.72743982	0.0840887	0.29492433	-1.07526234	0.530289402
DAPP1	55.8723859	-3.87721565	2.2416078	-1.72965835	0.08369133	0.29408499	-1.07731951	0.531527139
PTPN6	25.0013239	-4.0435639	2.33081101	-1.7348313	0.08277069	0.29183501	-1.08212343	0.534862603
DOCK10	3.73890232	-4.26526897	2.45856466	-1.73486142	0.08276535	0.29183501	-1.08215142	0.534862603
MT.ND4	542.739493	-1.72411391	0.99377823	-1.73490812	0.08275708	0.29183501	-1.08219484	0.534862603
RTF1	7.50423737	-3.78789852	2.18139476	-1.73645714	0.08248304	0.29156143	-1.08363536	0.535269933
GIT1	4.48535572	-5.00064483	2.87203554	-1.74115005	0.08165728	0.2893795	-1.08800509	0.538532239
DCTN5	3.5822342	-4.49649816	2.57324107	-1.74740649	0.08056685	0.2864905	-1.09384361	0.542889781

PDC	4.55988662	-5.18167122	2.96092297	-1.75001892	0.08011505	0.28561564	-1.0962859	0.54421802
VPS4B	10.3680133	-4.74007638	2.70681458	-1.75116405	0.07991766	0.28515606	-1.09735726	0.544917396
EIF4E3	2.74270198	-4.44765827	2.52889155	-1.75873824	0.07862198	0.2822258	-1.10445602	0.549403293
HIST1H3E	2.82854393	-4.49297067	2.55354108	-1.75950593	0.07849161	0.28200093	-1.10517674	0.549749453
KNTC1	3.55317692	-4.49060849	2.54872614	-1.7619031	0.07808567	0.28078474	-1.10742866	0.551626494
IREB2	4.31207474	-4.08901034	2.31711929	-1.76469565	0.07761493	0.28030231	-1.11005473	0.552373317
U2AF1L4	3.51039072	-3.71232418	2.10234494	-1.76580166	0.07742913	0.2801172	-1.11109562	0.552660221
SLC25A5	4.71775121	-5.23196512	2.96085796	-1.7670436	0.07722093	0.2796069	-1.11226499	0.553452112
MED20	2.7415307	-4.44976106	2.51444272	-1.76968082	0.07678033	0.27849589	-1.11475005	0.555181213
SEMA4B	7.19762246	-3.60256262	2.02676726	-1.77749201	0.07548732	0.27452333	-1.12212597	0.561420736
ROCK2	32.6298208	-3.65070173	2.04370272	-1.7863174	0.07404788	0.27031033	-1.13048737	0.568137358
DPP8	17.0074036	-3.87073814	2.16250344	-1.78993387	0.07346454	0.26881051	-1.13392222	0.570553757
ASRGL1	3.6277419	-4.60012176	2.56371905	-1.79431586	0.07276277	0.26671141	-1.13809079	0.573958401
PPA1	3.84000705	-2.36939656	1.31939564	-1.79581961	0.07252321	0.26662048	-1.13952298	0.574106502
SMG6	7.03749827	-4.7328597	2.63533543	-1.79592307	0.07250675	0.26662048	-1.13962155	0.574106502
SETD3	4.04902213	-4.6502966	2.5847092	-1.79915659	0.07199391	0.26529439	-1.14270422	0.576271942
KCMF1	2.91756652	-4.28283501	2.37702373	-1.80176368	0.0715826	0.26424639	-1.14519256	0.57799094
HADH	6.52218838	-5.33300081	2.959037	-1.80227581	0.07150202	0.26418316	-1.14568168	0.578094871
COG7	5.35317516	-3.69057334	2.04615542	-1.80366228	0.07128427	0.26367375	-1.14700631	0.578933111
SF3B3	4.3956318	-4.80203049	2.66079415	-1.80473581	0.07111603	0.26367375	-1.14803248	0.578933111
BAMBI	4.04088743	-4.33488514	2.38847667	-1.81491626	0.06953678	0.25966091	-1.15778543	0.585593424
NDEL1	8.56689662	-3.6855657	2.02841743	-1.8169661	0.0692223	0.25913749	-1.15975395	0.586469758
GAB2	10.5174819	-5.36771509	2.95322969	-1.81757454	0.06912919	0.25909445	-1.16033856	0.586541894
DDAH2	5.50808345	-4.49204911	2.46408671	-1.82300772	0.06830222	0.25738502	-1.16556517	0.589416731
HMGN2	5.42614066	-4.53824122	2.47987084	-1.83003128	0.06724526	0.25455388	-1.17233831	0.594220278
KDM4B	4.0249473	-3.1583255	1.71800552	-1.83836749	0.06600827	0.25170187	-1.18040163	0.599113549
EIF2B4	4.50132915	-5.46363918	2.96176937	-1.84472135	0.06507808	0.24883851	-1.18656524	0.6040824
PPP1R16A	4.8378704	-4.03394399	2.18133772	-1.84929823	0.06441476	0.24675535	-1.1910146	0.607733422
DYTN	4.59687362	-4.83687564	2.61291177	-1.85114388	0.06414886	0.24618971	-1.19281108	0.608730095
MEIS1	817.300671	-2.29989745	1.24207457	-1.85165811	0.06407493	0.24618971	-1.19331184	0.608730095
PGPEP1	6.76589971	-4.75336815	2.56350747	-1.85424393	0.06370427	0.24584959	-1.19583146	0.609330511
CCP110	5.71357866	-5.50809469	2.96053873	-1.86050419	0.06281423	0.24376369	-1.20194198	0.613030983
CNST	423.367735	-2.21770542	1.19170816	-1.86094674	0.0627517	0.2437482	-1.20237452	0.613058585
LGALSL	158.068987	-5.20505002	2.7884202	-1.8666663	0.0619482	0.24152838	-1.20797129	0.617031823
HDAC7	5.80957673	-5.53132115	2.96051455	-1.86836479	0.06171124	0.24083	-1.20963572	0.618289416
HDAC1	188.415456	-3.94607683	2.10802003	-1.87193517	0.06121557	0.23957917	-1.21313808	0.620550953
PTAFR	11.0433776	-3.23546596	1.7279987	-1.87237754	0.06115439	0.23957917	-1.21357236	0.620550953
RNF139	5.81522506	-5.55992594	2.96077198	-1.8778636	0.06039983	0.23771728	-1.21896431	0.623939253
MYLK	37.4909584	-2.81196898	1.49725271	-1.87808575	0.06036944	0.23771728	-1.21918288	0.623939253
ULK1	60.5060777	-2.70622149	1.43875579	-1.88094569	0.05997931	0.23673349	-1.22199855	0.625740291
LZTS2	19.829986	-4.29052949	2.2791819	-1.88248665	0.05976997	0.23635533	-1.22351695	0.626434603
MOV10	4.99596174	-4.63291726	2.460921	-1.88259487	0.05975529	0.23635533	-1.22362363	0.626434603
TOX4	13.5702497	-3.89372109	2.06644773	-1.88425821	0.05953006	0.23585458	-1.2252637	0.627355692
SUPT6H	5.42693295	-4.98981426	2.64656621	-1.88539181	0.05937696	0.23547205	-1.22638205	0.628060628
DSE	34.6385274	-3.65801807	1.93385186	-1.89157098	0.05854817	0.23285058	-1.23248671	0.632922671
ZNF765	5.80299039	-4.46088005	2.35679846	-1.89277111	0.05838831	0.23243685	-1.23367406	0.633695024
UBE2C	9.13329101	-5.60278299	2.95764182	-1.89434128	0.05817973	0.23205017	-1.23522834	0.634418113
VPS8	6.56247513	-4.34505862	2.29216239	-1.89561553	0.0580109	0.231887	-1.23649038	0.634723596
OPRD1	8.97135537	-3.47524688	1.83083748	-1.89817333	0.05767325	0.23113708	-1.23902556	0.636130382
TMEM41B	4.21248941	-4.3668879	2.29466384	-1.90306215	0.05703244	0.22905123	-1.24387808	0.640067374
CCDC47	11.8392195	-5.65167327	2.95419102	-1.91310353	0.0557348	0.22575847	-1.25387355	0.646355945
SIRT2	4.15054973	-4.12866761	2.14240706	-1.92711631	0.05396514	0.22138998	-1.26788672	0.654842046
SNRNP200	15.2760382	-5.26791589	2.73107407	-1.92888064	0.05374568	0.2211433	-1.26965642	0.655326211
SLX4IP	3.39497171	-4.75813398	2.46635413	-1.92921767	0.05370385	0.2211433	-1.26999462	0.655326211
CXCL16	6.60852605	-5.71785659	2.96033391	-1.93149042	0.05342243	0.22090468	-1.27227634	0.655795087
HOOK3	67.3375809	-2.91182373	1.50383512	-1.93626528	0.05283522	0.21956672	-1.27707649	0.658433495
C3orf52	19.5109273	-5.70616537	2.94323682	-1.9387381	0.05253324	0.21899881	-1.27956584	0.659558252
FAM212A	533.900237	-2.31510836	1.19249864	-1.94139287	0.05221064	0.21849761	-1.28224096	0.660553319
DDX46	5.27648096	-5.3929219	2.77469073	-1.94361189	0.05194227	0.21759317	-1.28447907	0.662354732
ARHGAP32	22.0807446	-4.2605823	2.18950576	-1.94591053	0.05166549	0.21720018	-1.28679946	0.663139822
MRNIP	4.89753632	-4.83887981	2.47773277	-1.95294661	0.05082592	0.21529923	-1.29391473	0.666957528
TRAPPC8	6.95204144	-5.79074376	2.96026943	-1.95615429	0.05044698	0.21434819	-1.29716482	0.668880176

XYLT2	12.2984351	-4.98250157	2.53603431	-1.96468224	0.04945103	0.21162803	-1.30582469	0.674426809
TRIM8	6.34183987	-4.62123931	2.35200498	-1.96480847	0.04943641	0.21162803	-1.30595308	0.674426809
TSC1	8.08775061	-5.68485081	2.88504585	-1.97045423	0.04878634	0.20979261	-1.3117018	0.678209816
DFFA	3.02775892	-3.77118398	1.91378092	-1.97054111	0.04877639	0.20979261	-1.31179036	0.678209816
CRAT	257.059293	-3.89178426	1.97302371	-1.97249747	0.04855285	0.2092899	-1.31378531	0.67925172
IGFN1	7.25038265	-5.16616251	2.61388795	-1.97642845	0.04810627	0.20801092	-1.31779829	0.68191386
RERE	22.6209656	-3.93580038	1.98932336	-1.97846185	0.04787663	0.20723314	-1.31987645	0.683540796
PNMA1	43.1619571	-4.73790745	2.38657766	-1.98523079	0.0471188	0.20544784	-1.32680577	0.687298425
ZCCHC24	5.29499653	-5.34883641	2.69359792	-1.98575903	0.04706009	0.20540692	-1.32734728	0.68738493
MT.ND4L	274.609642	-1.76926927	0.88479186	-1.99964461	0.04553865	0.20044709	-1.34161982	0.698000236
TMEM104	12.0360177	-3.64346351	1.82103337	-2.00076702	0.0454175	0.20012538	-1.34277674	0.69869783
BCAP31	8.07207943	-5.32454411	2.6558815	-2.00481238	0.04498311	0.19863167	-1.34695053	0.701951506
TMEM140	150.730007	-3.80250492	1.89352198	-2.00816518	0.04462574	0.19809596	-1.35041456	0.703124379
WBSCR22	8.02938863	-5.32970646	2.6478906	-2.01281218	0.04413439	0.19692691	-1.35522285	0.705694929
CDK5RAP3	5.80402445	-4.85666117	2.40626433	-2.01834068	0.04355579	0.19564868	-1.36095407	0.708523086
NCOA1	13.2318638	-3.56152885	1.75370068	-2.03086472	0.04226872	0.19277869	-1.37398091	0.71494097
MFSD5	8.19377599	-6.02788146	2.96008081	-2.03639084	0.04171112	0.19107274	-1.37974811	0.718801268
AHI1	24.717916	-5.30389582	2.60253277	-2.03797465	0.04155247	0.19107274	-1.38140318	0.718801268
COQ8A	6.42595738	-4.22362005	2.06770493	-2.04266091	0.04108602	0.19051467	-1.38630596	0.72007158
SIGLEC1	6.36897598	-5.33618352	2.60959848	-2.04482933	0.04087169	0.18994387	-1.38857744	0.721374727
RHOG	4.69437874	-5.22377894	2.54840822	-2.04982032	0.04038197	0.18850955	-1.39381252	0.724666649
HLTF	92.1390417	-3.22945619	1.57113758	-2.05548912	0.03983179	0.18763422	-1.39977023	0.72668796
GEN1	3.410144	-4.43101105	2.15538504	-2.0557863	0.03980312	0.18763422	-1.40008289	0.72668796
CDC42BPA	91.5040976	-3.81647202	1.85563376	-2.05669464	0.03971561	0.18763422	-1.40103878	0.72668796
TBC1D15	211.109122	-3.48995929	1.69359551	-2.06068053	0.03933353	0.18633084	-1.40523709	0.729715247
BTG2	11.8912563	-5.09086359	2.46560975	-2.06474832	0.03894682	0.18491968	-1.409528	0.733016869
RAP1GAP2	15.774327	-4.12262027	1.99080575	-2.07083001	0.03837469	0.18386631	-1.41595518	0.735497846
COL11A2	6.27833918	-6.13766182	2.96120833	-2.07268829	0.0382013	0.18339502	-1.41792188	0.736612452
BCORL1	3.40336363	-3.94991832	1.9046791	-2.07379727	0.03809814	0.18318784	-1.41909619	0.737103364
TOB1	23.1463129	-5.2167667	2.51450774	-2.07466718	0.03801739	0.18301088	-1.42001769	0.73752308
ADAM9	5.89876892	-4.78815245	2.30407816	-2.07812067	0.03769825	0.18265317	-1.42367886	0.738372792
BCAP29	7.06445668	-5.81368136	2.79633754	-2.07903419	0.03761421	0.18254727	-1.42464808	0.738624648
FYN	195.847115	-2.37986497	1.14048482	-2.08671342	0.03691405	0.18041091	-1.43280833	0.743737203
HECW1	6.74525375	-5.08430667	2.43562421	-2.08747583	0.03684514	0.18034547	-1.43361975	0.743894754
NIT1	7.17982098	-5.83757043	2.79075144	-2.09175577	0.03646037	0.17948108	-1.43817894	0.745981322
CLIC4	15.5674364	-4.66082009	2.2252476	-2.09451752	0.0362139	0.17887864	-1.44112465	0.747441505
ANGEL2	11.9120689	-5.19857499	2.47898132	-2.09706098	0.03598818	0.17808843	-1.44384014	0.749364297
LUC7L2	5.92616428	-4.52058283	2.14410813	-2.10837447	0.03499861	0.17495104	-1.45594919	0.757083468
SLC39A3	12.5508265	-3.29306043	1.56175015	-2.10857059	0.03498166	0.17495104	-1.45615954	0.757083468
C6orf106	5.14344175	-5.35708052	2.53143955	-2.11621902	0.03432618	0.17389324	-1.46437447	0.759717305
PSMA5	7.26848689	-5.17273852	2.44236359	-2.11792321	0.03418157	0.17357569	-1.46620797	0.760511104
TRAPPC3L	5.22946618	-5.37971665	2.52725599	-2.12867896	0.03328083	0.17066672	-1.47780584	0.767851147
GREB1	5.35699822	-4.16046905	1.9519316	-2.13146252	0.03305106	0.17028119	-1.48081465	0.768833312
ZBTB37	6.10171179	-5.60362173	2.62265961	-2.136618	0.03262907	0.1696223	-1.48639525	0.770517041
KANTR	21.5352047	-4.91083457	2.28391542	-2.15018233	0.0315408	0.16747824	-1.50112736	0.776041608
LIN54	19.581782	-4.55047443	2.11116269	-2.15543523	0.03112779	0.16670969	-1.50685168	0.778039159
CCDC174	27.8293703	-4.46051679	2.06471263	-2.1603572	0.03074503	0.16604708	-1.51222511	0.779768763
BACH1	5.49785506	-4.38290839	2.01683164	-2.17316523	0.02976788	0.16203068	-1.52625204	0.790402753
TBCE	8.83853673	-6.13736366	2.81437897	-2.18071686	0.02920437	0.16060568	-1.53455223	0.794239101
GNB1	7.64665412	-5.31880656	2.43532536	-2.18402299	0.02896056	0.15972419	-1.53819306	0.796629292
RTKN2	6.15614833	-5.61533432	2.56966436	-2.18524038	0.02887123	0.15965443	-1.53953477	0.796819024
CEP170	12.6451323	-5.92559902	2.70731011	-2.1887404	0.02861571	0.15887444	-1.54339542	0.798945961
SEMA6C	9.01551538	-6.16563064	2.81155472	-2.19296128	0.02831017	0.15802084	-1.54805754	0.801285644
AFAP1	21.0829694	-4.42814923	2.01812607	-2.19418861	0.02822185	0.15773932	-1.54941447	0.802060023
GRB2	5.24465225	-4.9378911	2.24712997	-2.19742123	0.02799038	0.15686666	-1.55299126	0.804469352
PTPN11	12.7179056	-5.92437316	2.69057926	-2.20189505	0.02767272	0.15610834	-1.55794811	0.8065739
RAB7A	56.414968	-5.6525625	2.5624233	-2.20594408	0.02738792	0.15587166	-1.56244102	0.807232828
ALDOA	225.895026	-3.4351189	1.54517636	-2.22312417	0.02620743	0.15220046	-1.58157559	0.817584025
RFC2	11.672107	-5.97731549	2.68767269	-2.22397448	0.02615016	0.15220046	-1.58252563	0.817584025
POU2F2	4.9300287	-5.29657406	2.37612552	-2.22908008	0.02580858	0.15085726	-1.58823594	0.821433796
OTUD6B	9.7946929	-6.28509707	2.8174989	-2.2307363	0.0256986	0.15071687	-1.59009051	0.821838136
CDC42BPB	3.9411116	-4.30417895	1.91144303	-2.25179557	0.02433519	0.14496674	-1.61376519	0.838731641

MICU1	10.8692537	-3.6901368	1.63699364	-2.25421573	0.0241826	0.14496674	-1.61649701	0.838731641
GPANK1	12.0338887	-3.73396179	1.65551314	-2.25547094	0.02410378	0.1448314	-1.61791477	0.83913728
ZDHHC21	6.23771927	-4.97638479	2.20380252	-2.25809016	0.02394004	0.14426385	-1.62087515	0.840842478
ADORA2A	7.82894336	-5.73069816	2.53728676	-2.25859302	0.02390871	0.14426385	-1.62144381	0.840842478
SUGP2	11.7939042	-4.55035659	2.0076914	-2.26646216	0.0234231	0.14322143	-1.6303556	0.843991988
SLX4	29.2141206	-5.29253751	2.32564677	-2.27572716	0.02286234	0.14233724	-1.64087923	0.846681447
SEZ6	15.4990994	-5.69025396	2.49760762	-2.27828179	0.02270979	0.14220087	-1.64378681	0.847097733
TWSG1	70.7464393	-5.13525934	2.25320372	-2.27909234	0.02266158	0.14211266	-1.64470987	0.847367229
CCR4	60.6071145	-4.45119037	1.95028696	-2.28232586	0.02247011	0.14112448	-1.64839483	0.850397634
CHRAC1	3.62640883	-6.77095767	2.96220819	-2.28578048	0.0222671	0.14048518	-1.65233626	0.852369475
EGLN2	20.2998879	-5.81501763	2.51660549	-2.3106592	0.02085169	0.13337392	-1.68085881	0.874929081
FBXO42	22.4406339	-4.32069529	1.86912812	-2.31161002	0.02079918	0.13324277	-1.6819537	0.87535634
CLN3	9.91094024	-5.4388775	2.35116862	-2.3132656	0.02070804	0.13324277	-1.68386099	0.87535634
UBTF	48.5795345	-5.41325794	2.33357396	-2.31972847	0.02035557	0.13183713	-1.69131671	0.879962245
BAG2	8.36205442	-6.05762014	2.60749004	-2.32316137	0.02017049	0.13183713	-1.69528366	0.879962245
ZNF26	14.5926578	-4.70511209	2.01856627	-2.33091782	0.01975769	0.13086597	-1.70426376	0.883173279
RPS27L	31.2561942	-4.79022714	2.05237417	-2.33399309	0.01959608	0.13086597	-1.70783071	0.883173279
PSMG3	7.22889405	-5.40292912	2.30845255	-2.34049823	0.01925803	0.12954836	-1.71538815	0.88756808
GRAMD1C	11.3529316	-6.17708915	2.62802256	-2.35047037	0.0187497	0.12760375	-1.72700566	0.894136559
TBCD	7.49879558	-4.83691145	2.05026413	-2.35916503	0.01831611	0.12502996	-1.7371668	0.902985915
TRIP11	95.5090632	-3.87862152	1.64161941	-2.36268011	0.01814332	0.12466798	-1.74128317	0.904245071
PFKL	12.4290107	-5.58667047	2.36440248	-2.3628255	0.01813621	0.12466798	-1.74145354	0.904245071
ARL15	16.0823629	-4.79597609	2.02464141	-2.36880273	0.01784577	0.12384964	-1.74846473	0.907105264
CAMK2G	9.19720397	-4.49075804	1.86432233	-2.40878842	0.01600557	0.11353869	-1.79572874	0.944856123
GABRB2	7.92769792	-5.08515592	2.09402402	-2.42841336	0.01516505	0.11059065	-1.81915622	0.956281604
DDHD2	17.9844528	-5.08779659	2.09144808	-2.43266694	0.01498808	0.10996484	-1.82425398	0.958746141
MYO18A	5.43837318	-5.4371218	2.23104512	-2.43702906	0.01480849	0.10952495	-1.82948924	0.960486918
SEC14L5	7.15874391	-6.63281422	2.69580192	-2.46042344	0.01387732	0.10583361	-1.85769446	0.975376387
PLEKHG3	150.911473	-5.49572006	2.23087608	-2.46348065	0.01375953	0.10551509	-1.86139633	0.976685443
X1.Mar	10.7732501	-5.76102566	2.33531466	-2.46691624	0.01362822	0.10533289	-1.86556076	0.977435984
MCUR1	10.864096	-6.43531621	2.59010284	-2.48457942	0.01297046	0.10209638	-1.88704471	0.990989662
ZC3H12A	10.4761658	-7.17779507	2.88712841	-2.48613641	0.01291385	0.10203653	-1.88894442	0.991244311
SPOCD1	19.094856	-5.43774751	2.17918772	-2.49530935	0.01258474	0.10122639	-1.90015588	0.99470625
PLEKHA8	7.91321459	-5.02725161	1.99367147	-2.52160483	0.01168209	0.09748338	-1.93247959	1.01106942
KDM2A	79.4730514	-4.32504235	1.71289718	-2.52498656	0.01157027	0.09684675	-1.93665646	1.013914934
ABO	12.6303517	-6.65224897	2.63411353	-2.52542227	0.01155593	0.09684675	-1.93719494	1.013914934
SIAH2	11.8298618	-6.55785128	2.57613364	-2.54561766	0.01090846	0.09327069	-1.96223658	1.03025482
ESYT2	14.6998585	-6.84149554	2.6874554	-2.545715	0.01090542	0.09327069	-1.96235767	1.03025482
ABCC9	11.9857536	-6.24489001	2.44832723	-2.5506762	0.01075142	0.09288153	-1.96853433	1.032070646
CPOX	12.7014737	-6.00101262	2.34376932	-2.56041095	0.01045485	0.0912662	-1.98068239	1.039690044
RTN3	13.8639508	-6.78692876	2.63532001	-2.57537177	0.01001324	0.08871304	-1.9994254	1.052012539
ARHGEF7	118.901975	-5.52591741	2.1429807	-2.57861278	0.00991979	0.08827385	-2.00349746	1.054167936
TOB2	10.9413227	-5.78905457	2.24145585	-2.58272077	0.00980246	0.08777947	-2.00866481	1.056607044
FGFR1OP	14.7474037	-6.1897915	2.3867431	-2.59340501	0.00950308	0.08602352	-2.02213567	1.065382785
SLC12A6	10.2752646	-6.03034322	2.31990985	-2.59938688	0.00933905	0.08509362	-2.02969752	1.070102997
DKC1	61.0857016	-6.08162979	2.33939198	-2.59966258	0.00933155	0.08509362	-2.03004638	1.070102997
MEX3C	14.2846419	-6.50993944	2.47395867	-2.63138569	0.00850375	0.08011222	-2.07038967	1.096301221
YTHDF3	26.1641865	-7.74817717	2.92101318	-2.65256494	0.00798828	0.07737423	-2.09754694	1.111403676
CSE1L	16.2455781	-7.0152949	2.6389139	-2.65840235	0.00785121	0.07674281	-2.10506341	1.114962286
HIVEP2	19.8703323	-5.46411219	2.05281658	-2.66176348	0.00777325	0.07659078	-2.10939749	1.115823493
E2F3	21.0973549	-5.7809559	2.16950255	-2.66464582	0.00770694	0.07659078	-2.11311776	1.115823493
TTC37	10.1779735	-6.01720874	2.25761	-2.66530036	0.00769196	0.07659078	-2.11396305	1.115823493
EFTUD2	21.4651274	-5.36378581	2.00835546	-2.67073529	0.00756853	0.07594063	-2.12098842	1.119525796
WDR11	11.9826622	-7.94251351	2.96053596	-2.68279582	0.00730095	0.0739688	-2.13662033	1.13095143
TM2D2	17.7818908	-7.32097769	2.7287843	-2.68287153	0.0072993	0.0739688	-2.13671864	1.13095143
GALNT5	15.1806246	-6.59653363	2.45418676	-2.68786945	0.00719095	0.07393957	-2.14321371	1.131123052
MGA	7.33772976	-7.7760903	2.87185919	-2.70768509	0.00677543	0.07124465	-2.16906327	1.147247739
LSR	19.5339118	-4.38678557	1.61843618	-2.71050884	0.00671801	0.07118009	-2.17275963	1.147641462
FLYWCH1	14.3586079	-5.85919698	2.15816265	-2.71490056	0.00662957	0.07042224	-2.17851481	1.15229016
SLF2	16.1186065	-6.68333968	2.45111203	-2.72665614	0.00639797	0.06866272	-2.19395798	1.163278982
SMIM3	78.6026421	-7.70200254	2.82151827	-2.7297369	0.00633849	0.06820018	-2.19801426	1.166214506
USP31	21.3681457	-4.81783576	1.75599429	-2.74365115	0.00607601	0.06605873	-2.21638174	1.180069797

CDKN1A	49.142852	-4.46947658	1.6250337	-2.75039008	0.00595244	0.06563459	-2.22530528	1.182867211
LIPT2	14.033316	-7.89826133	2.8675384	-2.75436986	0.00588053	0.06512371	-2.23058374	1.186260861
UBE2J2	30.4296337	-6.7890673	2.42726201	-2.79700637	0.00515785	0.06032948	-2.28753116	1.219470437
FKRP	20.7313222	-7.61431164	2.72042178	-2.79894526	0.00512698	0.06013734	-2.29013814	1.220855769
SCAMP1	15.0355925	-7.60531331	2.70577629	-2.81076944	0.00494232	0.05879226	-2.30606925	1.230679861
ATP7B	32.6735319	-6.4424607	2.28560923	-2.81870611	0.00482176	0.05786117	-2.31679402	1.237612776
SLC25A36	51.9567952	-6.88711359	2.44197486	-2.82030487	0.0047978	0.05786117	-2.31895747	1.237612776
TMEM219	31.2021866	-6.18275574	2.16986374	-2.84937512	0.00438052	0.05412607	-2.35847434	1.26659352
XIRP2	12.5595161	-7.67181752	2.69026604	-2.85169475	0.00434868	0.05389262	-2.36164216	1.268470717
CCAR1	17.8046278	-6.12785669	2.08919694	-2.93311586	0.00335579	0.04464375	-2.47420569	1.350239311
TNFAIP8	8.2822191	-5.7226716	1.94700973	-2.93921058	0.00329049	0.04405665	-2.48273886	1.355988528
NCOA7	15.3462711	-7.90787552	2.66767108	-2.96433679	0.00303336	0.04114305	-2.5180759	1.385703554
CHMP1B	18.1152835	-6.51886048	2.19584069	-2.96873107	0.00299032	0.04095954	-2.52428207	1.387644936
SCAND1	18.7425492	-7.22243503	2.42762514	-2.97510308	0.0029289	0.04066198	-2.5332953	1.390811435
UBALD2	16.8189536	-6.74703566	2.25471423	-2.99241278	0.00276782	0.03920133	-2.55786255	1.406699164
ATG16L2	10.3518595	-7.69818766	2.54062038	-3.03004247	0.00244519	0.03597804	-2.61168676	1.443962479
PNPLA6	11.7810648	-6.55292679	2.12823949	-3.07903636	0.00207671	0.03133128	-2.68262345	1.504021814
NDST1	252.763478	-5.62832174	1.79622301	-3.13342035	0.00172782	0.02777851	-2.76250199	1.556291034
INSIG1	20.6219898	-7.36044685	2.30032886	-3.19973678	0.00137553	0.02337842	-2.86152947	1.631184832
WWC3	16.5006916	-6.69433576	2.08826136	-3.20569823	0.00134735	0.02318336	-2.87051891	1.634823557
POGZ	25.2054563	-7.40265642	2.29901861	-3.21992018	0.00128226	0.02234035	-2.8920229	1.650910083
TMED8	19.1901685	-8.23370277	2.52756087	-3.25756853	0.00112371	0.02044727	-2.94934529	1.689364755
ARL6IP6	32.5000976	-7.88049902	2.40242565	-3.28022598	0.00103724	0.01954329	-2.98412087	1.709002422
UBE2E3	29.7618408	-8.98304575	2.7381659	-3.28067987	0.00103557	0.01954329	-2.98481965	1.709002422
CCPG1	83.2157033	-8.2157583	2.49792724	-3.28903027	0.00100533	0.01920277	-2.99769045	1.716636225
GPATCH8	64.4388658	-8.46770005	2.57025421	-3.29449904	0.00098597	0.01900736	-3.00613506	1.721078093
RNF166	20.3487132	-8.4302158	2.52825925	-3.33439532	0.00085485	0.01736389	-3.06810972	1.760352862
MYBL2	40.4025308	-8.33028578	2.44225527	-3.41089889	0.00064749	0.01375588	-3.18876627	1.861511624
RHOT2	38.3294156	-7.97622985	2.28431741	-3.49173448	0.0004799	0.01080153	-3.31885374	1.966514749
HINFP	53.3205982	-8.15441773	2.31350176	-3.52470781	0.00042395	0.00980738	-3.37268522	2.008447001
TSPAN18	175.547986	-6.34220074	1.75455479	-3.61470657	0.00030069	0.00736509	-3.52188393	2.132822128
NDFIP1	33.3686698	-9.93959741	2.73639312	-3.63237188	0.00028083	0.00696052	-3.55155957	2.157358127
C1orf43	98.1092054	-9.32100887	2.54106917	-3.66814449	0.00024432	0.00620327	-3.61204611	2.207379241
RNF8	46.8115271	-9.06961055	2.41267092	-3.75915773	0.00017049	0.00460977	-3.76831042	2.336320424
SPARC	205.378142	-7.64426994	1.99693342	-3.82800442	0.00012919	0.00368447	-3.88878308	2.433625218
FAM189B	3.93728088	-20.4780971	2.96200624	-6.91359012	4.7254E-12	1.8563E-10	-11.3255621	9.731357279
REEP3	3.89086394	-20.8436663	2.9620333	-7.03694529	1.965E-12	7.7926E-11	-11.7066375	10.10831611
TBCC	5.09638694	-21.116818	2.96150832	-7.13042669	1.0006E-12	4.0062E-11	-11.9997471	10.39726968
TPRA1	3.86835842	-21.1607451	2.96204605	-7.14396224	9.0678E-13	3.6659E-11	-12.0424965	10.43582303
L3HYPDH	4.02927343	-21.2078613	2.96195704	-7.16008404	8.0628E-13	3.2915E-11	-12.0935162	10.48260562
TXNDC17	4.04767193	-21.2233787	2.96194719	-7.16534676	7.759E-13	3.1989E-11	-12.1101948	10.49500543
DOLPP1	4.26845404	-21.2536614	2.96183682	-7.17583808	7.1866E-13	2.9925E-11	-12.1434791	10.5239684
TUFM	4.86843824	-21.4699642	2.96158759	-7.24947804	4.1838E-13	1.796E-11	-12.3784283	10.74568929
PIGA	5.18837948	-21.5579138	2.96147819	-7.27944371	3.352E-13	1.4539E-11	-12.4746967	10.83745716
BEND6	5.38387419	-21.6062519	2.96141898	-7.29591187	2.9664E-13	1.3141E-11	-12.5277659	10.88138298
RAB33B	5.42714415	-21.6116212	2.96140633	-7.29775615	2.9261E-13	1.3101E-11	-12.5337163	10.88268851
RPS19BP1	5.48244936	-21.6185115	2.96138851	-7.30012675	2.875E-13	1.3012E-11	-12.541367	10.88564415
FAM104A	5.9513901	-21.7434654	2.96126534	-7.34262652	2.0944E-13	1.0024E-11	-12.6789354	10.99894396
ZNF394	6.18340494	-21.7863805	2.96121072	-7.35725434	1.8773E-13	9.0897E-12	-12.726463	11.04145069
ULK3	6.90675774	-21.9445219	2.96106393	-7.41102606	1.2533E-13	6.1395E-12	-12.901959	11.21186719
TSPAN15	7.79779247	-22.1096715	2.96092126	-7.46715957	8.1944E-14	4.111E-12	-13.0864808	11.3860482
FBXO3	8.61456745	-22.2399061	2.96081585	-7.51141145	5.8493E-14	3.0077E-12	-13.2328944	11.52176159
DUSP7	9.53611946	-22.3725081	2.96071915	-7.55644386	4.1424E-14	2.2114E-12	-13.3827495	11.65533334
LZTFL1	10.8572453	-22.4133201	2.96060859	-7.57051108	3.7176E-14	2.0104E-12	-13.4297388	11.69671882
FGD4	5.57505847	-21.6537232	2.85190373	-7.59272586	3.1324E-14	1.7163E-12	-13.5041161	11.765419
ZMAT2	13.7006254	-22.7169356	2.96044338	-7.67349098	1.6738E-14	1.0101E-12	-13.7763034	11.9956418
FAM53C	12.8738041	-22.7791759	2.96048396	-7.69440951	1.4215E-14	8.7045E-13	-13.8472559	12.06025407
FAM210A	14.1852503	-22.9179619	2.96042171	-7.74145175	9.8288E-15	6.3159E-13	-14.0074995	12.19956628
CYP46A1	15.2696868	-23.0155086	2.96037863	-7.77451519	7.5737E-15	5.0866E-13	-14.1206925	12.29357343
CRY1	15.7292456	-23.0589659	2.96036189	-7.78923885	6.7414E-15	4.6018E-13	-14.1712495	12.33706861
CCDC30	22.3553372	-22.8779524	2.89094516	-7.91365836	2.4993E-15	1.8584E-13	-14.6021754	12.73085273
TRIM4	25.789152	-23.7297435	2.96014737	-8.01640612	1.0888E-15	9.2529E-14	-14.9630356	13.033721

ITGB8	26.172939	-23.7510425	2.9601425	-8.02361458	1.0268E-15	8.9465E-14	-14.9885221	13.04834649
DIO2	14.8995265	-22.9683095	2.86057875	-8.02925265	9.8068E-16	8.8773E-14	-15.0084717	13.05171885
NT5DC2	13.2026807	-22.82279	2.82603573	-8.07590285	6.6979E-16	6.3387E-14	-15.1740601	13.19800205
TEX15	23.988792	-23.6340526	2.89607744	-8.16071157	3.3306E-16	3.556E-14	-15.4774819	13.4490358
TAF7	36.352547	-24.2002116	2.96004988	-8.17560938	2.9437E-16	3.2333E-14	-15.5311002	13.4903476
CHTF8	25.6700551	-23.7196806	2.8784679	-8.24038391	1.7166E-16	2.0423E-14	-15.7653336	13.68989088
TFPI	45.1948426	-24.4925702	2.90465253	-8.43218594	3.3927E-17	4.4147E-15	-16.469458	14.35509721
ENO2	84.5156785	-24.9748632	2.95991423	-8.43769826	3.2365E-17	4.4147E-15	-16.4899273	14.35509721
UBL4A	29.8505654	-23.4944826	2.65699596	-8.84249843	9.3602E-19	2.0514E-16	-18.0287167	15.68795955
KEAP1	13.0377422	-22.7975182	2.54181762	-8.96898267	2.9927E-19	1.0142E-16	-18.5239431	15.99387054
C15orf52	52.8747154	-24.7100842	2.71397668	-9.10475183	8.6464E-20	4.0004E-17	-19.0631653	16.39789705
RGL2	43.149096	-23.0261213	2.51437665	-9.15778522	5.2973E-20	3.1512E-17	-19.2759428	16.50153014
IER5	25.8980288	-23.7375104	2.51083173	-9.4540427	3.2599E-21	3.3936E-18	-20.4867942	17.46934344

Supplementary Table 2 - Reagents

Antibodies

**Target**	**Source**	**Catalog #**	**Lot#**	**Use**	**Dilution**
KEAP1	Sigma	HPA005558	R004223	ICC and IHC	1:500
KEAP1	Abcam	ab227828	GR3332622-2	Western blot	1:1000
Tubulin	Sigma	T9026	047M4789V	Western blot	1:1000
NRF2	Cell Signaling Technologies	12721	10	Western blot	1:1000
Mouse PD-L1	Biolegend	155403	B281732	Flow cytometry	1:20
Human PD-L1	Biolegend	329705	B262099	Flow cytometry	1:20
Human HLA-A/B/C	Biolegend	311405	B327578	Flow cytometry	1:20
					Notes
mouse PD1	Leinco	P377	0321L255	Mouse treatment	Leinco Technologies Rat anti-Mouse CD279 (PD-1) (Clone 29F.1A12)
IgG control	Leinco	I1177	1021L325	Mouse treatment	Leinco Technologies Rat IgG2a Isotype Control; In Vivo PLATINUM; Clone 1-1

qRT-PCR

Target	Forward sequence (5'-3')	Reverse sequence (5'-3')
Keap1	CTGTGCCTCTATGAGCGTGCCA	TAGATGCCACTCGTCCCGCT
Nfe2l2	CCAGCACATCCAGACAGACACC	GCATCTTGTTTGGGAATGTGGGC
Nqo1	GCTGCTGTAGAGGCTCTGAAGA	AGTTCTTCGAGTCCTTCAGCTCA
Gclm	ACAAGACACAGTTGGAGCAGCT	CGGGTCATTGTGAGTCAGTAGCT
Txnrd1	TCGGTGAACACATGGAAGAACA	CCCTCTATGGTCTCCTCGCTGT
Gapdh	GTTGTCTCCTGCGACTTCA	GGTGGTCCAGGGTTTCTTA
H2-D1	AGTGGTGCTGCAGAGCATTACAA	GGTGACTTCACCTTTAGATCTGGG

Plasmids

LentiCRISPRv2 puro	addgene #98290
LentiGuide hygro	addgene #160090

sgRNA sequences (5'-3')

sgCtl	CCAATCTTGAACGTCATGTT
sgRosa	CTCCTTTAGATCATTCCTTG
sgKeap1 #1	TCAAATACGACTGCCCGCAG
sgKeap1 #2	CATGTACCAGATTGACAGCG
sgNfe2l2	GGTGGGATTTGAGTCTAAGG

Reagents

RPMI-1640 w/ GlutaMAX	Gibco	61870-036
EGF	Gibco	PHG031
bFGF	Gibco	PHG0261
b27 supplement	Gibco	17504-044
Polybrene	EMD Millipore	TR-1003-G
Mouse interferon gamma	Cell Signalling Technologies	39127
Human interferon gamma	Cell Signalling Technologies	80385
MycoAlert	Lonza	LT07-701
Citrate buffer (10X) heat induced antigen retrieval	Thermo scientific	AP-9003
DAB Substrate kit	Vector Laboratories	SK-4100
ImmPRESS Duet double staining polymer kit	Vector Laboratories	MP-7724
Bloxall blocking solution	Vector Laboratories	SP-6000
Envision System HRP labelled polymer anti rabbit	Dako	K4003
DPX mountant for histology	Sigma	6522
Antibody diluent	Dako	S0809
Citrate buffer 10X	Thermo scientific	AP-9003
RNeasy mini kit	Qiagen	74106
Superscipt IV VILO	Invitrogen	11756500
Power SYBRgreen PCR Master Mix	Applied Biosystems	4367659
Immobilon Crescendo Western HRP substrate	EMD Millipore	WBLUR
Immobilon Classico Western HRP substrate	EMD Millipore	WBLUC
RIPA buffer	Sigma	R0278

Supplementary Table 3 - NRF2 gene signature

Romero_NRF2_GENE_SIGNATURE

TXNRD1	MEGF9	ALDH3A1
TKT	ME1	AKR1C3
SRXN1	MCM10	AKR1C1
NQO1	MARS	AKR1B10
GCLC	MAP2	ADAM23
G6PD	MAFG	ABHD4
FTH1	LRP8	ABCG2
EPHX1	LRP12	ABCC2
DDC	KIAA0319	ABCC1
CBR3	IDH1	ABCB6
UCHL1	HTATIP2	
TXN	HSPA1B	
TSPAN7	HMOX1	
TRIM16	HGD	
TGFB2	GSR	
TFRC	GPX2	
TALDO1	GPC1	
SQSTM1	GLA	
SPP1	GCLM	
SORD	GALNT13	
SLC7A11	FZD7	
SLC6A6	FAM55C	
SLC38A6	F2RL2	
SFN	DOCK10	
SERPINE1	DGKG	
RRM2	DEGS1	
PRDX1	CYP4F11	
PPAT	CXCR7	
POPDC3	CPLX2	
PIR	CLN5	
PGD	CES1	
PCK2	CDKN2B	
PA2G4	CCND3	
P2RY6	CCDC77	
OSGIN1	CBR1	
NRCAM	CAMKK1	
NQO2	C1S	
NLN	C14ORF149	
NFE2L2	BLVRB	
NEIL3	ASPH	
NCF2	ASNS	
MSC	ASF1A	
MGST1	ALDH3A2	
