# Supplementary material for: Downregulation of KEAP1 in melanoma promotes resistance to immune checkpoint blockade
Source: NPJ Precis Oncol. 2023 Mar 2;7:25. doi: 10.1038/s41698-023-00362-3 (PMC9981575; doi:10.1038/s41698-023-00362-3)
Supplement: Supplementary file 2 — REPORTING SUMMARY [file 41698_2023_362_MOESM2_ESM.pdf]

nature portfolio

Corresponding author(s): Daniel A. Haber and Shyamala Maheswaran

Last updated by author(s): Jan 31, 2023

Reporting Summary

Nature Portfolio wishes to improve the reproducibility of the work that we publish. This form provides structure for consistency and transparency in reporting. For further information on Nature Portfolio policies, see our Editorial Policies and the Editorial Policy Checklist.

Statistics

For all statistical analyses, confirm that the following items are present in the figure legend, table legend, main text, or Methods section.

n/a	Confirmed	
☐	☒	The exact sample size (*n*) for each experimental group/condition, given as a discrete number and unit of measurement
☐	☒	A statement on whether measurements were taken from distinct samples or whether the same sample was measured repeatedly
☐	☒	The statistical test(s) used AND whether they are one- or two-sided<br>*Only common tests should be described solely by name; describe more complex techniques in the Methods section.*
☒	☐	A description of all covariates tested
☐	☒	A description of any assumptions or corrections, such as tests of normality and adjustment for multiple comparisons
☐	☒	A full description of the statistical parameters including central tendency (e.g. means) or other basic estimates (e.g. regression coefficient) AND variation (e.g. standard deviation) or associated estimates of uncertainty (e.g. confidence intervals)
☐	☒	For null hypothesis testing, the test statistic (e.g. *F*, *t*, *r*) with confidence intervals, effect sizes, degrees of freedom and *P* value noted<br>*Give P values as exact values whenever suitable.*
☒	☐	For Bayesian analysis, information on the choice of priors and Markov chain Monte Carlo settings
☒	☐	For hierarchical and complex designs, identification of the appropriate level for tests and full reporting of outcomes
☒	☐	Estimates of effect sizes (e.g. Cohen's *d*, Pearson's *r*), indicating how they were calculated

Our web collection on statistics for biologists contains articles on many of the points above.

Software and code

Policy information about availability of computer code

Data collection	No software was used to collect data.
Data analysis	Data analysis, statistical testing and visualization were conducted in Graphpad Prism (v9.1.3) and RStudio software (version 4.1.0; R Foundation for Statistical Computing). Quantification of IHC was performed using ImageJ2 (version 2.3.0/1.53q). Gene Set Enrichment Analysis was performed using GSEA software (Broad Institute; v4.2.3). For RNAseq analysis, RNA-SeQC (v2.3.5), Picard Tools (v2.18.24), STAR (2.6.1d), and DESeq2 (v1.36) softwares were used.

For manuscripts utilizing custom algorithms or software that are central to the research but not yet described in published literature, software must be made available to editors and reviewers. We strongly encourage code deposition in a community repository (e.g. GitHub). See the Nature Portfolio guidelines for submitting code & software for further information.

Data

Policy information about availability of data

All manuscripts must include a data availability statement. This statement should provide the following information, where applicable:

- Accession codes, unique identifiers, or web links for publicly available datasets
- A description of any restrictions on data availability
- For clinical datasets or third party data, please ensure that the statement adheres to our policy

RNA sequencing data of primary melanoma CTCs from this study were published by Hong et al. and are deposited into the Gene Expression Omnibus repository

under accession number GSE157745. Matched RNA and bisulfite sequencing data for cultured melanoma CTCs are deposited under accession number GSE218431. RNA sequencing and survival data for tumors from anti-CTLA4 naïve melanoma patients receiving anti-PD1 therapy are publicly available from Liu et al.

Human research participants

Policy information about studies involving human research participants and Sex and Gender in Research.

Reporting on sex and gender	The sex-based analysis of melanoma patients was not considered because the sample sizes are too small given the difficulty in isolating rare circulating tumor cells.
Population characteristics	In this study, patients with metastatic melanoma who were undergoing evaluation or treatment at the Massachusetts General Hospital Cancer Center were selected for study. Patients were at different ages.
Recruitment	Metastatic melanoma patients who were undergoing, or who were later selected for, treatment with immune checkpoint blockade were selected for this study.
Ethics oversight	Written informed consent was obtained from patients with metastatic melanoma undergoing evaluation or treatment at the Massachusetts General Hospital Cancer Center, and blood collections (10–15 mL) were performed as per IRB protocol (DF/HCC-0500), in accordance with the U.S. ethical guidelines.

Note that full information on the approval of the study protocol must also be provided in the manuscript.

Field-specific reporting

Please select the one below that is the best fit for your research. If you are not sure, read the appropriate sections before making your selection.

☒ Life sciences ☐ Behavioural & social sciences ☐ Ecological, evolutionary & environmental sciences

For a reference copy of the document with all sections, see nature.com/documents/nr-reporting-summary-flat.pdf

Life sciences study design

All studies must disclose on these points even when the disclosure is negative.

Sample size	For animal studies, sample size calculations were not predetermined, but previous studies using these models were used as reference. For the bioinformatic analysis, statistical methods were not used to determine the sample size for RNA-seq experiments. Sample size was determined based on our previous experience with single cell CTC analysis. For patient data, the sample size was determined by the total number of CTCs available for isolation.
Data exclusions	No data were excluded from the analysis.
Replication	Experiments to determine if KEAP1 affects α-PD-1 response were performed twice with two independent guide RNA, and all attempts at replication were successful. KEAP1 and NRF2 double knockout was performed once.
Randomization	All mice were randomized before the start of each experiment and randomly allocated in different groups before the start of each drug treatment.
Blinding	For animal experiments, the investigators were not blinded during treatment and tumor measurements.

Reporting for specific materials, systems and methods

We require information from authors about some types of materials, experimental systems and methods used in many studies. Here, indicate whether each material, system or method listed is relevant to your study. If you are not sure if a list item applies to your research, read the appropriate section before selecting a response.

Materials & experimental systems

n/a	Involved in the study	
☐	☒	Antibodies
☐	☒	Eukaryotic cell lines
☒	☐	Palaeontology and archaeology
☐	☒	Animals and other organisms
☒	☐	Clinical data
☒	☐	Dual use research of concern

Methods

n/a	Involved in the study	
☒	☐	ChIP-seq
☐	☒	Flow cytometry
☒	☐	MRI-based neuroimaging

Antibodies

Antibodies used	The following primary antibodies were used: KEAP1 (IHC, Sigma HPA005558, polyclonal, Lot #R004223, 1:500), KEAP1 (Western, Abcam ab227828, clone EPR22664-26, Lot# GR3332622-2, 1:1000), mouse PD-L1 (Flow, Biolegend, 155403, Lot B281732, 1:20), human PD-L1 (Flow, Biolegend, 329705, Lot B262099,1:20), Human HLA-A/B/C (Flow, Biolegend, 311405, Lot B327578, 1:20), Tubulin (Western, Sigma T9026, Lot# 047M4789V, 1:1000), NRF2 (Western, Cell Signaling Technologies 12721, Lot# 10, 1:1000). The following antibodies were used for mouse treatments: mouse PD-1 (Leinco P377, clone 29F.1A12, Lot# 0321L255, 100µg per dose), IgG control (Leinco I1177, clone 1-1, Lot# 1021L325, 100µg per dose).
Validation	For KEAP1 IHC experiments, the antibody was validated by the manufacturer for this application, and it was additionally tested on cell lines with or without genetic knockout. For western blotting, manufacturer suggested dilutions were used, and molecular weight was used for validation. For mouse experiments, established doses commonly used in the field were used.

Eukaryotic cell lines

Policy information about cell lines and Sex and Gender in Research

Cell line source(s)	Human derived CTC cultures (MEL-167) were generated from a patient with melanoma at the Massachusetts General Hospital Cancer Center. B16-OVA cells were obtained from Debattama Sen at the Massachusetts General Hospital Cancer Center.
Authentication	Authentication is not applicable for the human CTC-derived cultures or the B16-OVA cells.
Mycoplasma contamination	Cell lines were tested monthy for mycoplasma contamination using MycoAlert kit (Lonza)
Commonly misidentified lines (See ICLAC register)	No commonly misindentified lines were used in this study.

Animals and other research organisms

Policy information about studies involving animals; ARRIVE guidelines recommended for reporting animal research, and Sex and Gender in Research

Laboratory animals	8 week-old female C57BL/6J mice were purchased from The Jackson Laboratory.
Wild animals	No wild animals were used in this study.
Reporting on sex	Female mice were used. Male mice were not used because their higher aggression increases the likelihood of hemorrhaging of sub-cutaneous tumors.
Field-collected samples	There were no samples collected from the field in this study.
Ethics oversight	Animal studies were performed under the oversight of Massachusetts General Hospital Institutional Animal Care and Use committee (IACUC No. 2010N000006).

Note that full information on the approval of the study protocol must also be provided in the manuscript.

Flow Cytometry

Plots

Confirm that:

☒ The axis labels state the marker and fluorochrome used (e.g. CD4-FITC).

☒ The axis scales are clearly visible. Include numbers along axes only for bottom left plot of group (a 'group' is an analysis of identical markers).

☒ All plots are contour plots with outliers or pseudocolor plots.

☒ A numerical value for number of cells or percentage (with statistics) is provided.

Methodology

Sample preparation	All flow experiments were performed on cultured cells.
Instrument	Flow experiments were performed on a BD Fortessa X-20 instrument
Software	Flow data were analyzed using FlowJo 10.8.1
Cell population abundance	All cells within the single cell gate were included for flow plots showing HLA-A/B/C and PD-L1 analysis, which represented no less than 70% of all events.

Gating strategy

Gate 1 (Cells): SSC-A vs FSC-A
Gate 2 (single cells): FSC-H vs FSC-A

☒ Tick this box to confirm that a figure exemplifying the gating strategy is provided in the Supplementary Information.
